# Influence of Silver Nanoparticles (AgNPs) on Vegetative Growth and Concentrations of Nutrients and Phytohormones in Tomato

**DOI:** 10.3390/plants15030405

**Published:** 2026-01-28

**Authors:** Gabriela Abigail Guzmán-Báez, Libia I. Trejo-Téllez, Diego E. Navarro-López, Jorge L. Mejía-Méndez, Fernando Carlos Gómez-Merino

**Affiliations:** 1 Colegio de Postgraduados Campus Montecillo, Carretera México-Texcoco km 36.5, Montecillo, Texcoco 56264, Mexico; guzman.gabriela@colpos.mx (G.A.G.-B.); tlibia@colpos.mx (L.I.T.-T.); 2 Tecnológico de Monterrey, Escuela de Ingeniería y Ciencias, Epigmenio González 500, San Pablo, Santiago de Querétaro 76130, Mexico; diegonl@tec.mx (D.E.N.-L.); mejia.jorge@tec.mx (J.L.M.-M.)

**Keywords:** nanotechnology, nanobiotechnology, *Solanum lycopersicum*, silver nanoparticles, precision agriculture, One Health

## Abstract

This study examined the effects of applying silver nanoparticles (AgNPs; 0, 5 and 10 mg L^−1^) in a hydroponic system for seven days on growth parameters and on nutrient and phytohormone concentrations in two tomato cultivars, Vengador and Rio Grande. The results indicated that AgNPs at concentrations of 5 and 10 mg L^−1^ did not change leaf number, stem length, or fresh/dry biomass weight. In leaves of Vengador, P and K concentrations decreased, while Mg and S increased in response to AgNPs. In stems and roots, both P and K decreased. Zn concentrations increased in leaves, Mn in stems and roots. In leaves of Rio Grande, K, Mg, S, Cu and Mn concentrations increased, while P decreased in AgNP-treated plants, as compared to the control. In stems, N, S and Mn concentrations increased, but P, K, Ca, Mg and B decreased. In roots, P, K, Ca, Mg, Cu, Zn, Mn and B decreased, whereas S increased. Silver was only detected in roots of plants treated with AgNPs in both cultivars under study. In leaves of Rio Grande plants, kinetin concentrations decreased with AgNPs applications. In roots of Vengador, indole-acetic acid concentrations increased with 10 mg AgNP L^−1^; in Rio Grande, roots exhibited an increased concentration of gibberellic acid and abscisic acid in plants exposed to 5 mg AgNP L^−1^. The evidence retrieved from this work unveils the impact of metal-based NMs on the modulation of nutrient and phytohormone concentrations in a so important food crop such as tomato.

## 1. Introduction

Nanotechnology is a rapidly maturing discipline that has gained significant relevance in recent years, owing to its potential applications in diverse fields such as medicine, food, and agriculture. Nanomaterials (NMs) are found in sizes from 1 to 100 nm, and their physical and chemical features differ significantly from those of their bulk counterparts [1]. Depending on their chemical composition, NMs can be categorized into two main types: organic and inorganic. Inorganic NMs, such as metal-based nanostructures, are advantageous because their high surface area enables enhanced reactivity and interaction with biological components [2]. Additionally, they are convenient for various applications due to their thermal stability, biocompatibility, electrical conductivity, and high tailoring capacity. For agricultural applications, metal-based NMs are also particularly attractive since they can facilitate nutrient delivery, pesticide efficiency, disease resistance, stress tolerance, improved yield, and growth promotion of crops with economic importance such as coffee (*Coffea arabica* L.) [3], rice (*Oryza sativa* L.) [4], and tomato (*Solanum lycopersicum* L.) [5].

Tomato is one of the world’s most cultivated and popular vegetables, ranking second to the potato in terms of global production volume. A typical tomato plant is characterized by green and hairy leaves with a lobed or serrated margin and star-shaped flowers. A tomato fruit can vary in shape, size, and color, but it is considered an abundant source of vitamins (e.g., ascorbic acid, niacin, and alpha-tocopherol) [6], flavonoids (e.g., quercetin, kaempferol, and chlorogenic acid) [7], carotenoids (e.g., lycopene, β-carotene, and lutein) [8], and minerals (e.g., K, P, Mg, and Ca) [9], with significant implications in human health for preventing or treating many disorders [10]. Economically, the global tomato market has been valued in the billions of dollars, with China, India, the United States of America, and Mexico being among the largest producers [11].

Modern tomato cultivars result from extensive genetic breeding that has transformed small wild species, originally native to the Andes, into high-yielding global crops. This evolution has progressed through traditional selective breeding to highly precise molecular and genetic engineering techniques. Vengador is a cultivar that produces medium to large fruits with a deep red color and a round and flattened shape and exhibits resistance against *Fusarium* and *Verticillium* [12]. Rio Grande is another cultivar that produces smaller and oval-shaped tomatoes with a slightly tangy flavor and has moderate resistance to fungi pathogens. Both cultivars display significant market versatility and consistent production [13].

In tomato, AgNPs can modulate various biochemical and molecular phenomena. For example, their application can influence the expression of specific transporters involved in the uptake of essential nutrients aimed at mitigating stress conditions while optimizing multifaceted metabolic responses. H^+^-ATPase, potassium, and sulfate transporters are some of the proteins that can be regulated by AgNPs [14]. AgNPs can influence the expression of the membrane and vacuolar type of H^+^-ATPase, as well as that of isocitrate dehydrogenase and catalase [15]. By upregulating the expression of ethylene-inducing xylanase, peroxidase 51, and phenylalanine ammonia lyase activity, AgNPs induce defense responses [16]. AgNPs can modulate plant development by modifying jasmonic and gibberellic acid-mediated signaling pathways, as well as microtubule arrangement in wild-type and mutant Arabidopsis seedlings [17].

Considering the beneficial roles of AgNPs in growth promotion, plant development, and nutritional status of crops, the objective of this study was to evaluate the effect of commercially available AgNPs on vegetative growth and on nutrient and phytohormone concentration in tomato plants. This was hypothesized considering that plant responses to AgNPs vary significantly based on both interspecific (different species) and intraspecific (different genotypes or cultivars of the same species) factors, as well as on the unique physical and chemical features of the evaluated nanostructures. We analyzed the responses of two cultivars of tomato, Vengador and Rio Grande, established in a hydroponic system. We measured the number of leaves, stem length, and the fresh and dry biomass weight of the leaves, stems, and roots, as well as the concentrations of nutrients and phytohormones in different plant tissues.

## 2. Results

### 2.1. Growth and Biomass Production

**Figure 1:**
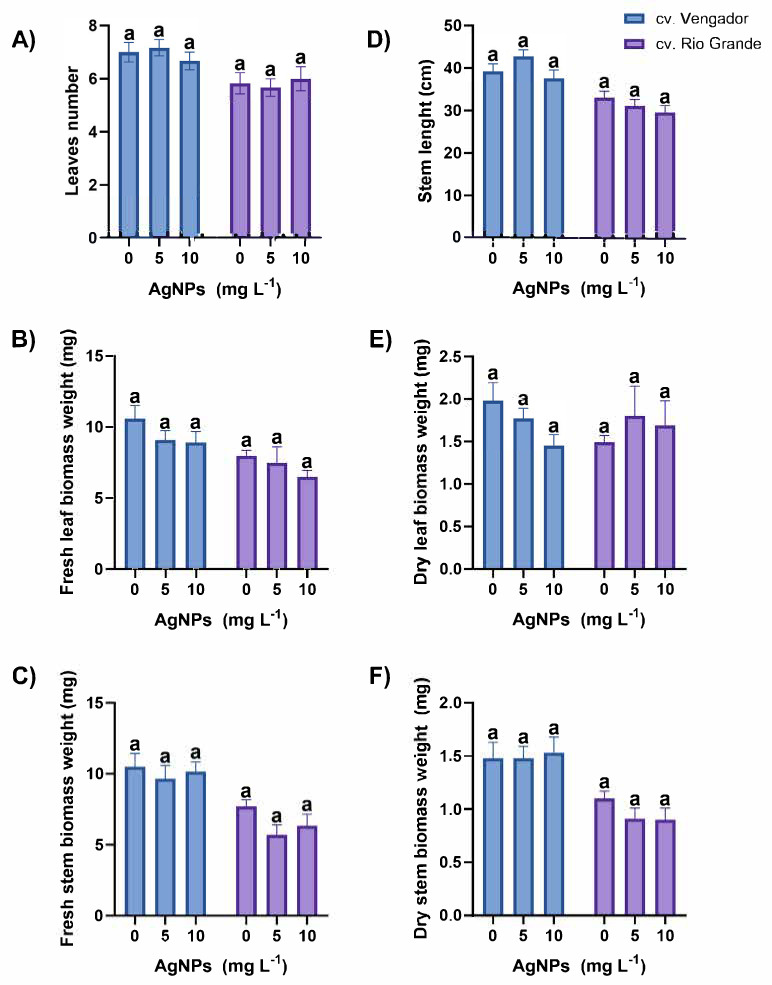
Growth variables in 45-day-old tomato (*Solanum lycopersicum* L.) plants cv. Vengador and Rio Grande exposed to different concentrations of AgNPs for seven days. (**A**) Leaf number; (**B**) Fresh leaf biomass weight; (**C**) Fresh stem biomass weight; (**D**) Stem length; (**E**) Dry leaf biomass weight; (**F**) Dry stem biomass weight. The data are represented as the mean ± standard deviation from five independent experiments (*n* = 5). Similar letters above the columns for each variable analyzed indicate that no significant differences among treatments tested were found (Tukey; *p* > 0.05).

The statistical analysis of data revealed no effects of the treatments tested on growth and biomass production of tomato plants exposed to different concentrations of AgNPs (Figure 1). Regardless of the absence of significant differences among treatments, there are some interesting data to be observed. In general, Vengador plants displayed higher means as compared to Rio Grande. For instance, Vengador plants treated with 5 and 10 mg AgNPs L^−1^ produced 7.17 ± 0.31 and 6.67 ± 0.33 leaves, respectively. Instead, Rio Grande produced 5.83 ± 0.40 and 6.00 ± 0.45 leaves when exposed to 5 or 10 mg AgNPs L^−1^, respectively (Figure 1A).

In leaves of Vengador plants, fresh biomass weight was 9.09 ± 0.66 and 8.91 ± 0.79 mg when exposed to either 5 or 10 mg AgNPs L^−1^. In Rio Grande, the application of AgNPs at either 5 or 10 mg AgNPs L^−1^ resulted in 7.49 ± 1.13 and 6.51 ± 0.45 mg, respectively (Figure 1B). Similarly, in stems of Vengador, fresh biomass weight resulted in 9.66 ± 0.93 and 10.17 ± 9.68 mg in plants exposed to 5 or 10 mg AgNPs L^−1^, respectively. In Rio Grande, fresh biomass of stems was 5.68 ± 0.71 and 6.34 ± 0.82 mg in plants exposed to 5 or 10 mg AgNPs L^−1^, respectively (Figure 1C).

The application of 5 and 10 mg AgNPs L^−1^ caused a stem length of 42.74 ± 1.54 and 37.57 ± 1.96 cm in Vengador. Similar results were obtained when the same concentrations were evaluated in Rio Grande, with a stem length of 31.08 ± 1.48 cm (5 mg AgNPs L^−1^) and 29.51 ± 1.65 cm (10 mg AgNPs L^−1^) (Figure 1D).

In leaves of Vengador plants, application of AgNPs at 5 and 10 mg L^−1^ resulted in 1.77 ± 0.12 and 1.45 ± 0.13 mg of dry biomass, respectively. At the same concentrations tested, leaves of Rio Grande plants reached 1.80 ± 0.35 and 1.69 ± 0.29 mg of dry biomass (Figure 1E). Stems of Vengador weighed 1.48 ± 0.11 and 1.53 ± 0.15 mg at 5 and 10 mg AgNPs L^−1^, respectively. Stems of Rio Grande presented 0.91 ± 0.10 and 0.90 ± 0.11 mg of dry biomass weight after the application of 5 and 10 mg AgNPs L^−1^ (Figure 1F).

### 2.2. Concentrations of Macronutrients and Micronutrients

AgNP treatments differentially affected the concentrations of macronutrients in leaves, stems, and roots of tomato plants cv. Vengador (Table 1). The concentrations of N and Ca were not influenced by the AgNPs in any of the tissues analyzed. In plants exposed to 5 and 10 mg AgNPs L^−1^, foliar P concentrations were 10.8 and 32.9% lower, respectively, as compared to the control. Likewise, in these treatments, decreases in foliar K concentration of 13.8 and 28.4% were seen compared to the control. Foliar Mg concentration showed a positive relationship with AgNP concentration, resulting in increases of 14 and 28.5% at doses of 5 and 10 mg AgNPs L^−1^ compared to the control. Interestingly, a 154% increase in S concentration in leaves was observed compared to the control when plants were treated with 5 mg AgNPs L^−1^

In stems, treatments with 5 and 10 mg AgNPs L^−1^ resulted in a 27.5 and 25.0% decrease in P concentration, respectively, compared to the control. A dose of 10 mg AgNPs L^−1^ also reduced K concentration in stems by 21.5%, while Ca, Mg and S concentrations were not impacted by the treatments. 

In roots, the dose of 10 mg AgNPs L^−1^ reduced the concentrations of P, K, and Mg by 28.7%, 21.5%, and 70.3%, respectively, in all cases compared to the control. Concentrations of N, Ca and S were not impaired by AgNP applications in root tissues.

Micronutrient concentrations were also differentially affected by the AgNP concentrations tested (Table 2). Concentrations of Fe, Cu, and B were not influenced by AgNP treatments in leaves, stems, and roots, nor was Zn concentration in stems and roots. In leaves, Zn concentration increased with the application of 5 and 10 mg AgNPs L^−1^ by 24.7 and 61.6% compared to the control. Likewise, Mn concentration in stems was 116.4% higher with 10 mg AgNPs L^−1^ compared to the control. In roots, the high dose of AgNPs reduced Mn concentration by 91.4%. The presence of silver was only detected in roots when AgNPs were applied; no significant differences were observed among treatments, although a higher concentration was seen with the addition of 10 mg AgNPs L^−1^.

In leaves of Rio Grande plants, N and Ca were not affected by the treatments tested. P concentration was reduced by 13% with a dose of 10 mg AgNPs L^−1^ compared to the control. In contrast, foliar K, Mg, and S concentrations were higher in plants treated with AgNPs (Table 3). Noteworthy increases were observed in K, by 34.8%, and S, by 154%, with a dose of 5 mg AgNPs L^−1^; the 10 mg AgNP L^−1^ dose increased Mg concentration by 28.4% compared to the control. In stems of cv. Rio Grande, it is noteworthy that the low and high doses of AgNPs increased N concentration by 57.1 and 85.8%, and S concentration by 797.8 and 236.6%, as compared to the control. Conversely, 5 mg AgNPs L^−1^ reduced P, K, Ca, and Mg concentrations by 30.9, 49.2, 22.6, and 57.4%, respectively, compared to the control. In roots, detrimental effects were observed on the concentrations of P, K, Ca, and Mg of Rio Grande plants treated with AgNPs, with average reductions of 57.8, 96.0, 53.9, and 84.4%, respectively, compared to the control. In contrast, treatment with 5 and 10 mg AgNPs L^−1^ increased the concentration of S in roots by 164.2 and 51.7%, respectively, compared to the control. N concentration was not affected by AgNPs treatments. 

As noted in Table 4, there was no significant effect of treatments on Fe concentration in stems and roots of cv. Rio Grande, nor on Zn concentrations in stems.

In leaves of this cultivar, doses of 5 and 10 mg AgNPs L^−1^ increased Cu concentration by 38.1% and 49.8%, respectively, compared to the control, while the dose of 5 mg AgNPs L^−1^ increased Mn concentration. In stems, the application of 5 and 10 mg AgNPs L^−1^ increased Mn concentration by 28.3% and 57.7%. In roots, AgNPs reduced the concentrations of Cu, Zn, Mn, and B, in all cases compared to the control. Ag was only found in roots of plants treated with AgNPs treatments (Table 4).

### 2.3. Concentrations of Phytohormones

**Figure 2:**
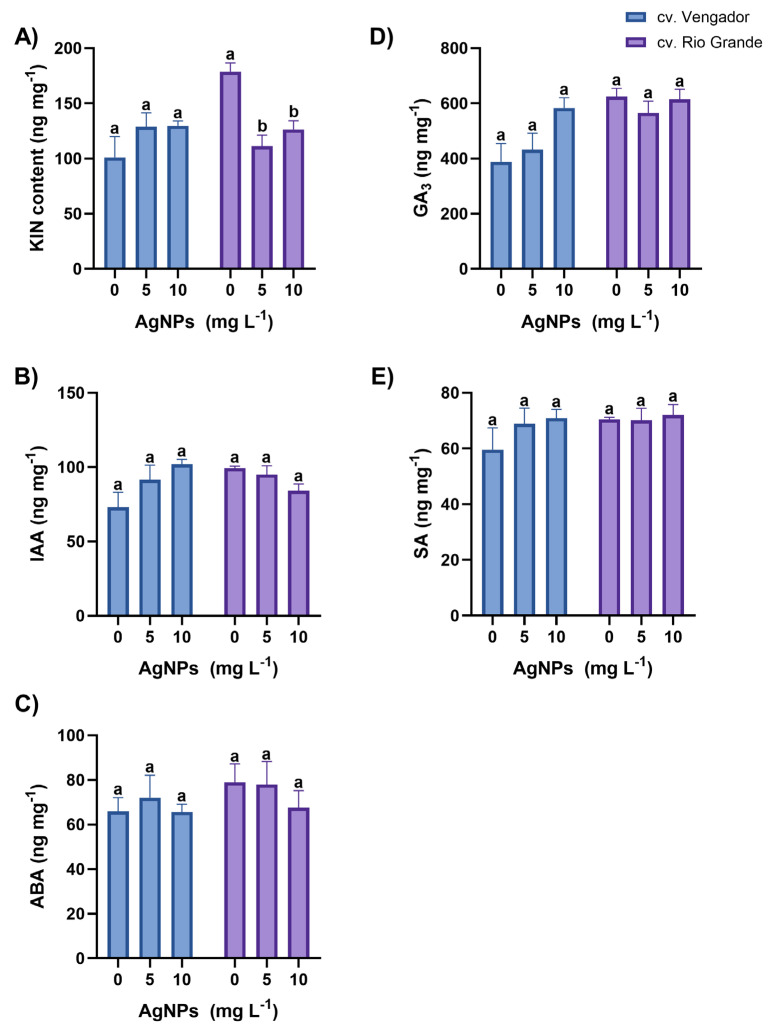
Phytohormone concentrations in the leaves of 45-day-old tomato (*Solanum lycopersicum* L.) plants cv. Vengador and Rio Grande exposed to different concentrations of silver nanoparticles (AgNPs) for seven days. (**A**) Kinetin (KIN) concentration; (**B**) Indole-acetic acid (IAA) concentration; (**C**) Abscisic acid (ABA) concentration; (**D**) Gibberellic acid (GA_3_) concentration; (**E**) Salicylic acid (SA) concentration. Different letters above the columns for each phytohormone analyzed indicate significant differences among treatments tested (Tukey; *p* ≤ 0.05). Data are mean ± SD. DBW: dry biomass weight. *n* = 5.

In leaves of Vengador plants, concentrations of the phytohormones kinetin (KIN), indole-acetic acid (IAA), abscisic acid (ABA), gibberellic acid (GA_3_), and salicylic acid (SA), did not differ among treatments tested. In Rios Grande, a significant reduction in KIN concentration was observed in leaves of plants treated with AgNPs (in both concentrations), as compared to the control. The magnitude of this reduction was of 37.8% and 29.5% with 5 and 10 mg AgNPs L^−1^, respectively, compared to the control (Figure 2). The HPLC chromatograms from the analysis are included in the Supplementary Materials.

**Figure 3:**
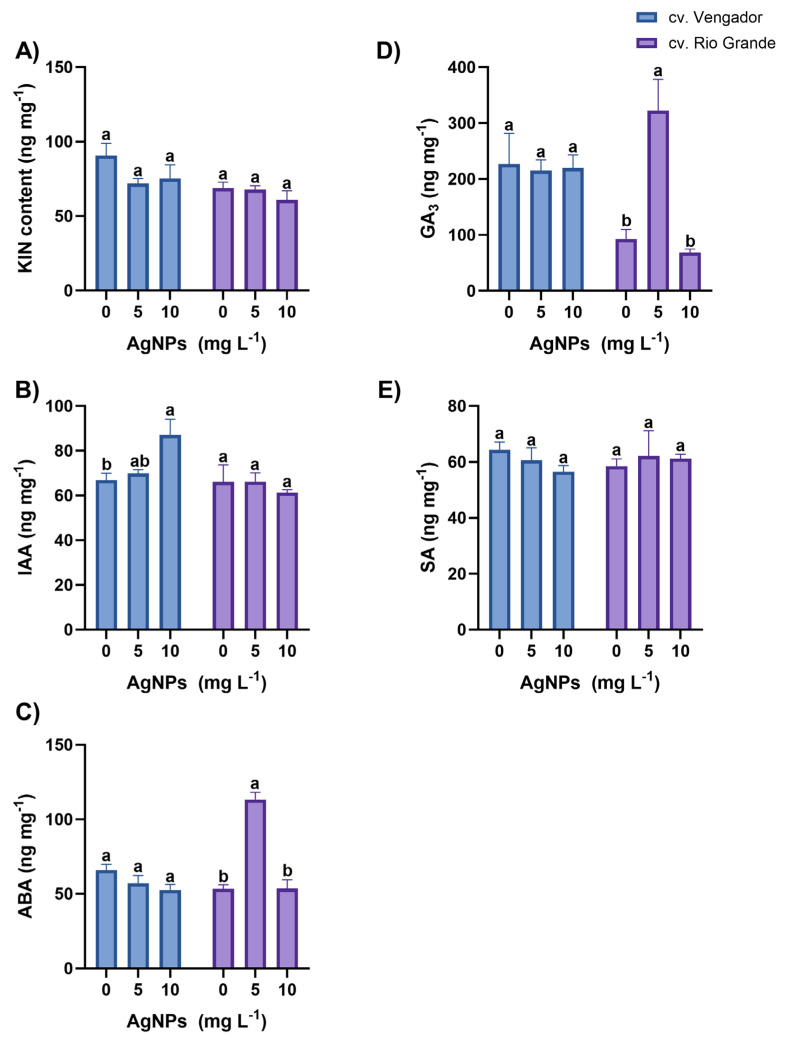
Phytohormone concentrations in the roots of 45-day-old tomato (*Solanum lycopersicum* L.) plants cv. Vengador and Rio Grande exposed to different concentrations of silver nanoparticles (AgNPs) for seven days. (**A**) Kinetin (KIN) concentration; (**B**) Indole-acetic acid (IAA) concentration; (**C**). Abscisic acid (ABA) concentration; (**D**) Gibberellic acid (GA_3_) concentration; (**E**) Salicylic acid (SA) concentration. Different letters above the columns for each phytohormone analyzed indicate significant differences among treatments tested (Tukey; *p* ≤ 0.05). Data are mean ± SD. DBW: dry biomass weight. *n* = 5.

In roots of Vengador plants, the concentrations of KIN, ABA, GA_3_, and SA were not affected by the application of AgNPs. In this cultivar, IAA concentration in roots increased by 30.1% when plants were treated with 10 mg AgNPs L^−1^ compared to the control (Figure 3). In roots of Rios Grande plants, AgNP treatments did not affect the concentrations of KIN, IAA, and SA. Interestingly, ABA increased by more than 100% in roots of plants treated with 5 mg AgNPs L^−1^ as compared to the control and to the plants exposed to 10 mg AgNPs L^−1^. A significant increase in GA_3_ concentration, of almost three-fold, was also observed in roots of Rio Grande plants exposed to 5 mg AgNPs L^−1^ as compared to the control and to the plants exposed to 10 mg AgNPs L^−1^ (Figure 3).

### 2.4. PCA of Phytohormonal Profiles in Cultivars Vengador and Rio Grande

**Figure 4:**
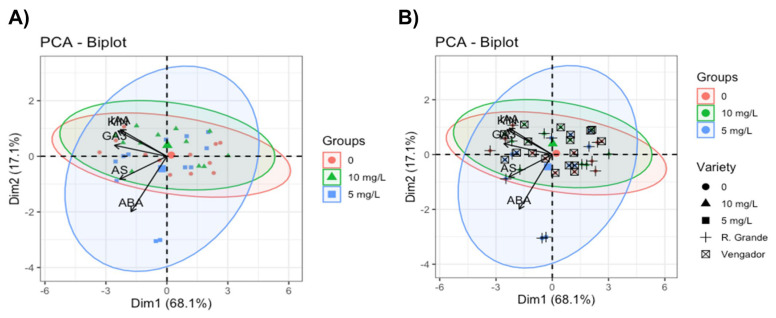
Principal Component Analysis (PCA) of phytohormones in tomato (*Solanum lycopersicum* L.) plants cv. Vengador and Rios Grande. (**A**) PCA of the phytohormone profile in tomato under different concentrations of AgNPs (0, 5 and 10 mg L^−1^). PC1 (68.1%) mainly separates increases in KIN, GA_3_ and IAA, while PC2 (17.1%) is associated with AS and ABA. (**B**) PCA of the hormonal profile differentiating cultivars (Rio Grande and Vengador) and AgNP concentration.

The Principal Component Analysis (PCA) performed (Figure 4A,B) revealed that 85.2% of the total data variation can be explained using only two components (PC1 = 68.1%, PC2 = 17.1%). PC1 was strongly associated with KIN, GA_3_, and IAA, indicating that this component describes a physiological development. PC2, primarily influenced by ABA and AS, constituted a stress signaling axis. The sample distribution showed a clear separation between the AgNP treatments. Plants in the control (0 mg AgNPs L^−1^) treatment clustered around negative PC1 values, reflecting basal growth hormone profiles. The 5 mg AgNPs L^−1^ concentration shifted the samples toward more positive PC1 values, indicating coordinated activation of KIN, GA_3_, and IAA. In contrast, 10 mg AgNPs L^−1^ produced greater dispersion, with shifts toward mixed values in PC1 and PC2, suggesting a heterogeneous physiological response, partially associated with increases in ABA.

The cultivars showed differentiated behaviors: Vengador presented more stable profiles centered in the PCA space, while Rio Grande exhibited greater variability and shifts toward negative PC1 and positive PC2 values, indicating greater sensitivity to AgNPs, especially stress-related hormones. Differences among tissues were also clear: leaves clustered in positive PC1 ranges, while roots were in negative zones, consistent with their lower concentration of hormones influencing growth.

**Figure 5:**
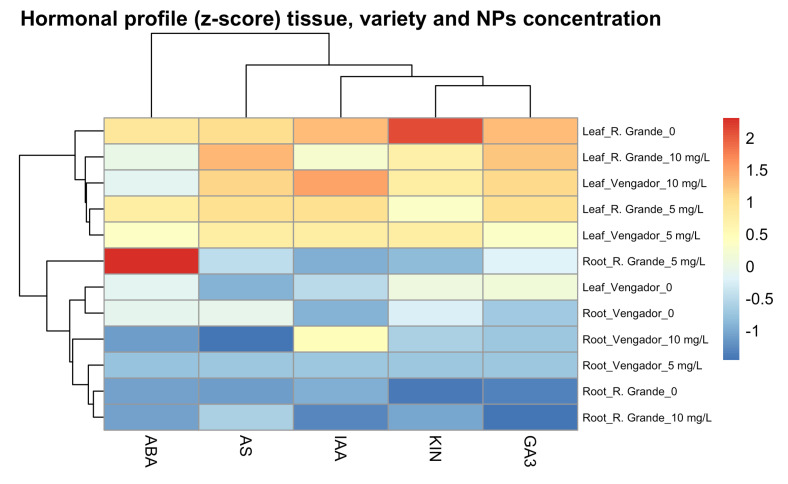
Heatmap of the standardized hormonal profile (z-score) in leaves and roots of two tomato (*Solanum lycopersicum* L.) cv. Vengador and Rio Grande exposed to different concentrations of AgNPs (0, 5 and 10 mg L^−1^).

Hormonal profile analysis (z-scores) revealed clear differentiation among tissues, cultivars, and AgNP concentrations (Figure 5).

Hierarchical clustering clearly separated leaf and root samples, indicating a structural contrast in phytohormone accumulation. Leaf samples showed positive values for IAA, KIN, and GA_3_, suggesting a physiological state associated with greater growth activity. At the same time, roots exhibited negative values for these same hormones, reflecting a more conserved and less dynamic profile. Within each cultivar, specific responses to AgNP concentration were observed. In Río Grande, leaves treated with 5 mg AgNPs L^−1^ and 10 mg AgNPs L^−1^ showed similar increases in growth-promoting hormones (IAA, KIN, GA_3_), resulting in their clustering within the same group. However, in the roots of this cultivar, the 5 mg AgNPs L^−1^ treatment resulted in a marked increase in ABA, setting it apart from the other samples, potentially due to this stress-related hormonal signal. In contrast, Vengador plants showed a more uniform pattern in both leaves and roots, with moderate variations in all hormones and no extreme peaks, resulting in a more homogeneous distribution within the dendrogram.

## 3. Discussion

AgNPs have been shown to stimulate plant metabolism and growth. Although the mechanisms underlying the accumulation of NPs or other metallic components in plant tissues are not fully understood, factors such as particle size, species, dosage, application methods, and the presence of additional substances, such as capping agents, are known to have a significant influence on the final concentration [18,19].

In tomato, scientific evidence has suggested that green synthesized AgNPs with an aqueous seed extract of the African juniper (*Juniperus procera* Hochst. ex Endl.) improve the seed germination rate, total protein and soluble sugar, enzyme activity, and phenolic compound concentration [20]. Other reports involving green synthesis have revealed that AgNPs can improve tomato development and yield and enzyme activity (e.g., superoxide dismutase, catalase, and phenylalanine ammonia-lyase) while conferring protection against *Alternaria solani* by diminishing disease severity index and incidence [21]. Contrary to green synthesized approaches, other studies have demonstrated that AgNPs obtained by a chemical method with sodium citrate can enhance the germination and vigor index of seven varieties of tomato within the range of 25–100 mg mL^−1^ [22]. Despite the wide evidence about the implications of AgNPs obtained from different approaches, a limited number of studies have been conducted considering commercially available formulations.

In this work, commercially available AgNPs (Agrovit^TM^) were evaluated. Regarding their physical and chemical features, the employed AgNPs have been reported to be spherical, with an average diameter of 35 ± 15 nm, a negative surface charge (-15 mV), a surface plasmon resonance at 420 nm, and a coating of 18.8% polyvinylpyrrolidone [23]. These AgNPs induce differentiated postharvest biosynthesis of secondary metabolites of pharmaceutical interest [24], as well as the accumulation of phenolic-related compounds in prickly pear [*Opuntia ficus-indica* (L.) Mill] [25] and *in vitro* regeneration of vanilla (*Vanilla planifolia* Jacks. ex-Andrews) [26]. Despite this, the effects of commercially available AgNPs on the physiological, biochemical, and molecular responses of different tomato cultivars have not been fully elucidated.

**Scheme 1:**
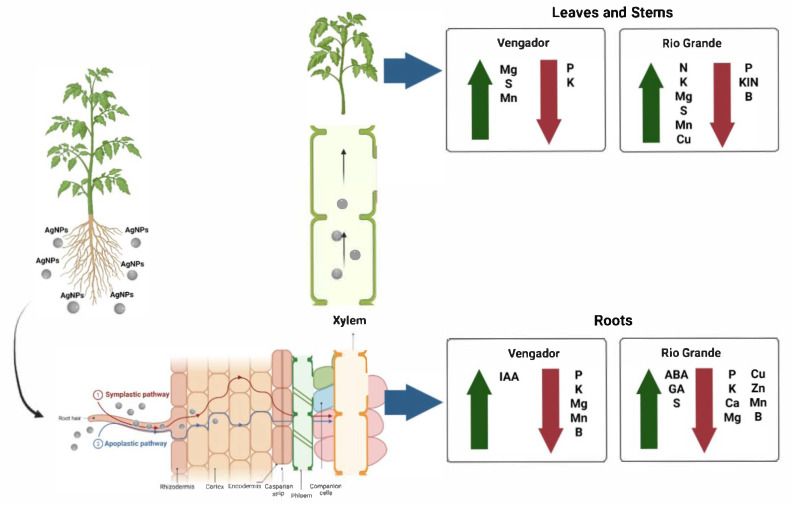
Schematic representation of the key findings of the effects of AgNPs on the concentrations of macronutrients, micronutrients, and phytohormones in the underground part (roots) and the aboveground part (leaves and stems) of tomato plants cv. Vengador and Rio Grande. Increases in means of traits measured are indicated with green upward arrows, whereas decreases are represented by red downward arrows. Created in BioRender version 2026 (https://BioRender.com/e2th5hj).

Scheme 1 summarizes the key findings and main message of our study, highlighting the effects of AgNPs on the concentrations of macronutrients, micronutrients, and phytohormones in the underground part (roots) and the aboveground part of the plant (leaves and stems).

The capacity of metal-based nanostructures to improve physiological variables of crops must be evaluated to identify optimal concentrations at which nanomaterials can exert stimulant effects of interest in agriculture. Additionally, such experiments are required to assess the potential guidelines to tailor the application of emerging alternatives to promote growth and productivity of different crops. Under our experimental conditions, growth variables of cv. Vengador and Rio Grande were not influenced by the treatments (Figure 1), but concentrations of some essential elements and phytohormones were indeed affected.

For agricultural applications, the nutrient concentration of crops upon exposure to nanomaterials must be investigated since it reflects their potential impact for improving the overall health benefits for consumers, resulting in public health promotion and alleviation of malnutrition. In the same sense, it must be analyzed to retrieve information about the capability of nanostructures to act as biofortification agents when considered in agricultural practices for meeting consumer demand.

Silves nanoparticles have been demonstrated to display both stimulatory and inhibitory effects in plants. For instance, in rice seedlings exposed to AgNPs caused significant physiological and molecular changes, oxidative stress and also resulted in the induction oxidative stress tolerance mechanisms [27]. In radish (Raphanus sativus), AgNPs significantly affected growth, nutrient content and macromolecule conformation, with unknown consequences for human health [28]. In *Arabidopsis thaliana*, the application of CeO_2_ NPs or In_2_O_3_ NPs differentially affected plant biomass, growth, chlorophyll, anthocyanin, MDA, root elongation and expression of genes central to the stress response such as the sulfur assimilation and glutathione biosynthesis pathway, directly related to the decreased phytotoxicity relative to NP treatment, in a dose-dependent fashion [29]. Interestingly, AgNPs may also help plants grow, alleviate stresses, and fight against pathogens [30].

Our results demonstrated that AgNPs exerted differential effects on nutrient and phytohormone concentrations depending on the cultivar, tissue analyzed and AgNPs dose applied. In Vengador plants, AgNPs did not affect N and Ca levels but reduced P in leaves, stems or roots. In leaves and stems, P concentration decreased with both 5 or 10 mg AgNPs L^−1^, while in roots, P decreased in plants exposed to 10 mg AgNPs L^−1^. It is well documented that P deficiency renders major detrimental impacts in plants, including stunted development; altered root architecture, with a concomitant increased root-to-shoot ratio; increased lateral roots and root hairs, impaired photosynthesis, and metabolic adjustments [31,32]. Reduced concentration of K and increased levels of Mg and S were also observed in leaves. In stems, AgNPs did not affect Mg and S, in addition to N and Ca. In roots, the application of 10 mg AgNPs L^−1^ reduced Mg concentration, whereas S was not affected, in addition to N and Ca.

Nutrient concentrations in Vengador and Rio Grande may differ between the two cultivars as a result of their intrinsic genetic background, which may significantly influence how plants acquire, move, and metabolize nutrients. These differences result from a complex trait governed by multiple interacting genetic factors across three main stages: uptake, transport (translocation), and assimilation. Genetic variation among cultivars leads to distinct physiological and molecular responses to nutrient availability, such as varying expression levels of transporter genes and proteins or differences in root architecture [33]. Importantly, high doses of AgNPs can impair the activity of these transporters [28,34].

In leaves of Río Grande plants, AgNPs did not affect N and Ca concentrations, while P decreased with 10 mg AgNPs L^−1^. Interestingly, K and Mg increased with both doses of AgNPs tested, while S increased with 5 mg AgNPs L^−1^. In stems, N concentration increased with AgNP application at both doses tested. Also in stems, concentrations of P. K, Ca and Mg decreased at 5 mg AgNPs L^−1^, whereas S increased at 10 mg AgNPs L^−1^ (Table 3). It is worth noting that the increase in N concentration in stems of Rio Grande is unique, since no other tissue was found to display increased concentrations of this vital element in response to AgNPs under our experimental conditions. Plants respond to metal stress by accumulating soluble nitrogen compounds that regulate metabolic processes and cellular osmotic potential [35]. Instead, reductions of P concentrations were observed in both stems and roots, which can be attributed to a potential decrease in the activity of P transporters [36]. Ag^+^ ions interfere with K channels, leading to increased gene expression and enzymatic activity [37]. Root Ca decreased with 5-10 mg AgNPs L^−1^, as AgNPs block Ca transport and binding proteins [38]. Mg increased in leaves but decreased in stems and roots with AgNPs, which has also been reported in banana (*Musa* spp.) [39]. S increased in all tissues, possibly due to an upregulation of genes encoding sulfur transporters and antioxidant enzymes [40,41].

Regarding micronutrients in Vengador, concentrations of Fe, Cu, and B were not affected by AgNPs in any of the three tissues analyzed. In leaves, Zn significantly increased with AgNPs applications. In stems, Mn increased at 10 mg AgNPs L^−1^. In roots, Mn dramatically decreased with 10 mg AgNPs L^−1^. In Rio Grande, micronutrient concentrations were more affected as compared to Vengador. In leaves, Cu and Mn increased, while Fe, Zn and Mn were not statistically different to the control. In stems, Fe, Cu and Zn were not statistically different to the control, while concentration of Mn increased and that of B decreased in AgNPs-treated plants as compared to the control (Table 4). The observed increases in micronutrient concentrations could be attributed to a potential induction of ion transporter activity [42], whereas the decrease in the concentrations of some of them could be a consequence of the inhibition in the activity of transporters in response to AgNPs [43,44,45].

Silver was detected in plants treated with 10 mg AgNPs L^−1^ (Table 2 and Table 4). AgNPs are prone to be absorbed by plant roots; the quantity and rate of absorption and consequent transformation will depend on their size, shape, and surface charge [46]. Furthermore, uptake of AgNPs is also concentration dependent; consequently, high concentrations and accumulation rates are observed upon exposure to high concentrations of AgNPs. The effects of AgNPs in nutrient concentrations can also be attributed to their capability to interfere in nutrient cycling among crops due to their interaction and influence with microbial soil activity [47]. As a matter of fact, nutrient levels arise from biochemical and molecular changes in crops. Some of them encompass phytohormone levels, photosynthetic activity, expression of transport genes and proteins, and enzymatic activity of proteins involved in nutrient uptake, transport, assimilation, and recycling.

Phytohormones are small molecules that regulate growth, development, and signal transduction pathways in response to stress [48]. Similar to nutrients, phytohormones may contribute to optimizing agricultural practices aimed at enhancing productivity while minimizing negative impacts on the environment. Phytohormones include cytokinins (CKs: BAP, KIN, ZEA), gibberellins (GA_1_, GA_2_, GA_3_), auxins (AUXs; IAA, IBA, NAA), brassinosteroids (BR; BL, CS, 24-EpiBL), salicylic acid (SA), abscisic acid (ABA), and jasmonates (JAs; JA, MeJA, JA-Ile). The synthesis of each one may depend on the production of the others, causing them to interact to regulate various physiological processes and signaling cascades [49], which can promote plant health and productivity by enhancing cell elongation, nutrient mobilization, fruit development, defense mechanisms, and regulation of photosynthesis [50]. There are various factors that can upregulate or downregulate the activity of phytohormones, including drought, salinity, cold or heat, nutrient deficiencies, and metal toxicity. Current approaches for mitigating the consequences of such factors in the production of phytohormones involve genetic engineering and biotechnological applications [51]. Still, despite their effectiveness, the use of both approaches has been associated with regulatory hurdles, unintended phenotypic changes or aberrations in metabolic pathways, high costs, and biodiversity concerns. Contrary to this, nanotechnology has paved the way for innovating crop resilience and productivity by yielding cost-effective alternatives and enhancing targeted delivery, adaptive responses, and plant growth while minimizing waste and potential side effects [52]. Importantly, nutrients and phytohormones are interconnected within plant metabolism, and their optimization may contribute to better plant performance in terms of growth, development, yield and responses to environmental cues.

Accordingly, this study aimed at determining the impact of AgNPs on growth and on nutrient and phytohormone concentrations in tomato plants at an early vegetative stage. In order to visualize potential effects of genotypes, we tested two tomato cultivars: Vengador and Rios Grande. Plant growth was not significantly affected by the treatments tested, which can be attributed to the short period of time of the experiment (just seven days). Nonetheless, as discussed above, nutrient concentrations were differentially affected by AgNPs, depending on the tissue analyzed and the genotype evaluated. Changes in phytohormone concentrations were observed in leaves of both cultivars. However, in roots, the application of 10 mg AgNPs L^−1^ increased IAA levels in Vengador plants. In Arabidopsis, exposure to 100 mg AgNPs L^−1^ induced overexpression of genes related to IAA synthesis, leading to suppression of lateral root development [53]. In Rios Grande, KIN concentration decreased in plants exposed to AgNPs, which may be correlated to the accumulation of ABA. Abscisic acid can activate enzymes involved in CK degradation and prevent the expression of genes that determine CK biosynthesis, which in turn may help improve plant resistance to metal stress [54]. In roots, the exposure to 5 mg AgNPs L^−1^ increased the concentration of GA_3_ (Figure 3), which can enhance the content of endogenous amino acids and promote greater tolerance to stress by increasing the activity of key enzymes involved in nutrient uptake and water absorption, thus stimulating mitotic activity, and biochemical and metabolic processes [55]. In Rio Grande, the application of 5 mg AgNPs L^−1^ increased the concentration of ABA in roots. Likewise, in Arabidopsis, the application of ZnO-NPs increased ABA concentrations in roots [56]. ABA is a phytohormone involved in sensing and responding to toxic metal stress, and the presence of AgNPs induces the expression of the NCED3 gene, which encodes a key enzyme for ABA biosynthesis, in plants exposed to silver nanoparticles [53].

The biological impact of AgNPs in different living organisms is driven by their size, shape, and surface charge. However, the biochemical and molecular mechanisms that explain these responses in plants is limited. In general, small NPs (<40 nm) can be readily absorbed by plant roots [57], thus potentially leading to highly effective interactions within root cells, with concomitant robust responses that can result in increased phytohormone biosynthesis. The surface charge of AgNPs can determine their interactions with positively or negatively charged molecules within the cell, possibly resulting in enhanced phytohormone production [17]. Negative-surface-charge nanostructures can improve interactions with cell membrane receptors involved in hormonal signaling and upregulate activation of pathways that stimulate hormone synthesis [58]. Accordingly, it can be hypothesized that similar events occurred during the application of AgNPs to the evaluated cultivars in this work, but further approaches are required to unveil the molecular mechanisms behind our findings reported here.

To summarize, here, we report that during a time span of seven days during which tomato plants were under treatment, AgNPs did not affect their growth or biomass production. Despite the short period of time of the experiment, silver nanoparticles did affect both nutrient and phytohormone concentrations in a different manner depending on the concentration of AgNPs tested, the tissue analyzed, and the genotype evaluated. Interestingly, the positive effects were greater than the negative ones. Since this study was carried out during the early vegetative stage of tomato plants, further studies are needed to elucidate the potential of this technology to promote fruit set, quality, and uniformity. Furthermore, additional studies are required for determining the possible accumulation of AgNPs and related metal-based nanostructures in edible parts of crops by employing cutting-edge technologies such as artificial intelligence and machine learning, advanced spectroscopic and mass spectrometry techniques, nanotechnology-enabled sensors, and blockchain for enhanced traceability and risk assessment.

By utilizing nanoparticles, we can achieve sustainable crop productivity and empower plants to thrive in challenging environments. They provide innovative solutions in crop protection, nutrient delivery, and soil remediation, ultimately enhancing plant growth, improving yields, and mitigating environmental stressors.

## 4. Materials and Methods

**Scheme 2:**
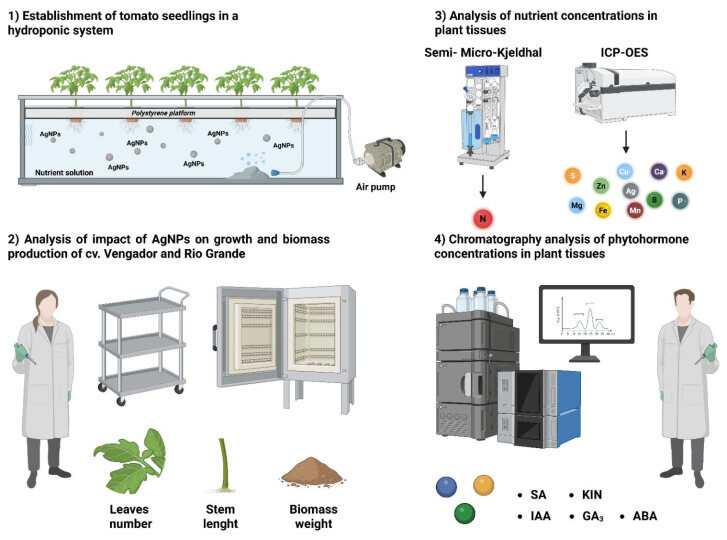
Experimental workflow followed in this study consisting of four main stages: (**1**) Establishment of tomato *(Solanum lycopersicum* L.) seedlings in a hydroponic system; (**2**) Analysis of impact of AgNPs on growth and biomass production of cv. Vengador and Rio Grande; (**3**) Spectrometry analysis of nutrient concentrations in plant tissues; (**4**) Chromatography analysis of phytohormone concentrations in plant tissues. Created in BioRender version 2026.

The experimental workflow followed in this study is depicted in Scheme 2.

### 4.1. Plant Material

To evaluate the effect of silver nanoparticles (AgNPs) on growth, biomass production, and nutrient and phytohormone concentrations, tomato (*Solanum lycopersicum* L.) seeds of the cultivars Vengador (Syngenta; Culiacán, Sinaloa, Mexico) and Rio Grande (Caloro; Guadalajara, Jalisco, Mexico) were used. The seeds were germinated in plastic germination trays using agrolite as a substrate. The former cultivar is mostly grown for fresh domestic consumption, whereas the latter is for export and industrialization. Because of their different uses and performance, they were selected to test whether AgNPs have divergent effects among genotypes of the same species.

### 4.2. Preparation of AgNP Solutions

AgNPs (Agrovit^®^) were produced by Bionag (Tijuana, B.C., Mexico). The product is a solution of spherical silver nanoparticles containing 12 mg of metallic silver per mL and 188 mg polyvinylpyrrolidone (PVP, 15–30 kD) per mL of water, with an average concentration of 20% AgNPs (200 mg AgNPs mL^−1^), with a diameter of 70 nm.

### 4.3. Application of AgNPs to Tomato Plants

When the seedlings reached 30 days of age, they were transplanted into 3 L plastic containers containing 100% Steiner nutrient solution, which was replaced every 7 days for 2 weeks. The hydroponic experiment lasted two weeks; the first week was a period of acclimatization of the plants to the hydroponic system, and in the second week, AgNP treatments were applied, exposing the plants to 0, 5, and 10 mg AgNPs L^−1^ for seven days [16,59]. AgNP treatments were applied through the nutrient solution.

### 4.4. Greenhouse Experiment Conditions

The greenhouse conditions were as follows: 75% relative humidity, 25 °C average temperature, and 231 μmol m^−2^ s^−1^ photosynthetically active radiation (PAR) with a 12 h photoperiod. Plants were harvested after 7 d of treatment with AgNPs in hydroponics under greenhouse conditions [16,59].

### 4.5. Growth and Biomass Measurements

After applying the treatments, the following variables were recorded for both cultivars: leaf number, stem length, fresh biomass weight, and dry biomass weight of leaves, stems, and roots. Harvested plants were washed with double-distilled water and dried at room temperature. The leaves, stems, and roots were then separated. Each of the three separated organs was placed in a labeled paper bag, and the samples were dried in a forced-air oven (Riossa, HCF-125; Guadalajara, Jalisco, Mexico) at 72 °C for 72 h to achieve a constant weight. The tissues were then ground in a Wiley mill (Thomas Scientific; Swedesboro, NJ, USA) with a 2 mm sieve. The weights were recorded on an electronic scale (Ohaus, Adventure Pro AV213C; Pine Brook, NJ, USA).

### 4.6. Analysis of Nutrient Concentrations

To measure concentrations of ionic silver and the essential nutrients N, P, K, Ca, Mg, S, Fe, Cu, Zn, Mn, and B in leaves, stems, and roots, the protocols described by Alcántar and Sandoval [60] were followed. The micro-Kjeldahl method was used to quantify total N concentrations [60]. To determine the concentrations of the other nutrients and silver, an acid digestion was performed with 5 mL of 70.0% nitric acid (HNO_3_) (J. T. Baker^®^; Phillipsburg, NJ, USA); the samples were left in predigestion overnight, and 2 mL of 30% H_2_O_2_ was added. To determine the concentrations of these elements, an Inductively Coupled Plasma Optical Emission Spectroscopy equipment (ICP-OES 725-ES; Agilent; Santa Clara, CA, USA) was used.

### 4.7. Analysis of Phytohormone Concentrations

For phytohormone extraction, the protocols described by Pan et al. [61] and Zhou et al. [62] were used. Leaf and root samples were harvested and ground using liquid nitrogen. For each tissue, a sample of 0.1 g was weighed into a plastic tube, and a volume of 500 μL of extraction solution [isopropyl alcohol (J. T. Baker^®^), HPLC-grade water (J. T. Baker^®^), and reagent-grade concentrated hydrochloric acid (HCl) (J. T. Baker^®^) was added. The tubes containing the samples were placed in an ultrasonic bath for 30 min at 4 °C (Scientific, CS-UB32; Victorville, CA, USA). Subsequently, the organic phase (lower phase) was extracted with a micropipette and deposited in a clean tube; the upper phase was washed by adding 500 μL of HPLC-grade dichloromethane and centrifuged again for 15 min at 3750 rpm at 4 °C until the lower phase of the sample was extracted. The solvent was evaporated with nitrogen gas; once evaporated, 500 μL of HPLC-grade methanol, as well as 200 ng of each of the analyzed phytohormones, was added to the 2 mL tubes containing methanol. Samples were filtered through 0.45 μm membrane syringe filters (Millipore^®^, Millex-HV; 0.45 µm, PVDF, 13 mm; Carrigtwohill, Ireland) and stored at 4 °C until chromatographic analysis by HPLC (Agilent Technologies, Agilent 1200 Series Gradient HPLC System; Waldbroon, Germany), with a UV/VIS DAD detector at a wavelength of 203 and 208 nm, an isocratic phase consisting of HPLC-grade water, acetonitrile, and 0.1% phosphoric acid in a 40:20:40 ratio at a flow rate of 1 mL min-1, using a C18 reversed phase column (150 × 4.6 mm 5 µm) (ThermoFisher^®^, Hypersil Gold model; Waltham, MA, USA). The quantitative standards of phytohormones were kinetin (98% purity), gibberellic acid (90% purity), 3-indoleacetic acid (98% purity), salicylic acid (99.9% purity), and (+)-abscisic acid (98% purity), all provided by Sigma-Aldrich (St. Louis, MI, USA). For each standard, a stock solution in methanol at a concentration of 1 mg mL^−1^ was prepared.

### 4.8. Statistical Analysis

With the data obtained, an analysis of variance was performed, and the means were compared with the Tukey test with a significance level of 0.05 using the statistical procedures of the GLIMMIX package of SAS version 9.4. PCA and heatmaps were elaborated using the R software (R Core Team, 2025; Integrated Development Environment for R. Posit Software, PBC, Boston, MA, USA) within the RStudio environment (RStudio Team, 2025).

## 5. Conclusions

This study highlights the impact of a commercial formulation of silver nanoparticles (AgNPs) on growth, biomass production, and concentrations of nutrients and phytohormones of two tomato cultivars: Vengador and Rio Grande. Even though the treatment did not influence growth and biomass production in either of the cultivars evaluated, it was evident that AgNPs can affect nutrient and phytohormone concentrations, which according to PCA analyses can be an event attributed to complex interactions among the phytohormones analyzed. The effects of nutrient and phytohormone concentrations were different between cultivars and among tissues analyzed. In the aboveground parts Vengador plants (leaves and stems), AgNPs had positive effects on Mg, S, Zn, and Mn concentrations, but decreased P and K, while in Rio Grande, the concentrations of N, K, Mg, S, Cu, and Mn, were increased in response to AgNPs, though P, B and the phytohormone KIN were decreased. In the underground part of Vengador plants (roots), only IAA increased in response to AgNPs, but P, K, Mg, Mn, and B decreased; instead, S, ABA and GA increased in roots of Rio Grande in response to AgNPs, whereas P, K, Ca, Mg, Cu, Zn, Mn and B decreased. Therefore, AgNPs significantly decreased the concentration of more nutrients in roots of Rio Grande as compared to other tissues and Vengador as a whole. Interestingly, only the phytohormone KIN was decreased in response to AgNPs, while ABA, IAA and GA were increased. Overall, more positive than negative effects of AgNPs were observed in the aboveground part of the plant, while the underground part was more negatively affected in both cultivars evaluated. Aso interesting is the fact that a greater number of changes were registered in Rio Grande as compared to Vengador. These differential responses observed among cultivars, further demonstrates that the genetic background is a key determinant of variation in the response to AgNPs. Importantly, different genotypes within a species may exhibit marked differences in their stimulation, sensitivity, or toxicity responses, as observed here. Therefore, further studies are needed to explore the real potential of this technology to foster sustainable agriculture and address the great challenges of food security related to climate change, soil degradation, reduced nutrient availability, and increased pesticide use that jeopardize food production and ecosystem health. AgNPs represent an innovative solution that can boost agricultural productivity while maintaining ecological balance, if properly managed. The evidence retrieved from this work unveils the impact of metal-based NMs for agricultural applications. It reveals the impact of nanostructured materials in modulating nutrient and phytohormone concentrations in a so important food crop such as tomato.

## Figures and Tables

**Table 1:** Macronutrient concentrations in 45-day-old tomato (*Solanum lycopersicum* L.) plants cv. Vengador exposed to different concentrations of silver nanoparticles (AgNPs) for seven days.

AgNPs (mg L^−1^)	N	P	K	Ca	Mg	S
	g kg^−1^ DBW					
Leaves						
0	21.89 ± 0.87 a	1.67 ±0.05 a	8.08 ± 0.30 a	11.56 ± 0.62 a	4.92 ± 0.17 c	4.17± 0.31 b
5	23.17 ± 0.87 a	1.49 ± 0.03 b	7.10 ± 0.24 b	9.27 ± 0.85 a	5.61 ± 0.15 b	10.59 ± 1.01 a
10	21.65 ± 0.87 a	1.12 ± 0.04 c	7.00 ± 0.20 b	11.28 ± 0.68 a	6.32 ± 0.11 a	6.50 ± 0.59 b
**Stems**						
0	13.30 ± 0.45 a	2.40 ± 0.10 a	11.52 ± 0.74 a	3.69 ± 0.42 a	2.42 ± 0.13 a	1.30 ± 0.15 a
5	13.18 ± 0.52 a	1.74 ± 1.74 b	10.00 ± 0.29 ab	3.40 ± 0.35 a	2.06 ± 0.11 a	1.19 ± 0.18 a
10	12.83 ± 0.84 a	1.80 ± 1.80 b	9.04 ± 0.31 b	2.54 ± 0.46 a	2.13 ± 0.15 a	2.12 ± 0.37 a
**Roots**						
0	28.35 ± 2.10 a	4.49 ± 0.52 a	15.86 ± 0.96 a	3.29 ± 0.47 a	3.87 ± 0.61 a	3.60 ± 0.84 a
5	29.52 ± 1.20 a	3.94 ± 0.33 a	13.94 ± 0.47 a	4.33 ± 0.14 a	4.00 ± 0.30 a	3.81 ± 0.36 a
10	34.30 ± 2.62 a	2.20 ± 0.04 b	4.52 ± 0.68 b	4.27 ± 0.17 a	1.15 ± 0.06 b	4.62 ± 0.14 a

Different letters in columns in each tissue analyzed and for each macronutrient measured indicate significant differences among treatments tested (Tukey; *p* ≤ 0.05). Data are mean ± SD. DBW: dry biomass weight. *n* = 5.

**Table 2:** Micronutrient and silver concentrations in 45-day-old tomato (*Solanum lycopersicum* L.) plants cv. Vengador exposed to different concentrations of silver nanoparticles (AgNPs) for seven days.

AgNPs (mg L^−1^)	Fe	Cu	Zn	Mn	B	Ag
	mg kg^−1^ DBW					
**Leaves**						
0	89.64 ± 15.96 a	9.53 ± 0.60 a	36.76 ± 1.73 c	26.00 ± 0.84 a	9.53 ± 0.60 a	ND
5	73.33 ± 12.01 a	9.33 ± 0.32 a	45.84 ± 1.44 b	24.71 ± 0.64 a	9.33 ± 0.32 a	ND
10	102.15 ± 10.72 a	10.59 ± 0.32 a	59.40 ± 2.79 a	24.65 ± 0.35 a	10.59 ± 0.32 a	ND
**Stems**						
0	22.58 ± 6.82 a	2.52 ± 0.31 a	8.89 ± 1.12 a	13.32 ± 1.28 b	15.82 ± 0.76 a	ND
5	15.82 ± 5.20 a	1.99 ± 0.27 a	7.03 ± 1.35 a	17.51 ± 2.81 b	14.50 ± 0.38 a	ND
10	30.38 ± 6.19 a	2.88 ± 0.33 a	7.37 ± 1.00 a	28.83 ± 3.15 a	14.40 ± 0.47 a	ND
**Roots**						
0	145.33 ± 40.61 a	25.37 ± 5.07 a	15.25 ± 3.39 a	301.12 ± 83.22 a	19.84 ± 2.69 a	ND
5	220.88 ± 27.31 a	26.04 ± 3.87 a	19.58 ± 1.98 a	320.66 ± 44.25 a	19.36 ± 0.87 a	57.56 ± 11.08 a
10	155.62 ± 23.86 a	14.43 ± 1.73 a	14.94 ± 0.39 a	25.77 ± 1.04 b	15.20 ± 0.40 a	69.98 ± 12.13 a

Different letters in columns in each tissue and element analyzed indicate significant differences among treatments tested (Tukey; *p* ≤ 0.05). Data are mean ± SD. DBW: dry biomass weight. *n* = 5. ND: Not detected.

**Table 3:** Macronutrient concentrations in 45-day-old tomato (*Solanum lycopersicum* L.) plants cv. Rio Grande exposed to different concentrations of AgNPs for seven days.

AgNPs (mg L^−1^)	N	P	K	Ca	Mg	S
	g kg^−1^ DBW					
**Leaves**						
0	16.08 ± 0.87 a	1.85 ± 0.07 a	7.34 ± 0.32 c	11.04 ± 0.36 a	4.92 ± 0.17 c	4.17 ± 0.31 b
5	18.43 ± 0.52 a	2.05 ± 0.04 a	9.90 ± 0.25 a	9.37 ± 0.96 a	5.61 ± 0.15 b	10.59 ± 1.01 a
10	16.69 ± 0.54 a	1.61 ± 0.06 b	8.70 ± 0.30 b	9.84 ± 0.80 a	6.32 ± 0.11 a	6.46 ± 0.59 b
**Stems**						
0	14.63 ± 0.82 b	2.59 ± 0.18 a	10. 04 ± 0.24 a	4.20 ± 0.18 a	2.58 ± 0.13 a	1.34 ± 0.07 c
5	22.98 ± 1.96 a	1.79 ± 0.06 b	5.10 ± 0.31 b	3.25 ± 0.36 b	1.10 ± 0.05 b	12.03 ± 0.38 a
10	27.18 ± 2.03 a	2.43 ± 0.03 a	9.75 ± 0.11 a	3.39 ± 0.06 ab	1.37 ± 0.03 b	4.51 ± 0.04 b
**Roots**						
0	33.32 ± 0.86 a	3.87 ± 0.25 a	17.13 ± 0.45 a	5.04 ± 0.18 a	3.53 ± 0.20 a	3.83 ± 0.24 c
5	37.82 ± 3.14 a	1.54 ± 0.10 b	0.63 ± 0. 04 b	2.03 ± 0.37 b	0.60 ± 0.11 b	10.12 ± 0.56 a
10	31.56 ± 2.01 a	1.73 ± 0.07 b	0.73 ± 0.07 b	2.62 ± 0.27 b	0.50 ± 0.03 b	5.81 ± 0.42 b

Different letters in columns in each tissue and each element analyzed indicate significant differences among treatments tested (Tukey; *p* ≤ 0.05). Data are mean ± SD. DBW: dry biomass weight. *n* = 5.

**Table 4:** Micronutrient and silver concentrations in 45-day-old tomato (*Solanum lycopersicum* L.) plants cv. Rio Grande exposed to different concentrations of silver nanoparticles (AgNPs) for seven days.

AgNPs	Fe	Cu	Zn	Mn	B	Ag
	mg kg^−1^ DBW					
**Leaves**						
0	73.84 ± 5.76 ab	2.91 ± 0.09 b	11.42 ± 11.42 ab	51.00 ± 2.37 b	31.31 ± 1.11 ab	ND
5	58.42 ± 5.00 b	4.02 ± 0.15 a	13.07 ± 0.78 a	59.36 ± 1.55 a	31.14 ± 0.60 b	ND
10	77.58 ± 3.22 a	4.36 ± 0.19 a	9.76 ± 0.40 b	56.39 ± 0.55 ab	34.57 ± 0.94 a	ND
**Stems**						
0	34.35 ± 7.02 a	3.73 ± 0.21 ab	9.31 ± 0.65 a	16.77 ± 1.55 c	16.55 ± 0.78 a	ND
5	46.12 ± 5.24 a	3.04± 0.27 b	10.26 ± 0.58 a	21.51 ± 0.60 b	11.03 ± 0.51 c	ND
10	33.30 ± 3.13 a	3.80 ± 0.10 a	10.43 ± 0.33 a	26.45 ± 0.39 a	14.41 ± 0.22 b	ND
**Roots**						
0	234.03 ± 35.14 a	22.32 ± 3.59 a	16.64 ± 0.99 a	273.7 ± 27.59 a	20.56 ± 1.38 a	ND
5	142.20 ± 10.39 a	4.13 ± 0.39 b	5.86 ± 0.83 b	17.05 ± 1.58 b	6.22 ± 0.88 b	67.69 ± 7.96 a
10	262.84 ± 53.56 a	5.37 ± 0.37 b	7.30 ± 0.64 b	15.54 ± 0.71 b	8.02 ± 1.21 b	106.19 ± 7.96 a

Different letters in columns in each tissue and each element analyzed indicate significant differences among treatments tested (Tukey; *p* ≤ 0.05). Data are mean ± SD. DBW: dry biomass weight. *n* = 5. ND: Not detected.

## Data Availability

The original contributions presented in this study are included in the article/Supplementary Materials. Further inquiries can be directed to the corresponding author.

## Supplementary Materials

The following supporting information can be downloaded at: https://www.mdpi.com/article/10.3390/plants15030405/s1, S1: HPLC Chromatograms of phytohormones determined in tomato plants exposed to different concentrations of silver nanoparticles (AgNPs).
