# Supplementary material for: Influence of Silver Nanoparticles (AgNPs) on Vegetative Growth and Concentrations of Nutrients and Phytohormones in Tomato
Source: Plants (Basel). 2026 Jan 28;15(3):405. doi: 10.3390/plants15030405 (PMC12899181; doi:10.3390/plants15030405)
Supplement: Supplementary file 1 [file plants-15-00405-s001.zip › S1. HPLC Analysis (plants-4015186)/cv. Rio Grande/Leaves/10 ppm/RG-10-L-R1.pdf]

===

Acq. Operator : TMG   Seq. Line : 40
Acq. Instrument : Instrument 1   Location : Vial 40
Injection Date : 10/4/2012 6:27:55 AM   Inj : 1
Inj Volume : 200.0 µl
Different Inj Volume from Sequence !   Actual Inj Volume : 50.0 µl
Acq. Method : C:\CHEM32\1\DATA\FITOHORMTMG\FITOHOR GABY Y ALE 30-11-2020 2012-10-03 09-08-53\FITOHORMONAS DR SOTO.M
Last changed : 8/14/2013 11:13:25 AM by TMG
Analysis Method : C:\CHEM32\1\METHODS\LAVADO COLUMNA ACET.M
Last changed : 10/21/2012 12:24:49 PM by TMG
(modified after loading)

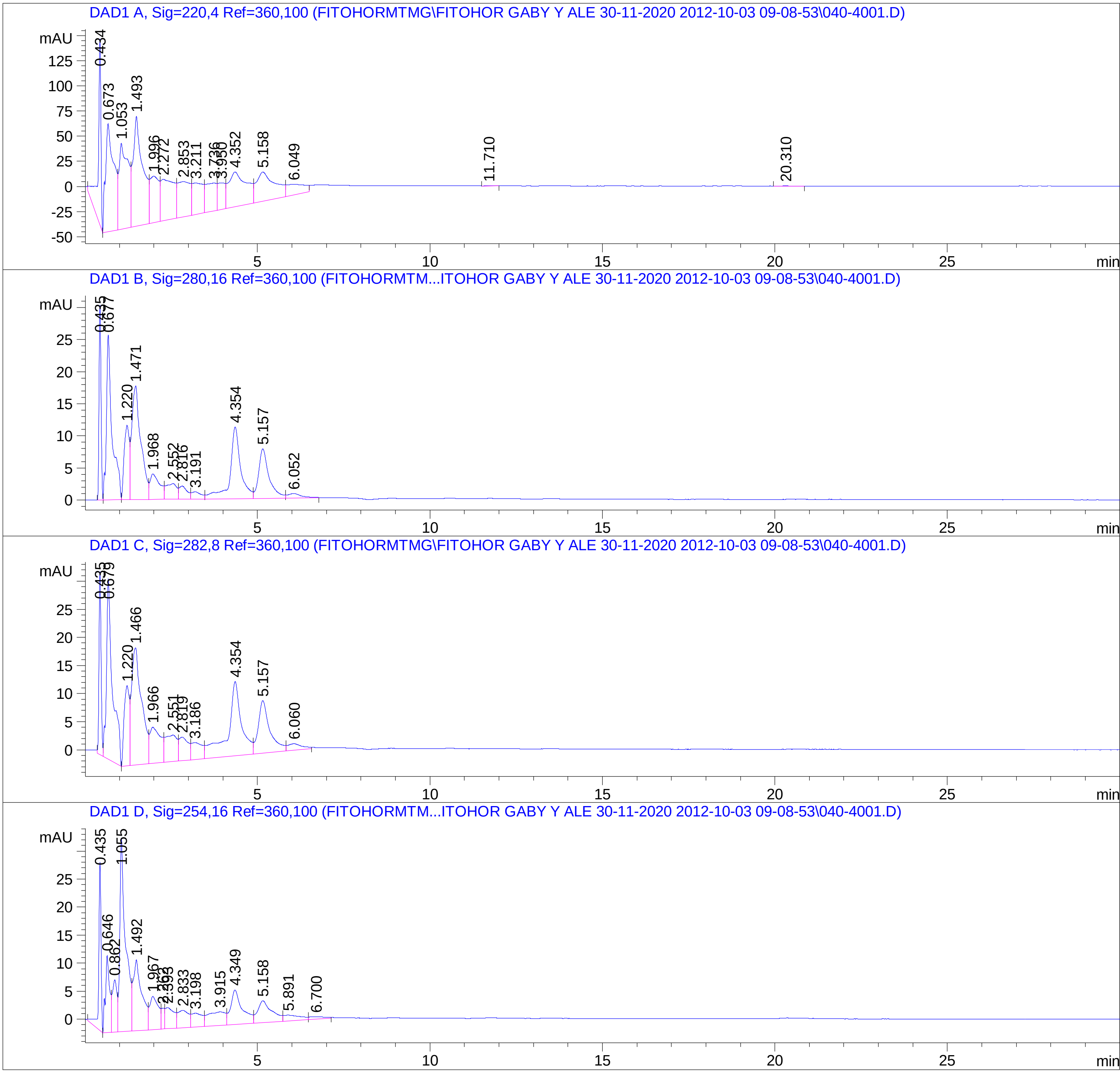

===

Area Percent Report

===

Sorted By : Signal
Multiplier: : 1.0000
Dilution: : 1.0000
Use Multiplier & Dilution Factor with ISTDs

Signal 1: DAD1 A, Sig=220,4 Ref=360,100

Peak #	RetTime [min]	Type	Width [min]	Area [mAU*s]	Height [mAU]	Area %
1	0.434	BV	0.0892	1098.55444	185.32292	7.9247
2	0.673	VV	0.2244	1806.65930	107.31684	13.0328
3	1.053	VV	0.2340	1569.82532	85.58635	11.3243
4	1.493	VV	0.2546	2163.69800	109.46771	15.6084
5	1.996	VV	0.2612	846.46106	46.32051	6.1062
6	2.272	VV	0.3352	1107.47144	41.51182	7.9890
7	2.853	VV	0.3317	878.77637	35.14817	6.3393
8	3.211	VV	0.2993	681.93317	31.15752	4.9193
9	3.736	VV	0.2862	600.42554	27.55269	4.3313
10	3.950	VV	0.2310	410.52664	26.17356	2.9614
11	4.352	VV	0.4665	1228.65027	34.57867	8.8632
12	5.158	VV	0.4901	1067.91467	28.96473	7.7037
13	6.049	VV	0.4640	390.15878	10.53673	2.8145
14	11.710	BB	0.1694	3.31287	2.65783e-1	0.0239
15	20.310	VB	0.4118	8.04388	2.63746e-1	0.0580

Totals : 1.38624e4 770.16776

Signal 2: DAD1 B, Sig=280,16 Ref=360,100

Peak #	RetTime [min]	Type	Width [min]	Area [mAU*s]	Height [mAU]	Area %
1	0.435	BV	0.0636	123.77355	30.35481	8.4026
2	0.677	VV	0.1587	292.56174	25.71295	19.8611
3	1.220	VV	0.1679	121.11599	11.59041	8.2222
4	1.471	VV	0.2589	322.67514	17.68530	21.9054
5	1.968	VV	0.2859	78.64550	3.99113	5.3390
6	2.552	VV	0.2835	52.26342	2.44379	3.5480
7	2.816	VV	0.2369	33.77072	2.04201	2.2926
8	3.191	VV	0.2697	21.75120	1.12428	1.4766
9	4.354	VV	0.3089	244.39339	11.18618	16.5911
10	5.157	VV	0.3029	162.18059	7.72751	11.0099
11	6.052	VB	0.3964	19.91098	7.25083e-1	1.3517

Totals : 1473.04221 114.58346

Signal 3: DAD1 C, Sig=282,8 Ref=360,100

Peak #	RetTime [min]	Type	Width [min]	Area [mAU*s]	Height [mAU]	Area %
1	0.435	BV	0.0657	138.75798	32.56410	6.5251
2	0.679	VV	0.1668	386.44522	32.02530	18.1727
3	1.220	VV	0.1680	149.13962	14.26707	7.0133
4	1.466	VV	0.2839	425.07172	20.84546	19.9891
5	1.966	VV	0.2803	138.60956	6.45950	6.5181
6	2.551	VV	0.3112	113.23888	4.73240	5.3251
7	2.819	VV	0.2630	79.16005	4.21690	3.7225
8	3.186	VV	0.3035	67.27414	3.02406	3.1636
9	4.354	VV	0.3850	374.63217	13.20574	17.6171
10	5.157	VV	0.3333	220.78722	9.35575	10.3826
11	6.060	VB	0.4055	33.40418	1.17558	1.5708
Totals :				2126.52073	141.87187	

Signal 4: DAD1 D, Sig=254,16 Ref=360,100

Peak #	RetTime [min]	Type	Width [min]	Area [mAU*s]	Height [mAU]	Area %
1	0.435	BV	0.0733	142.96098	30.16282	8.4424
2	0.646	VV	0.1159	115.58834	13.76889	6.8259
3	0.862	VV	0.1357	87.82294	9.34120	5.1863
4	1.055	VV	0.1610	411.77957	34.55461	24.3171
5	1.492	VV	0.2316	224.75752	12.63001	13.2728
6	1.967	VV	0.2403	104.87720	5.93099	6.1934
7	2.262	VV	0.0929	24.51733	3.71881	1.4478
8	2.393	VV	0.2449	67.46870	3.76768	3.9843
9	2.833	VV	0.3097	67.76496	3.09147	4.0018
10	3.198	VV	0.2992	53.46586	2.44436	3.1574
11	3.915	VV	0.4919	86.29234	2.39736	5.0959
12	4.349	VV	0.3238	144.87010	6.12907	8.5551
13	5.158	VV	0.3880	111.91683	3.90899	6.6091
14	5.891	VV	0.4246	35.39748	1.04664	2.0904
15	6.700	VB	0.3695	13.89349	4.63794e-1	0.8205
Totals :				1693.37365	133.35670	

==

*** End of Report ***
