# Supplementary material for: Influence of Silver Nanoparticles (AgNPs) on Vegetative Growth and Concentrations of Nutrients and Phytohormones in Tomato
Source: Plants (Basel). 2026 Jan 28;15(3):405. doi: 10.3390/plants15030405 (PMC12899181; doi:10.3390/plants15030405)
Supplement: Supplementary file 1 [file plants-15-00405-s001.zip › S1. HPLC Analysis (plants-4015186)/cv. Rio Grande/Leaves/10 ppm/RG-10-L-R2.pdf]

Data File C:\CHEM32\...ORMTMG\FITOHOR GABY Y ALE 30-11-2020 2012-10-03 09-08-53\041-4101.D
Sample Name: 10 PPM RIO GRANDE HOJA R2

===

Acq. Operator : TMG Seq. Line : 41
Acq. Instrument : Instrument 1 Location : Vial 41
Injection Date : 10/4/2012 7:00:03 AM Inj : 1
Inj Volume : 200.0 µl
Different Inj Volume from Sequence ! Actual Inj Volume : 50.0 µl
Acq. Method : C:\CHEM32\1\DATA\FITOHORMTMG\FITOHOR GABY Y ALE 30-11-2020 2012-10-03 09-08-53\FITOHORMONAS DR SOTO.M
Last changed : 8/14/2013 11:13:25 AM by TMG
Analysis Method : C:\CHEM32\1\METHODS\LAVADO COLUMNA ACET.M
Last changed : 10/21/2012 12:24:49 PM by TMG
(modified after loading)

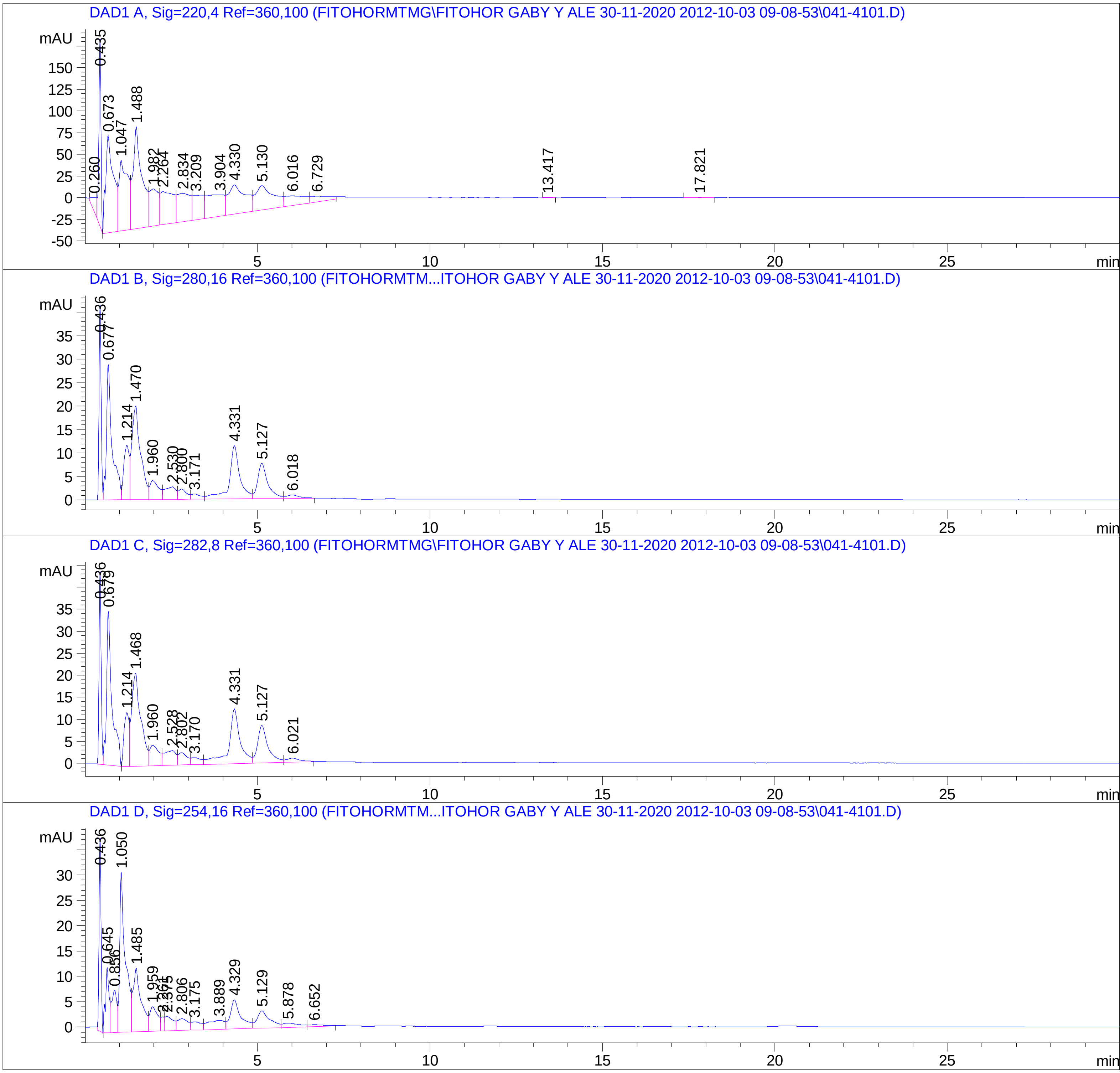

===

Area Percent Report

===

Sorted By : Signal
Multiplier: : 1.0000
Dilution: : 1.0000
Use Multiplier & Dilution Factor with ISTDs

Signal 1: DAD1 A, Sig=220,4 Ref=360,100

Peak #	RetTime [min]	Type	Width [min]	Area [mAU*s]	Height [mAU]	Area %
1	0.260	BV	0.1447	156.31114	15.62907	1.1585
2	0.435	VV	0.0708	942.96326	216.45215	6.9885
3	0.673	VV	0.2076	1755.90930	111.58024	13.0134
4	1.047	VV	0.2355	1466.62610	80.91369	10.8695
5	1.488	VV	0.2365	2135.94482	117.26332	15.8300
6	1.982	VV	0.2630	786.58789	42.68097	5.8296
7	2.264	VV	0.3312	989.35443	38.06350	7.3323
8	2.834	VV	0.3854	886.76300	32.59623	6.5720
9	3.209	VV	0.2719	582.48712	28.32571	4.3169
10	3.904	VV	0.4667	909.90338	24.64476	6.7435
11	4.330	VV	0.4602	1174.72485	33.57236	8.7062
12	5.130	VV	0.4839	1021.58459	28.11447	7.5712
13	6.016	VV	0.5325	441.48105	10.99359	3.2719
14	6.729	VB	0.4482	234.18539	6.26313	1.7356
15	13.417	BB	0.1743	3.27680	2.68644e-1	0.0243
16	17.821	VB	0.2284	4.93782	2.87473e-1	0.0366

Totals : 1.34930e4 787.64930

Signal 2: DAD1 B, Sig=280,16 Ref=360,100

Peak #	RetTime [min]	Type	Width [min]	Area [mAU*s]	Height [mAU]	Area %
1	0.436	BV	0.0669	174.13536	41.52046	10.9129
2	0.677	VV	0.1595	330.19965	28.86054	20.6934
3	1.214	VV	0.1721	127.51778	11.61773	7.9914
4	1.470	VV	0.2502	349.01462	19.94256	21.8725
5	1.960	VV	0.2630	73.11172	4.04304	4.5819
6	2.530	VV	0.3016	60.96664	2.65784	3.8207
7	2.800	VV	0.2423	36.29382	2.17897	2.2745
8	3.171	VV	0.2741	21.86884	1.10877	1.3705
9	4.331	VV	0.3074	245.67830	11.30998	15.3965
10	5.127	VV	0.3012	155.88708	7.54012	9.7693
11	6.018	VB	0.3691	21.00435	7.63094e-1	1.3163

Totals : 1595.67814 131.54310

Signal 3: DAD1 C, Sig=282,8 Ref=360,100

Peak #	RetTime [min]	Type	Width [min]	Area [mAU*s]	Height [mAU]	Area %
1	0.436	BV	0.0675	185.92404	43.82600	10.1578
2	0.679	VV	0.1519	383.73618	34.99721	20.9651
3	1.214	VV	0.1638	125.17197	12.18610	6.8387
4	1.468	VV	0.2595	385.90320	21.08644	21.0835
5	1.960	VV	0.2723	87.21391	4.66105	4.7649
6	2.528	VV	0.3117	79.55178	3.31875	4.3462
7	2.802	VV	0.2574	50.26081	2.82790	2.7460
8	3.170	VV	0.2750	31.89495	1.62480	1.7426
9	4.331	VV	0.3290	292.25473	12.39870	15.9671
10	5.127	VV	0.3084	181.76898	8.53692	9.9308
11	6.021	VB	0.4005	26.67636	9.30372e-1	1.4574

Totals : 1830.35691 146.39423

Signal 4: DAD1 D, Sig=254,16 Ref=360,100

Peak #	RetTime [min]	Type	Width [min]	Area [mAU*s]	Height [mAU]	Area %
1	0.436	BV	0.0658	156.61539	38.22682	10.7469
2	0.645	VV	0.1083	98.65290	12.74524	6.7695
3	0.856	VV	0.1392	81.06832	8.35470	5.5629
4	1.050	VV	0.1587	369.48175	31.54058	25.3537
5	1.485	VV	0.2144	204.59300	12.53413	14.0391
6	1.959	VV	0.2325	81.87383	4.86153	5.6182
7	2.261	VV	0.0880	17.20903	2.78879	1.1809
8	2.375	VV	0.2396	49.27896	2.85244	3.3815
9	2.806	VV	0.3086	49.63150	2.32950	3.4057
10	3.175	VV	0.2785	33.71519	1.66305	2.3135
11	3.889	VV	0.4621	61.80767	1.81114	4.2412
12	4.329	VV	0.2991	123.43332	5.73471	8.4700
13	5.129	VV	0.3616	89.72455	3.42490	6.1569
14	5.878	VV	0.4184	27.85142	8.49747e-1	1.9112
15	6.652	VB	0.4141	12.37177	3.77790e-1	0.8489

Totals : 1457.30860 130.09506

===

*** End of Report ***
