# Supplementary material for: Influence of Silver Nanoparticles (AgNPs) on Vegetative Growth and Concentrations of Nutrients and Phytohormones in Tomato
Source: Plants (Basel). 2026 Jan 28;15(3):405. doi: 10.3390/plants15030405 (PMC12899181; doi:10.3390/plants15030405)
Supplement: Supplementary file 1 [file plants-15-00405-s001.zip › S1. HPLC Analysis (plants-4015186)/cv. Rio Grande/Leaves/10 ppm/RG-10-L-R3.pdf]

```
=====================================================================
Acq. Operator   : TMG                            Seq. Line :  42
Acq. Instrument : Instrument 1                    Location : Vial 42
Injection Date  : 10/4/2012 7:32:12 AM                 Inj :   1
                                                Inj Volume : 200.0 µl
Different Inj Volume from Sequence !     Actual Inj Volume : 50.0 µl
Acq. Method     : C:\CHEM32\1\DATA\FITOHORMTMG\FITOHOR GABY Y ALE 30-11-2020 2012-10-03 09-08-
                  53\FITOHORMONAS DR SOTO.M
Last changed    : 8/14/2013 11:13:25 AM by TMG
Analysis Method : C:\CHEM32\1\METHODS\LAVADO COLUMNA ACET.M
Last changed    : 10/21/2012 12:24:49 PM by TMG
                  (modified after loading)
```

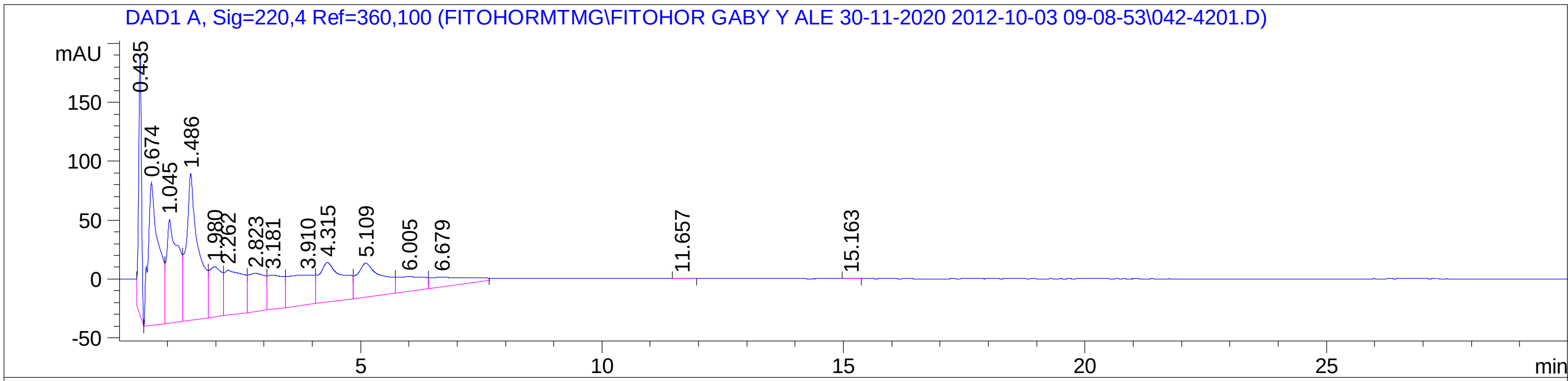

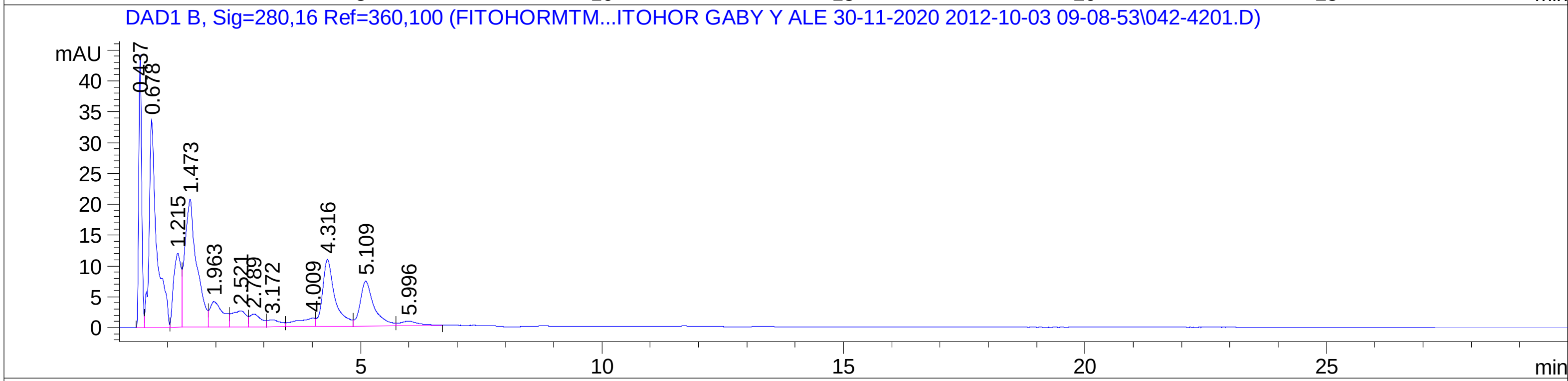

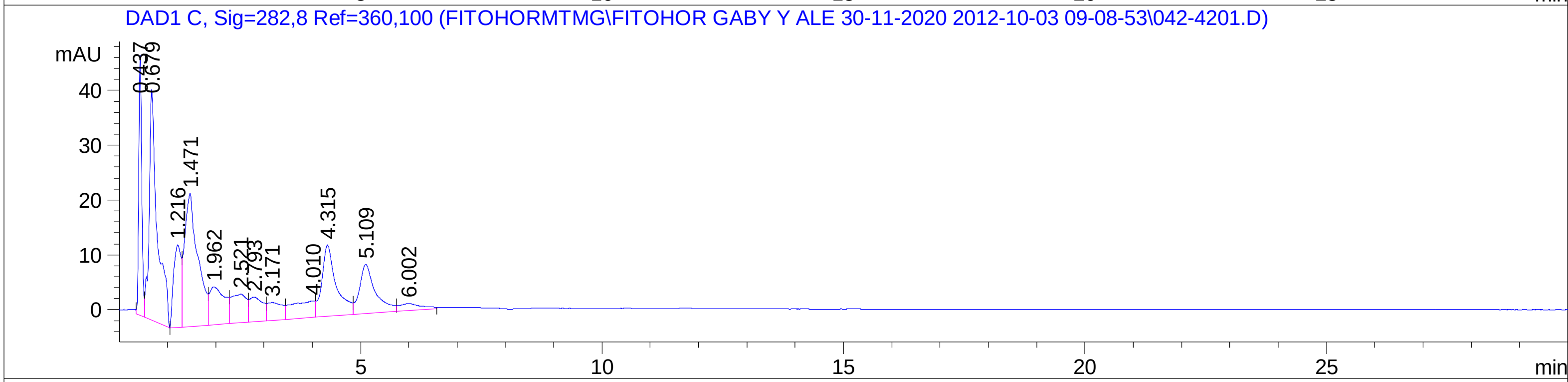

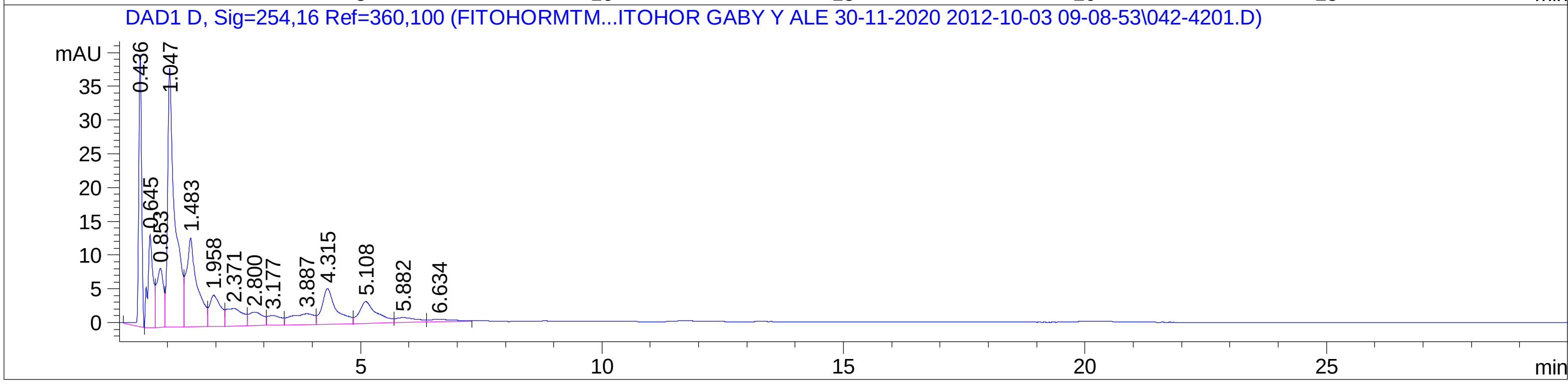

===

Area Percent Report

===

Sorted By : Signal
Multiplier: : 1.0000
Dilution: : 1.0000
Use Multiplier & Dilution Factor with ISTDs

Signal 1: DAD1 A, Sig=220,4 Ref=360,100

Peak #	RetTime [min]	Type	Width [min]	Area [mAU*s]	Height [mAU]	Area %
1	0.435	BV	0.0640	905.00293	219.75465	6.6023
2	0.674	VV	0.2049	1831.86072	120.80356	13.3641
3	1.045	VV	0.2199	1501.30249	87.61148	10.9526
4	1.486	VV	0.2278	2170.80469	124.29805	15.8368
5	1.980	VV	0.2551	768.59680	42.47745	5.6072
6	2.262	VV	0.3398	1019.13257	38.13857	7.4349
7	2.823	VV	0.3379	769.33478	32.29647	5.6126
8	3.181	VV	0.3061	647.27167	28.80666	4.7221
9	3.910	VV	0.4777	952.32153	24.92525	6.9475
10	4.315	VV	0.4600	1172.63562	33.53452	8.5548
11	5.109	VV	0.4835	1040.12390	28.65149	7.5881
12	6.005	VB	0.5094	475.48364	12.34320	3.4688
13	6.679	BB	0.6816	444.68872	8.23993	3.2442
14	11.657	BB	0.2029	4.97227	3.56088e-1	0.0363
15	15.163	BV	0.1886	3.79417	2.56408e-1	0.0277

Totals : 1.37073e4 802.49380

Signal 2: DAD1 B, Sig=280,16 Ref=360,100

Peak #	RetTime [min]	Type	Width [min]	Area [mAU*s]	Height [mAU]	Area %
1	0.437	BV	0.0668	185.07730	44.28492	11.4174
2	0.678	VV	0.1560	368.09836	33.55010	22.7079
3	1.215	VV	0.1699	127.21843	11.98386	7.8481
4	1.473	VV	0.2436	352.53201	20.81882	21.7476
5	1.963	VV	0.2754	79.74510	4.12732	4.9195
6	2.521	VV	0.2751	53.69286	2.62035	3.3123
7	2.789	VV	0.2386	34.41183	2.06268	2.1229
8	3.172	VV	0.2742	21.31232	1.10901	1.3148
9	4.009	VV	0.3472	36.30175	1.32682	2.2394
10	4.316	VV	0.2627	195.47711	10.82556	12.0590
11	5.109	VV	0.2932	146.70363	7.27609	9.0501
12	5.996	VB	0.3767	20.44188	7.43625e-1	1.2611

Totals : 1621.01258 140.72914

Signal 3: DAD1 C, Sig=282,8 Ref=360,100

Peak #	RetTime [min]	Type	Width [min]	Area [mAU*s]	Height [mAU]	Area %
1	0.437	BV	0.0685	205.67592	47.51775	8.6960
2	0.679	VV	0.1605	482.52945	41.84834	20.4014
3	1.216	VV	0.1722	160.20174	15.05006	6.7734
4	1.471	VV	0.2719	465.11380	24.23298	19.6651
5	1.962	VV	0.3057	151.73456	6.92368	6.4154
6	2.521	VV	0.2988	117.25282	5.20547	4.9575
7	2.793	VV	0.2705	87.45937	4.50501	3.6978
8	3.171	VV	0.3075	70.31197	3.26153	2.9728
9	4.010	VV	0.4288	105.21377	3.04708	4.4485
10	4.315	VV	0.2957	271.77380	13.01080	11.4906
11	5.109	VV	0.3260	208.25429	8.99845	8.8050
12	6.002	VB	0.4145	39.65551	1.26919	1.6766

Totals : 2365.17698 174.87034

Signal 4: DAD1 D, Sig=254,16 Ref=360,100

Peak #	RetTime [min]	Type	Width [min]	Area [mAU*s]	Height [mAU]	Area %
1	0.436	BV	0.0669	169.07236	40.35844	11.5767
2	0.645	VV	0.1069	104.97060	13.78167	7.1875
3	0.853	VV	0.1399	84.25243	8.78383	5.7689
4	1.047	VV	0.1463	409.01770	38.39183	28.0063
5	1.483	VV	0.2053	204.93210	13.18436	14.0321
6	1.958	VV	0.2367	75.55767	4.62300	5.1736
7	2.371	VV	0.3101	62.85196	2.61772	4.3036
8	2.800	VV	0.2938	40.25550	1.99144	2.7564
9	3.177	VV	0.2774	28.47862	1.44837	1.9500
10	3.887	VV	0.4460	53.52998	1.57709	3.6653
11	4.315	VV	0.2917	109.70870	5.25250	7.5120
12	5.108	VV	0.3501	81.69373	3.21921	5.5937
13	5.882	VV	0.4113	22.43454	7.32442e-1	1.5361
14	6.634	VB	0.4560	13.69540	3.71590e-1	0.9378

Totals : 1460.45127 136.33349

===

*** End of Report ***
