# Supplementary material for: Influence of Silver Nanoparticles (AgNPs) on Vegetative Growth and Concentrations of Nutrients and Phytohormones in Tomato
Source: Plants (Basel). 2026 Jan 28;15(3):405. doi: 10.3390/plants15030405 (PMC12899181; doi:10.3390/plants15030405)
Supplement: Supplementary file 1 [file plants-15-00405-s001.zip › S1. HPLC Analysis (plants-4015186)/cv. Rio Grande/Leaves/5 ppm/RG-5-L-R1.pdf]

Data File C:\CHEM32\...ORMTMG\FITOHOR GABY Y ALE 30-11-2020 2012-10-03 09-08-53\028-2801.D
Sample Name: 5 PPM RIO GRANDE HOJA R1

===

Acq. Operator : TMG Seq. Line : 28
Acq. Instrument : Instrument 1 Location : Vial 28
Injection Date : 10/4/2012 12:02:31 AM Inj : 1
Inj Volume : 200.0 µl
Different Inj Volume from Sequence ! Actual Inj Volume : 50.0 µl
Acq. Method : C:\CHEM32\1\DATA\FITOHORMTMG\FITOHOR GABY Y ALE 30-11-2020 2012-10-03 09-08-53\FITOHORMONAS DR SOTO.M
Last changed : 8/14/2013 11:13:25 AM by TMG
Analysis Method : C:\CHEM32\1\METHODS\LAVADO COLUMNA ACET.M
Last changed : 10/21/2012 12:24:49 PM by TMG
(modified after loading)

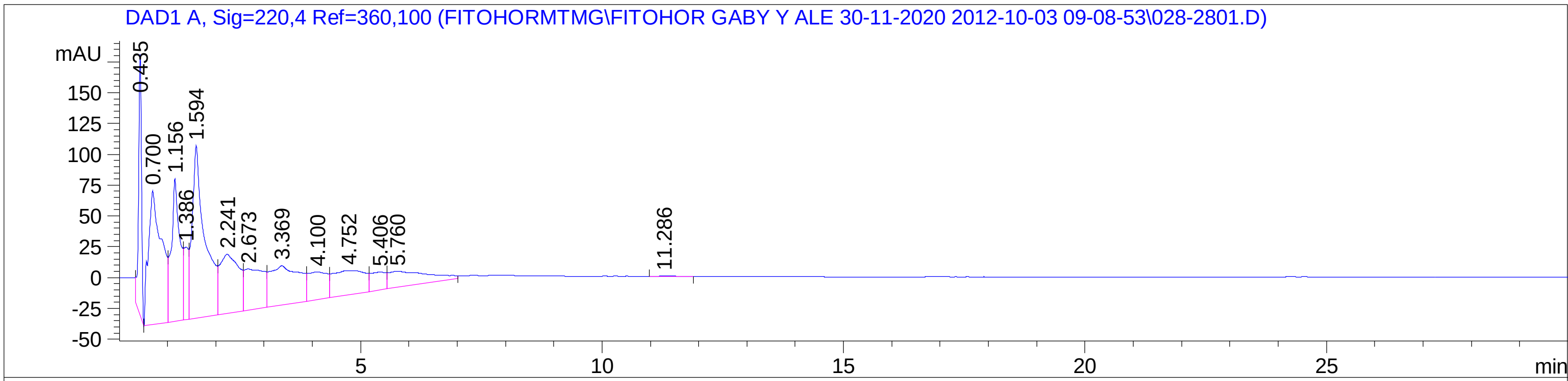

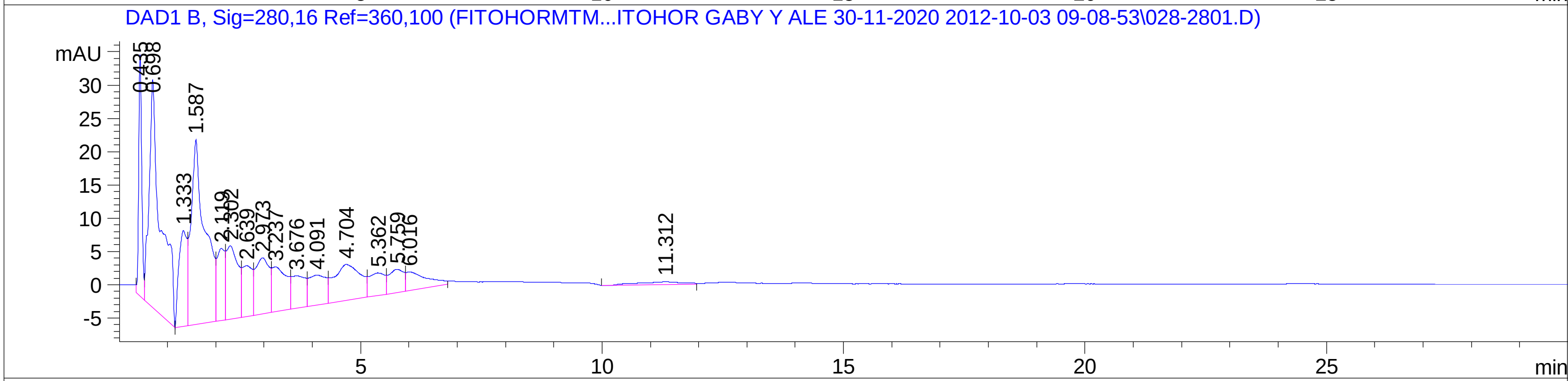

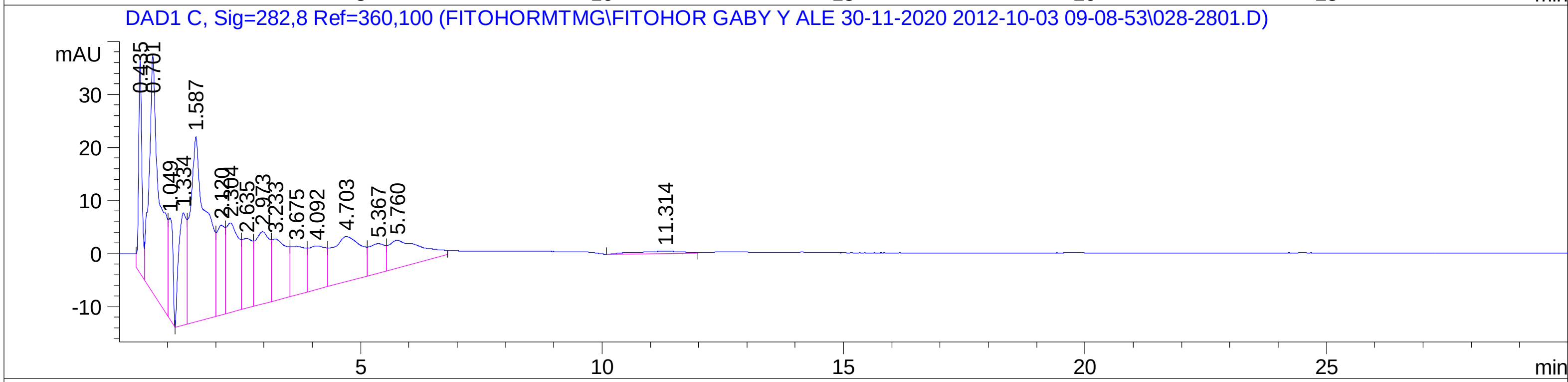

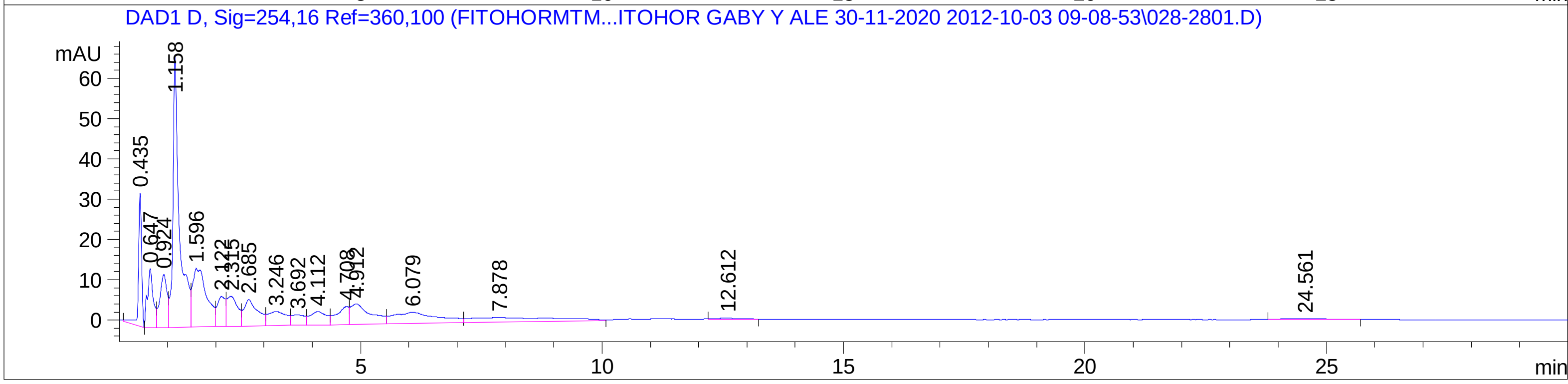

===

Area Percent Report

===

Sorted By : Signal
Multiplier: : 1.0000
Dilution: : 1.0000
Use Multiplier & Dilution Factor with ISTDs

Signal 1: DAD1 A, Sig=220,4 Ref=360,100

Peak #	RetTime [min]	Type	Width [min]	Area [mAU*s]	Height [mAU]	Area %
1	0.435	BV	0.0658	894.59534	209.52379	6.6406
2	0.700	VV	0.2451	2048.15259	108.06505	15.2034
3	1.156	VV	0.1650	1432.55237	115.23535	10.6338
4	1.386	VV	0.0954	378.57541	58.61090	2.8102
5	1.594	VV	0.2438	2582.92017	139.62213	19.1730
6	2.241	VV	0.3619	1318.10815	47.77078	9.7843
7	2.673	VV	0.3577	929.32166	33.29303	6.8983
8	3.369	VV	0.5264	1335.41943	31.76983	9.9128
9	4.100	VV	0.3656	610.84094	22.43936	4.5343
10	4.752	VV	0.5879	911.81445	19.70221	6.7684
11	5.406	VV	0.2935	315.45767	14.51837	2.3416
12	5.760	VB	0.6853	703.66742	13.12882	5.2233
13	11.286	BB	0.2998	10.25124	4.46665e-1	0.0761
Totals :				1.34717e4	814.12629	

Signal 2: DAD1 B, Sig=280,16 Ref=360,100

Peak #	RetTime [min]	Type	Width [min]	Area [mAU*s]	Height [mAU]	Area %
1	0.435	BV	0.0691	158.92635	36.30022	5.5882
2	0.698	VV	0.2137	558.29547	34.33137	19.6309
3	1.333	VV	0.1860	165.24130	14.38850	5.8102
4	1.587	VV	0.2650	562.91168	27.71070	19.7932
5	2.119	VV	0.1579	120.26001	10.80320	4.2286
6	2.302	VV	0.2318	187.85101	10.96964	6.6052
7	2.639	VV	0.2045	109.74704	7.60013	3.8589
8	2.973	VV	0.2775	167.11592	8.42025	5.8762
9	3.237	VV	0.2870	140.49477	6.74626	4.9401
10	3.676	VV	0.2687	97.31435	4.87873	3.4218
11	4.091	VV	0.3491	113.28107	4.50976	3.9832
12	4.704	VV	0.5381	205.95294	5.41071	7.2417
13	5.362	VV	0.3206	74.83506	3.37560	2.6314
14	5.759	VV	0.3077	75.07838	3.48022	2.6399
15	6.016	VB	0.4108	82.06189	2.76040	2.8855

Peak #	RetTime [min]	Type	Width [min]	Area [mAU*s]	Height [mAU]	Area %
16	11.312	BV	0.7418	24.59969	3.91115e-1	0.8650

Totals : 2843.96693 182.07680

Signal 3: DAD1 C, Sig=282,8 Ref=360,100

Peak #	RetTime [min]	Type	Width [min]	Area [mAU*s]	Height [mAU]	Area %
1	0.435	BV	0.0725	188.12370	40.29311	4.2976
2	0.701	VV	0.1983	670.58490	44.87532	15.3193
3	1.049	VV	0.0986	117.90305	18.95610	2.6935
4	1.334	VV	0.1828	236.40891	21.08657	5.4007
5	1.587	VV	0.3012	809.80194	34.83859	18.4996
6	2.120	VV	0.1569	189.05716	16.84591	4.3189
7	2.304	VV	0.2427	304.40341	16.85418	6.9540
8	2.635	VV	0.2054	190.84193	13.14452	4.3597
9	2.973	VV	0.2916	283.60504	13.58184	6.4788
10	3.233	VV	0.2903	244.16324	11.56319	5.5778
11	3.675	VV	0.2787	189.36462	9.10334	4.3260
12	4.092	VV	0.3486	205.09296	8.17703	4.6853
13	4.703	VV	0.5777	351.20206	8.47135	8.0231
14	5.367	VV	0.3314	127.19646	5.50778	2.9058
15	5.760	VB	0.5926	240.47630	5.22861	5.4936
16	11.314	BV	0.7915	29.17277	4.36606e-1	0.6664

Totals : 4377.39847 268.96404

Signal 4: DAD1 D, Sig=254,16 Ref=360,100

Peak #	RetTime [min]	Type	Width [min]	Area [mAU*s]	Height [mAU]	Area %
1	0.435	BV	0.0704	149.26956	33.20433	6.0910
2	0.647	VV	0.1169	123.97749	14.63142	5.0589
3	0.924	VV	0.1573	138.34236	13.08775	5.6451
4	1.158	VV	0.1279	627.43915	67.84641	25.6029
5	1.596	VV	0.2568	297.05914	14.51443	12.1216
6	2.122	VV	0.1664	88.45800	7.45787	3.6096
7	2.315	VV	0.2167	115.42574	7.45226	4.7100
8	2.685	VV	0.2692	131.75204	6.59120	5.3762
9	3.246	VV	0.3811	94.68333	3.50512	3.8636
10	3.692	VV	0.2534	48.51232	2.57987	1.9796
11	4.112	VV	0.3220	77.96996	3.31992	3.1816
12	4.708	VV	0.2441	78.31925	4.43441	3.1958
13	4.912	VV	0.3931	147.92110	5.08947	6.0360

Peak #	RetTime [min]	Type	Width [min]	Area [mAU*s]	Height [mAU]	Area %
14	6.079	VV	0.8116	176.10896	2.77792	7.1862
15	7.878	VV	1.3934	132.43326	1.13154	5.4040
16	12.612	BB	0.3908	7.05901	2.25767e-1	0.2880
17	24.561	BB	0.6856	15.92991	2.76742e-1	0.6500

Totals : 2450.66059 188.12644

*** End of Report ***
