# Supplementary material for: Influence of Silver Nanoparticles (AgNPs) on Vegetative Growth and Concentrations of Nutrients and Phytohormones in Tomato
Source: Plants (Basel). 2026 Jan 28;15(3):405. doi: 10.3390/plants15030405 (PMC12899181; doi:10.3390/plants15030405)
Supplement: Supplementary file 1 [file plants-15-00405-s001.zip › S1. HPLC Analysis (plants-4015186)/cv. Rio Grande/Leaves/5 ppm/RG-5-L-R2.pdf]

Data File C:\CHEM32\...ORMTMG\FITOHOR GABY Y ALE 30-11-2020 2012-10-03 09-08-53\029-2901.D
Sample Name: 5 PPM RIO GRANDE HOJA R2

===

Acq. Operator : TMG Seq. Line : 29
Acq. Instrument : Instrument 1 Location : Vial 29
Injection Date : 10/4/2012 12:34:37 AM Inj : 1
Inj Volume : 200.0 µl
Different Inj Volume from Sequence ! Actual Inj Volume : 50.0 µl
Acq. Method : C:\CHEM32\1\DATA\FITOHORMTMG\FITOHOR GABY Y ALE 30-11-2020 2012-10-03 09-08-53\FITOHORMONAS DR SOTO.M
Last changed : 8/14/2013 11:13:25 AM by TMG
Analysis Method : C:\CHEM32\1\METHODS\LAVADO COLUMNA ACET.M
Last changed : 10/21/2012 12:24:49 PM by TMG
(modified after loading)

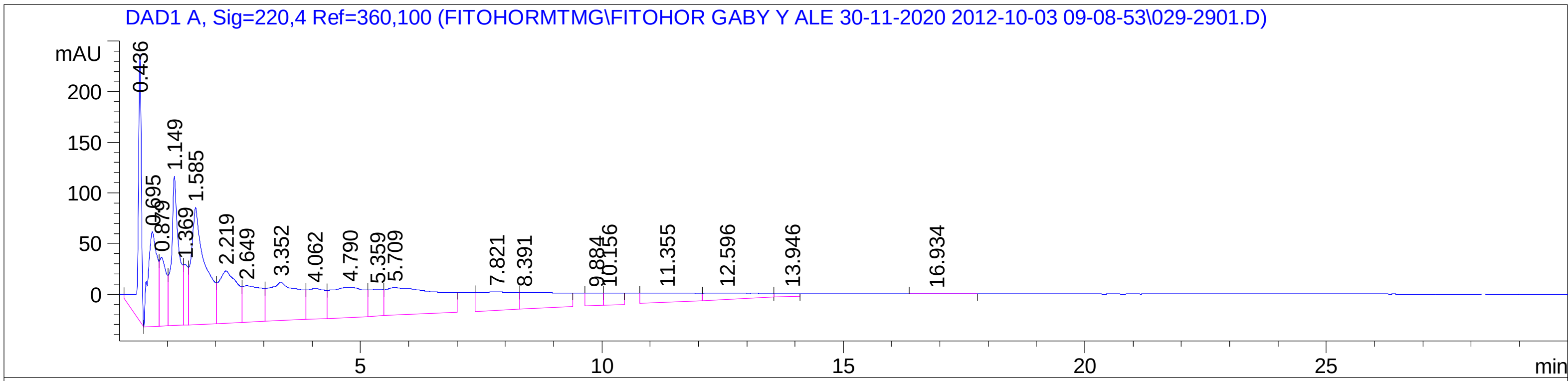

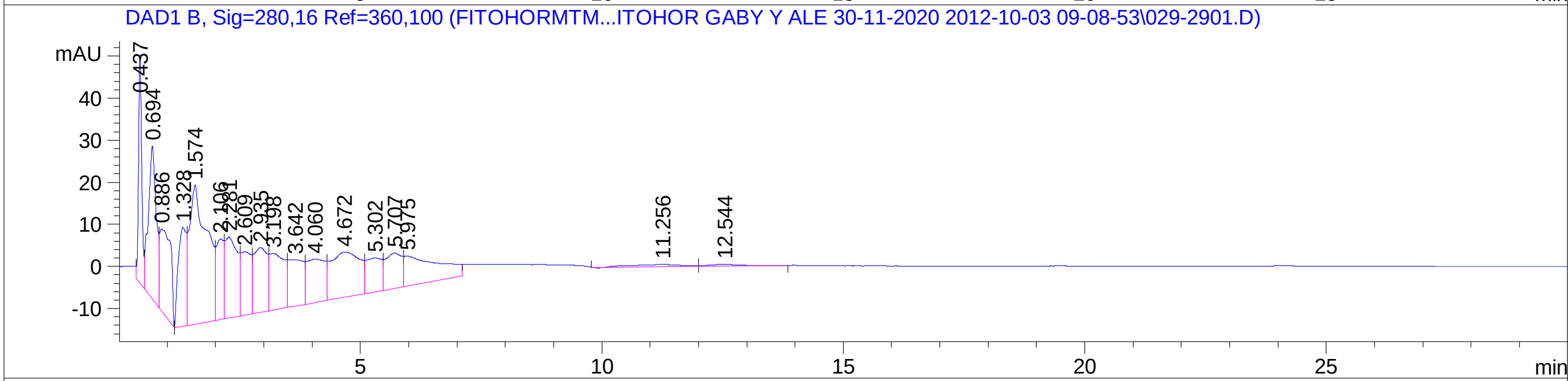

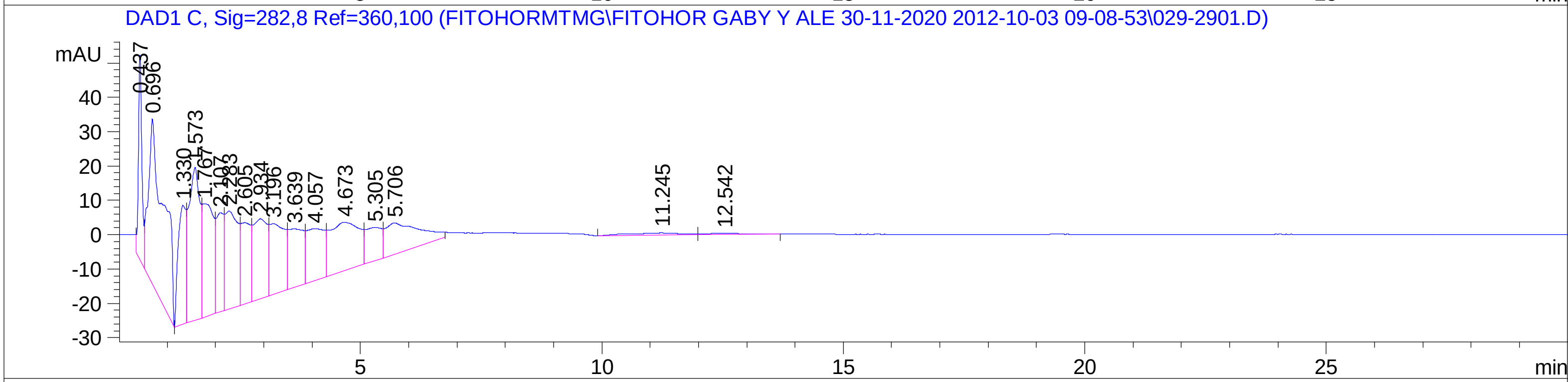

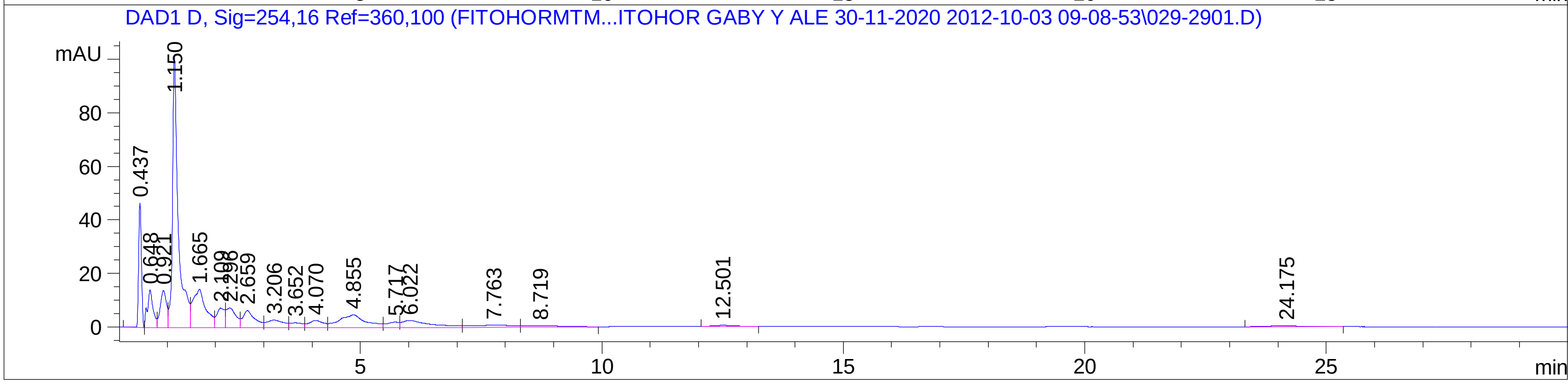

===

Area Percent Report

===

Sorted By : Signal
Multiplier: : 1.0000
Dilution: : 1.0000
Use Multiplier & Dilution Factor with ISTDs

Signal 1: DAD1 A, Sig=220,4 Ref=360,100

Peak #	RetTime [min]	Type	Width [min]	Area [mAU*s]	Height [mAU]	Area %
1	0.436	BV	0.0765	1276.72656	263.61047	6.3167
2	0.695	VV	0.1868	1210.70679	93.69147	5.9900
3	0.879	VV	0.1369	656.70886	67.83731	3.2491
4	1.149	VV	0.1480	1615.62146	147.30685	7.9934
5	1.369	VV	0.0933	375.13217	59.70579	1.8560
6	1.585	VV	0.2664	2355.65747	115.30078	11.6548
7	2.219	VV	0.3584	1387.42529	51.47829	6.8644
8	2.649	VV	0.3445	977.95471	36.04952	4.8385
9	3.352	VV	0.5459	1653.84753	37.84340	8.1825
10	4.062	VV	0.3437	749.52557	29.98048	3.7083
11	4.790	VV	0.6779	1445.06445	29.58101	7.1495
12	5.359	VV	0.2730	519.83728	26.47849	2.5719
13	5.709	VB	0.9560	2123.21509	27.40031	10.5047
14	7.821	BV	0.7118	1000.59583	18.13277	4.9505
15	8.391	VB	0.7296	984.93304	16.37846	4.8730
16	9.884	BV	0.3001	292.80276	12.55651	1.4487
17	10.156	VB	0.3215	296.77267	11.96030	1.4683
18	11.355	BV	0.9040	686.90912	9.31861	3.3985
19	12.596	VV	0.9229	487.48508	6.27097	2.4119
20	13.946	VV	0.4410	100.15118	2.88515	0.4955
21	16.934	BB	0.5108	14.91592	3.63443e-1	0.0738

Totals : 2.02120e4 1064.13039

Signal 2: DAD1 B, Sig=280,16 Ref=360,100

Peak #	RetTime [min]	Type	Width [min]	Area [mAU*s]	Height [mAU]	Area %
1	0.437	BV	0.0699	250.67252	54.30143	4.8248
2	0.694	VV	0.1575	410.74432	36.43271	7.9058
3	0.886	VV	0.2138	326.67120	19.46875	6.2876
4	1.328	VV	0.1862	269.76166	23.44441	5.1922
5	1.574	VV	0.3282	837.75311	33.00220	16.1246
6	2.106	VV	0.1539	209.76060	19.14058	4.0373
7	2.281	VV	0.2383	339.63196	19.20584	6.5370

Peak #	RetTime [min]	Type	Width [min]	Area [mAU*s]	Height [mAU]	Area %
8	2.609	VV	0.2052	218.25232	15.04948	4.2008
9	2.935	VV	0.2777	306.13693	15.41530	5.8923
10	3.198	VV	0.2924	289.07343	13.46973	5.5639
11	3.642	VV	0.2872	236.14545	11.05669	4.5452
12	4.060	VV	0.3668	272.38556	10.35653	5.2427
13	4.672	VV	0.5889	446.76126	10.70471	8.5990
14	5.302	VV	0.3204	179.20485	8.02934	3.4492
15	5.707	VV	0.3309	200.04956	8.36816	3.8504
16	5.975	VB	0.6195	344.54361	7.08343	6.6316
17	11.256	BV	0.8230	36.80828	5.32113e-1	0.7085
18	12.544	VB	0.6004	21.14826	4.22223e-1	0.4070

Totals : 5195.50488 305.48359

Signal 3: DAD1 C, Sig=282,8 Ref=360,100

Peak #	RetTime [min]	Type	Width [min]	Area [mAU*s]	Height [mAU]	Area %
1	0.437	BV	0.0735	294.11636	59.64774	4.1064
2	0.696	VV	0.2825	1065.53589	48.09562	14.8767
3	1.330	VV	0.1819	385.10056	34.57856	5.3767
4	1.573	VV	0.2192	714.50452	44.52263	9.9757
5	1.767	VV	0.2384	527.56586	33.02417	7.3657
6	2.107	VV	0.1561	316.59238	28.84290	4.4202
7	2.283	VV	0.2439	518.12787	28.53186	7.2339
8	2.605	VV	0.2069	342.07184	23.63162	4.7759
9	2.934	VV	0.2842	473.72989	23.20246	6.6141
10	3.196	VV	0.2943	444.30539	20.54541	6.2033
11	3.639	VV	0.2855	362.70258	16.95848	5.0639
12	4.057	VV	0.3604	389.67358	15.13194	5.4405
13	4.673	VV	0.5985	599.91418	14.04142	8.3758
14	5.305	VV	0.3278	222.85532	9.63676	3.1114
15	5.706	VB	0.6118	435.99042	9.11897	6.0872
16	11.245	BV	0.9333	50.14159	6.34934e-1	0.7001
17	12.542	VB	0.5800	19.52318	4.05290e-1	0.2726

Totals : 7162.45139 410.55076

Signal 4: DAD1 D, Sig=254,16 Ref=360,100

Peak #	RetTime [min]	Type	Width [min]	Area [mAU*s]	Height [mAU]	Area %
1	0.437	BV	0.0654	188.94754	46.50340	7.6975
2	0.648	VV	0.1171	118.15435	14.19366	4.8135

Peak #	RetTime [min]	Type	Width [min]	Area [mAU*s]	Height [mAU]	Area %
3	0.921	VV	0.1428	129.74010	13.90752	5.2855
4	1.150	VV	0.1177	850.46497	101.58106	34.6470
5	1.665	VV	0.2400	266.40381	14.38796	10.8530
6	2.109	VV	0.1617	82.30248	7.28952	3.3529
7	2.296	VV	0.2019	105.79520	7.35187	4.3100
8	2.659	VV	0.2378	111.55901	6.38722	4.5448
9	3.206	VV	0.3441	69.14577	2.78123	2.8169
10	3.652	VV	0.2702	32.63205	1.77757	1.3294
11	4.070	VV	0.3015	56.23027	2.65082	2.2908
12	4.855	VV	0.5006	181.67361	4.72500	7.4012
13	5.717	VV	0.2458	34.32127	1.98377	1.3982
14	6.022	VV	0.5486	106.77801	2.59211	4.3500
15	7.763	VV	0.7156	49.20770	8.52217e-1	2.0047
16	8.719	VV	0.7737	40.38837	6.23516e-1	1.6454
17	12.501	BB	0.4150	11.73345	3.73035e-1	0.4780
18	24.175	BB	0.7056	19.17785	3.24452e-1	0.7813

Totals :				2454.65579	230.28593	

===

*** End of Report ***
