# Supplementary material for: Influence of Silver Nanoparticles (AgNPs) on Vegetative Growth and Concentrations of Nutrients and Phytohormones in Tomato
Source: Plants (Basel). 2026 Jan 28;15(3):405. doi: 10.3390/plants15030405 (PMC12899181; doi:10.3390/plants15030405)
Supplement: Supplementary file 1 [file plants-15-00405-s001.zip › S1. HPLC Analysis (plants-4015186)/cv. Rio Grande/Leaves/5 ppm/RG-5-L-R3.pdf]

Data File C:\CHEM32\...ORMTMG\FITOHOR GABY Y ALE 30-11-2020 2012-10-03 09-08-53\030-3001.D
Sample Name: 5 PPM RIO GRANDE HOJA R3

===

```
Acq. Operator   : TMG                              Seq. Line :  30
Acq. Instrument : Instrument 1                      Location : Vial 30
Injection Date  : 10/4/2012 1:06:44 AM                   Inj :   1
                                                  Inj Volume : 200.0 µl
Different Inj Volume from Sequence !       Actual Inj Volume : 50.0 µl
Acq. Method     : C:\CHEM32\1\DATA\FITOHORMTMG\FITOHOR GABY Y ALE 30-11-2020 2012-10-03 09-08-
                  53\FITOHORMONAS DR SOTO.M
Last changed    : 8/14/2013 11:13:25 AM by TMG
Analysis Method : C:\CHEM32\1\METHODS\LAVADO COLUMNA ACET.M
Last changed    : 10/21/2012 12:24:49 PM by TMG
                  (modified after loading)
```

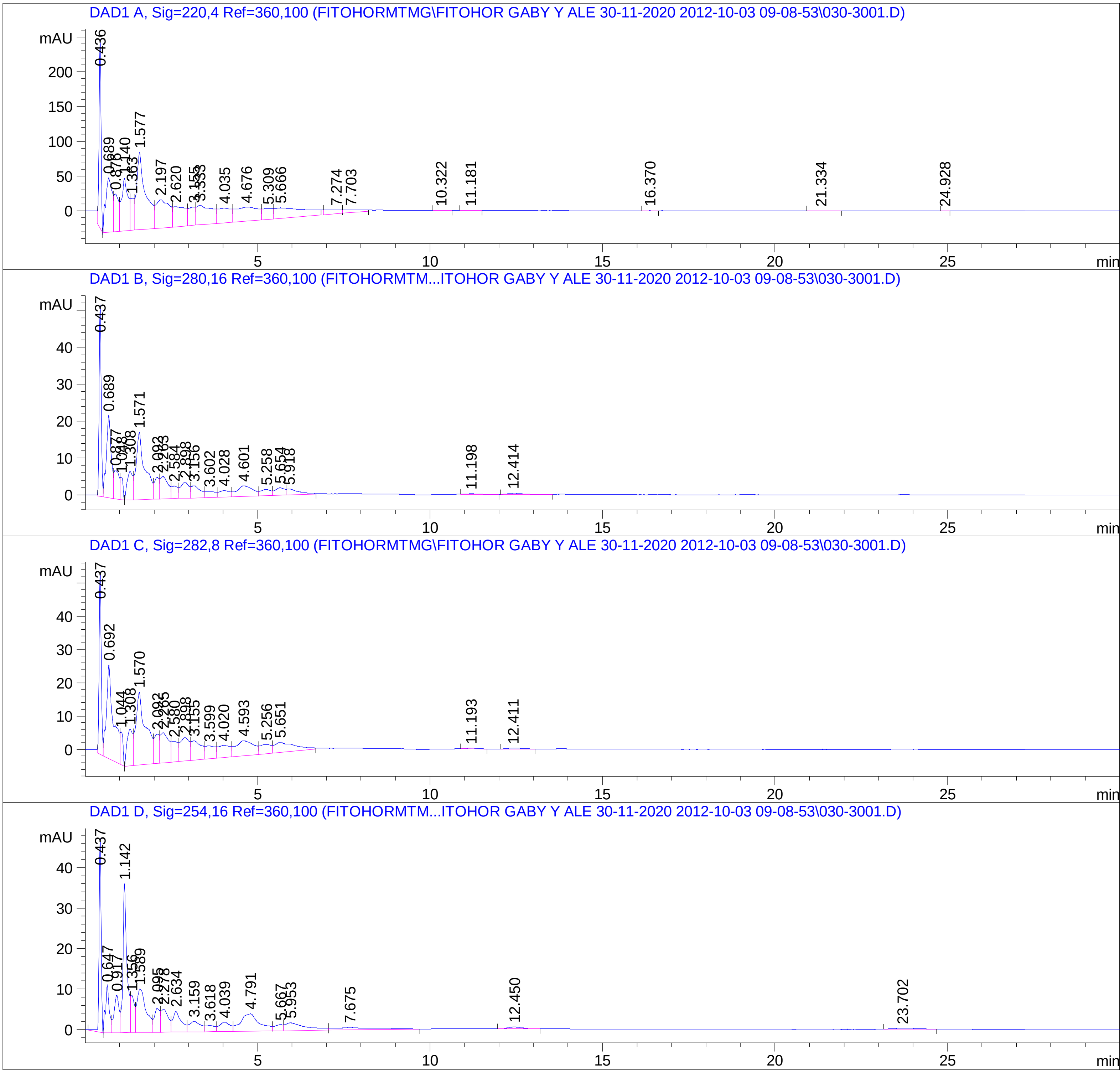

===

Area Percent Report

===

Sorted By : Signal
Multiplier: : 1.0000
Dilution: : 1.0000
Use Multiplier & Dilution Factor with ISTDs

Signal 1: DAD1 A, Sig=220,4 Ref=360,100

Peak #	RetTime [min]	Type	Width [min]	Area [mAU*s]	Height [mAU]	Area %
1	0.436	BV	0.0640	1109.49475	269.64685	9.1289
2	0.689	VV	0.1897	1025.12488	77.81344	8.4347
3	0.876	VV	0.1329	506.99619	54.32589	4.1715
4	1.140	VV	0.1733	994.06860	75.67323	8.1792
5	1.363	VV	0.0992	313.88034	46.21585	2.5826
6	1.577	VV	0.2400	2053.31445	110.91513	16.8946
7	2.197	VV	0.3674	1114.85901	40.71943	9.1730
8	2.620	VV	0.3206	715.99609	28.94916	5.8912
9	3.155	VV	0.1964	362.17078	26.34558	2.9799
10	3.333	VV	0.3942	867.34680	28.25079	7.1365
11	4.035	VV	0.3591	560.88342	21.03685	4.6149
12	4.676	VV	0.5983	922.07397	19.69269	7.5868
13	5.309	VV	0.2871	321.90588	15.70553	2.6486
14	5.666	VB	0.7693	930.38531	14.96625	7.6552
15	7.274	BV	0.4126	186.25372	5.42474	1.5325
16	7.703	VB	0.4936	145.43065	3.91287	1.1966
17	10.322	BB	0.1898	3.88935	2.73615e-1	0.0320
18	11.181	BB	0.2957	10.96355	4.64015e-1	0.0902
19	16.370	BB	0.1743	3.12538	2.30397e-1	0.0257
20	21.334	VB	0.2194	3.67504	2.28744e-1	0.0302
21	24.928	BV	0.1354	1.83579	2.34330e-1	0.0151

Totals : 1.21537e4 841.02537

Signal 2: DAD1 B, Sig=280,16 Ref=360,100

Peak #	RetTime [min]	Type	Width [min]	Area [mAU*s]	Height [mAU]	Area %
1	0.437	BV	0.0673	219.42157	51.88128	13.7848
2	0.689	VV	0.1473	216.05299	22.24329	13.5732
3	0.877	VV	0.1330	79.39366	7.78529	4.9878
4	1.048	VV	0.0842	33.64267	6.11226	2.1135
5	1.308	VV	0.1756	84.35705	7.71283	5.2996
6	1.571	VV	0.2355	323.92108	18.21588	20.3498
7	2.092	VV	0.1469	59.89788	5.87837	3.7630

Peak #	RetTime [min]	Type	Width [min]	Area [mAU*s]	Height [mAU]	Area %
8	2.263	VV	0.2137	95.81025	6.08635	6.0191
9	2.584	VV	0.1792	42.05276	3.37783	2.6419
10	2.898	VV	0.2421	74.00230	4.35850	4.6491
11	3.156	VV	0.2656	62.92209	3.31254	3.9530
12	3.602	VV	0.2713	32.63747	1.67501	2.0504
13	4.028	VV	0.3215	39.75215	1.74699	2.4974
14	4.601	VV	0.4768	91.20404	2.86951	5.7297
15	5.258	VV	0.3095	35.49369	1.64678	2.2298
16	5.654	VV	0.2825	40.19329	2.03477	2.5251
17	5.918	VB	0.3472	42.14965	1.61147	2.6480
18	11.198	BB	0.4334	8.68277	2.54881e-1	0.5455
19	12.414	BB	0.4116	10.17645	3.26562e-1	0.6393
Totals :				1591.76380	149.13039	

Signal 3: DAD1 C, Sig=282,8 Ref=360,100

Peak #	RetTime [min]	Type	Width [min]	Area [mAU*s]	Height [mAU]	Area %
1	0.437	BV	0.0689	239.28067	54.86665	10.1308
2	0.692	VV	0.1862	391.24347	28.14657	16.5646
3	1.044	VV	0.0898	57.19658	9.84742	2.4216
4	1.308	VV	0.1750	120.46375	11.06854	5.1002
5	1.570	VV	0.2670	448.61133	21.90007	18.9935
6	2.092	VV	0.1482	92.75941	8.85814	3.9273
7	2.265	VV	0.2274	153.53053	9.07323	6.5002
8	2.580	VV	0.1808	79.03123	6.19839	3.3461
9	2.898	VV	0.2587	129.33138	7.02644	5.4757
10	3.155	VV	0.2873	121.87275	5.79595	5.1599
11	3.599	VV	0.2802	77.04424	3.83856	3.2619
12	4.020	VV	0.3396	87.99258	3.62001	3.7255
13	4.593	VV	0.4982	161.05479	4.49350	6.8188
14	5.256	VV	0.3293	64.41425	2.79095	2.7272
15	5.651	VB	0.5436	126.67941	3.00634	5.3634
16	11.193	BB	0.2885	4.41658	1.89064e-1	0.1870
17	12.411	BB	0.3385	6.99884	2.51663e-1	0.2963
Totals :				2361.92177	180.97149	

Signal 4: DAD1 D, Sig=254,16 Ref=360,100

Peak #	RetTime [min]	Type	Width [min]	Area [mAU*s]	Height [mAU]	Area %
1	0.437	BV	0.0669	201.49213	48.06653	11.9317
2	0.647	VV	0.1129	91.95000	11.54531	5.4450
3	0.917	VV	0.1476	91.74554	9.25974	5.4329
4	1.142	VV	0.1156	300.63019	36.67782	17.8024
5	1.356	VV	0.1048	68.04810	9.14353	4.0296
6	1.589	VV	0.2672	201.68182	10.73254	11.9430
7	2.095	VV	0.1655	69.41134	5.88847	4.1103
8	2.278	VV	0.2065	80.90812	5.67009	4.7911
9	2.634	VV	0.2364	87.99241	5.07083	5.2106
10	3.159	VV	0.3247	61.44711	2.59059	3.6387
11	3.618	VV	0.2669	27.96449	1.50396	1.6560
12	4.039	VV	0.3084	50.89342	2.31601	3.0137
13	4.791	VV	0.4906	162.95790	4.29564	9.6499
14	5.667	VV	0.2389	25.67639	1.52146	1.5205
15	5.953	VV	0.5737	82.63798	1.92933	4.8936
16	7.675	VV	1.0463	56.62408	6.39919e-1	3.3531
17	12.450	BB	0.3843	13.77340	4.56109e-1	0.8156
18	23.702	BB	0.5993	12.87339	2.54852e-1	0.7623
Totals :				1688.70780	157.56274	

===

*** End of Report ***
