# Supplementary material for: Influence of Silver Nanoparticles (AgNPs) on Vegetative Growth and Concentrations of Nutrients and Phytohormones in Tomato
Source: Plants (Basel). 2026 Jan 28;15(3):405. doi: 10.3390/plants15030405 (PMC12899181; doi:10.3390/plants15030405)
Supplement: Supplementary file 1 [file plants-15-00405-s001.zip › S1. HPLC Analysis (plants-4015186)/cv. Rio Grande/Leaves/Control/RG-T-L-R1.pdf]

Data File C:\CHEM32\...ORMTMG\FITOHOR GABY Y ALE 30-11-2020 2012-10-03 09-08-53\016-1601.D
Sample Name: TESTIGO RIO GRANDE HOJA R1

===

```
Acq. Operator   : TMG                            Seq. Line :  16
Acq. Instrument : Instrument 1                    Location : Vial 16
Injection Date  : 10/3/2012 5:37:10 PM                 Inj :   1
                                                Inj Volume : 200.0 µl
Different Inj Volume from Sequence !     Actual Inj Volume : 50.0 µl
Acq. Method     : C:\CHEM32\1\DATA\FITOHORMTMG\FITOHOR GABY Y ALE 30-11-2020 2012-10-03 09-08-
                  53\FITOHORMONAS DR SOTO.M
Last changed    : 8/14/2013 11:13:25 AM by TMG
Analysis Method : C:\CHEM32\1\METHODS\LAVADO COLUMNA ACET.M
Last changed    : 10/21/2012 12:24:49 PM by TMG
                  (modified after loading)
```

DAD1 A, Sig=220,4 Ref=360,100 (FITOHORMTMG\FITOHOR GABY Y ALE 30-11-2020 2012-10-03 09-08-53\016-1601.D)

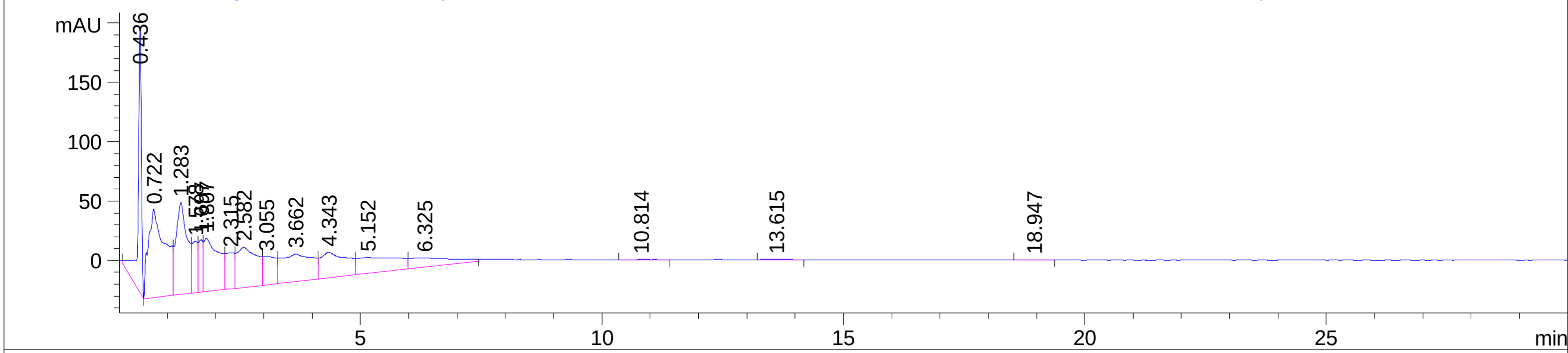

DAD1 B, Sig=280,16 Ref=360,100 (FITOHORMTM...ITOHOR GABY Y ALE 30-11-2020 2012-10-03 09-08-53\016-1601.D)

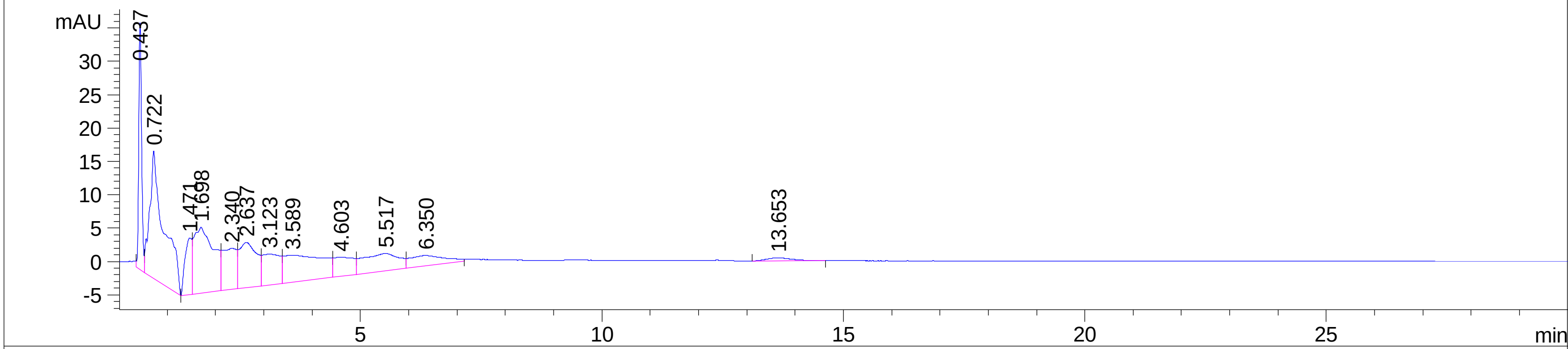

DAD1 C, Sig=282,8 Ref=360,100 (FITOHORMTMG\FITOHOR GABY Y ALE 30-11-2020 2012-10-03 09-08-53\016-1601.D)

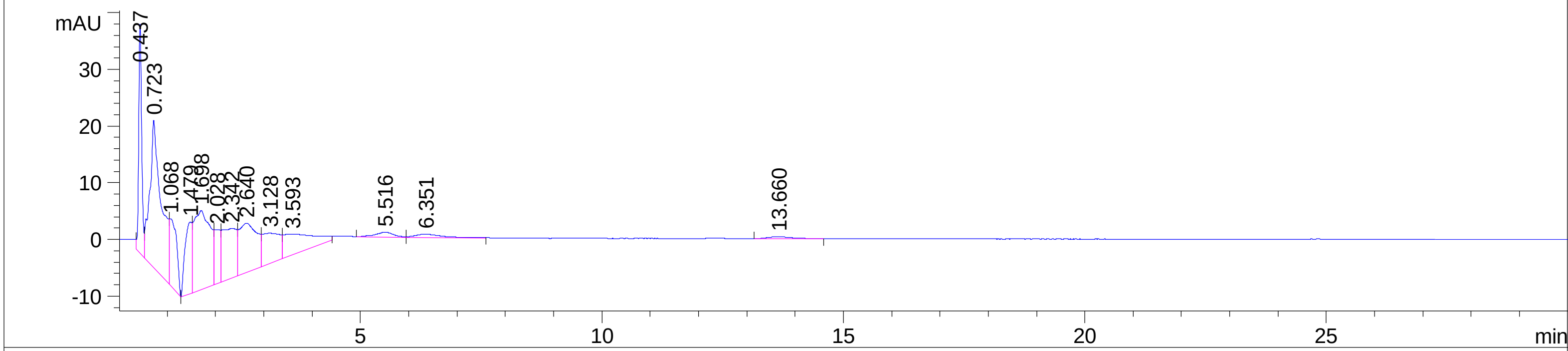

DAD1 D, Sig=254,16 Ref=360,100 (FITOHORMTM...ITOHOR GABY Y ALE 30-11-2020 2012-10-03 09-08-53\016-1601.D)

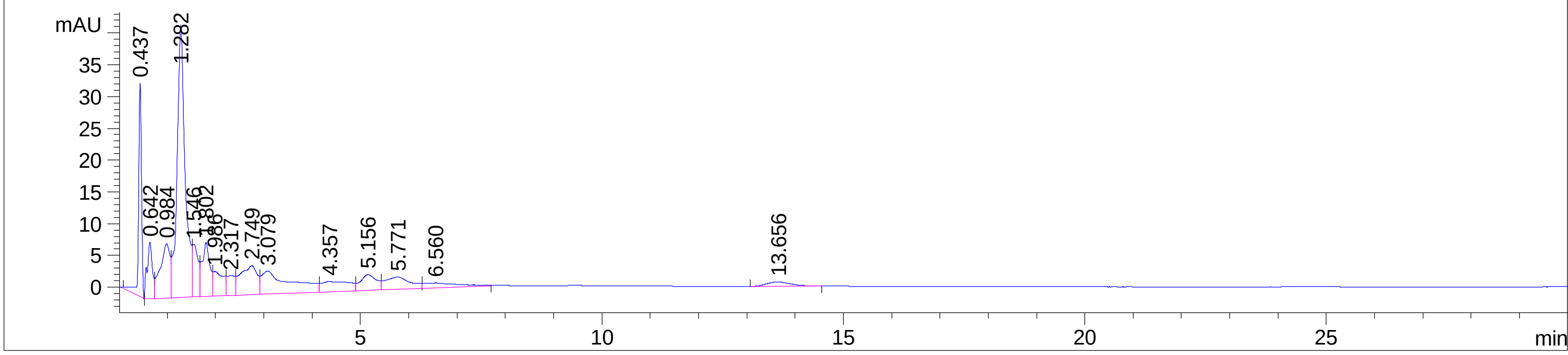

===

Area Percent Report

===

Sorted By : Signal
Multiplier: : 1.0000
Dilution: : 1.0000
Use Multiplier & Dilution Factor with ISTDs

Signal 1: DAD1 A, Sig=220,4 Ref=360,100

Peak #	RetTime [min]	Type	Width [min]	Area [mAU*s]	Height [mAU]	Area %
1	0.436	BV	0.0732	1094.23486	223.21132	10.2668
2	0.722	VV	0.2958	1769.78650	73.78148	16.6052
3	1.283	VV	0.2324	1250.81616	77.44706	11.7359
4	1.578	VV	0.1099	336.44897	43.65545	3.1568
5	1.697	VV	0.0934	293.20938	44.21637	2.7511
6	1.807	VV	0.2886	975.11804	45.03373	9.1491
7	2.315	VV	0.1759	376.64212	30.52991	3.5339
8	2.582	VV	0.3849	986.68787	33.76666	9.2577
9	3.055	VV	0.2561	427.51895	23.97243	4.0112
10	3.662	VV	0.5836	1051.00403	23.06406	9.8611
11	4.343	VV	0.4958	821.54620	21.59808	7.7082
12	5.152	VV	0.7117	764.05176	13.51164	7.1688
13	6.325	VB	0.7465	474.55307	7.70600	4.4525
14	10.814	BB	0.3594	9.48194	3.37859e-1	0.0890
15	13.615	BB	0.4105	19.37254	5.84756e-1	0.1818
16	18.947	BB	0.3106	7.55460	3.07526e-1	0.0709

Totals : 1.06580e4 662.72432

Signal 2: DAD1 B, Sig=280,16 Ref=360,100

Peak #	RetTime [min]	Type	Width [min]	Area [mAU*s]	Height [mAU]	Area %
1	0.437	BV	0.0662	152.83185	37.01162	8.2546
2	0.722	VV	0.2557	385.01740	19.05610	20.7953
3	1.471	VV	0.1604	80.40601	8.49942	4.3428
4	1.698	VV	0.3567	285.37762	9.88787	15.4136
5	2.340	VV	0.2625	121.90131	6.16951	6.5840
6	2.637	VV	0.3527	172.99849	6.80233	9.3439
7	3.123	VV	0.3268	115.77633	4.61334	6.2532
8	3.589	VB	0.6798	221.64871	4.05438	11.9715
9	4.603	BV	0.3530	79.18783	2.82532	4.2770
10	5.517	VV	0.6962	139.91122	2.61502	7.5568
11	6.350	VB	0.6471	79.63900	1.57185	4.3014
12	13.653	BB	0.4525	16.76838	4.46162e-1	0.9057

Peak #	RetTime [min]	Type	Width [min]	Area [mAU*s]	Height [mAU]	Area %
Totals :				1851.46414	103.55293	

Signal 3: DAD1 C, Sig=282,8 Ref=360,100

Peak #	RetTime [min]	Type	Width [min]	Area [mAU*s]	Height [mAU]	Area %
1	0.437	BV	0.0684	174.80298	40.44053	8.7239
2	0.723	VV	0.2109	425.87817	26.02173	21.2542
3	1.068	VV	0.1447	126.65336	11.84907	6.3209
4	1.479	VV	0.1621	120.80071	12.57715	6.0288
5	1.698	VV	0.3048	332.62863	13.91985	16.6004
6	2.028	VV	0.1201	79.74963	9.47986	3.9801
7	2.342	VV	0.2731	179.98615	8.70768	8.9825
8	2.640	VV	0.3559	222.82767	8.66922	11.1206
9	3.128	VV	0.3198	132.47394	5.33266	6.6114
10	3.593	VB	0.5365	153.24861	3.70587	7.6482
11	5.516	BV	0.3748	21.97871	8.46901e-1	1.0969
12	6.351	VB	0.4200	19.05890	5.70188e-1	0.9512
13	13.660	BB	0.4500	13.64534	3.63450e-1	0.6810
Totals :				2003.73280	142.48417	

Signal 4: DAD1 D, Sig=254,16 Ref=360,100

Peak #	RetTime [min]	Type	Width [min]	Area [mAU*s]	Height [mAU]	Area %
1	0.437	BV	0.0682	144.62794	33.64286	8.9945
2	0.642	VV	0.1105	68.19367	8.79524	4.2410
3	0.984	VV	0.1996	120.57629	8.49503	7.4987
4	1.282	VV	0.1729	494.74796	42.79233	30.7687
5	1.546	VV	0.1163	68.46165	8.29413	4.2577
6	1.802	VV	0.1549	96.42696	8.46751	5.9969
7	1.986	VV	0.1904	54.62174	3.87585	3.3970
8	2.317	VV	0.1657	36.73021	3.11205	2.2843
9	2.749	VV	0.3227	111.83527	4.58270	6.9551
10	3.079	VV	0.5473	153.34659	3.61230	9.5367
11	4.357	VV	0.5033	65.47542	1.64160	4.0720
12	5.156	VV	0.3297	58.25817	2.50171	3.6231
13	5.771	VV	0.4858	70.01196	1.93634	4.3541
14	6.560	VB	0.6119	39.96070	7.85047e-1	2.4852
15	13.656	BB	0.4709	24.68546	6.84298e-1	1.5352
Totals :				1607.95998	133.21898	

===

*** End of Report ***
