# Supplementary material for: Influence of Silver Nanoparticles (AgNPs) on Vegetative Growth and Concentrations of Nutrients and Phytohormones in Tomato
Source: Plants (Basel). 2026 Jan 28;15(3):405. doi: 10.3390/plants15030405 (PMC12899181; doi:10.3390/plants15030405)
Supplement: Supplementary file 1 [file plants-15-00405-s001.zip › S1. HPLC Analysis (plants-4015186)/cv. Rio Grande/Leaves/Control/RG-T-L-R2.pdf]

Data File C:\CHEM32\...ORMTMG\FITOHOR GABY Y ALE 30-11-2020 2012-10-03 09-08-53\017-1701.D
Sample Name: TESTIGO RIO GRANDE HOJA R2

===

Acq. Operator : TMG   Seq. Line : 17
Acq. Instrument : Instrument 1   Location : Vial 17
Injection Date : 10/3/2012 6:09:17 PM   Inj : 1
Inj Volume : 200.0 µl
Different Inj Volume from Sequence !   Actual Inj Volume : 50.0 µl
Acq. Method : C:\CHEM32\1\DATA\FITOHORMTMG\FITOHOR GABY Y ALE 30-11-2020 2012-10-03 09-08-53\FITOHORMONAS DR SOTO.M
Last changed : 8/14/2013 11:13:25 AM by TMG
Analysis Method : C:\CHEM32\1\METHODS\LAVADO COLUMNA ACET.M
Last changed : 10/21/2012 12:24:49 PM by TMG
(modified after loading)

DAD1 A, Sig=220,4 Ref=360,100 (FITOHORMTMG\FITOHOR GABY Y ALE 30-11-2020 2012-10-03 09-08-53\017-1701.D)

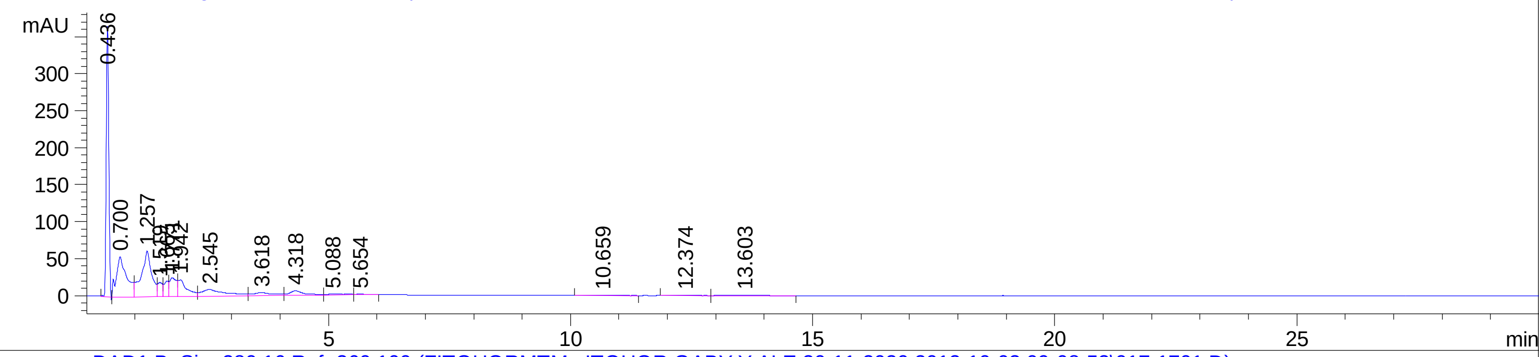

DAD1 B, Sig=280,16 Ref=360,100 (FITOHORMTM...ITOHOR GABY Y ALE 30-11-2020 2012-10-03 09-08-53\017-1701.D)

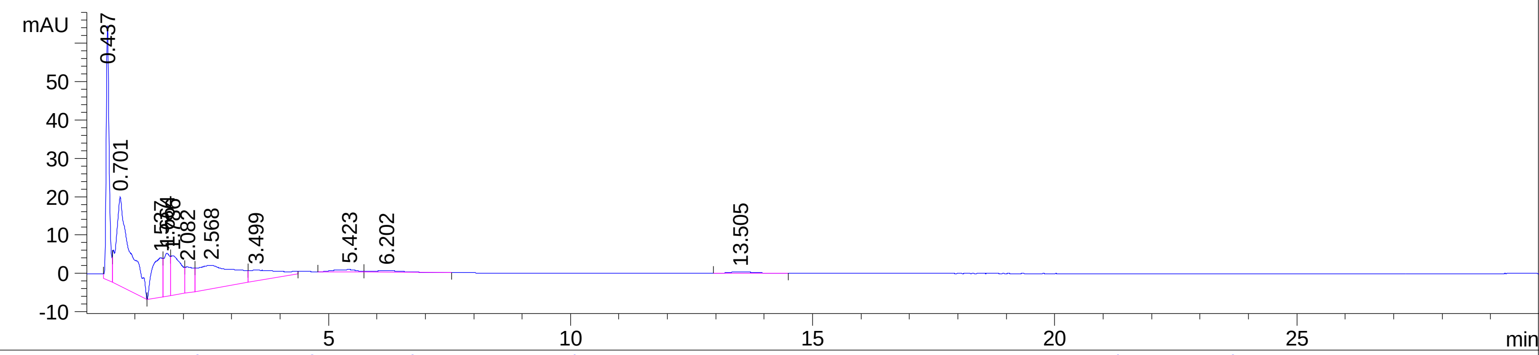

DAD1 C, Sig=282,8 Ref=360,100 (FITOHORMTMG\FITOHOR GABY Y ALE 30-11-2020 2012-10-03 09-08-53\017-1701.D)

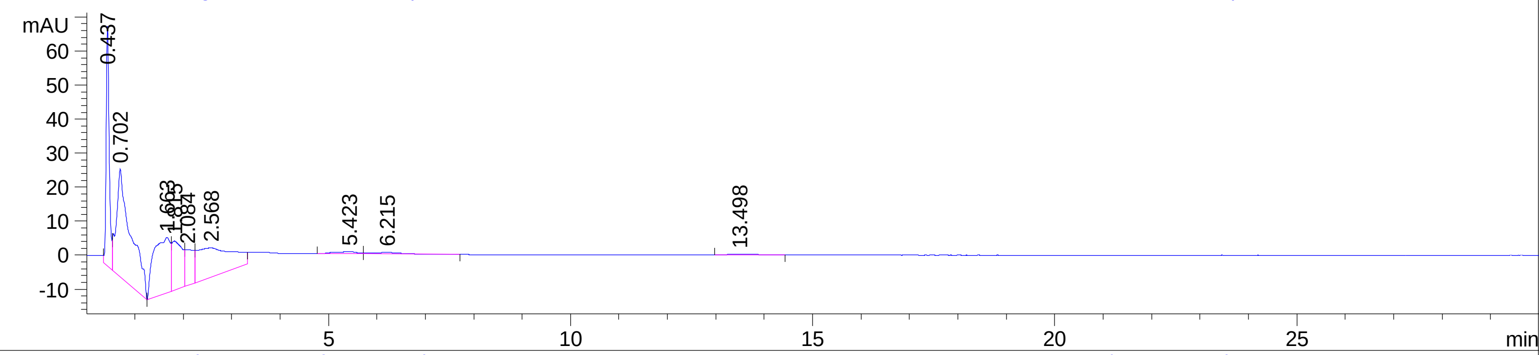

DAD1 D, Sig=254,16 Ref=360,100 (FITOHORMTM...ITOHOR GABY Y ALE 30-11-2020 2012-10-03 09-08-53\017-1701.D)

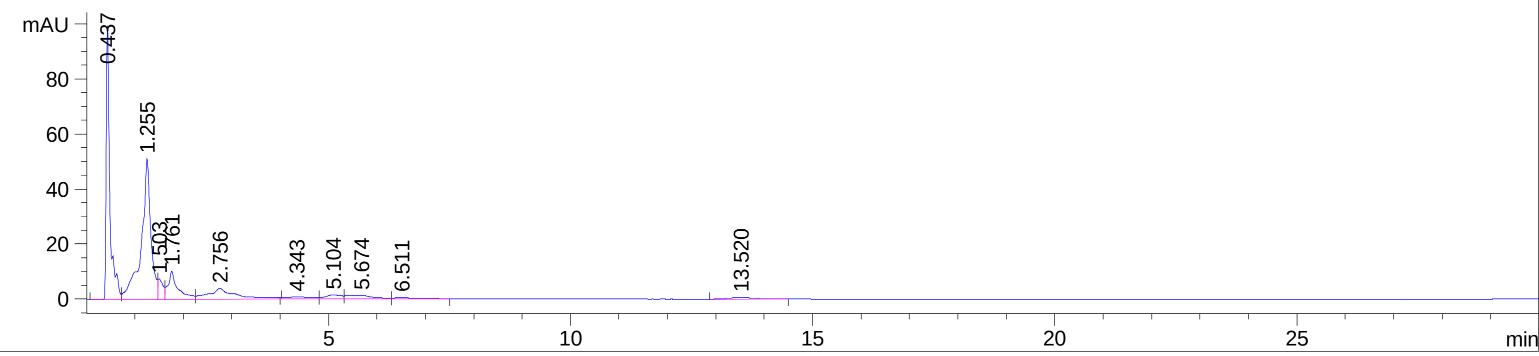

===

Area Percent Report

===

Sorted By : Signal
Multiplier: : 1.0000
Dilution: : 1.0000
Use Multiplier & Dilution Factor with ISTDs

Signal 1: DAD1 A, Sig=220,4 Ref=360,100

Peak #	RetTime [min]	Type	Width [min]	Area [mAU*s]	Height [mAU]	Area %
1	0.436	BV	0.0590	1338.73572	363.07153	29.5067
2	0.700	VV	0.2050	807.11487	54.42668	17.7894
3	1.257	VV	0.1940	916.00732	61.41972	20.1894
4	1.519	VV	0.1048	137.73743	18.94520	3.0358
5	1.665	VV	0.0875	128.47255	20.96896	2.8316
6	1.771	VV	0.1385	259.43674	25.53764	5.7182
7	1.942	VV	0.1736	283.33994	22.09937	6.2450
8	2.545	VB	0.4588	310.65854	8.99754	6.8471
9	3.618	BV	0.3879	123.04549	4.27310	2.7120
10	4.318	VB	0.3054	133.43195	6.14247	2.9409
11	5.088	BB	0.3892	37.76799	1.19358	0.8324
12	5.654	BB	0.2737	15.94407	7.82652e-1	0.3514
13	10.659	BB	0.4417	8.60684	2.55077e-1	0.1897
14	12.374	BV	0.4321	11.22700	3.34020e-1	0.2475
15	13.603	VB	0.5539	25.53586	5.79665e-1	0.5628

Totals : 4537.06230 589.02721

Signal 2: DAD1 B, Sig=280,16 Ref=360,100

Peak #	RetTime [min]	Type	Width [min]	Area [mAU*s]	Height [mAU]	Area %
1	0.437	BV	0.0677	282.28354	66.21624	16.3779
2	0.701	VV	0.2540	462.57498	23.26313	26.8383
3	1.537	VV	0.1930	151.82620	10.24126	8.8089
4	1.664	VV	0.1229	98.92771	11.21809	5.7397
5	1.786	VV	0.2062	155.49077	10.29807	9.0215
6	2.082	VV	0.1733	81.29142	6.71107	4.7165
7	2.568	VV	0.6979	321.47083	6.10893	18.6515
8	3.499	VB	0.5096	116.48132	2.83370	6.7582
9	5.423	BV	0.4200	19.01251	6.16198e-1	1.1031
10	6.202	VB	0.5441	18.70770	4.39955e-1	1.0854
11	13.505	BB	0.5022	15.49605	4.06971e-1	0.8991

Totals : 1723.56302 138.35363

Signal 3: DAD1 C, Sig=282,8 Ref=360,100

Peak #	RetTime [min]	Type	Width [min]	Area [mAU*s]	Height [mAU]	Area %
1	0.437	BV	0.0694	311.23398	70.62861	14.1422
2	0.702	VV	0.2635	657.87390	31.77946	29.8933
3	1.663	VV	0.3049	392.04318	16.28413	17.8141
4	1.815	VV	0.2009	213.95099	14.43819	9.7218
5	2.084	VV	0.1787	131.40923	10.59572	5.9711
6	2.568	VV	0.6830	442.74557	8.62329	20.1180
7	5.423	BV	0.4174	19.79367	6.60923e-1	0.8994
8	6.215	VB	0.5015	19.56350	4.68152e-1	0.8889
9	13.498	BB	0.4505	12.12911	3.22703e-1	0.5511

Totals : 2200.74313 153.80117

Signal 4: DAD1 D, Sig=254,16 Ref=360,100

Peak #	RetTime [min]	Type	Width [min]	Area [mAU*s]	Height [mAU]	Area %
1	0.437	BV	0.0804	533.23212	99.63196	30.4030
2	1.255	VV	0.1844	710.65100	51.06203	40.5188
3	1.503	VV	0.1048	55.46305	7.45497	3.1623
4	1.761	VV	0.1876	145.24834	10.23058	8.2815
5	2.756	VB	0.4975	156.43974	3.98958	8.9196
6	4.343	BV	0.4604	29.48932	8.26181e-1	1.6814
7	5.104	VV	0.3187	33.84591	1.51482	1.9298
8	5.674	VV	0.5044	48.94160	1.29075	2.7905
9	6.511	VB	0.4966	16.76839	4.24734e-1	0.9561
10	13.520	BB	0.4894	23.80092	6.17857e-1	1.3570

Totals : 1753.88039 177.04347

===

*** End of Report ***
