# Supplementary material for: Influence of Silver Nanoparticles (AgNPs) on Vegetative Growth and Concentrations of Nutrients and Phytohormones in Tomato
Source: Plants (Basel). 2026 Jan 28;15(3):405. doi: 10.3390/plants15030405 (PMC12899181; doi:10.3390/plants15030405)
Supplement: Supplementary file 1 [file plants-15-00405-s001.zip › S1. HPLC Analysis (plants-4015186)/cv. Rio Grande/Leaves/Control/RG-T-L-R3.pdf]

Data File C:\CHEM32\...ORMTMG\FITOHOR GABY Y ALE 30-11-2020 2012-10-03 09-08-53\018-1801.D
Sample Name: TESTIGO RIO GRANDE HOJA R3

===

```
Acq. Operator   : TMG                          Seq. Line :  18
Acq. Instrument : Instrument 1                  Location : Vial 18
Injection Date  : 10/3/2012 6:41:23 PM               Inj :   1
                                              Inj Volume : 200.0 µl
Different Inj Volume from Sequence !   Actual Inj Volume : 50.0 µl
Acq. Method     : C:\CHEM32\1\DATA\FITOHORMTMG\FITOHOR GABY Y ALE 30-11-2020 2012-10-03 09-08-
                  53\FITOHORMONAS DR SOTO.M
Last changed    : 8/14/2013 11:13:25 AM by TMG
Analysis Method : C:\CHEM32\1\METHODS\LAVADO COLUMNA ACET.M
Last changed    : 10/21/2012 12:24:49 PM by TMG
                  (modified after loading)
```

DAD1 A, Sig=220,4 Ref=360,100 (FITOHORMTMG\FITOHOR GABY Y ALE 30-11-2020 2012-10-03 09-08-53\018-1801.D)

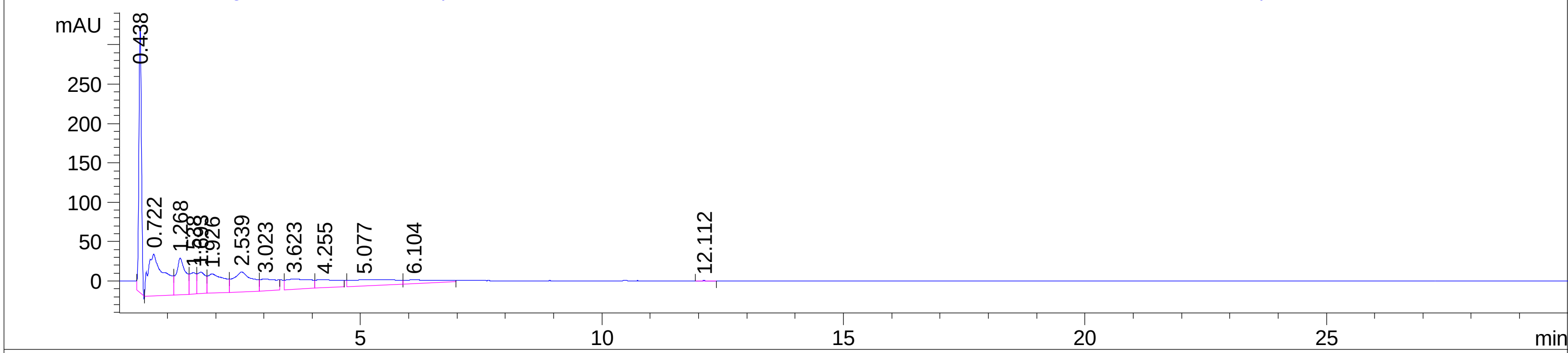

DAD1 B, Sig=280,16 Ref=360,100 (FITOHORMTM...ITOHOR GABY Y ALE 30-11-2020 2012-10-03 09-08-53\018-1801.D)

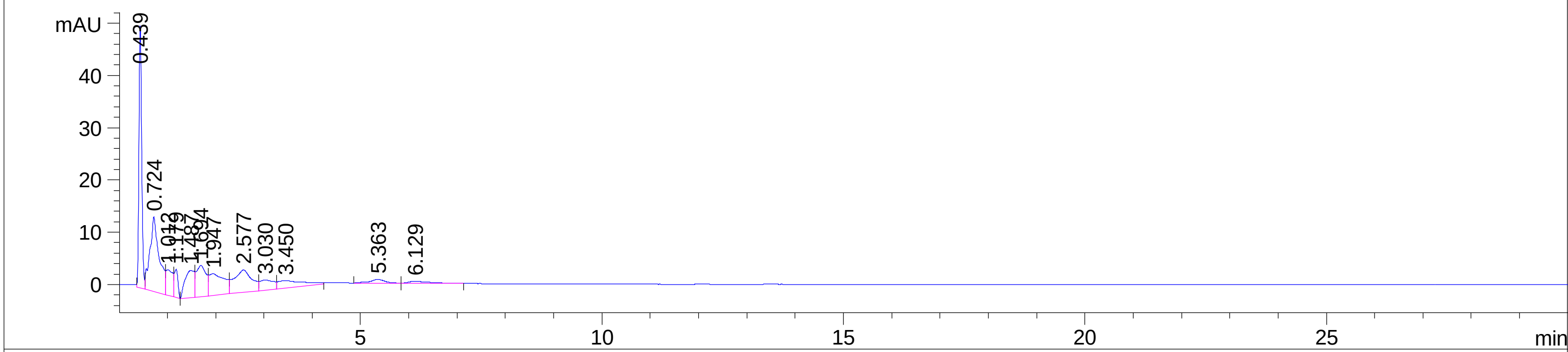

DAD1 C, Sig=282,8 Ref=360,100 (FITOHORMTMG\FITOHOR GABY Y ALE 30-11-2020 2012-10-03 09-08-53\018-1801.D)

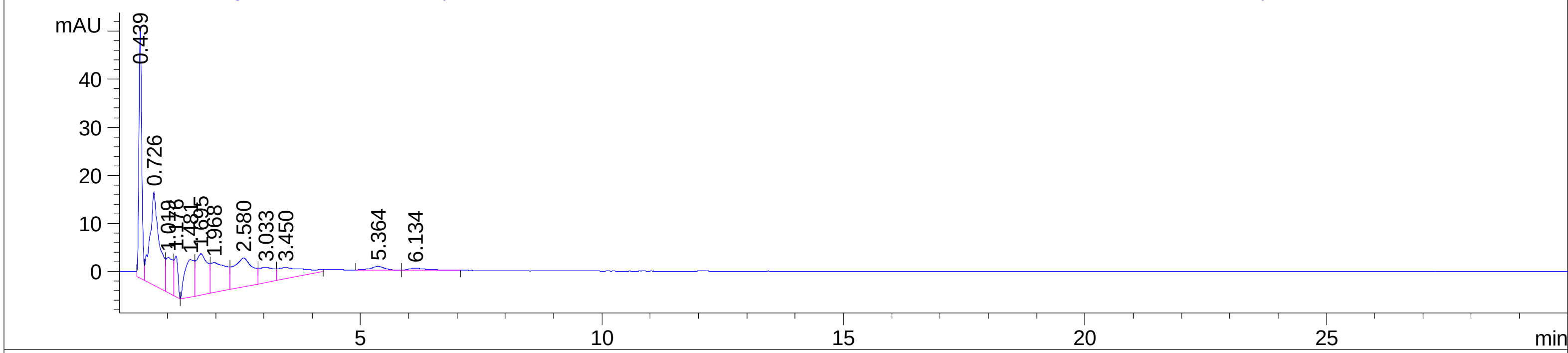

DAD1 D, Sig=254,16 Ref=360,100 (FITOHORMTM...ITOHOR GABY Y ALE 30-11-2020 2012-10-03 09-08-53\018-1801.D)

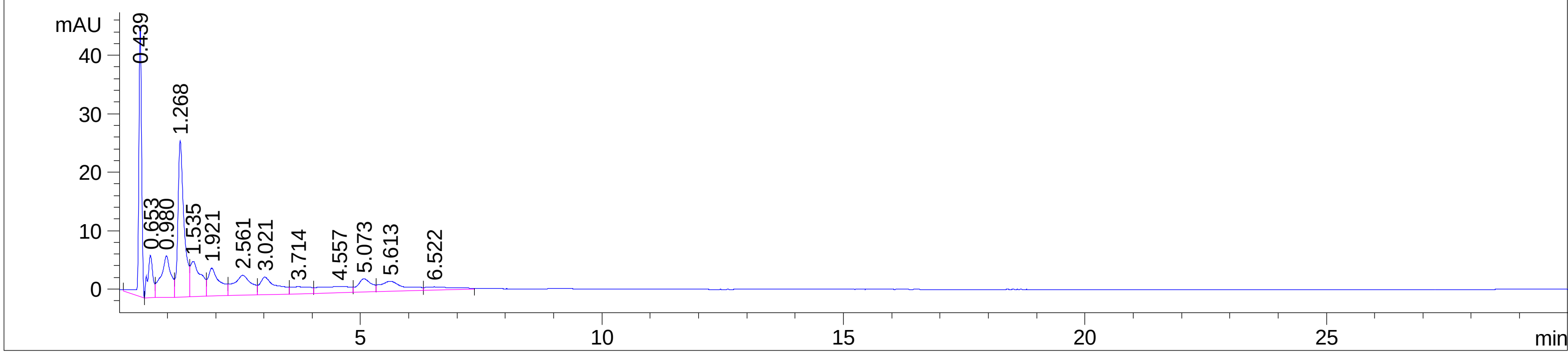

===

Area Percent Report

===

Sorted By : Signal
Multiplier: : 1.0000
Dilution: : 1.0000
Use Multiplier & Dilution Factor with ISTDs

Signal 1: DAD1 A, Sig=220,4 Ref=360,100

Peak #	RetTime [min]	Type	Width [min]	Area [mAU*s]	Height [mAU]	Area %
1	0.438	BV	0.0594	1248.73572	335.84320	18.5020
2	0.722	VV	0.2861	1206.73096	52.90215	17.8797
3	1.268	VV	0.1831	636.21661	46.65518	9.4266
4	1.538	VV	0.1252	244.10133	27.07594	3.6168
5	1.693	VV	0.1664	324.46490	27.35813	4.8075
6	1.926	VV	0.3040	569.23462	24.41512	8.4341
7	2.539	VV	0.3678	696.67120	25.09835	10.3223
8	3.023	VB	0.3249	351.11917	14.58324	5.2024
9	3.623	BV	0.4422	455.56461	13.21514	6.7499
10	4.255	VB	0.4424	343.47321	9.86083	5.0891
11	5.077	BV	0.7489	468.57150	7.88954	6.9426
12	6.104	VB	0.5873	199.59453	4.45008	2.9573
13	12.112	BB	0.1984	4.70747	3.22380e-1	0.0697

Totals : 6749.18583 589.66927

Signal 2: DAD1 B, Sig=280,16 Ref=360,100

Peak #	RetTime [min]	Type	Width [min]	Area [mAU*s]	Height [mAU]	Area %
1	0.439	BV	0.0625	200.65610	50.37470	21.3832
2	0.724	VV	0.1755	190.80804	14.31732	20.3337
3	1.012	VV	0.1300	47.23154	4.92179	5.0333
4	1.179	VV	0.0841	29.40956	5.35687	3.1341
5	1.487	VV	0.2309	70.52082	5.17748	7.5151
6	1.694	VV	0.1970	83.69727	5.98952	8.9193
7	1.947	VV	0.2895	90.51737	4.13369	9.6461
8	2.577	VV	0.3424	108.56484	4.27392	11.5694
9	3.030	VV	0.2757	37.92617	1.89358	4.0417
10	3.450	VB	0.4676	51.23734	1.43128	5.4602
11	5.363	BV	0.3120	15.61132	7.17141e-1	1.6636
12	6.129	VB	0.4072	12.20235	4.14753e-1	1.3004

Totals : 938.38272 99.00203

Signal 3: DAD1 C, Sig=282,8 Ref=360,100

Peak #	RetTime [min]	Type	Width [min]	Area [mAU*s]	Height [mAU]	Area %
1	0.439	BV	0.0636	215.14157	52.69720	16.2159
2	0.726	VV	0.1771	261.06534	19.39337	19.6774
3	1.019	VV	0.1304	70.60911	7.32741	5.3220
4	1.176	VV	0.0856	47.63460	8.46850	3.5904
5	1.481	VV	0.2155	102.69981	7.80693	7.7408
6	1.695	VV	0.2278	142.75455	8.59178	10.7599
7	1.968	VV	0.2863	138.04988	6.23596	10.4053
8	2.580	VV	0.3717	167.88867	6.01351	12.6543
9	3.033	VV	0.2844	66.26586	3.18997	4.9947
10	3.450	VB	0.4835	87.33791	2.35072	6.5830
11	5.364	BV	0.2940	15.37861	7.66676e-1	1.1591
12	6.134	VB	0.3817	11.90276	4.34333e-1	0.8972
Totals :				1326.72867	123.27635	

Signal 4: DAD1 D, Sig=254,16 Ref=360,100

Peak #	RetTime [min]	Type	Width [min]	Area [mAU*s]	Height [mAU]	Area %
1	0.439	BV	0.0635	189.34526	46.47554	16.8870
2	0.653	VV	0.1094	54.37320	7.25533	4.8494
3	0.980	VV	0.1915	101.96163	7.10212	9.0936
4	1.268	VV	0.1285	239.74702	26.75990	21.3822
5	1.535	VV	0.1961	89.09203	6.03962	7.9458
6	1.921	VV	0.2333	82.15654	4.76208	7.3273
7	2.561	VV	0.3462	87.14222	3.40909	7.7719
8	3.021	VV	0.3211	72.37342	3.00359	6.4547
9	3.714	VV	0.3621	33.34639	1.18573	2.9740
10	4.557	VV	0.5709	48.56310	1.07534	4.3312
11	5.073	VV	0.2824	43.72917	2.23330	3.9001
12	5.613	VV	0.4626	57.87983	1.71114	5.1621
13	6.522	VB	0.4901	21.53579	5.41330e-1	1.9207
Totals :				1121.24560	111.55412	

==

*** End of Report ***
