# Supplementary material for: Influence of Silver Nanoparticles (AgNPs) on Vegetative Growth and Concentrations of Nutrients and Phytohormones in Tomato
Source: Plants (Basel). 2026 Jan 28;15(3):405. doi: 10.3390/plants15030405 (PMC12899181; doi:10.3390/plants15030405)
Supplement: Supplementary file 1 [file plants-15-00405-s001.zip › S1. HPLC Analysis (plants-4015186)/cv. Rio Grande/Roots/10 ppm/RG-10-R-R1.pdf]

Data File C:\CHEM32\...ORMTMG\FITOHOR GABY Y ALE 30-11-2020 2012-10-03 09-08-53\043-4301.D
Sample Name: 10 PPM RIO GRANDE RAIZ R1

===

Acq. Operator : TMG Seq. Line : 43
Acq. Instrument : Instrument 1 Location : Vial 43
Injection Date : 10/4/2012 8:04:18 AM Inj : 1
Inj Volume : 200.0 µl
Different Inj Volume from Sequence ! Actual Inj Volume : 50.0 µl
Acq. Method : C:\CHEM32\1\DATA\FITOHORMTMG\FITOHOR GABY Y ALE 30-11-2020 2012-10-03 09-08-53\FITOHORMONAS DR SOTO.M
Last changed : 8/14/2013 11:13:25 AM by TMG
Analysis Method : C:\CHEM32\1\METHODS\LAVADO COLUMNA ACET.M
Last changed : 10/21/2012 12:24:49 PM by TMG
(modified after loading)

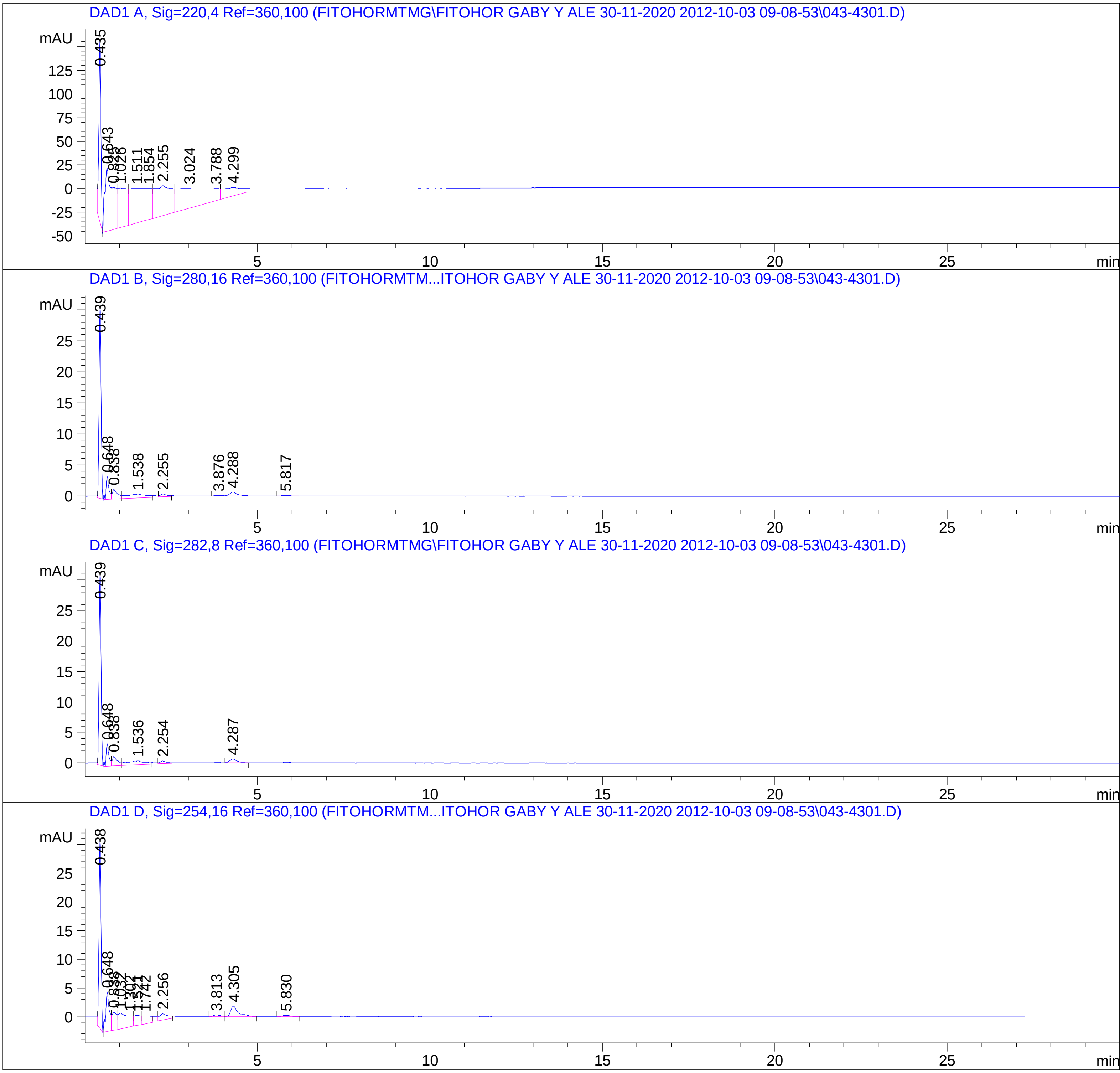

===

Area Percent Report

===

Sorted By : Signal
Multiplier: : 1.0000
Dilution: : 1.0000
Use Multiplier & Dilution Factor with ISTDs

Signal 1: DAD1 A, Sig=220,4 Ref=360,100

Peak #	RetTime [min]	Type	Width [min]	Area [mAU*s]	Height [mAU]	Area %
1	0.435	BV	0.0691	811.61755	192.98656	11.3959
2	0.643	VV	0.1506	744.95795	66.56416	10.4599
3	0.825	VV	0.1310	435.57086	44.19395	6.1158
4	1.026	VV	0.2281	741.47046	41.56240	10.4110
5	1.511	VV	0.3658	1065.15381	36.35856	14.9558
6	1.854	VV	0.1783	432.67859	32.71833	6.0752
7	2.255	VV	0.4341	1101.06714	31.62896	15.4601
8	3.024	VV	0.4653	752.82471	20.65072	10.5704
9	3.788	VV	0.6505	677.48480	12.86337	9.5126
10	4.299	VB	0.5063	359.17715	8.98589	5.0432
Totals :				7122.00302	488.51289	

Signal 2: DAD1 B, Sig=280,16 Ref=360,100

Peak #	RetTime [min]	Type	Width [min]	Area [mAU*s]	Height [mAU]	Area %
1	0.439	BV	0.0659	127.56292	31.09360	62.2076
2	0.648	VV	0.0809	19.65081	3.64300	9.5830
3	0.838	VV	0.1468	16.77468	1.54364	8.1804
4	1.538	VB	0.4665	23.57700	6.24379e-1	11.4976
5	2.255	BB	0.1756	5.54040	4.32178e-1	2.7018
6	3.876	BV	0.1825	9.48025e-1	6.89244e-2	0.4623
7	4.288	VB	0.2307	9.33654	5.89590e-1	4.5531
8	5.817	BB	0.2291	1.66951	9.98528e-2	0.8142
Totals :				205.05988	38.09516	

Signal 3: DAD1 C, Sig=282,8 Ref=360,100

Peak #	RetTime [min]	Type	Width [min]	Area [mAU*s]	Height [mAU]	Area %
1	0.439	BV	0.0659	130.43419	31.74348	64.3720
2	0.648	VV	0.0808	19.62469	3.64718	9.6852
3	0.838	VV	0.1428	15.94670	1.51443	7.8700
4	1.536	VB	0.4596	22.74003	6.11851e-1	11.2227
5	2.254	BB	0.1851	5.25045	3.94983e-1	2.5912
6	4.287	BB	0.2229	8.62950	5.75739e-1	4.2588

Totals : 202.62555 38.48766

Signal 4: DAD1 D, Sig=254,16 Ref=360,100

Peak #	RetTime [min]	Type	Width [min]	Area [mAU*s]	Height [mAU]	Area %
1	0.438	BV	0.0664	138.00455	33.24912	35.8663
2	0.648	VV	0.1081	53.06316	6.87352	13.7907
3	0.838	VV	0.1346	30.71177	3.12551	7.9817
4	1.032	VV	0.1930	41.11340	2.74066	10.6850
5	1.302	VV	0.1334	18.96880	1.95102	4.9298
6	1.521	VV	0.1978	24.81500	1.72527	6.4492
7	1.742	VB	0.2367	24.09457	1.37351	6.2620
8	2.256	BB	0.2289	18.92605	1.09926	4.9187
9	3.813	BV	0.1869	3.53914	2.77284e-1	0.9198
10	4.305	VB	0.2232	27.93076	1.79812	7.2590
11	5.830	BB	0.2295	3.60799	2.19776e-1	0.9377

Totals : 384.77518 54.43305

===

*** End of Report ***
