# Supplementary material for: Influence of Silver Nanoparticles (AgNPs) on Vegetative Growth and Concentrations of Nutrients and Phytohormones in Tomato
Source: Plants (Basel). 2026 Jan 28;15(3):405. doi: 10.3390/plants15030405 (PMC12899181; doi:10.3390/plants15030405)
Supplement: Supplementary file 1 [file plants-15-00405-s001.zip › S1. HPLC Analysis (plants-4015186)/cv. Rio Grande/Roots/10 ppm/RG-10-R-R2.pdf]

Data File C:\CHEM32\...ORMTMG\FITOHOR GABY Y ALE 30-11-2020 2012-10-03 09-08-53\044-4401.D
Sample Name: 10 PPM RIO GRANDE RAIZ R2

===

Acq. Operator : TMG Seq. Line : 44
Acq. Instrument : Instrument 1 Location : Vial 44
Injection Date : 10/4/2012 8:36:25 AM Inj : 1
Inj Volume : 200.0 µl
Different Inj Volume from Sequence ! Actual Inj Volume : 50.0 µl
Acq. Method : C:\CHEM32\1\DATA\FITOHORMTMG\FITOHOR GABY Y ALE 30-11-2020 2012-10-03 09-08-53\FITOHORMONAS DR SOTO.M
Last changed : 8/14/2013 11:13:25 AM by TMG
Analysis Method : C:\CHEM32\1\METHODS\LAVADO COLUMNA ACET.M
Last changed : 10/21/2012 12:24:49 PM by TMG
(modified after loading)

DAD1 A, Sig=220,4 Ref=360,100 (FITOHORMTMG\FITOHOR GABY Y ALE 30-11-2020 2012-10-03 09-08-53\044-4401.D)

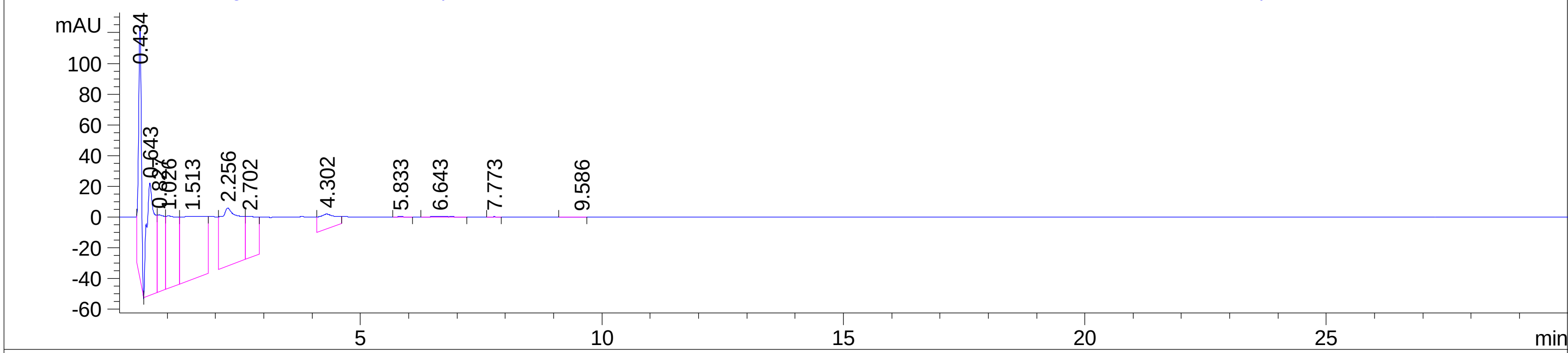

DAD1 B, Sig=280,16 Ref=360,100 (FITOHORMTM...ITOHOR GABY Y ALE 30-11-2020 2012-10-03 09-08-53\044-4401.D)

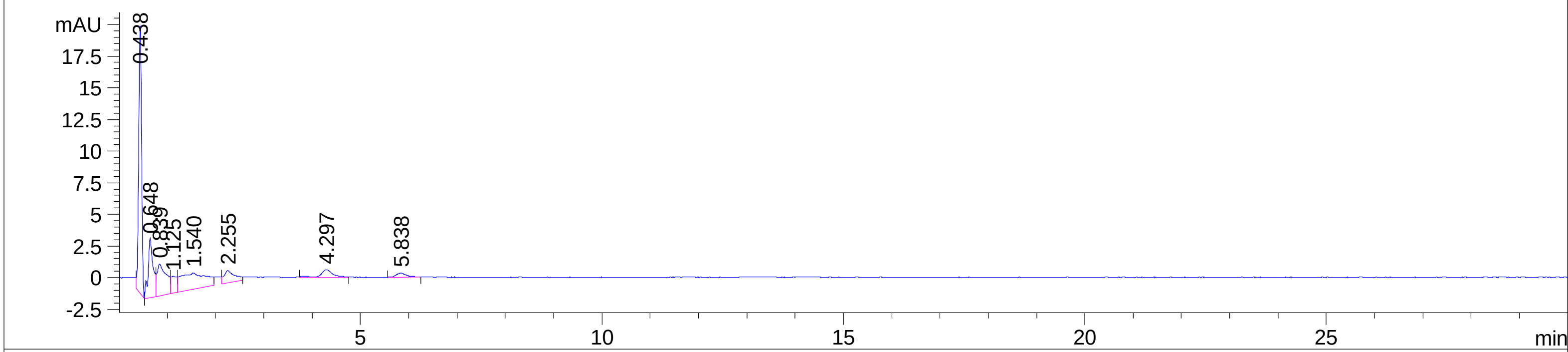

DAD1 C, Sig=282,8 Ref=360,100 (FITOHORMTMG\FITOHOR GABY Y ALE 30-11-2020 2012-10-03 09-08-53\044-4401.D)

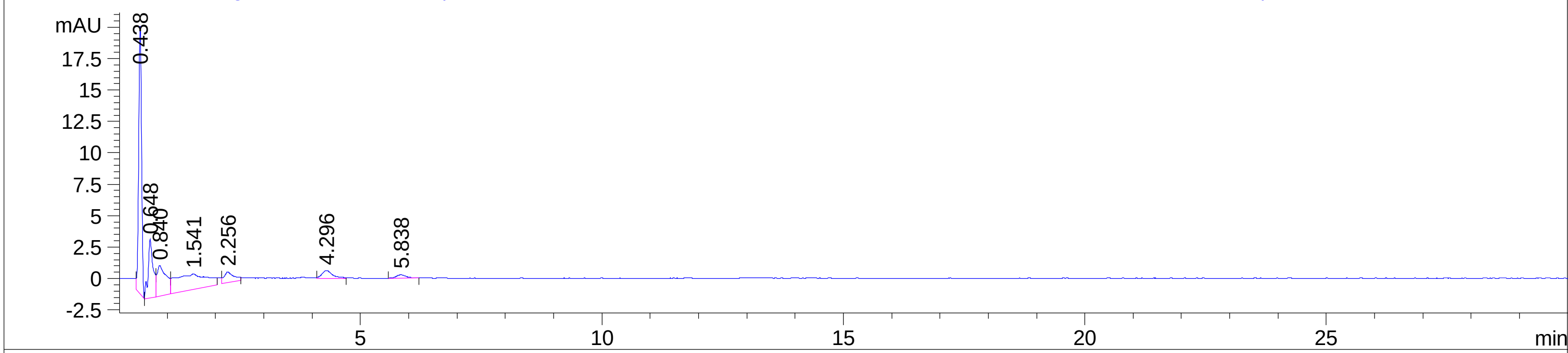

DAD1 D, Sig=254,16 Ref=360,100 (FITOHORMTM...ITOHOR GABY Y ALE 30-11-2020 2012-10-03 09-08-53\044-4401.D)

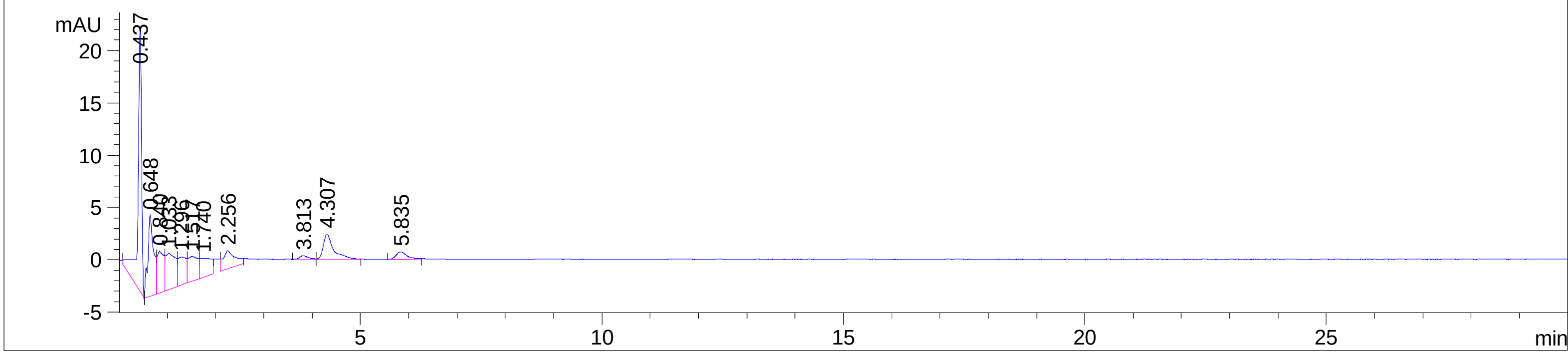

===

Area Percent Report

===

Sorted By : Signal
Multiplier: : 1.0000
Dilution: : 1.0000
Use Multiplier & Dilution Factor with ISTDs

Signal 1: DAD1 A, Sig=220,4 Ref=360,100

Peak #	RetTime [min]	Type	Width [min]	Area [mAU*s]	Height [mAU]	Area %
1	0.434	BV	0.0656	698.13953	164.37627	11.4659
2	0.643	VV	0.1502	835.35425	72.62746	13.7195
3	0.827	VV	0.1405	517.59180	50.10406	8.5007
4	1.026	VV	0.2200	806.60211	47.05144	13.2473
5	1.513	VB	0.4270	1436.08435	41.16543	23.5856
6	2.256	BV	0.3638	1072.73267	37.71722	17.6181
7	2.702	VB	0.2263	458.16534	26.94981	7.5247
8	4.302	BB	0.3381	241.11552	9.56592	3.9600
9	5.833	BB	0.1320	2.75257	3.34646e-1	0.0452
10	6.643	BB	0.3434	14.03708	5.19237e-1	0.2305
11	7.773	BV	0.1241	2.19787	2.78325e-1	0.0361
12	9.586	BV	0.2361	4.03805	2.26416e-1	0.0663

Totals : 6088.81114 450.91623

Signal 2: DAD1 B, Sig=280,16 Ref=360,100

Peak #	RetTime [min]	Type	Width [min]	Area [mAU*s]	Height [mAU]	Area %
1	0.438	BV	0.0667	88.34725	21.15781	36.1638
2	0.648	VV	0.1034	33.86890	4.62642	13.8638
3	0.839	VV	0.1769	33.74391	2.50855	13.8126
4	1.125	VV	0.1110	10.98476	1.32134	4.4965
5	1.540	VB	0.4548	47.00074	1.27280	19.2391
6	2.255	BB	0.2084	14.80007	9.57161e-1	6.0582
7	4.297	BB	0.2500	10.38432	6.05662e-1	4.2507
8	5.838	BB	0.2339	5.16745	3.17427e-1	2.1152

Totals : 244.29740 32.76717

Signal 3: DAD1 C, Sig=282,8 Ref=360,100

Peak #	RetTime [min]	Type	Width [min]	Area [mAU*s]	Height [mAU]	Area %
1	0.438	BV	0.0667	89.00930	21.33533	37.4743
2	0.648	VV	0.1028	33.46641	4.60168	14.0899
3	0.840	VV	0.1738	31.74771	2.44018	13.3663
4	1.541	VB	0.5825	58.12215	1.21841	24.4703
5	2.256	BB	0.1919	11.98214	8.52997e-1	5.0447
6	4.296	BB	0.2268	9.14652	6.10454e-1	3.8508
7	5.838	BB	0.2180	4.04696	2.71364e-1	1.7038
Totals :				237.52120	31.33042	

Signal 4: DAD1 D, Sig=254,16 Ref=360,100

Peak #	RetTime [min]	Type	Width [min]	Area [mAU*s]	Height [mAU]	Area %
1	0.437	BV	0.0792	133.32909	25.41564	28.8547
2	0.648	VV	0.1146	63.56326	7.67737	13.7562
3	0.840	VV	0.1369	40.17748	3.94240	8.6951
4	1.033	VV	0.1838	48.59513	3.46017	10.5168
5	1.296	VV	0.1617	31.52548	2.67048	6.8227
6	1.517	VV	0.1834	31.96100	2.36911	6.9169
7	1.740	VB	0.2134	30.68171	1.85057	6.6400
8	2.256	BB	0.2329	29.69935	1.70760	6.4274
9	3.813	BV	0.1929	4.42212	3.37342e-1	0.9570
10	4.307	VB	0.2204	36.54560	2.38979	7.9091
11	5.835	BB	0.2326	11.57052	7.23476e-1	2.5041
Totals :				462.07074	52.54394	

===

*** End of Report ***
