# Supplementary material for: Influence of Silver Nanoparticles (AgNPs) on Vegetative Growth and Concentrations of Nutrients and Phytohormones in Tomato
Source: Plants (Basel). 2026 Jan 28;15(3):405. doi: 10.3390/plants15030405 (PMC12899181; doi:10.3390/plants15030405)
Supplement: Supplementary file 1 [file plants-15-00405-s001.zip › S1. HPLC Analysis (plants-4015186)/cv. Rio Grande/Roots/10 ppm/RG-10-R-R3.pdf]

Data File C:\CHEM32\...ORMTMG\FITOHOR GABY Y ALE 30-11-2020 2012-10-03 09-08-53\045-4501.D
Sample Name: 10 PPM RIO GRANDE RAIZ R3

=====================================================================

| | | | |
|---|---|---|---|
| Acq. Operator | : TMG | Seq. Line : | 45 |
| Acq. Instrument | : Instrument 1 | Location : | Vial 45 |
| Injection Date | : 10/4/2012 9:08:33 AM | Inj : | 1 |
| | | Inj Volume : | 200.0 µl |
| Different Inj Volume from Sequence ! | | Actual Inj Volume : | 50.0 µl |

Acq. Method : C:\CHEM32\1\DATA\FITOHORMTMG\FITOHOR GABY Y ALE 30-11-2020 2012-10-03 09-08-53\FITOHORMONAS DR SOTO.M
Last changed : 8/14/2013 11:13:25 AM by TMG
Analysis Method : C:\CHEM32\1\METHODS\LAVADO COLUMNA ACET.M
Last changed : 10/21/2012 12:24:49 PM by TMG
(modified after loading)

DAD1 A, Sig=220,4 Ref=360,100 (FITOHORMTMG\FITOHOR GABY Y ALE 30-11-2020 2012-10-03 09-08-53\045-4501.D)

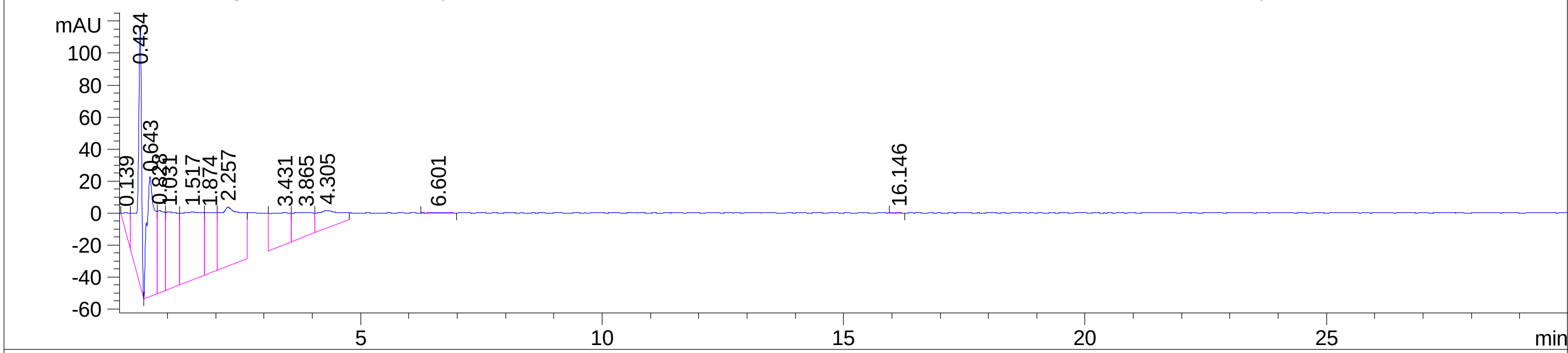


DAD1 B, Sig=280,16 Ref=360,100 (FITOHORMTM...ITOHOR GABY Y ALE 30-11-2020 2012-10-03 09-08-53\045-4501.D)

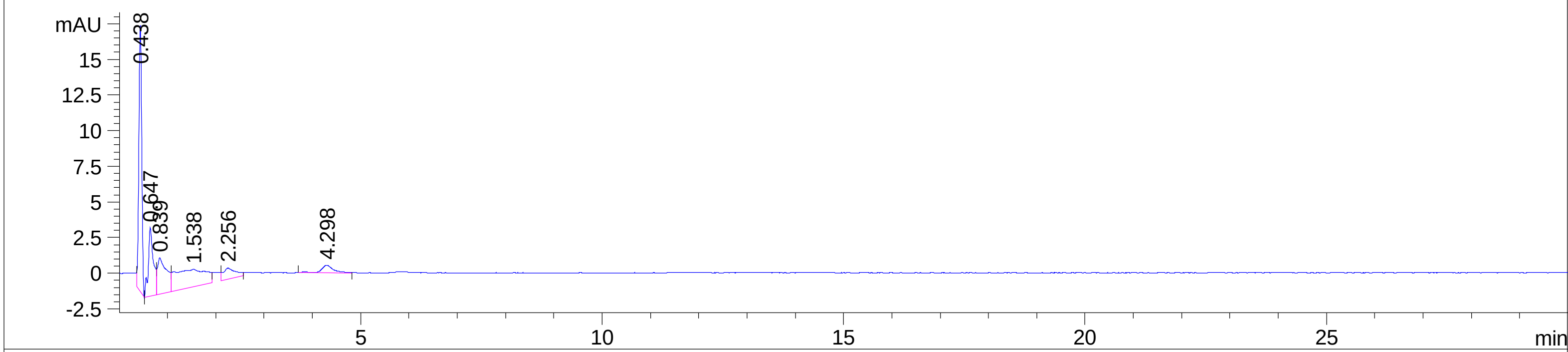


DAD1 C, Sig=282,8 Ref=360,100 (FITOHORMTMG\FITOHOR GABY Y ALE 30-11-2020 2012-10-03 09-08-53\045-4501.D)

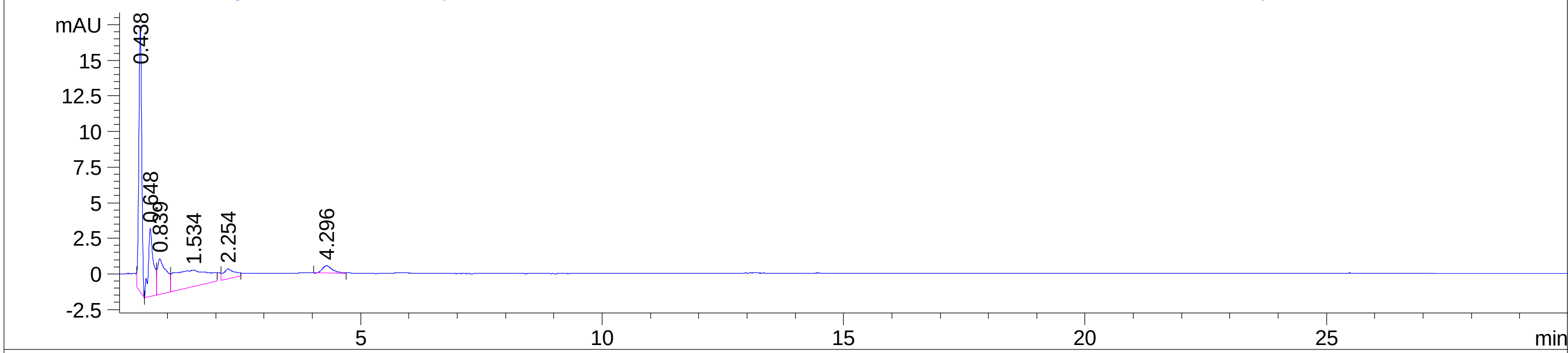


DAD1 D, Sig=254,16 Ref=360,100 (FITOHORMTM...ITOHOR GABY Y ALE 30-11-2020 2012-10-03 09-08-53\045-4501.D)

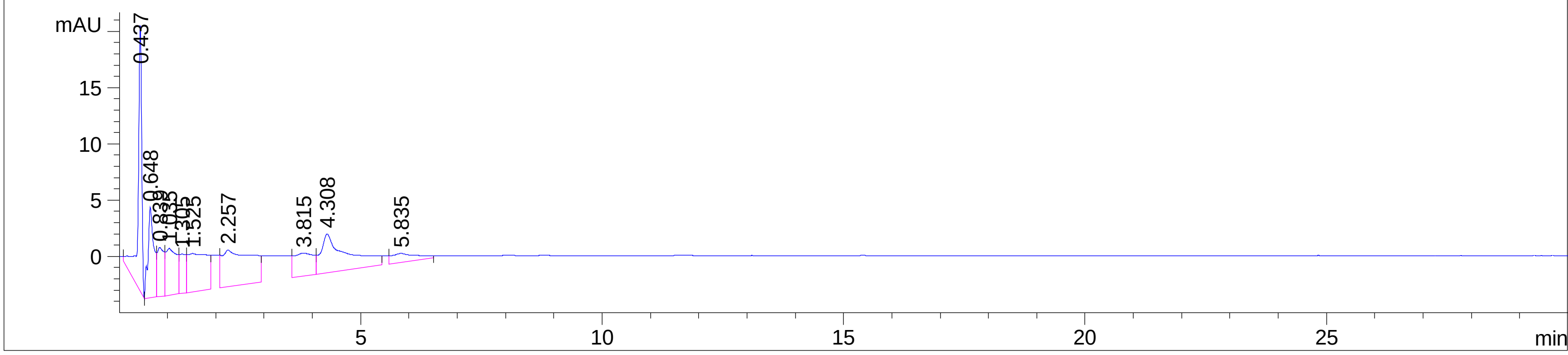

=====================================================================

Area Percent Report

=====================================================================

Sorted By : Signal
Multiplier: : 1.0000
Dilution: : 1.0000
Use Multiplier & Dilution Factor with ISTDs

Signal 1: DAD1 A, Sig=220,4 Ref=360,100

| Peak # | RetTime [min] | Type | Width [min] | Area [mAU*s] | Height [mAU] | Area % |
|---|---|---|---|---|---|---|
| 1 | 0.139 | BV | 0.1598 | 135.07106 | 12.32579 | 1.7173 |
| 2 | 0.434 | VV | 0.0848 | 944.16956 | 160.16486 | 12.0045 |
| 3 | 0.643 | VV | 0.1528 | 847.64490 | 74.47211 | 10.7772 |
| 4 | 0.828 | VV | 0.1394 | 542.88391 | 51.32117 | 6.9024 |
| 5 | 1.031 | VV | 0.2146 | 804.91083 | 48.25490 | 10.2339 |
| 6 | 1.517 | VV | 0.3859 | 1316.33008 | 42.43214 | 16.7362 |
| 7 | 1.874 | VV | 0.2010 | 590.77185 | 38.07349 | 7.5113 |
| 8 | 2.257 | VB | 0.4231 | 1251.55310 | 36.95633 | 15.9126 |
| 9 | 3.431 | BV | 0.3762 | 601.88397 | 19.71378 | 7.6525 |
| 10 | 3.865 | VV | 0.3745 | 446.19189 | 14.68248 | 5.6730 |
| 11 | 4.305 | VB | 0.4431 | 370.36951 | 10.93686 | 4.7090 |
| 12 | 6.601 | BV | 0.2937 | 10.74699 | 4.79126e-1 | 0.1366 |
| 13 | 16.146 | BV | 0.1549 | 2.62627 | 2.34158e-1 | 0.0334 |

Totals : 7865.15391 510.04718

Signal 2: DAD1 B, Sig=280,16 Ref=360,100

| Peak # | RetTime [min] | Type | Width [min] | Area [mAU*s] | Height [mAU] | Area % |
|---|---|---|---|---|---|---|
| 1 | 0.438 | BV | 0.0667 | 78.17081 | 18.72053 | 34.4398 |
| 2 | 0.647 | VV | 0.1033 | 34.97692 | 4.78637 | 15.4098 |
| 3 | 0.839 | VV | 0.1756 | 33.87909 | 2.54083 | 14.9261 |
| 4 | 1.538 | VB | 0.5740 | 56.81307 | 1.20950 | 25.0302 |
| 5 | 2.256 | BB | 0.2398 | 13.68399 | 7.60974e-1 | 6.0288 |
| 6 | 4.298 | BB | 0.2577 | 9.45454 | 5.36265e-1 | 4.1654 |

Totals : 226.97843 28.55448

Signal 3: DAD1 C, Sig=282,8 Ref=360,100

| Peak # | RetTime [min] | Type | Width [min] | Area [mAU*s] | Height [mAU] | Area % |
|---|---|---|---|---|---|---|
| 1 | 0.438 | BV | 0.0667 | 78.34995 | 18.76041 | 35.1988 |
| 2 | 0.648 | VV | 0.1032 | 34.90321 | 4.78043 | 15.6803 |
| 3 | 0.839 | VV | 0.1698 | 32.01859 | 2.49387 | 14.3844 |
| 4 | 1.534 | VB | 0.6210 | 58.89091 | 1.15854 | 26.4568 |
| 5 | 2.254 | BB | 0.2165 | 10.70894 | 6.69914e-1 | 4.8110 |
| 6 | 4.296 | BB | 0.2348 | 7.72084 | 5.15315e-1 | 3.4686 |

Totals : 222.59243 28.37848

Signal 4: DAD1 D, Sig=254,16 Ref=360,100

| Peak # | RetTime [min] | Type | Width [min] | Area [mAU*s] | Height [mAU] | Area % |
|---|---|---|---|---|---|---|
| 1 | 0.437 | BV | 0.0807 | 127.76701 | 23.77365 | 16.1051 |
| 2 | 0.648 | VV | 0.1164 | 67.94011 | 8.05783 | 8.5639 |
| 3 | 0.839 | VV | 0.1376 | 43.95439 | 4.36228 | 5.5405 |
| 4 | 1.035 | VV | 0.2010 | 64.51491 | 4.15894 | 8.1322 |
| 5 | 1.305 | VV | 0.1422 | 34.70547 | 3.48527 | 4.3747 |
| 6 | 1.525 | VB | 0.3609 | 97.14166 | 3.40413 | 12.2448 |
| 7 | 2.257 | BB | 0.5336 | 140.94847 | 3.26533 | 17.7667 |
| 8 | 3.815 | BV | 0.3738 | 58.41641 | 2.05408 | 7.3634 |
| 9 | 4.308 | VB | 0.4819 | 129.91272 | 3.44551 | 16.3756 |
| 10 | 5.835 | BB | 0.4668 | 28.02955 | 7.77112e-1 | 3.5331 |

Totals : 793.33069 56.78414

=====================================================================

*** End of Report ***
