# Supplementary material for: Influence of Silver Nanoparticles (AgNPs) on Vegetative Growth and Concentrations of Nutrients and Phytohormones in Tomato
Source: Plants (Basel). 2026 Jan 28;15(3):405. doi: 10.3390/plants15030405 (PMC12899181; doi:10.3390/plants15030405)
Supplement: Supplementary file 1 [file plants-15-00405-s001.zip › S1. HPLC Analysis (plants-4015186)/cv. Rio Grande/Roots/5 ppm/RG-5-R-R1.pdf]

===

Acq. Operator : TMG   Seq. Line : 31
Acq. Instrument : Instrument 1   Location : Vial 31
Injection Date : 10/4/2012 1:38:52 AM   Inj : 1
Inj Volume : 200.0 µl
Different Inj Volume from Sequence !   Actual Inj Volume : 50.0 µl
Acq. Method : C:\CHEM32\1\DATA\FITOHORMTMG\FITOHOR GABY Y ALE 30-11-2020 2012-10-03 09-08-53\FITOHORMONAS DR SOTO.M
Last changed : 8/14/2013 11:13:25 AM by TMG
Analysis Method : C:\CHEM32\1\METHODS\LAVADO COLUMNA ACET.M
Last changed : 10/21/2012 12:24:49 PM by TMG
(modified after loading)

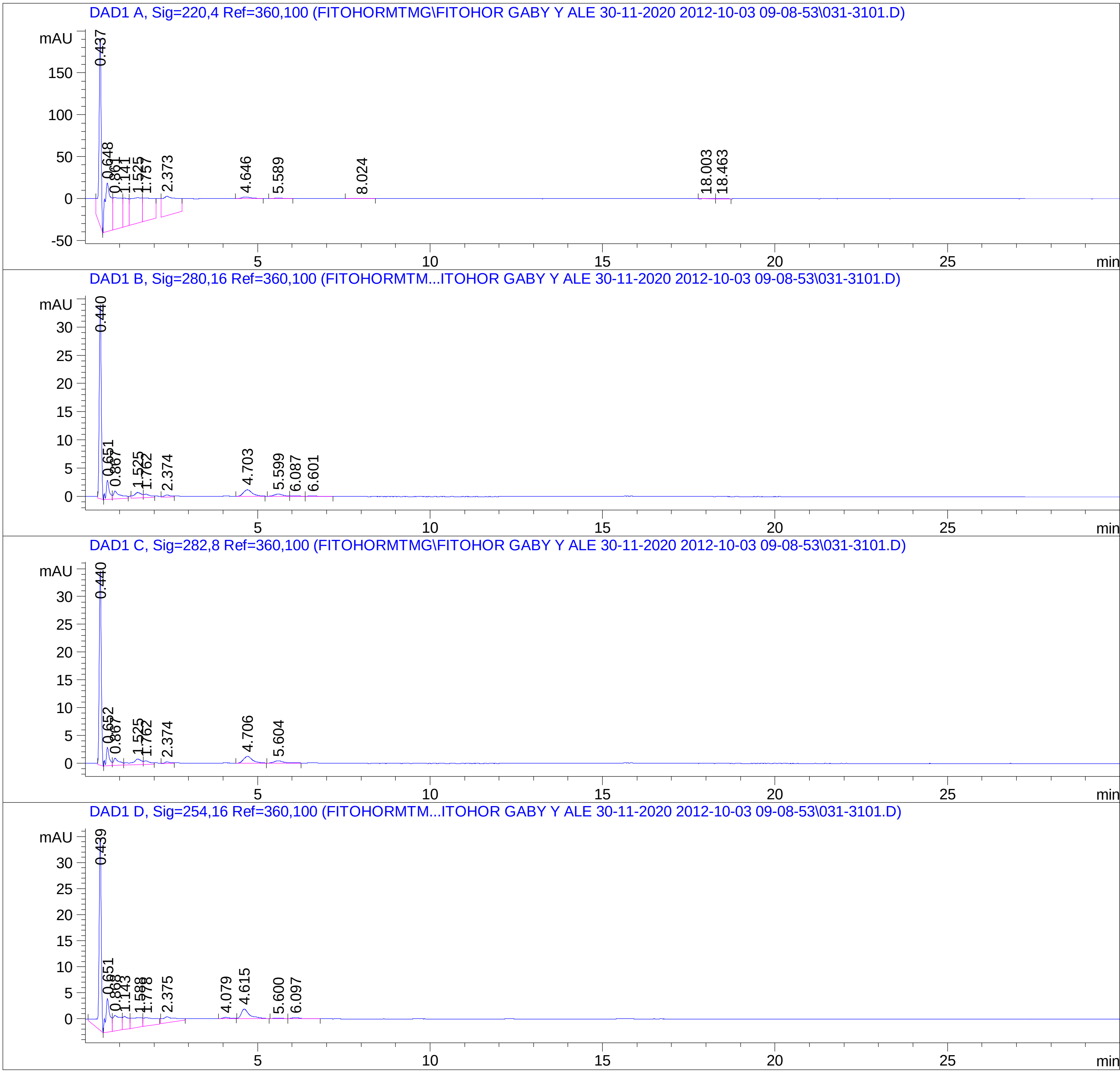

===

Area Percent Report

===

Sorted By : Signal
Multiplier: : 1.0000
Dilution: : 1.0000
Use Multiplier & Dilution Factor with ISTDs

Signal 1: DAD1 A, Sig=220,4 Ref=360,100

Peak #	RetTime [min]	Type	Width [min]	Area [mAU*s]	Height [mAU]	Area %
1	0.437	BV	0.0678	979.38068	220.51236	20.5117
2	0.648	VV	0.1593	688.72211	57.67894	14.4243
3	0.861	VV	0.2128	641.00415	37.99413	13.4249
4	1.141	VV	0.1668	382.97321	34.15185	8.0208
5	1.525	VV	0.3143	694.30542	30.41311	14.5412
6	1.757	VB	0.2890	619.65967	27.70004	12.9779
7	2.373	BB	0.3945	708.84625	22.81456	14.8458
8	4.646	BB	0.2737	33.10182	1.80682	0.6933
9	5.589	BB	0.2567	10.46917	6.20988e-1	0.2193
10	8.024	BV	0.3291	8.66441	3.31339e-1	0.1815
11	18.003	BB	0.1622	2.66208	2.54124e-1	0.0558
12	18.463	BV	0.2673	4.95016	2.37389e-1	0.1037

Totals : 4774.73912 434.51565

Signal 2: DAD1 B, Sig=280,16 Ref=360,100

Peak #	RetTime [min]	Type	Width [min]	Area [mAU*s]	Height [mAU]	Area %
1	0.440	BV	0.0654	139.60075	34.35443	58.0143
2	0.651	VV	0.0952	21.31119	3.30495	8.8563
3	0.867	VB	0.1820	19.49910	1.40408	8.1033
4	1.525	BV	0.2153	14.67441	9.87891e-1	6.0983
5	1.762	VB	0.1931	8.86927	6.26558e-1	3.6858
6	2.374	BB	0.1931	4.79381	3.47215e-1	1.9922
7	4.703	BB	0.2721	20.66795	1.15887	8.5890
8	5.599	BV	0.2688	7.48008	3.98835e-1	3.1085
9	6.087	VV	0.2208	1.96053	1.18643e-1	0.8147
10	6.601	VB	0.2672	1.77472	8.11389e-2	0.7375

Totals : 240.63180 42.78261

Signal 3: DAD1 C, Sig=282,8 Ref=360,100

Peak #	RetTime [min]	Type	Width [min]	Area [mAU*s]	Height [mAU]	Area %
1	0.440	BV	0.0654	141.88055	34.92551	59.0296
2	0.652	VV	0.0941	20.68505	3.25886	8.6060
3	0.867	VV	0.1514	15.03280	1.33491	6.2544
4	1.525	VV	0.2651	18.92999	9.98790e-1	7.8759
5	1.762	VB	0.1815	8.33267	6.25004e-1	3.4668
6	2.374	BB	0.1858	4.16684	3.16174e-1	1.7336
7	4.706	BB	0.2676	21.77532	1.22399	9.0597
8	5.604	BB	0.3089	9.55159	4.33845e-1	3.9740

Totals : 240.35481 43.11708

Signal 4: DAD1 D, Sig=254,16 Ref=360,100

Peak #	RetTime [min]	Type	Width [min]	Area [mAU*s]	Height [mAU]	Area %
1	0.439	BV	0.0699	171.20609	37.05239	37.6847
2	0.651	VV	0.1185	54.43500	6.44882	11.9818
3	0.868	VV	0.1945	44.67802	2.98656	9.8342
4	1.143	VV	0.1628	30.61721	2.50122	6.7392
5	1.588	VV	0.2850	41.27580	1.81719	9.0853
6	1.778	VB	0.3127	39.13593	1.59234	8.6143
7	2.375	BB	0.3402	31.02078	1.16663	6.8281
8	4.079	BB	0.2077	3.62120	2.46032e-1	0.7971
9	4.615	BB	0.2430	31.25212	1.85104	6.8790
10	5.600	BV	0.2561	2.62486	1.40423e-1	0.5778
11	6.097	VB	0.2726	4.44525	2.35079e-1	0.9785

Totals : 454.31226 56.03771

==

*** End of Report ***
