# Supplementary material for: Influence of Silver Nanoparticles (AgNPs) on Vegetative Growth and Concentrations of Nutrients and Phytohormones in Tomato
Source: Plants (Basel). 2026 Jan 28;15(3):405. doi: 10.3390/plants15030405 (PMC12899181; doi:10.3390/plants15030405)
Supplement: Supplementary file 1 [file plants-15-00405-s001.zip › S1. HPLC Analysis (plants-4015186)/cv. Rio Grande/Roots/5 ppm/RG-5-R-R2.pdf]

===

Acq. Operator : TMG   Seq. Line : 32
Acq. Instrument : Instrument 1   Location : Vial 32
Injection Date : 10/4/2012 2:11:00 AM   Inj : 1
Inj Volume : 200.0 µl
Different Inj Volume from Sequence !   Actual Inj Volume : 50.0 µl
Acq. Method : C:\CHEM32\1\DATA\FITOHORMTMG\FITOHOR GABY Y ALE 30-11-2020 2012-10-03 09-08-53\FITOHORMONAS DR SOTO.M
Last changed : 8/14/2013 11:13:25 AM by TMG
Analysis Method : C:\CHEM32\1\METHODS\LAVADO COLUMNA ACET.M
Last changed : 10/21/2012 12:24:49 PM by TMG
(modified after loading)

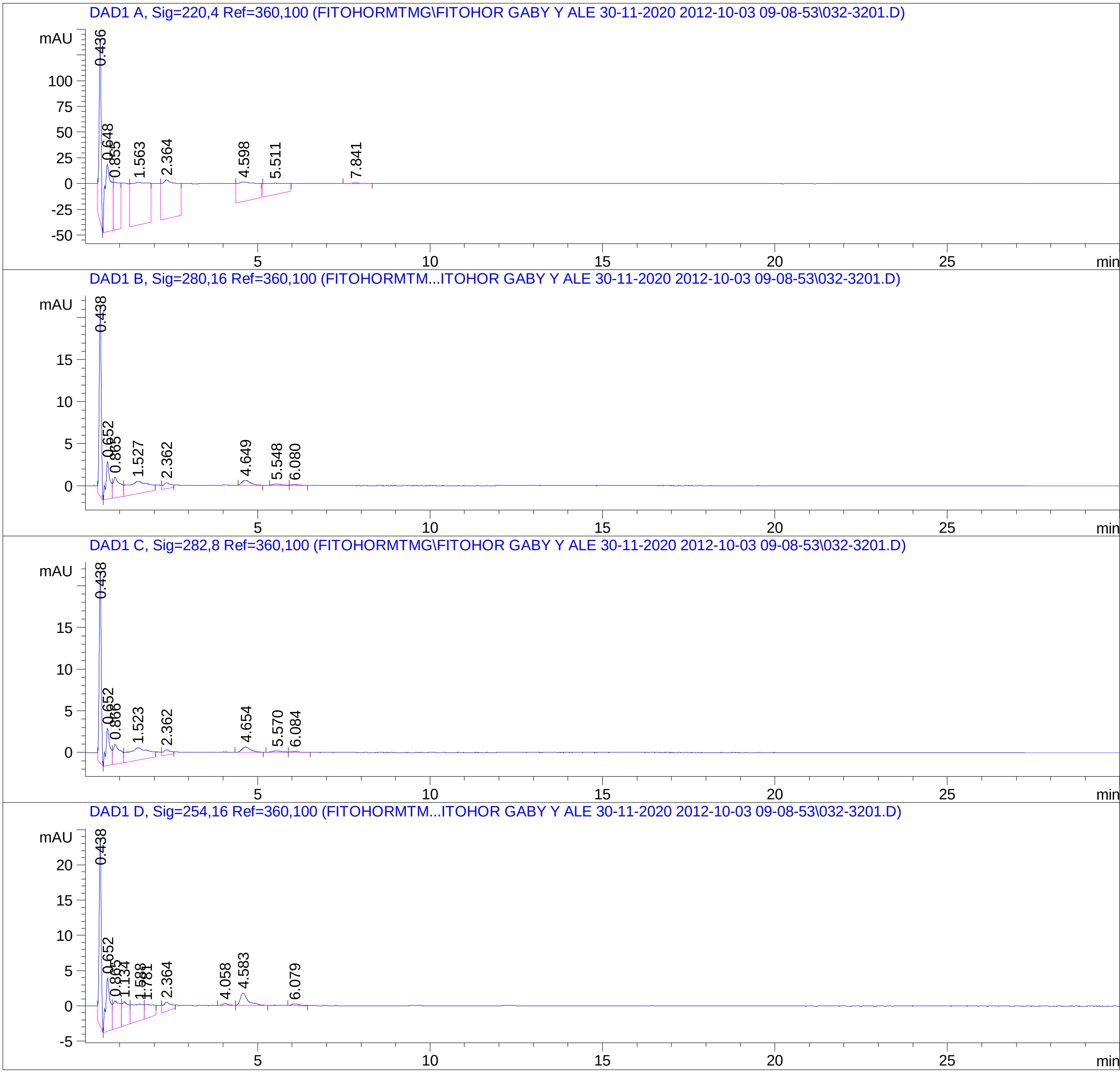

===

Area Percent Report

===

Sorted By : Signal
Multiplier: : 1.0000
Dilution: : 1.0000
Use Multiplier & Dilution Factor with ISTDs

Signal 1: DAD1 A, Sig=220,4 Ref=360,100

Peak #	RetTime [min]	Type	Width [min]	Area [mAU*s]	Height [mAU]	Area %
1	0.436	BV	0.0656	746.83209	175.83295	12.0426
2	0.648	VV	0.1681	833.51746	65.64864	13.4404
3	0.855	VB	0.1771	642.46918	46.53542	10.3597
4	1.563	BB	0.4776	1509.42114	40.99038	24.3393
5	2.364	BB	0.4020	1204.12195	37.56477	19.4163
6	4.598	BB	0.5118	747.61420	18.97117	12.0552
7	5.511	BV	0.5791	510.06009	10.95758	8.2247
8	7.841	BB	0.2957	7.55582	3.10713e-1	0.1218

Totals : 6201.59193 396.81161

Signal 2: DAD1 B, Sig=280,16 Ref=360,100

Peak #	RetTime [min]	Type	Width [min]	Area [mAU*s]	Height [mAU]	Area %
1	0.438	BV	0.0668	95.45708	22.81279	37.0730
2	0.652	VV	0.1177	37.52787	4.48293	14.5748
3	0.865	VV	0.1872	35.61630	2.48573	13.8324
4	1.527	VB	0.5449	62.97338	1.48467	24.4572
5	2.362	BB	0.2033	10.09185	6.71744e-1	3.9194
6	4.649	BB	0.2721	11.10530	6.22552e-1	4.3130
7	5.548	BB	0.2439	2.98609	1.67582e-1	1.1597
8	6.080	BB	0.2030	1.72613	1.13753e-1	0.6704

Totals : 257.48400 32.84175

Signal 3: DAD1 C, Sig=282,8 Ref=360,100

Peak #	RetTime [min]	Type	Width [min]	Area [mAU*s]	Height [mAU]	Area %
1	0.438	BV	0.0668	96.15760	22.99301	37.9095
2	0.652	VV	0.1173	37.14542	4.45390	14.6443

Peak #	RetTime [min]	Type	Width [min]	Area [mAU*s]	Height [mAU]	Area %
3	0.866	VV	0.1863	34.54059	2.42303	13.6174
4	1.523	VB	0.5307	60.69697	1.47309	23.9294
5	2.362	BB	0.1995	8.94291	6.08525e-1	3.5257
6	4.654	BB	0.2680	11.15602	6.37864e-1	4.3982
7	5.570	BV	0.2454	3.32197	1.88714e-1	1.3097
8	6.084	VB	0.2202	1.68906	1.00445e-1	0.6659
Totals :				253.65055	32.87858	

Signal 4: DAD1 D, Sig=254,16 Ref=360,100

Peak #	RetTime [min]	Type	Width [min]	Area [mAU*s]	Height [mAU]	Area %
1	0.438	BV	0.0677	114.16341	26.79504	26.0709
2	0.652	VV	0.1283	69.88915	7.52669	15.9602
3	0.865	VV	0.1826	55.39669	3.97509	12.6507
4	1.134	VV	0.1808	46.54997	3.37723	10.6304
5	1.588	VV	0.3227	57.65759	2.28265	13.1670
6	1.781	VB	0.2325	35.35865	1.92283	8.0747
7	2.364	BB	0.2304	21.91310	1.27578	5.0042
8	4.058	BV	0.2054	3.70021	2.67426e-1	0.8450
9	4.583	VB	0.2354	29.25903	1.80200	6.6817
10	6.079	BB	0.2280	4.00751	2.56905e-1	0.9152
Totals :				437.89533	49.48164	

==

*** End of Report ***
