# Supplementary material for: Influence of Silver Nanoparticles (AgNPs) on Vegetative Growth and Concentrations of Nutrients and Phytohormones in Tomato
Source: Plants (Basel). 2026 Jan 28;15(3):405. doi: 10.3390/plants15030405 (PMC12899181; doi:10.3390/plants15030405)
Supplement: Supplementary file 1 [file plants-15-00405-s001.zip › S1. HPLC Analysis (plants-4015186)/cv. Rio Grande/Roots/5 ppm/RG-5-R-R3.pdf]

Data File C:\CHEM32\...ORMTMG\FITOHOR GABY Y ALE 30-11-2020 2012-10-03 09-08-53\033-3301.D
Sample Name: 5 PPM RIO GRANDE RAIZ R3

===

Acq. Operator : TMG Seq. Line : 33
Acq. Instrument : Instrument 1 Location : Vial 33
Injection Date : 10/4/2012 2:43:07 AM Inj : 1
Inj Volume : 200.0 µl
Different Inj Volume from Sequence ! Actual Inj Volume : 50.0 µl
Acq. Method : C:\CHEM32\1\DATA\FITOHORMTMG\FITOHOR GABY Y ALE 30-11-2020 2012-10-03 09-08-53\FITOHORMONAS DR SOTO.M
Last changed : 8/14/2013 11:13:25 AM by TMG
Analysis Method : C:\CHEM32\1\METHODS\LAVADO COLUMNA ACET.M
Last changed : 10/21/2012 12:24:49 PM by TMG
(modified after loading)

DAD1 A, Sig=220,4 Ref=360,100 (FITOHORMTMG\FITOHOR GABY Y ALE 30-11-2020 2012-10-03 09-08-53\033-3301.D)

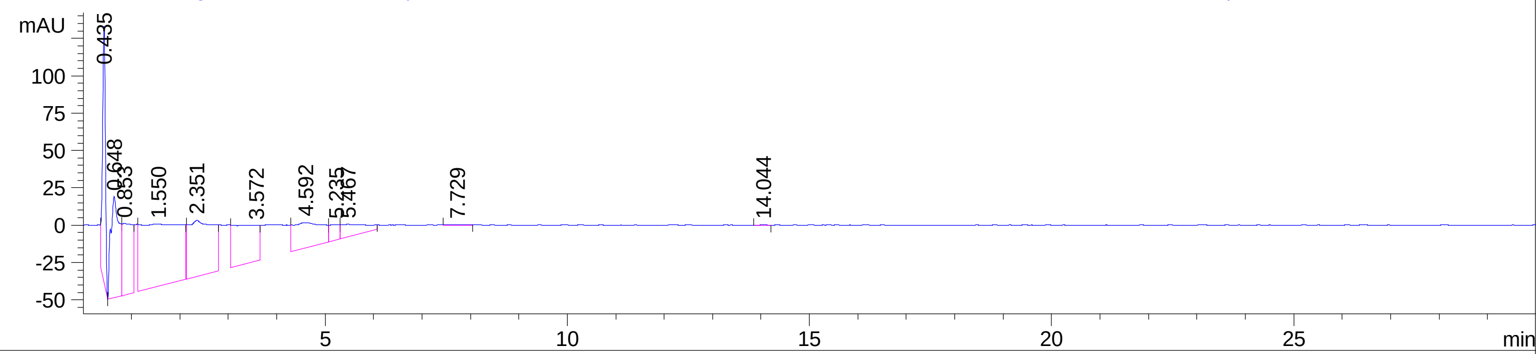

DAD1 B, Sig=280,16 Ref=360,100 (FITOHORMTM...ITOHOR GABY Y ALE 30-11-2020 2012-10-03 09-08-53\033-3301.D)

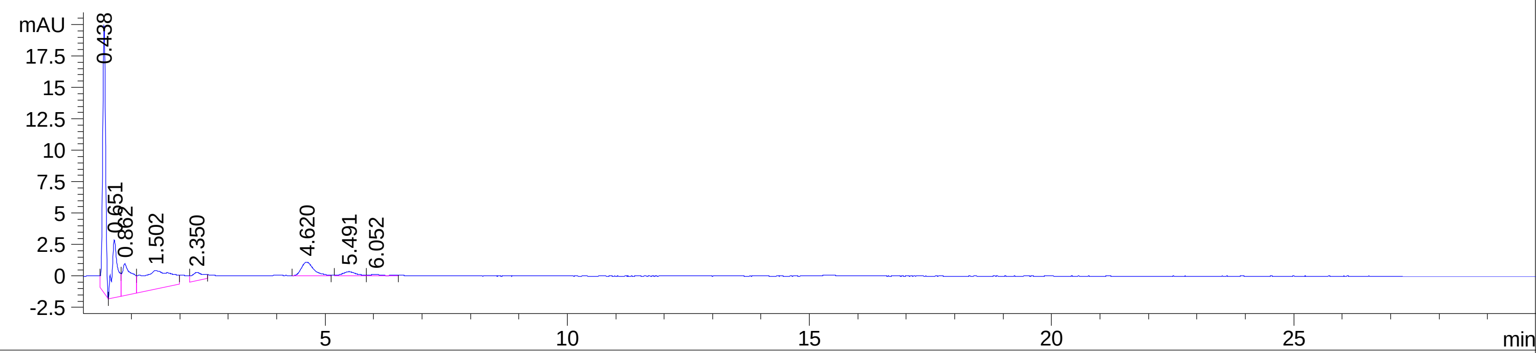

DAD1 C, Sig=282,8 Ref=360,100 (FITOHORMTMG\FITOHOR GABY Y ALE 30-11-2020 2012-10-03 09-08-53\033-3301.D)

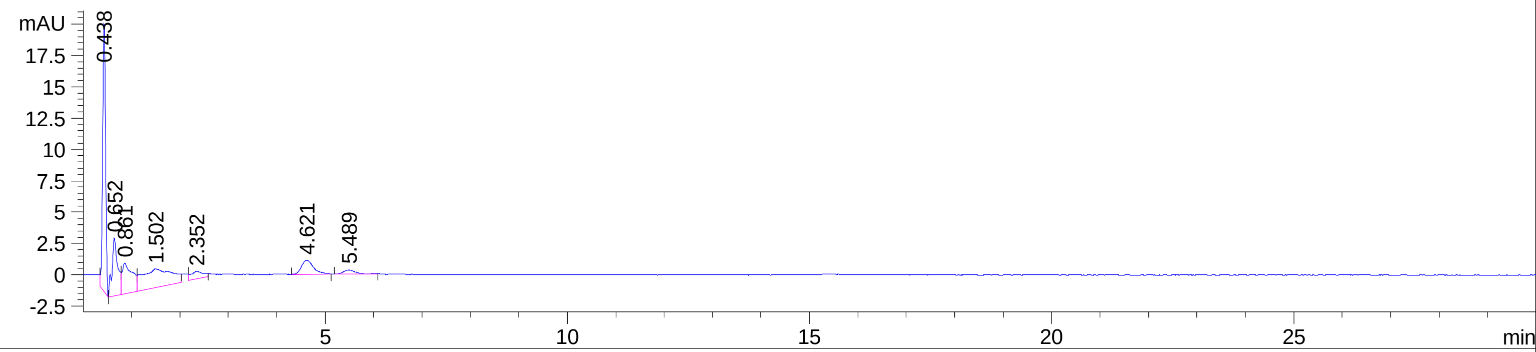

DAD1 D, Sig=254,16 Ref=360,100 (FITOHORMTM...ITOHOR GABY Y ALE 30-11-2020 2012-10-03 09-08-53\033-3301.D)

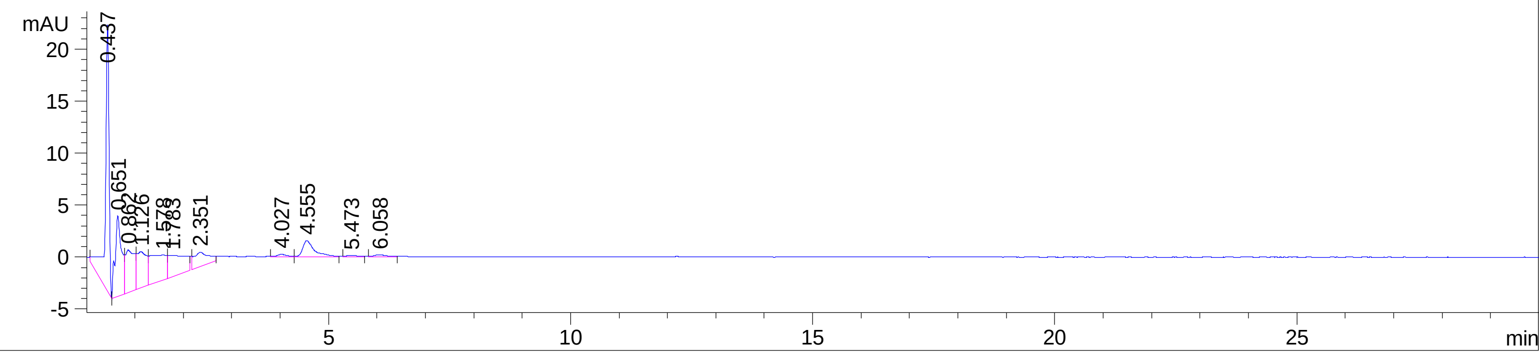

===

Area Percent Report

===

Sorted By : Signal
Multiplier: : 1.0000
Dilution: : 1.0000
Use Multiplier & Dilution Factor with ISTDs

Signal 1: DAD1 A, Sig=220,4 Ref=360,100

Peak #	RetTime [min]	Type	Width [min]	Area [mAU*s]	Height [mAU]	Area %
1	0.435	BV	0.0658	723.84869	169.70918	8.8866
2	0.648	VV	0.1652	841.37476	67.58346	10.3294
3	0.853	VB	0.1955	721.09082	47.94374	8.8527
4	1.550	BB	0.6982	2403.31812	41.58564	29.5051
5	2.351	BB	0.4533	1349.29944	37.36095	16.5651
6	3.572	BV	0.4871	948.73761	23.89997	11.6475
7	4.592	BV	0.5649	720.40356	17.05081	8.8443
8	5.235	VV	0.2118	148.23451	10.06706	1.8198
9	5.467	VB	0.4312	281.82474	8.40449	3.4599
10	7.729	BB	0.2320	4.86497	2.83829e-1	0.0597
11	14.044	BV	0.1683	2.43317	2.28395e-1	0.0299

Totals : 8145.43039 424.11753

Signal 2: DAD1 B, Sig=280,16 Ref=360,100

Peak #	RetTime [min]	Type	Width [min]	Area [mAU*s]	Height [mAU]	Area %
1	0.438	BV	0.0673	90.09756	21.33844	34.3594
2	0.651	VV	0.1139	37.89323	4.60898	14.4509
3	0.862	VV	0.1872	35.92603	2.50607	13.7007
4	1.502	VB	0.5206	61.91356	1.47236	23.6112
5	2.350	BB	0.2194	10.54589	6.56567e-1	4.0218
6	4.620	BB	0.2617	18.33239	1.06039	6.9912
7	5.491	BV	0.2578	5.68540	3.22334e-1	2.1682
8	6.052	VB	0.2755	1.82659	8.40773e-2	0.6966

Totals : 262.22066 32.04922

Signal 3: DAD1 C, Sig=282,8 Ref=360,100

Peak #	RetTime [min]	Type	Width [min]	Area [mAU*s]	Height [mAU]	Area %
1	0.438	BV	0.0672	90.40224	21.42709	34.5875
2	0.652	VV	0.1137	37.69145	4.59387	14.4206
3	0.861	VV	0.1872	35.16792	2.45403	13.4551
4	1.502	VB	0.5277	61.91241	1.46887	23.6874
5	2.352	BB	0.2394	10.62206	6.03433e-1	4.0640
6	4.621	BB	0.2633	19.28131	1.11743	7.3769
7	5.489	BB	0.2661	6.29499	3.49484e-1	2.4084
Totals :				261.37238	32.01421	

Signal 4: DAD1 D, Sig=254,16 Ref=360,100

Peak #	RetTime [min]	Type	Width [min]	Area [mAU*s]	Height [mAU]	Area %
1	0.437	BV	0.0811	139.11638	25.73283	28.6943
2	0.651	VV	0.1259	71.88742	7.77425	14.8276
3	0.862	VV	0.1699	52.80374	4.11008	10.8913
4	1.126	VV	0.1872	50.44596	3.52095	10.4050
5	1.578	VV	0.3137	59.69811	2.40398	12.3134
6	1.783	VB	0.2999	49.02273	2.04338	10.1115
7	2.351	BB	0.2832	29.53473	1.38281	6.0919
8	4.027	BV	0.2011	2.71849	2.01889e-1	0.5607
9	4.555	VB	0.2399	25.11199	1.52688	5.1796
10	5.473	BB	0.2176	1.69003	1.04007e-1	0.3486
11	6.058	BB	0.2273	2.79326	1.81717e-1	0.5761
Totals :				484.82284	48.98279	

===

*** End of Report ***
