# Supplementary material for: Influence of Silver Nanoparticles (AgNPs) on Vegetative Growth and Concentrations of Nutrients and Phytohormones in Tomato
Source: Plants (Basel). 2026 Jan 28;15(3):405. doi: 10.3390/plants15030405 (PMC12899181; doi:10.3390/plants15030405)
Supplement: Supplementary file 1 [file plants-15-00405-s001.zip › S1. HPLC Analysis (plants-4015186)/cv. Rio Grande/Roots/Control/RG-T-R-R1.pdf]

Data File C:\CHEM32\...ORMTMG\FITOHOR GABY Y ALE 30-11-2020 2012-10-03 09-08-53\019-1901.D
Sample Name: TESTIGO RIO GRANDE RAIZ R1

===

Acq. Operator	: TMG	Seq. Line :	19
Acq. Instrument	: Instrument 1	Location :	Vial 19
Injection Date	: 10/3/2012 7:13:29 PM	Inj :	1
		Inj Volume :	200.0 µl
Different Inj Volume from Sequence !		Actual Inj Volume :	50.0 µl

Acq. Method : C:\CHEM32\1\DATA\FITOHORMTMG\FITOHOR GABY Y ALE 30-11-2020 2012-10-03 09-08-53\FITOHORMONAS DR SOTO.M
Last changed : 8/14/2013 11:13:25 AM by TMG
Analysis Method : C:\CHEM32\1\METHODS\LAVADO COLUMNA ACET.M
Last changed : 10/21/2012 12:24:49 PM by TMG
(modified after loading)

DAD1 A, Sig=220,4 Ref=360,100 (FITOHORMTMG\FITOHOR GABY Y ALE 30-11-2020 2012-10-03 09-08-53\019-1901.D)

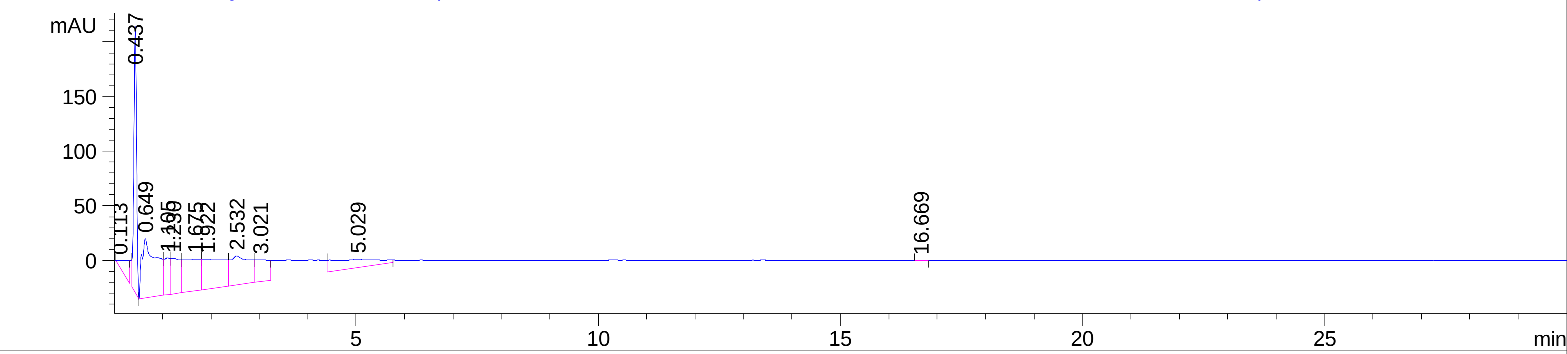

DAD1 B, Sig=280,16 Ref=360,100 (FITOHORMTM...ITOHOR GABY Y ALE 30-11-2020 2012-10-03 09-08-53\019-1901.D)

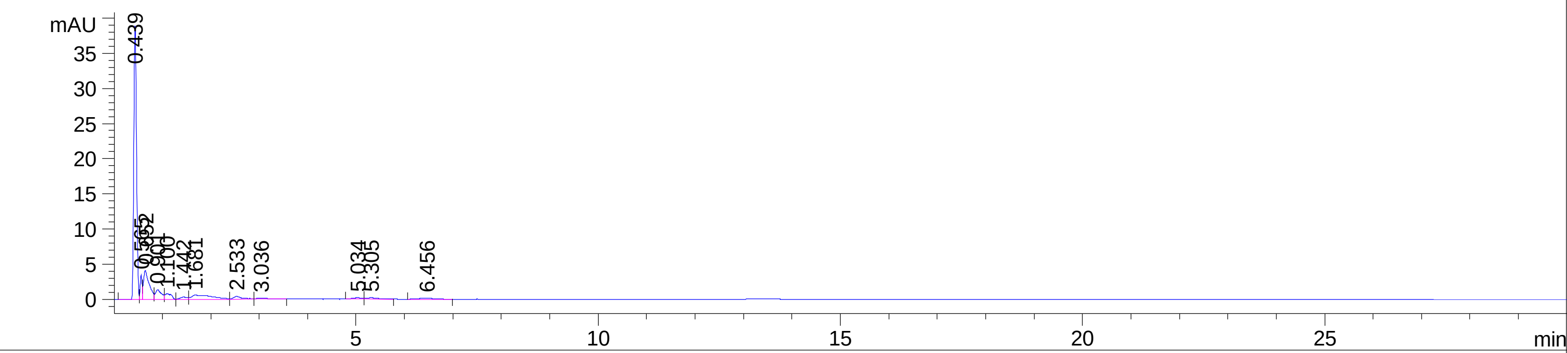

DAD1 C, Sig=282,8 Ref=360,100 (FITOHORMTMG\FITOHOR GABY Y ALE 30-11-2020 2012-10-03 09-08-53\019-1901.D)

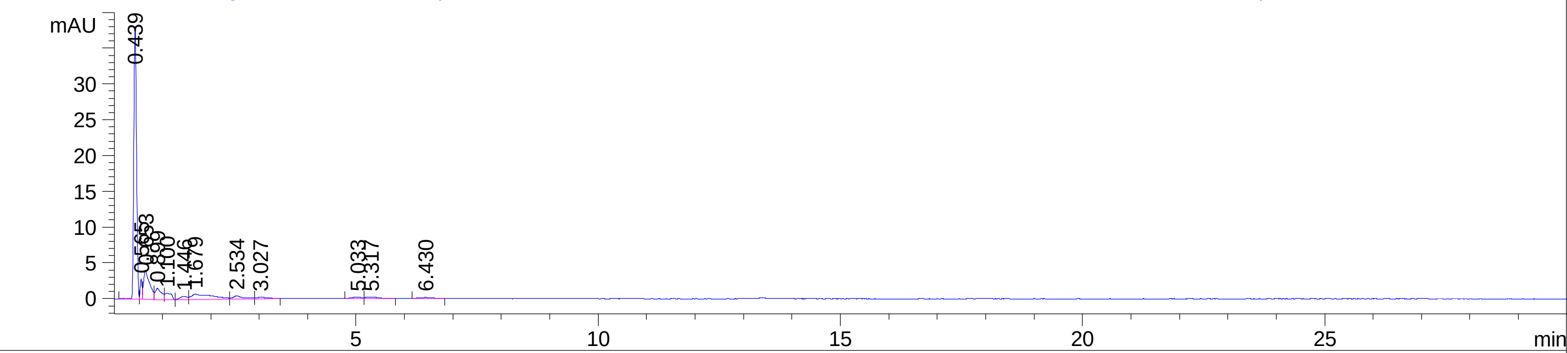

DAD1 D, Sig=254,16 Ref=360,100 (FITOHORMTM...ITOHOR GABY Y ALE 30-11-2020 2012-10-03 09-08-53\019-1901.D)

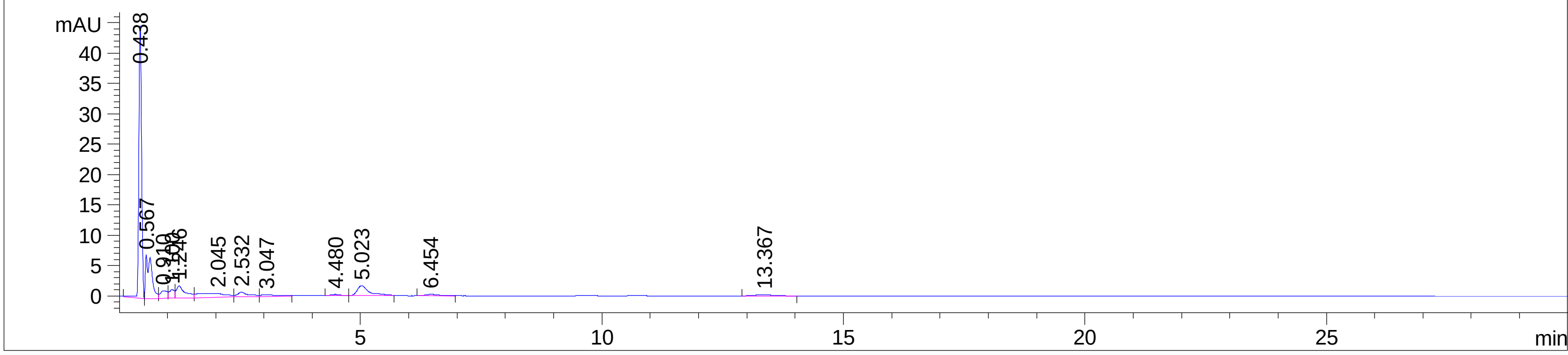

===

Area Percent Report

===

Sorted By : Signal
Multiplier: : 1.0000
Dilution: : 1.0000
Use Multiplier & Dilution Factor with ISTDs

Signal 1: DAD1 A, Sig=220,4 Ref=360,100

Peak #	RetTime [min]	Type	Width [min]	Area [mAU*s]	Height [mAU]	Area %
1	0.113	BB	0.3631	172.56870	6.00805	2.7559
2	0.437	BV	0.0622	957.49005	241.60899	15.2907
3	0.649	VV	0.2619	1121.20471	54.06785	17.9052
4	1.105	VV	0.1274	309.55869	33.62300	4.9435
5	1.230	VV	0.1709	410.44312	32.59042	6.5546
6	1.675	VV	0.3175	728.31525	28.95314	11.6309
7	1.922	VV	0.4053	871.88544	27.24639	13.9237
8	2.532	VV	0.3610	737.67670	26.48194	11.7804
9	3.021	VV	0.2676	393.90024	19.84126	6.2904
10	5.029	BB	0.8748	556.73456	7.85579	8.8908
11	16.669	BV	0.1273	2.12373	2.49624e-1	0.0339

Totals : 6261.90117 478.52647

Signal 2: DAD1 B, Sig=280,16 Ref=360,100

Peak #	RetTime [min]	Type	Width [min]	Area [mAU*s]	Height [mAU]	Area %
1	0.439	BV	0.0641	154.26749	39.02410	60.2791
2	0.565	VV	0.0406	9.66681	3.45580	3.7772
3	0.652	VV	0.1121	33.35256	4.13405	13.0323
4	0.901	VV	0.1206	12.10647	1.40404	4.7305
5	1.100	VV	0.1250	7.65491	8.19672e-1	2.9911
6	1.442	VV	0.1508	3.12975	3.12709e-1	1.2229
7	1.681	VV	0.3699	17.94592	6.16019e-1	7.0123
8	2.533	VV	0.1907	5.14808	3.88205e-1	2.0116
9	3.036	VB	0.2285	2.84499	1.76095e-1	1.1117
10	5.034	BV	0.1918	2.68088	1.84178e-1	1.0475
11	5.305	VB	0.2706	3.47883	1.83958e-1	1.3593
12	6.456	BB	0.2974	3.64548	1.59025e-1	1.4244

Totals : 255.92215 50.85786

Signal 3: DAD1 C, Sig=282,8 Ref=360,100

Peak #	RetTime [min]	Type	Width [min]	Area [mAU*s]	Height [mAU]	Area %
1	0.439	BV	0.0641	151.32979	38.25921	57.1779
2	0.565	VV	0.0390	7.55761	2.84137	2.8555
3	0.653	VV	0.1197	34.93896	4.00970	13.2012
4	0.899	VV	0.1217	13.82610	1.55482	5.2240
5	1.100	VV	0.1284	8.93788	9.27370e-1	3.3771
6	1.446	VV	0.1626	4.80753	4.42946e-1	1.8165
7	1.679	VV	0.3950	23.68303	7.52789e-1	8.9483
8	2.534	VV	0.2211	6.85163	4.31923e-1	2.5888
9	3.027	VB	0.2164	3.46666	2.03802e-1	1.3098
10	5.033	BV	0.1960	2.47733	1.68013e-1	0.9360
11	5.317	VB	0.2497	3.66457	2.05812e-1	1.3846
12	6.430	BB	0.2738	3.12393	1.51994e-1	1.1803

Totals : 264.66499 49.94975

Signal 4: DAD1 D, Sig=254,16 Ref=360,100

Peak #	RetTime [min]	Type	Width [min]	Area [mAU*s]	Height [mAU]	Area %
1	0.438	BV	0.0645	179.17831	44.95750	47.0986
2	0.567	VV	0.1120	62.45766	7.14104	16.4176
3	0.910	VV	0.1370	12.84972	1.25972	3.3777
4	1.100	VV	0.1004	10.23968	1.44901	2.6916
5	1.246	VV	0.1770	26.67616	2.03349	7.0121
6	2.045	VV	0.5135	27.28940	6.55769e-1	7.1733
7	2.532	VV	0.2296	12.99691	7.83361e-1	3.4164
8	3.047	VB	0.3001	6.37954	2.92864e-1	1.6769
9	4.480	BV	0.1971	2.62662	1.90194e-1	0.6904
10	5.023	VB	0.2602	29.30295	1.64182	7.7025
11	6.454	BB	0.2653	4.44775	2.28336e-1	1.1691
12	13.367	BB	0.4167	5.98738	1.73466e-1	1.5738

Totals : 380.43207 60.80657

===

*** End of Report ***
