# Supplementary material for: Influence of Silver Nanoparticles (AgNPs) on Vegetative Growth and Concentrations of Nutrients and Phytohormones in Tomato
Source: Plants (Basel). 2026 Jan 28;15(3):405. doi: 10.3390/plants15030405 (PMC12899181; doi:10.3390/plants15030405)
Supplement: Supplementary file 1 [file plants-15-00405-s001.zip › S1. HPLC Analysis (plants-4015186)/cv. Rio Grande/Roots/Control/RG-T-R-R2.pdf]

Data File C:\CHEM32\...ORMTMG\FITOHOR GABY Y ALE 30-11-2020 2012-10-03 09-08-53\020-2001.D
Sample Name: TESTIGO RIO GRANDE RAIZ R2

===

```
Acq. Operator   : TMG                              Seq. Line :  20
Acq. Instrument : Instrument 1                      Location : Vial 20
Injection Date  : 10/3/2012 7:45:37 PM                   Inj :   1
                                                  Inj Volume : 200.0 µl
Different Inj Volume from Sequence !       Actual Inj Volume : 50.0 µl
Acq. Method     : C:\CHEM32\1\DATA\FITOHORMTMG\FITOHOR GABY Y ALE 30-11-2020 2012-10-03 09-08-
                  53\FITOHORMONAS DR SOTO.M
Last changed    : 8/14/2013 11:13:25 AM by TMG
Analysis Method : C:\CHEM32\1\METHODS\LAVADO COLUMNA ACET.M
Last changed    : 10/21/2012 12:24:49 PM by TMG
                  (modified after loading)
```

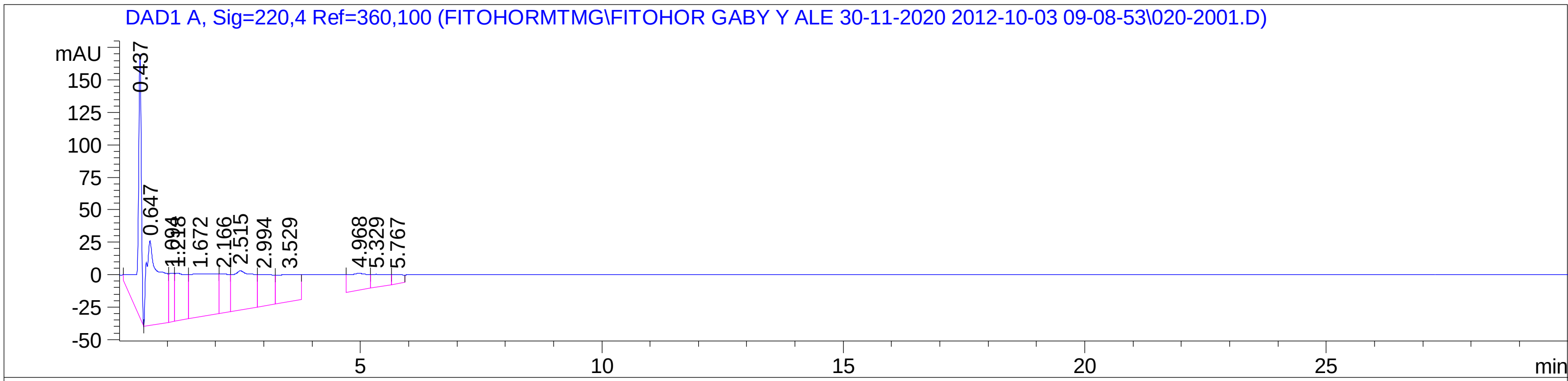

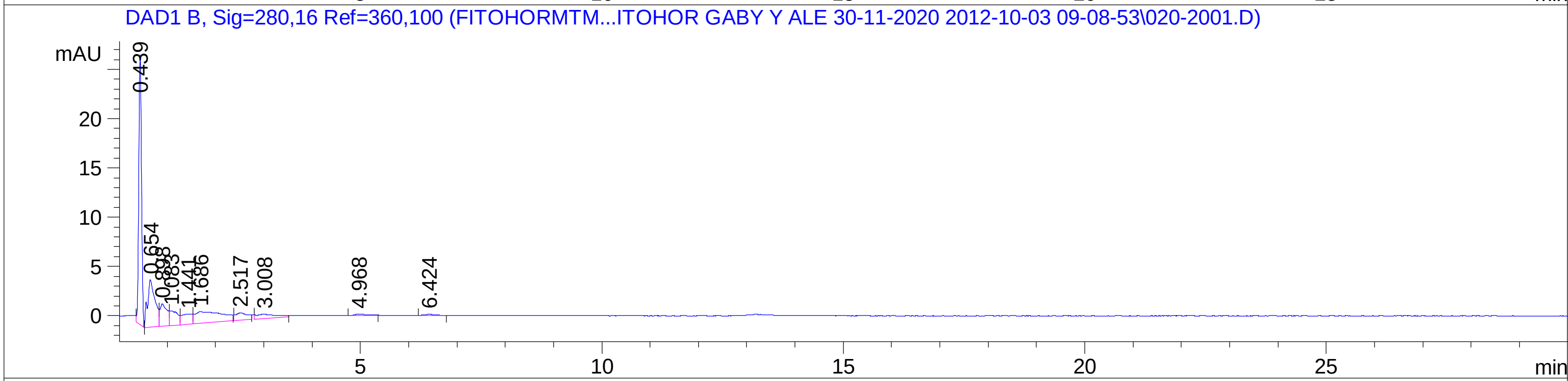

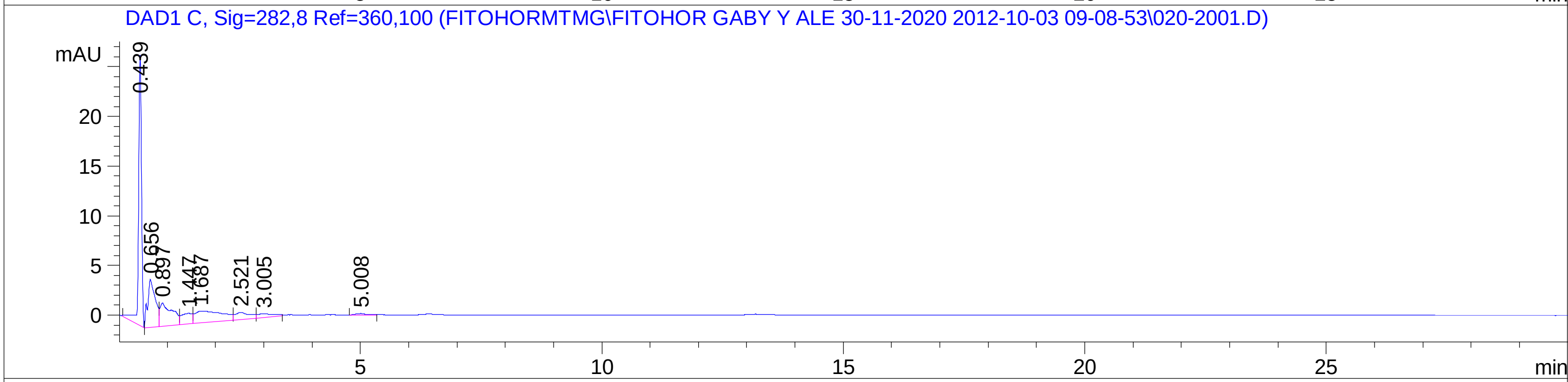

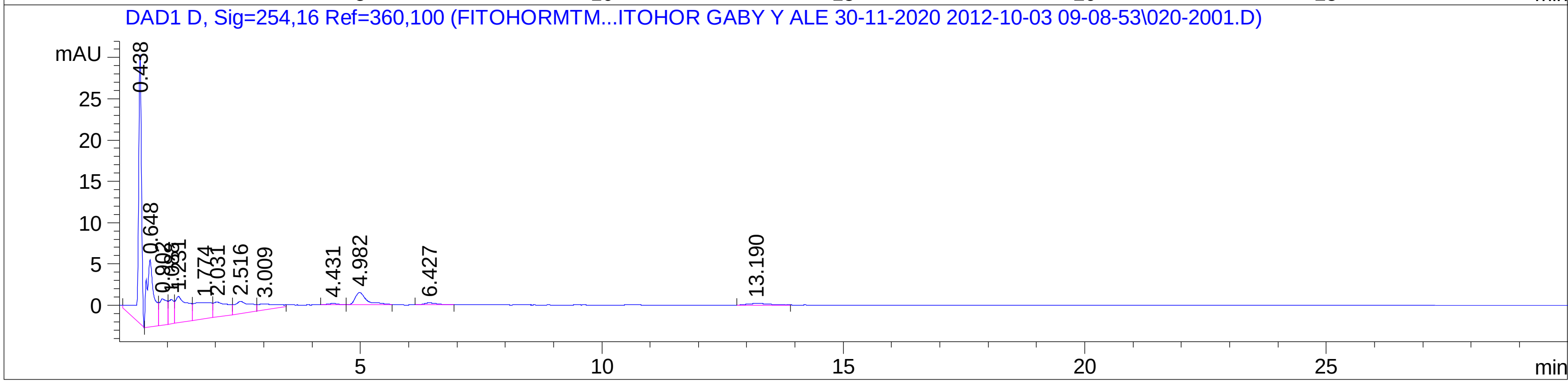

===

Area Percent Report

===

Sorted By : Signal
Multiplier: : 1.0000
Dilution: : 1.0000
Use Multiplier & Dilution Factor with ISTDs

Signal 1: DAD1 A, Sig=220,4 Ref=360,100

Peak #	RetTime [min]	Type	Width [min]	Area [mAU*s]	Height [mAU]	Area %
1	0.437	BV	0.0784	1080.80249	202.23077	13.8778
2	0.647	VV	0.2561	1309.44214	64.70721	16.8135
3	1.094	VV	0.1080	281.44611	37.31256	3.6138
4	1.218	VB	0.2127	604.75629	36.61064	7.7652
5	1.672	BB	0.4538	1227.96753	33.33240	15.7674
6	2.166	BV	0.2025	426.95029	29.91817	5.4821
7	2.515	VV	0.3938	927.94501	30.25707	11.9150
8	2.994	VV	0.3109	518.10553	24.47704	6.6526
9	3.529	VV	0.4281	691.51343	21.00220	8.8792
10	4.968	BV	0.3765	367.14426	12.88330	4.7142
11	5.329	VB	0.3222	243.77634	9.93753	3.1301
12	5.767	BV	0.2202	108.17395	7.00458	1.3890

Totals : 7788.02335 509.67349

Signal 2: DAD1 B, Sig=280,16 Ref=360,100

Peak #	RetTime [min]	Type	Width [min]	Area [mAU*s]	Height [mAU]	Area %
1	0.439	BV	0.0643	108.79005	27.41625	36.9614
2	0.654	VV	0.1460	52.12481	4.82572	17.7094
3	0.898	VV	0.1374	23.38253	2.28486	7.9442
4	1.083	VV	0.1501	17.60254	1.50880	5.9805
5	1.441	VV	0.1949	15.84943	1.04519	5.3848
6	1.686	VB	0.4798	46.19715	1.18773	15.6955
7	2.517	BB	0.2354	13.22023	7.65899e-1	4.4916
8	3.008	BB	0.3801	12.32340	4.27711e-1	4.1869
9	4.968	BB	0.2247	2.69793	1.55256e-1	0.9166
10	6.424	BB	0.2580	2.14631	1.11811e-1	0.7292

Totals : 294.33438 39.72923

Signal 3: DAD1 C, Sig=282,8 Ref=360,100

Peak #	RetTime [min]	Type	Width [min]	Area [mAU*s]	Height [mAU]	Area %
1	0.439	BV	0.0682	117.43665	27.26850	38.9868
2	0.656	VV	0.1487	53.24294	4.82713	17.6757
3	0.897	VV	0.2233	41.48877	2.35807	13.7735
4	1.447	VV	0.1952	16.41621	1.08102	5.4499
5	1.687	VV	0.4727	46.44794	1.21299	15.4199
6	2.521	VB	0.2855	15.56482	7.27794e-1	5.1672
7	3.005	BB	0.2730	8.42090	3.79354e-1	2.7956
8	5.008	BB	0.1987	2.20313	1.40721e-1	0.7314
Totals :				301.22136	37.99558	

Signal 4: DAD1 D, Sig=254,16 Ref=360,100

Peak #	RetTime [min]	Type	Width [min]	Area [mAU*s]	Height [mAU]	Area %
1	0.438	BV	0.0717	150.34004	32.63925	28.6235
2	0.648	VV	0.1378	82.01410	8.12090	15.6148
3	0.902	VV	0.1487	35.06346	3.17863	6.6758
4	1.088	VV	0.1074	21.82253	2.91332	4.1548
5	1.231	VV	0.2362	57.17685	3.17277	10.8860
6	1.774	VV	0.3124	48.87420	1.94981	9.3052
7	2.031	VV	0.2666	37.66275	1.81110	7.1707
8	2.516	VB	0.2991	33.48013	1.46226	6.3743
9	3.009	BB	0.3359	18.99587	7.24398e-1	3.6167
10	4.431	BV	0.2180	2.76969	1.81582e-1	0.5273
11	4.982	VB	0.2516	26.11632	1.51158	4.9723
12	6.427	BB	0.2711	4.64858	2.40879e-1	0.8851
13	13.190	BB	0.3976	6.26874	2.03363e-1	1.1935
Totals :				525.23326	58.10984	

===

*** End of Report ***
