# Supplementary material for: Influence of Silver Nanoparticles (AgNPs) on Vegetative Growth and Concentrations of Nutrients and Phytohormones in Tomato
Source: Plants (Basel). 2026 Jan 28;15(3):405. doi: 10.3390/plants15030405 (PMC12899181; doi:10.3390/plants15030405)
Supplement: Supplementary file 1 [file plants-15-00405-s001.zip › S1. HPLC Analysis (plants-4015186)/cv. Rio Grande/Roots/Control/RG-T-R-R3.pdf]

===

Acq. Operator : TMG Seq. Line : 21
Acq. Instrument : Instrument 1 Location : Vial 21
Injection Date : 10/3/2012 8:17:45 PM Inj : 1
Inj Volume : 200.0 µl
Different Inj Volume from Sequence ! Actual Inj Volume : 50.0 µl
Acq. Method : C:\CHEM32\1\DATA\FITOHORMTMG\FITOHOR GABY Y ALE 30-11-2020 2012-10-03 09-08-53\FITOHORMONAS DR SOTO.M
Last changed : 8/14/2013 11:13:25 AM by TMG
Analysis Method : C:\CHEM32\1\METHODS\LAVADO COLUMNA ACET.M
Last changed : 10/21/2012 12:24:49 PM by TMG
(modified after loading)

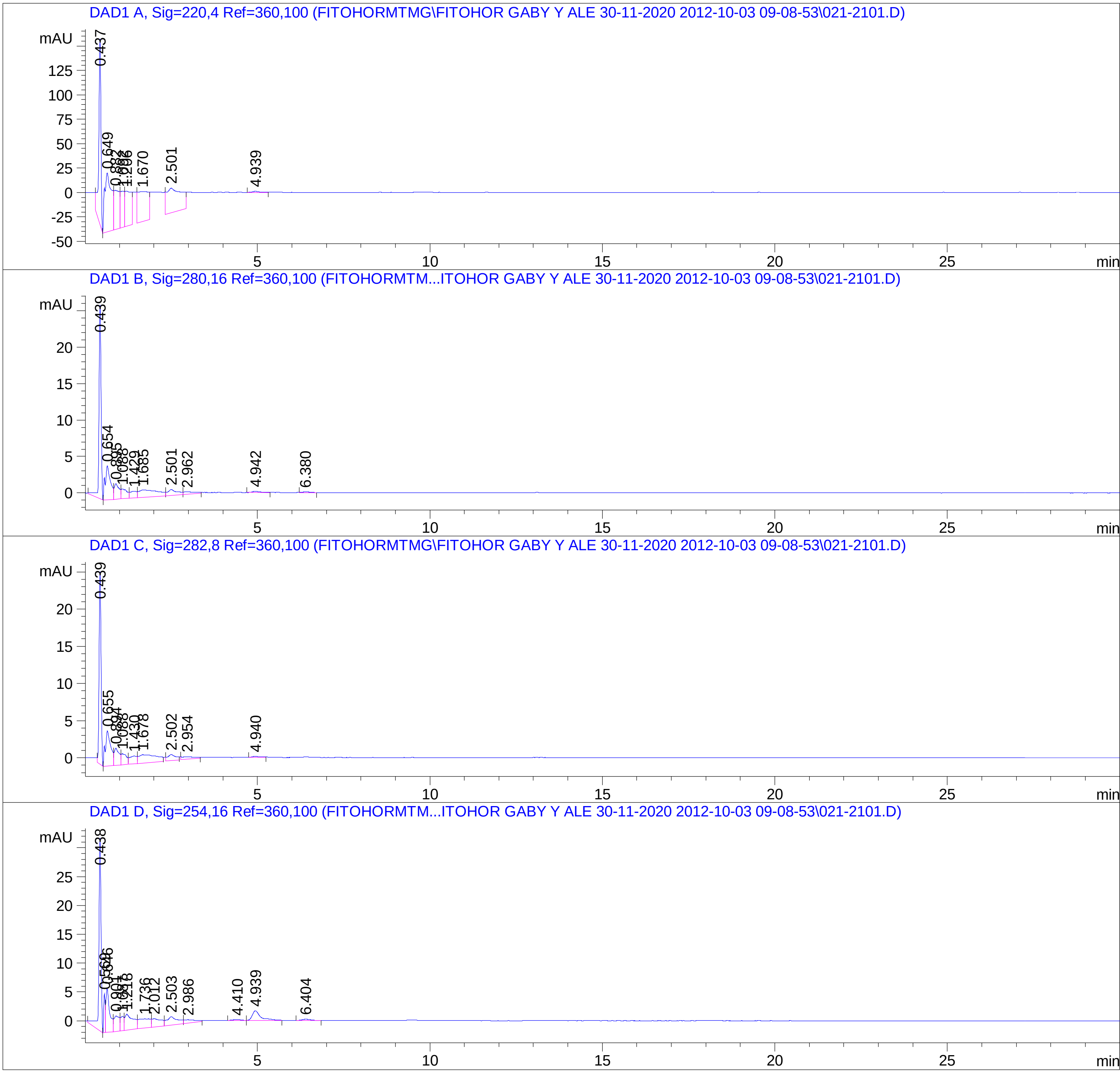

===

Area Percent Report

===

Sorted By : Signal
Multiplier: : 1.0000
Dilution: : 1.0000
Use Multiplier & Dilution Factor with ISTDs

Signal 1: DAD1 A, Sig=220,4 Ref=360,100

Peak #	RetTime [min]	Type	Width [min]	Area [mAU*s]	Height [mAU]	Area %
1	0.437	BV	0.0681	847.32275	189.71419	19.7294
2	0.649	VV	0.1800	825.29620	60.17337	19.2165
3	0.882	VV	0.1465	431.59756	39.81952	10.0495
4	1.092	VV	0.1110	287.85165	36.90656	6.7024
5	1.206	VB	0.1737	458.53137	35.74460	10.6766
6	1.670	BB	0.2860	671.72980	30.37869	15.6408
7	2.501	BB	0.3798	757.04535	25.39226	17.6273
8	4.939	BB	0.2134	15.35527	1.05746	0.3575

Totals : 4294.72996 419.18664

Signal 2: DAD1 B, Sig=280,16 Ref=360,100

Peak #	RetTime [min]	Type	Width [min]	Area [mAU*s]	Height [mAU]	Area %
1	0.439	BV	0.0675	112.98401	26.62101	40.6560
2	0.654	VV	0.1435	49.35719	4.66363	17.7606
3	0.895	VV	0.1346	21.27304	2.12908	7.6549
4	1.088	VV	0.1557	16.21015	1.35354	5.8330
5	1.429	VV	0.1925	12.54396	9.23034e-1	4.5138
6	1.685	VV	0.4826	38.23281	1.00788	13.7576
7	2.501	VV	0.2527	15.28529	7.93550e-1	5.5002
8	2.962	VB	0.3045	7.46882	3.42319e-1	2.6876
9	4.942	BB	0.2428	2.99384	1.72210e-1	1.0773
10	6.380	BB	0.2035	1.55333	9.77088e-2	0.5589

Totals : 277.90243 38.10397

Signal 3: DAD1 C, Sig=282,8 Ref=360,100

Peak #	RetTime [min]	Type	Width [min]	Area [mAU*s]	Height [mAU]	Area %
1	0.439	BV	0.0646	103.63766	25.93837	37.7231
2	0.655	VV	0.1457	51.11747	4.74329	18.6062
3	0.894	VV	0.1370	23.43752	2.29692	8.5310
4	1.088	VV	0.1422	16.24093	1.47789	5.9115
5	1.430	VV	0.2107	15.37727	1.03866	5.5972
6	1.678	VB	0.4431	39.97692	1.11826	14.5512
7	2.502	BB	0.2257	13.96254	8.24057e-1	5.0822
8	2.954	BB	0.3341	8.70023	3.57272e-1	3.1668
9	4.940	BB	0.2248	2.28231	1.39575e-1	0.8307

Totals : 274.73284 37.93430

Signal 4: DAD1 D, Sig=254,16 Ref=360,100

Peak #	RetTime [min]	Type	Width [min]	Area [mAU*s]	Height [mAU]	Area %
1	0.438	BV	0.0700	149.29733	33.51563	32.0283
2	0.569	VV	0.0458	19.57919	6.75644	4.2003
3	0.646	VV	0.1060	57.52491	7.62564	12.3407
4	0.901	VV	0.1462	29.15008	2.65379	6.2535
5	1.087	VV	0.1020	17.68825	2.45667	3.7946
6	1.218	VV	0.2253	47.67693	2.73583	10.2280
7	1.736	VV	0.3073	37.98801	1.58699	8.1495
8	2.012	VV	0.2486	26.88315	1.42185	5.7672
9	2.503	VB	0.2934	31.82471	1.43154	6.8273
10	2.986	BB	0.2913	13.10798	5.67837e-1	2.8120
11	4.410	BV	0.2175	2.81565	1.85117e-1	0.6040
12	4.939	VB	0.2525	28.83015	1.66071	6.1848
13	6.404	BB	0.2426	3.77527	2.15279e-1	0.8099

Totals : 466.14160 62.81331

==

*** End of Report ***
