# Supplementary material for: Influence of Silver Nanoparticles (AgNPs) on Vegetative Growth and Concentrations of Nutrients and Phytohormones in Tomato
Source: Plants (Basel). 2026 Jan 28;15(3):405. doi: 10.3390/plants15030405 (PMC12899181; doi:10.3390/plants15030405)
Supplement: Supplementary file 1 [file plants-15-00405-s001.zip › S1. HPLC Analysis (plants-4015186)/cv. Vengador/Leaves/10 ppm/V-10-L-R1.pdf]

Data File C:\CHEM32\...ORMTMG\FITOHOR GABY Y ALE 30-11-2020 2012-10-03 09-08-53\034-3401.D
Sample Name: 10 PPM VENGADOR HOJA R1

===

Acq. Operator : TMG   Seq. Line : 34
Acq. Instrument : Instrument 1   Location : Vial 34
Injection Date : 10/4/2012 3:15:13 AM   Inj : 1
                                                      Inj Volume : 200.0 µl
Different Inj Volume from Sequence !   Actual Inj Volume : 50.0 µl
Acq. Method : C:\CHEM32\1\DATA\FITOHORMTMG\FITOHOR GABY Y ALE 30-11-2020 2012-10-03 09-08-53\FITOHORMONAS DR SOTO.M
Last changed : 8/14/2013 11:13:25 AM by TMG
Analysis Method : C:\CHEM32\1\METHODS\LAVADO COLUMNA ACET.M
Last changed : 10/21/2012 12:24:49 PM by TMG
(modified after loading)

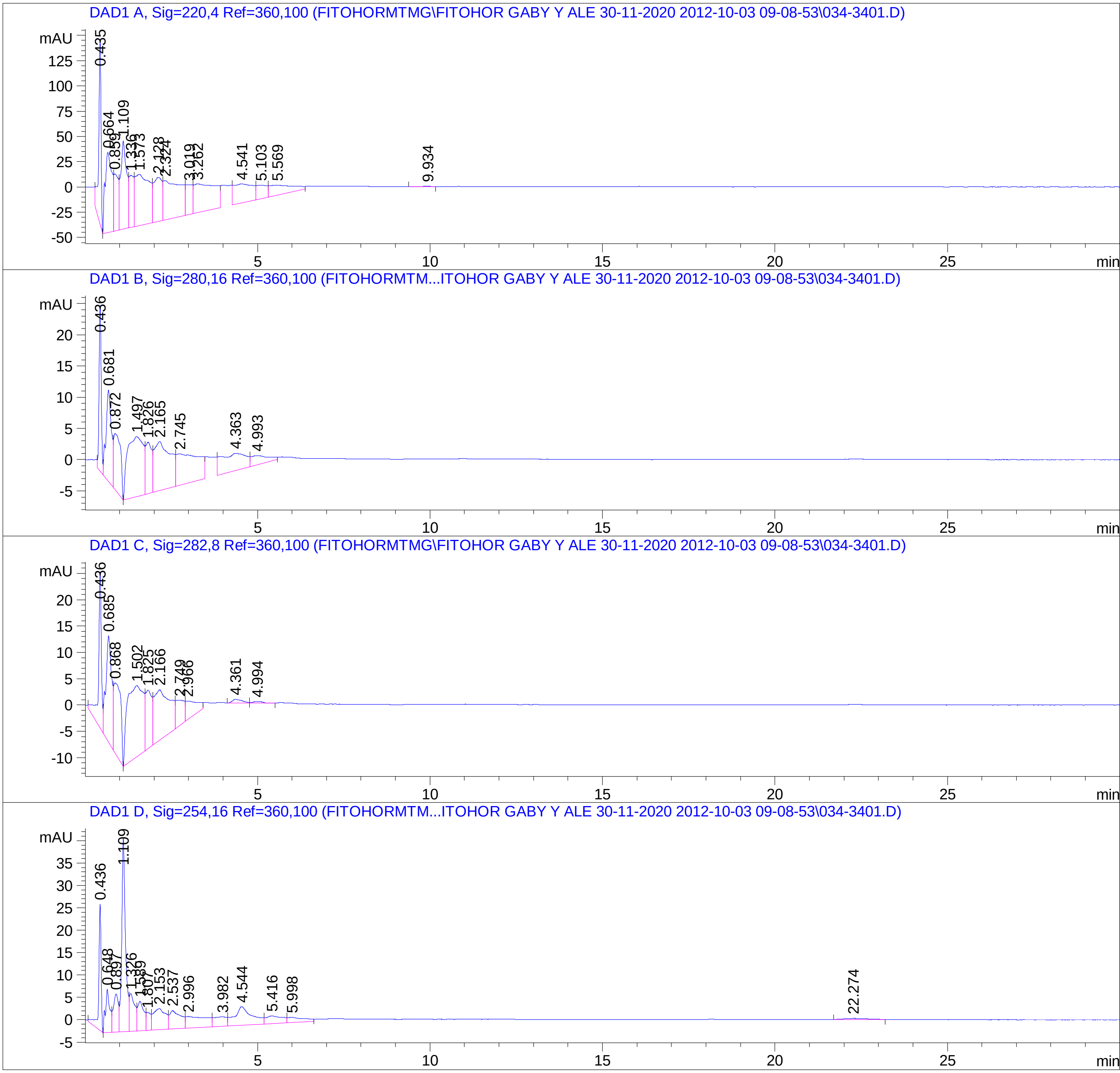

===

Area Percent Report

===

Sorted By : Signal
Multiplier: : 1.0000
Dilution: : 1.0000
Use Multiplier & Dilution Factor with ISTDs

Signal 1: DAD1 A, Sig=220,4 Ref=360,100

Peak #	RetTime [min]	Type	Width [min]	Area [mAU*s]	Height [mAU]	Area %
1	0.435	BV	0.0721	874.55646	181.79802	8.2468
2	0.664	VV	0.1868	1078.60986	79.26775	10.1710
3	0.859	VV	0.1347	515.70544	56.39817	4.8629
4	1.109	VV	0.1665	1091.95459	86.96488	10.2968
5	1.336	VV	0.1223	451.75995	51.52519	4.2600
6	1.573	VV	0.3693	1470.16858	50.85729	13.8632
7	2.128	VV	0.2459	753.97479	43.57176	7.1098
8	2.324	VB	0.4173	1299.91174	38.95730	12.2578
9	3.019	BV	0.1987	410.88431	29.45601	3.8745
10	3.262	VB	0.5272	1205.10681	28.62390	11.3638
11	4.541	BV	0.5107	718.78333	18.77072	6.7779
12	5.103	VV	0.2779	274.29803	13.23126	2.5865
13	5.569	VB	0.5787	452.10837	9.71879	4.2632
14	9.934	VV	0.3061	6.97167	2.96723e-1	0.0657
Totals :				1.06048e4	689.43776	

Signal 2: DAD1 B, Sig=280,16 Ref=360,100

Peak #	RetTime [min]	Type	Width [min]	Area [mAU*s]	Height [mAU]	Area %
1	0.436	BV	0.0691	116.34962	26.57936	7.9036
2	0.681	VV	0.1557	155.21518	14.64180	10.5438
3	0.872	VV	0.1864	131.74422	9.01822	8.9494
4	1.497	VV	0.4047	302.81717	9.58112	20.5704
5	1.826	VV	0.1710	100.84690	8.22529	6.8505
6	2.165	VV	0.4113	254.09045	7.85691	17.2604
7	2.745	VB	0.5529	221.56769	5.02007	15.0511
8	4.363	BV	0.6296	139.93456	2.76903	9.5058
9	4.993	VB	0.4414	49.53801	1.47664	3.3651
Totals :				1472.10379	85.16842	

Signal 3: DAD1 C, Sig=282,8 Ref=360,100

Peak #	RetTime [min]	Type	Width [min]	Area [mAU*s]	Height [mAU]	Area %
1	0.436	BV	0.0882	179.14667	29.77132	10.7726
2	0.685	VV	0.1651	234.39984	20.24476	14.0952
3	0.868	VV	0.1877	197.49518	13.26268	11.8760
4	1.502	VV	0.4114	433.54968	13.47528	26.0707
5	1.825	VV	0.1766	141.31090	11.09302	8.4975
6	2.166	VV	0.4026	300.97656	9.52577	18.0986
7	2.749	VV	0.2556	83.19464	4.81361	5.0027
8	2.966	VB	0.2749	76.56112	3.61749	4.6039
9	4.361	BV	0.2617	12.51849	6.64705e-1	0.7528
10	4.994	VB	0.2127	3.82605	2.52664e-1	0.2301

Totals : 1662.97913 106.72131

Signal 4: DAD1 D, Sig=254,16 Ref=360,100

Peak #	RetTime [min]	Type	Width [min]	Area [mAU*s]	Height [mAU]	Area %
1	0.436	BV	0.0748	137.38448	28.21443	9.2584
2	0.648	VV	0.1205	84.13982	9.58059	5.6702
3	0.897	VV	0.1521	87.51881	8.50428	5.8979
4	1.109	VV	0.1108	336.77838	43.25281	22.6956
5	1.326	VV	0.1509	93.46898	8.46121	6.2989
6	1.589	VV	0.1733	83.33498	6.60202	5.6160
7	1.807	VV	0.1279	36.36023	4.00738	2.4503
8	2.153	VV	0.3461	116.95183	4.70335	7.8814
9	2.537	VV	0.2971	93.74420	4.09529	6.3175
10	2.996	VV	0.5090	107.10281	2.57639	7.2177
11	3.982	VV	0.3341	55.15699	2.11585	3.7170
12	4.544	VV	0.4645	144.82819	4.13588	9.7600
13	5.416	VV	0.4519	56.14491	1.68013	3.7836
14	5.998	VB	0.4465	38.12727	1.12181	2.5694
15	22.274	BB	0.5983	12.85024	2.56575e-1	0.8660

Totals : 1483.89211 129.30800

==

*** End of Report ***
