# Supplementary material for: Influence of Silver Nanoparticles (AgNPs) on Vegetative Growth and Concentrations of Nutrients and Phytohormones in Tomato
Source: Plants (Basel). 2026 Jan 28;15(3):405. doi: 10.3390/plants15030405 (PMC12899181; doi:10.3390/plants15030405)
Supplement: Supplementary file 1 [file plants-15-00405-s001.zip › S1. HPLC Analysis (plants-4015186)/cv. Vengador/Leaves/10 ppm/V-10-L-R2.pdf]

===

Acq. Operator : TMG　　Seq. Line : 35
Acq. Instrument : Instrument 1　　Location : Vial 35
Injection Date : 10/4/2012 3:47:19 AM　　Inj : 1
Inj Volume : 200.0 µl
Different Inj Volume from Sequence !　　Actual Inj Volume : 50.0 µl
Acq. Method : C:\CHEM32\1\DATA\FITOHORMTMG\FITOHOR GABY Y ALE 30-11-2020 2012-10-03 09-08-53\FITOHORMONAS DR SOTO.M
Last changed : 8/14/2013 11:13:25 AM by TMG
Analysis Method : C:\CHEM32\1\METHODS\LAVADO COLUMNA ACET.M
Last changed : 10/21/2012 12:24:49 PM by TMG
(modified after loading)

DAD1 A, Sig=220,4 Ref=360,100 (FITOHORMTMG\FITOHOR GABY Y ALE 30-11-2020 2012-10-03 09-08-53\035-3501.D)

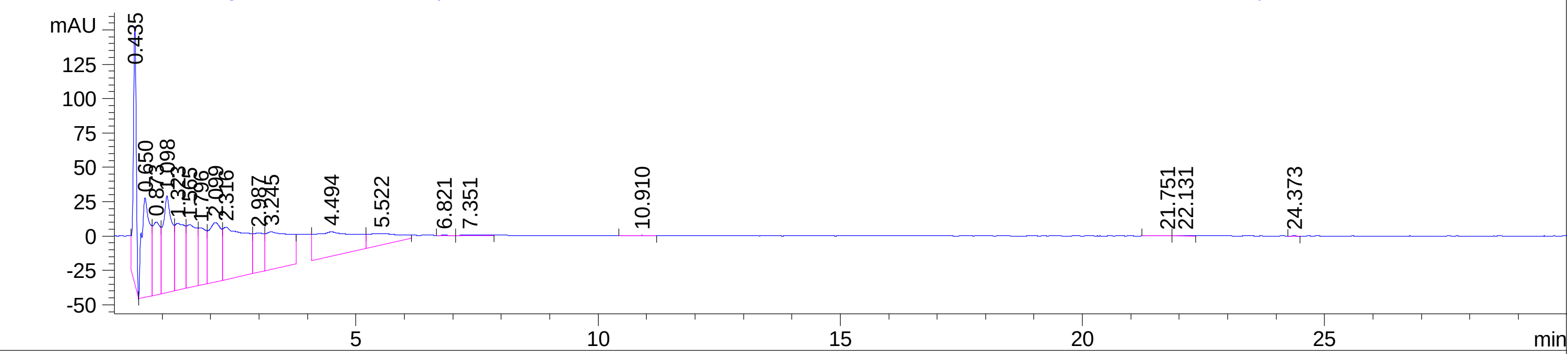

DAD1 B, Sig=280,16 Ref=360,100 (FITOHORMTM...ITOHOR GABY Y ALE 30-11-2020 2012-10-03 09-08-53\035-3501.D)

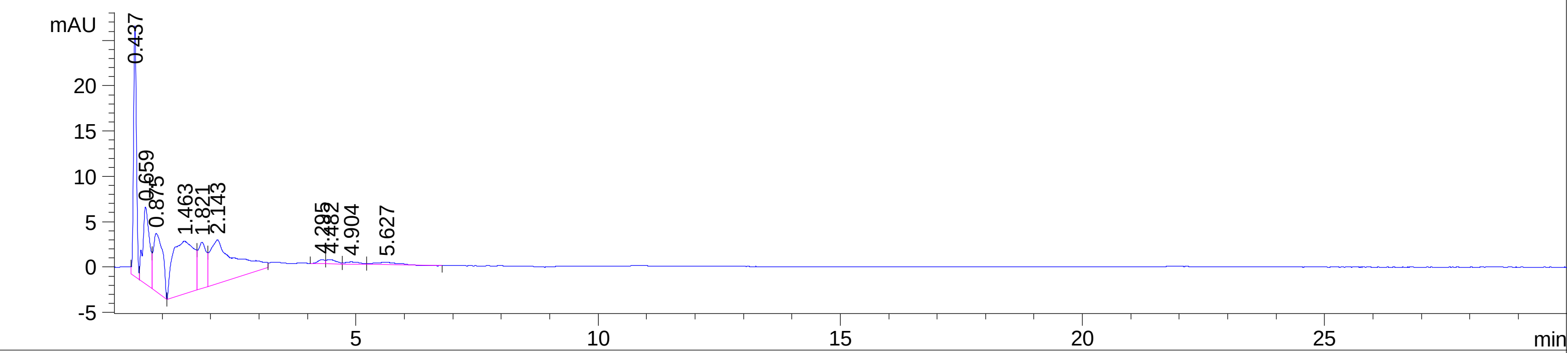

DAD1 C, Sig=282,8 Ref=360,100 (FITOHORMTMG\FITOHOR GABY Y ALE 30-11-2020 2012-10-03 09-08-53\035-3501.D)

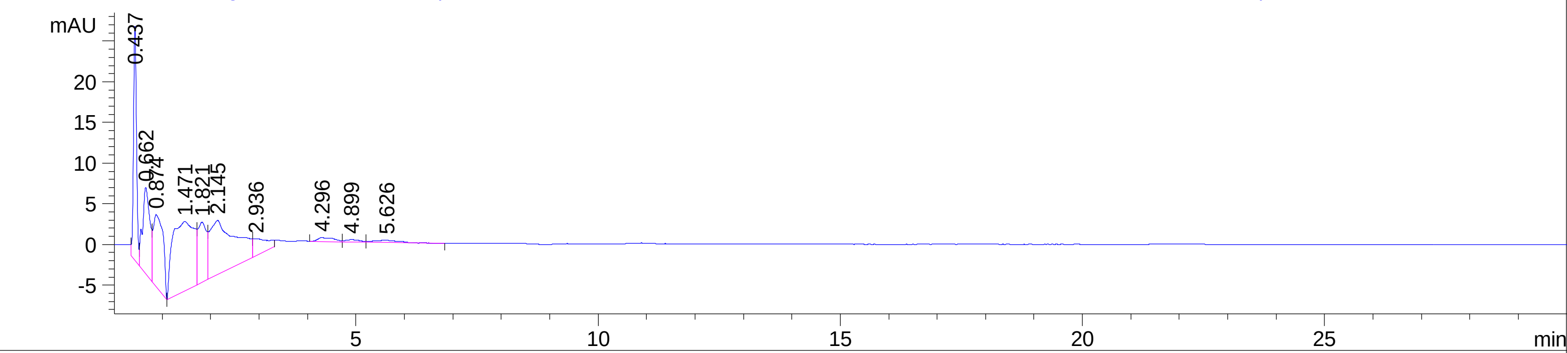

DAD1 D, Sig=254,16 Ref=360,100 (FITOHORMTM...ITOHOR GABY Y ALE 30-11-2020 2012-10-03 09-08-53\035-3501.D)

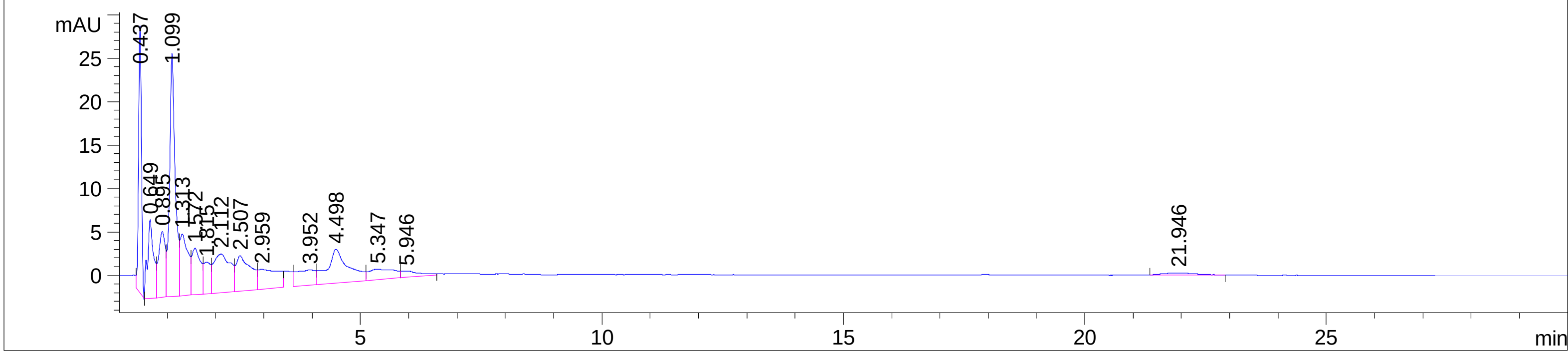

===

Area Percent Report

===

Sorted By : Signal
Multiplier: : 1.0000
Dilution: : 1.0000
Use Multiplier & Dilution Factor with ISTDs

Signal 1: DAD1 A, Sig=220,4 Ref=360,100

Peak #	RetTime [min]	Type	Width [min]	Area [mAU*s]	Height [mAU]	Area %
1	0.435	BV	0.0690	789.11804	187.72897	8.0957
2	0.650	VV	0.1610	874.72437	72.36367	8.9739
3	0.873	VV	0.1521	564.11200	53.04820	5.7873
4	1.098	VV	0.1770	917.64960	69.98856	9.4143
5	1.323	VV	0.1872	696.15540	48.58750	7.1420
6	1.565	VV	0.1886	643.74146	45.62149	6.6042
7	1.796	VV	0.1521	454.91602	41.43897	4.6670
8	2.099	VV	0.2477	752.09192	43.08278	7.7158
9	2.316	VV	0.4064	1249.66785	38.12546	12.8205
10	2.987	VV	0.2103	414.60931	28.40152	4.2535
11	3.245	VB	0.4469	963.73022	27.36610	9.8870
12	4.494	BV	0.7312	1024.52563	17.59772	10.5107
13	5.522	VB	0.5552	366.65958	8.43396	3.7616
14	6.821	BB	0.1763	3.18154	2.64588e-1	0.0326
15	7.351	BB	0.3326	11.27024	4.37471e-1	0.1156
16	10.910	BV	0.3156	5.41109	2.50926e-1	0.0555
17	21.751	BV	0.2658	6.92834	3.45775e-1	0.0711
18	22.131	VV	0.2510	7.25373	3.72745e-1	0.0744
19	24.373	BV	0.1117	1.66213	2.43185e-1	0.0171

Totals : 9747.40846 683.69960

Signal 2: DAD1 B, Sig=280,16 Ref=360,100

Peak #	RetTime [min]	Type	Width [min]	Area [mAU*s]	Height [mAU]	Area %
1	0.437	BV	0.0665	115.00897	27.66531	15.8999
2	0.659	VV	0.1395	82.89849	8.51820	11.4607
3	0.875	VV	0.1939	86.94247	6.42226	12.0197
4	1.463	VV	0.3891	179.10646	5.81857	24.7614
5	1.821	VV	0.1659	60.32895	5.10528	8.3404
6	2.143	VB	0.4507	171.99306	4.79286	23.7779
7	4.295	VV	0.1541	4.62558	4.42008e-1	0.6395
8	4.482	VV	0.2016	6.96000	4.67802e-1	0.9622
9	4.904	VV	0.2774	5.56950	2.60534e-1	0.7700

Peak #	RetTime [min]	Type	Width [min]	Area [mAU*s]	Height [mAU]	Area %
10	5.627	VB	0.4658	9.89700	2.67377e-1	1.3683

Totals : 723.33048 59.76019

Signal 3: DAD1 C, Sig=282,8 Ref=360,100

Peak #	RetTime [min]	Type	Width [min]	Area [mAU*s]	Height [mAU]	Area %
1	0.437	BV	0.0687	125.21745	28.81931	12.0820
2	0.662	VV	0.1522	114.91118	10.61924	11.0875
3	0.874	VV	0.2036	126.53022	8.80855	12.2086
4	1.471	VV	0.4020	267.21930	8.51809	25.7834
5	1.821	VV	0.1740	92.87357	7.42360	8.9612
6	2.145	VV	0.4543	241.16083	6.66214	23.2691
7	2.936	VB	0.2617	40.06855	2.09010	3.8661
8	4.296	VV	0.3186	12.57178	5.08250e-1	1.2130
9	4.899	VV	0.2758	6.34021	2.96095e-1	0.6118
10	5.626	VB	0.4410	9.50645	2.73882e-1	0.9173

Totals : 1036.39954 74.01924

Signal 4: DAD1 D, Sig=254,16 Ref=360,100

Peak #	RetTime [min]	Type	Width [min]	Area [mAU*s]	Height [mAU]	Area %
1	0.437	BV	0.0661	127.22128	30.83081	10.5889
2	0.649	VV	0.1212	79.34524	8.96572	6.6041
3	0.895	VV	0.1459	73.65340	7.54143	6.1304
4	1.099	VV	0.1180	230.41634	27.99226	19.1781
5	1.313	VV	0.1545	81.86277	7.10141	6.8136
6	1.572	VV	0.1669	65.79391	5.37294	5.4762
7	1.815	VV	0.1409	35.76715	3.63299	2.9770
8	2.112	VV	0.3448	107.68352	4.41160	8.9628
9	2.507	VV	0.2789	86.56745	4.05736	7.2052
10	2.959	VB	0.3716	66.94765	2.27344	5.5722
11	3.952	BV	0.3612	49.50996	1.77620	4.1208
12	4.498	VV	0.4072	120.19760	3.90438	10.0043
13	5.347	VV	0.4684	44.92098	1.23476	3.7389
14	5.946	VB	0.3518	19.34510	7.52803e-1	1.6101
15	21.946	BB	0.6114	12.22196	2.38681e-1	1.0173

Totals : 1201.45430 110.08677

===

*** End of Report ***
