# Supplementary material for: Influence of Silver Nanoparticles (AgNPs) on Vegetative Growth and Concentrations of Nutrients and Phytohormones in Tomato
Source: Plants (Basel). 2026 Jan 28;15(3):405. doi: 10.3390/plants15030405 (PMC12899181; doi:10.3390/plants15030405)
Supplement: Supplementary file 1 [file plants-15-00405-s001.zip › S1. HPLC Analysis (plants-4015186)/cv. Vengador/Leaves/10 ppm/V-10-L-R3.pdf]

Data File C:\CHEM32\...ORMTMG\FITOHOR GABY Y ALE 30-11-2020 2012-10-03 09-08-53\036-3601.D
Sample Name: 10 PPM VENGADOR HOJA R3

===

Acq. Operator	: TMG	Seq. Line :	36
Acq. Instrument	: Instrument 1	Location :	Vial 36
Injection Date	: 10/4/2012 4:19:27 AM	Inj :	1
		Inj Volume :	200.0 µl
Different Inj Volume from Sequence !		Actual Inj Volume :	50.0 µl

Acq. Method : C:\CHEM32\1\DATA\FITOHORMTMG\FITOHOR GABY Y ALE 30-11-2020 2012-10-03 09-08-53\FITOHORMONAS DR SOTO.M
Last changed : 8/14/2013 11:13:25 AM by TMG
Analysis Method : C:\CHEM32\1\METHODS\LAVADO COLUMNA ACET.M
Last changed : 10/21/2012 12:24:49 PM by TMG
(modified after loading)

DAD1 A, Sig=220,4 Ref=360,100 (FITOHORMTMG\FITOHOR GABY Y ALE 30-11-2020 2012-10-03 09-08-53\036-3601.D)

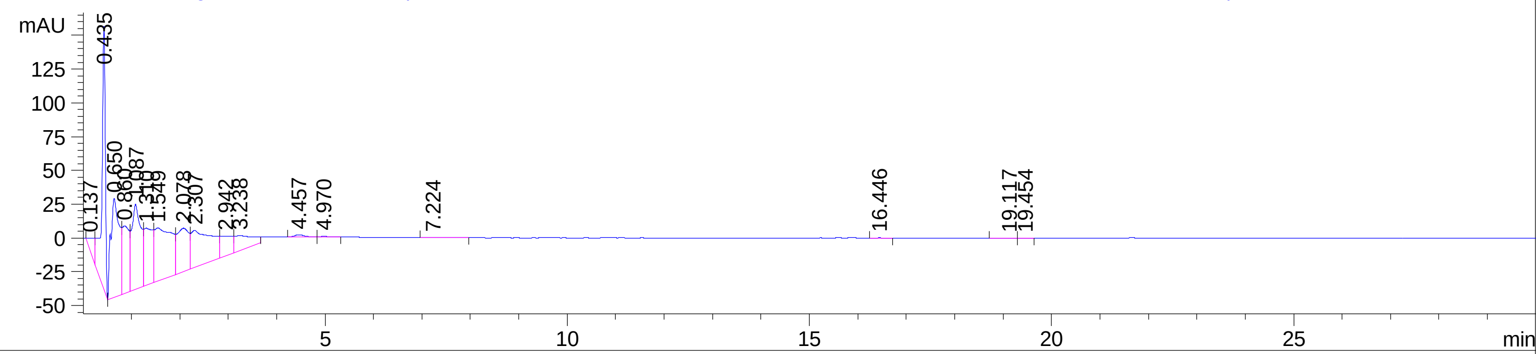

DAD1 B, Sig=280,16 Ref=360,100 (FITOHORMTM...ITOHOR GABY Y ALE 30-11-2020 2012-10-03 09-08-53\036-3601.D)

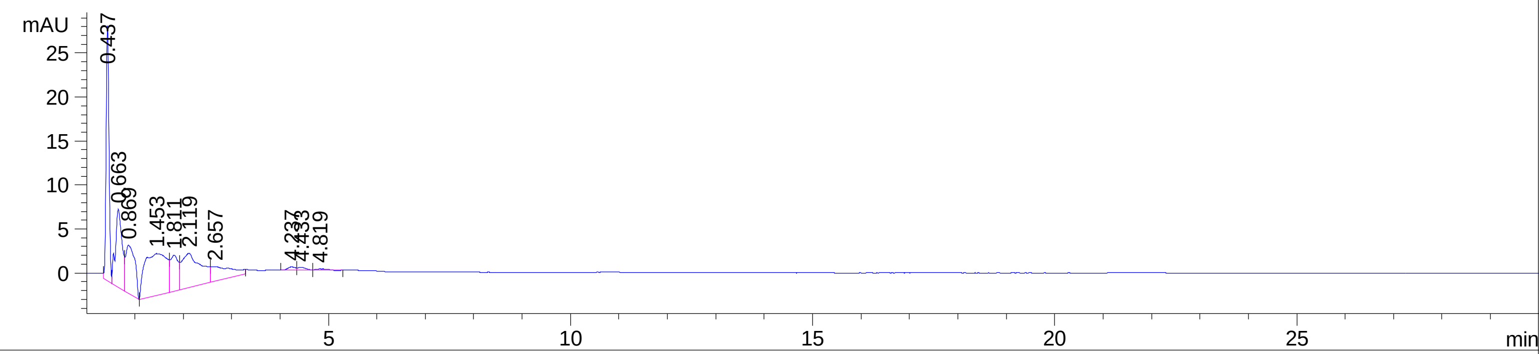

DAD1 C, Sig=282,8 Ref=360,100 (FITOHORMTMG\FITOHOR GABY Y ALE 30-11-2020 2012-10-03 09-08-53\036-3601.D)

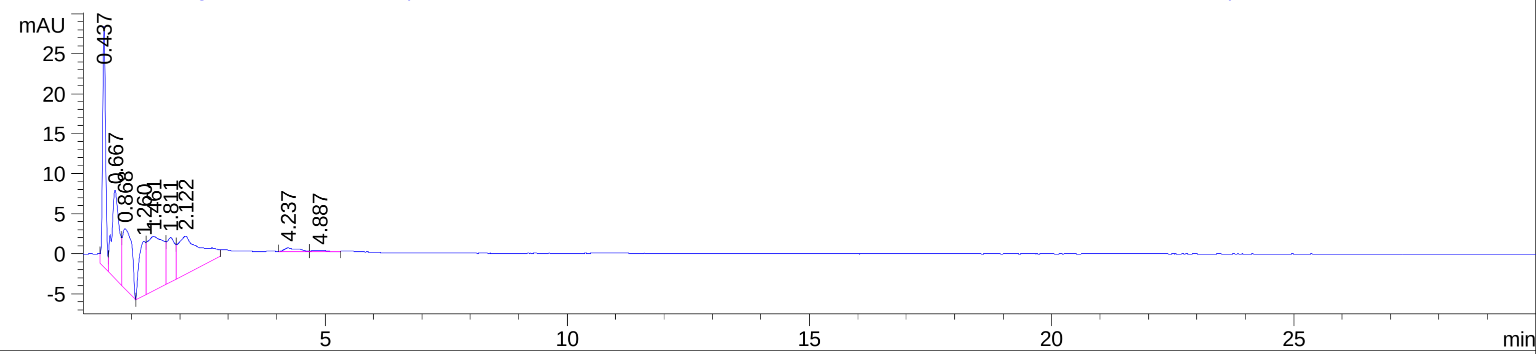

DAD1 D, Sig=254,16 Ref=360,100 (FITOHORMTM...ITOHOR GABY Y ALE 30-11-2020 2012-10-03 09-08-53\036-3601.D)

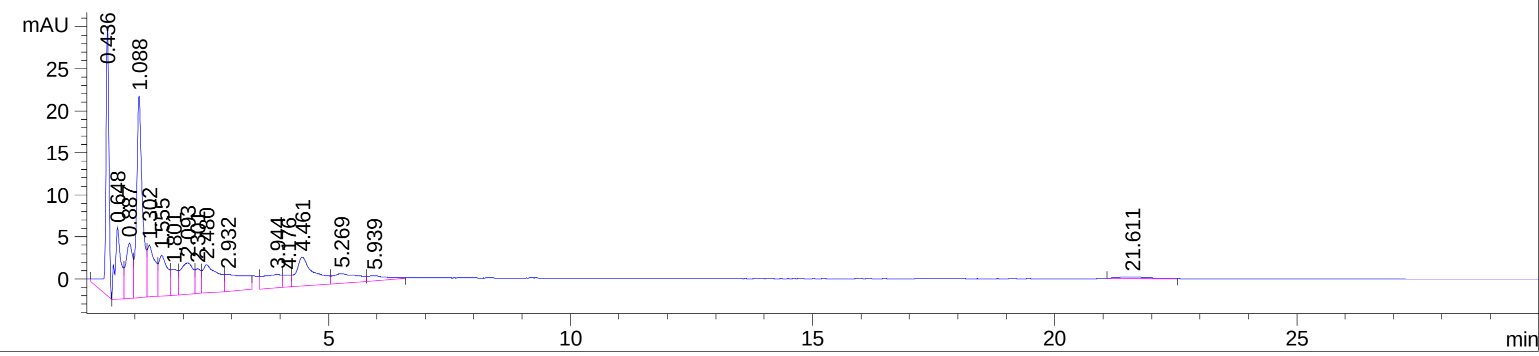

===

Area Percent Report

===

Sorted By : Signal
Multiplier: : 1.0000
Dilution: : 1.0000
Use Multiplier & Dilution Factor with ISTDs

Signal 1: DAD1 A, Sig=220,4 Ref=360,100

Peak #	RetTime [min]	Type	Width [min]	Area [mAU*s]	Height [mAU]	Area %
1	0.137	BV	0.1864	111.02087	7.97357	1.6559
2	0.435	VV	0.0751	978.06299	193.23228	14.5881
3	0.650	VV	0.1623	891.89606	73.10905	13.3029
4	0.860	VV	0.1446	499.24554	49.96130	7.4464
5	1.087	VV	0.1745	831.77301	62.80676	12.4062
6	1.310	VV	0.1692	541.27350	42.33461	8.0733
7	1.549	VV	0.3061	935.68109	39.25858	13.9560
8	2.078	VV	0.2496	554.06372	32.06289	8.2640
9	2.307	VV	0.3638	786.78827	27.33453	11.7352
10	2.942	VV	0.2273	246.63251	14.73084	3.6786
11	3.238	VB	0.3382	281.44135	11.16220	4.1978
12	4.457	BB	0.2018	20.57166	1.60232	0.3068
13	4.970	BB	0.2361	6.07983	3.69328e-1	0.0907
14	7.224	BB	0.4176	13.34007	4.03656e-1	0.1990
15	16.446	BB	0.1170	1.58155	2.39706e-1	0.0236
16	19.117	BV	0.2161	3.25614	2.26018e-1	0.0486
17	19.454	VV	0.1183	1.80736	2.23704e-1	0.0270

Totals : 6704.51552 557.03134

Signal 2: DAD1 B, Sig=280,16 Ref=360,100

Peak #	RetTime [min]	Type	Width [min]	Area [mAU*s]	Height [mAU]	Area %
1	0.437	BV	0.0662	119.89820	28.99700	18.8126
2	0.663	VV	0.1389	85.81355	8.86419	13.4646
3	0.869	VV	0.1894	71.78117	5.46034	11.2628
4	1.453	VV	0.3941	150.95148	4.73269	23.6851
5	1.811	VV	0.1599	46.84855	4.08224	7.3508
6	2.119	VV	0.3479	105.33710	3.89031	16.5279
7	2.657	VB	0.3612	46.18222	1.63644	7.2462
8	4.237	BV	0.1658	4.12947	3.82988e-1	0.6479
9	4.433	VV	0.1815	4.01381	3.26230e-1	0.6298
10	4.819	VB	0.2098	2.37247	1.45837e-1	0.3723

Totals : 637.32802 58.51825

Signal 3: DAD1 C, Sig=282,8 Ref=360,100

Peak #	RetTime [min]	Type	Width [min]	Area [mAU*s]	Height [mAU]	Area %
1	0.437	BV	0.0681	129.52296	30.14208	16.1876
2	0.667	VV	0.1519	117.84388	11.09686	14.7279
3	0.868	VV	0.1972	103.40553	7.48498	12.9234
4	1.260	VV	0.1561	62.74918	6.77512	7.8423
5	1.461	VV	0.3075	155.84941	6.74717	19.4778
6	1.811	VV	0.1649	65.95037	5.53979	8.2424
7	2.122	VB	0.4056	153.68341	4.77363	19.2071
8	4.237	BV	0.2721	8.52520	4.28555e-1	1.0655
9	4.887	VB	0.2001	2.60920	1.61831e-1	0.3261

Totals : 800.13915 73.15001

Signal 4: DAD1 D, Sig=254,16 Ref=360,100

Peak #	RetTime [min]	Type	Width [min]	Area [mAU*s]	Height [mAU]	Area %
1	0.436	BV	0.0719	148.68121	32.15681	13.7702
2	0.648	VV	0.1185	71.35634	8.45528	6.6087
3	0.887	VV	0.1449	64.38033	6.53791	5.9626
4	1.088	VV	0.1187	198.65291	23.97345	18.3984
5	1.302	VV	0.1516	68.19375	6.13935	6.3158
6	1.555	VV	0.1732	61.56576	4.81348	5.7019
7	1.801	VV	0.1360	29.73772	3.09798	2.7542
8	2.093	VV	0.2731	67.50571	3.69592	6.2521
9	2.301	VV	0.1096	22.39806	2.91661	2.0744
10	2.480	VV	0.2866	73.53535	3.34293	6.8105
11	2.932	VB	0.3935	62.41312	2.00329	5.7804
12	3.944	BV	0.3522	42.90608	1.57303	3.9738
13	4.176	VV	0.1636	16.50859	1.44120	1.5290
14	4.461	VV	0.3372	86.81383	3.45445	8.0403
15	5.269	VV	0.4623	39.13875	1.11846	3.6249
16	5.939	VB	0.3720	17.40852	6.22919e-1	1.6123
17	21.611	BB	0.5682	8.53613	1.78407e-1	0.7906

Totals : 1079.73217 105.52148

===

*** End of Report ***
