# Supplementary material for: Influence of Silver Nanoparticles (AgNPs) on Vegetative Growth and Concentrations of Nutrients and Phytohormones in Tomato
Source: Plants (Basel). 2026 Jan 28;15(3):405. doi: 10.3390/plants15030405 (PMC12899181; doi:10.3390/plants15030405)
Supplement: Supplementary file 1 [file plants-15-00405-s001.zip › S1. HPLC Analysis (plants-4015186)/cv. Vengador/Leaves/5 ppm/V-5-L-R1.pdf]

===

Acq. Operator : TMG   Seq. Line : 22
Acq. Instrument : Instrument 1   Location : Vial 22
Injection Date : 10/3/2012 8:49:53 PM   Inj : 1
                                                       Inj Volume : 200.0 µl
Different Inj Volume from Sequence !   Actual Inj Volume : 50.0 µl
Acq. Method : C:\CHEM32\1\DATA\FITOHORMTMG\FITOHOR GABY Y ALE 30-11-2020 2012-10-03 09-08-53\FITOHORMONAS DR SOTO.M
Last changed : 8/14/2013 11:13:25 AM by TMG
Analysis Method : C:\CHEM32\1\METHODS\LAVADO COLUMNA ACET.M
Last changed : 10/21/2012 12:24:49 PM by TMG
(modified after loading)

DAD1 A, Sig=220,4 Ref=360,100 (FITOHORMTMG\FITOHOR GABY Y ALE 30-11-2020 2012-10-03 09-08-53\022-2201.D)

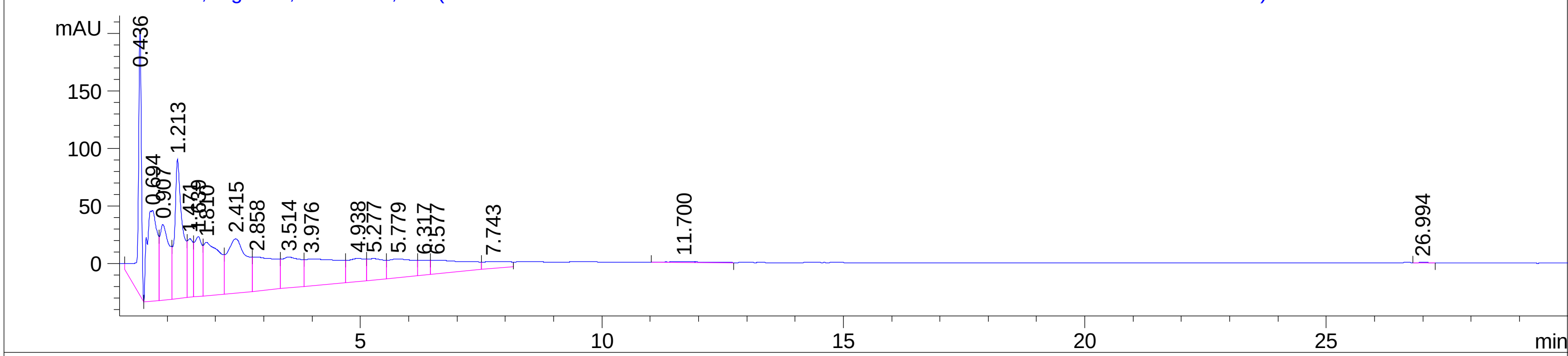

DAD1 B, Sig=280,16 Ref=360,100 (FITOHORMTM...ITOHOR GABY Y ALE 30-11-2020 2012-10-03 09-08-53\022-2201.D)

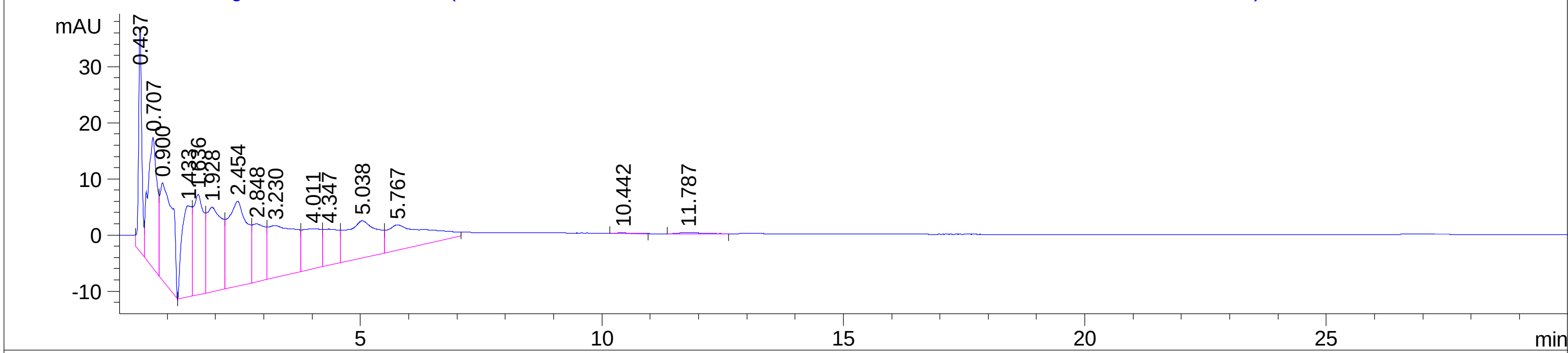

DAD1 C, Sig=282,8 Ref=360,100 (FITOHORMTMG\FITOHOR GABY Y ALE 30-11-2020 2012-10-03 09-08-53\022-2201.D)

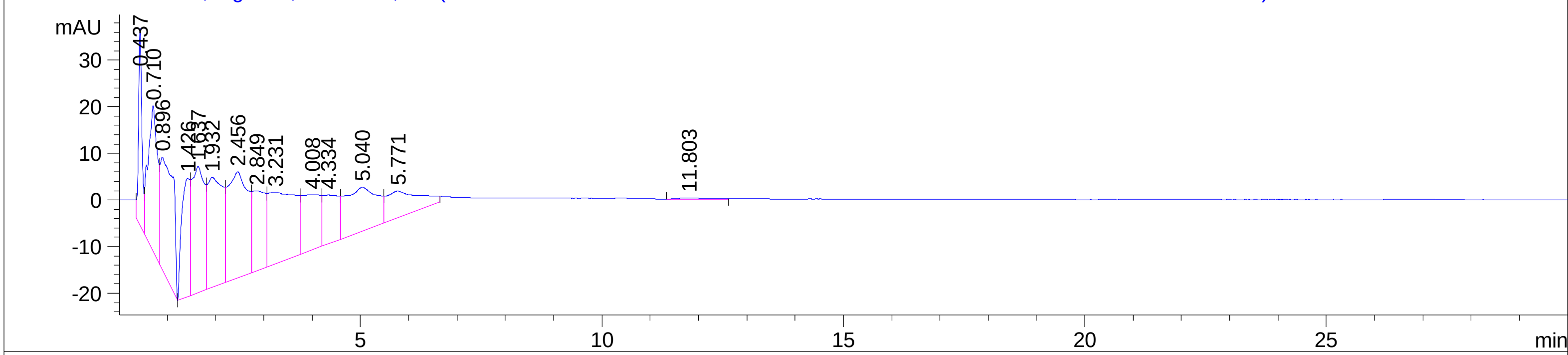

DAD1 D, Sig=254,16 Ref=360,100 (FITOHORMTM...ITOHOR GABY Y ALE 30-11-2020 2012-10-03 09-08-53\022-2201.D)

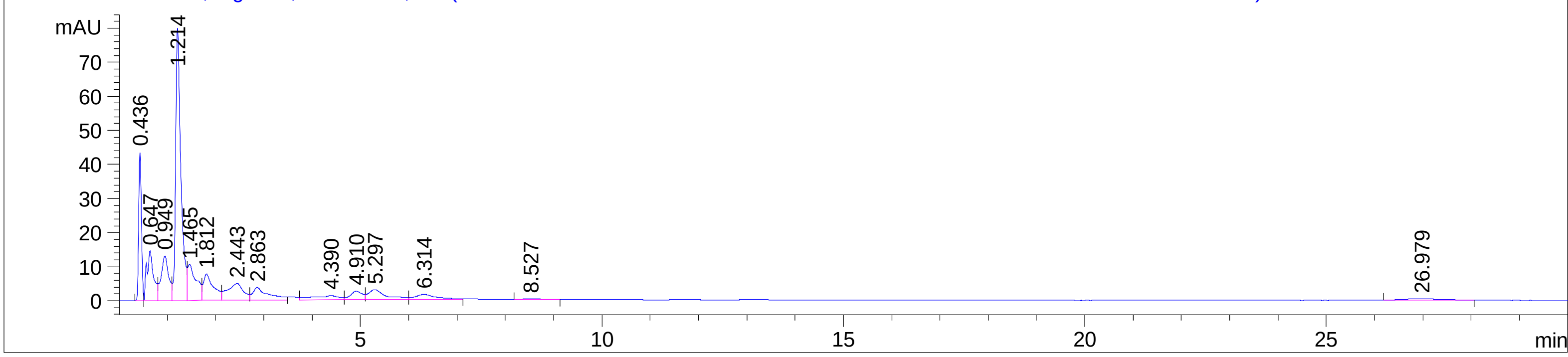

===

Area Percent Report

===

Sorted By : Signal
Multiplier: : 1.0000
Dilution: : 1.0000
Use Multiplier & Dilution Factor with ISTDs

Signal 1: DAD1 A, Sig=220,4 Ref=360,100

Peak #	RetTime [min]	Type	Width [min]	Area [mAU*s]	Height [mAU]	Area %
1	0.436	BV	0.0732	1130.93616	230.51549	8.4915
2	0.694	VV	0.2106	1144.60107	78.27274	8.5941
3	0.907	VV	0.1867	872.55573	65.79196	6.5515
4	1.213	VV	0.1577	1383.92664	120.74654	10.3911
5	1.471	VV	0.1145	413.03680	50.97004	3.1012
6	1.639	VV	0.1487	557.76166	52.18975	4.1879
7	1.810	VV	0.2935	1077.70898	46.65635	8.0919
8	2.415	VV	0.3883	1324.67102	47.07197	9.9461
9	2.858	VV	0.4279	975.87683	29.65730	7.3273
10	3.514	VV	0.3658	744.07446	26.97540	5.5868
11	3.976	VV	0.5916	1111.17175	23.16713	8.3431
12	4.938	VV	0.3519	503.76154	19.86001	3.7824
13	5.277	VV	0.3089	432.00980	18.46642	3.2437
14	5.779	VV	0.4898	596.05353	16.33310	4.4754
15	6.317	VV	0.2203	195.19756	12.91674	1.4656
16	6.577	VB	0.6232	602.31152	11.96713	4.5224
17	7.743	BV	0.4407	216.90666	5.95796	1.6286
18	11.700	BB	0.5401	31.55478	7.18934e-1	0.2369
19	26.994	BB	0.2214	4.32795	2.78306e-1	0.0325

Totals : 1.33184e4 858.51324

Signal 2: DAD1 B, Sig=280,16 Ref=360,100

Peak #	RetTime [min]	Type	Width [min]	Area [mAU*s]	Height [mAU]	Area %
1	0.437	BV	0.0708	181.29822	40.03060	5.1849
2	0.707	VV	0.1723	303.99783	23.29156	8.6940
3	0.900	VV	0.2349	322.30829	17.33965	9.2177
4	1.433	VV	0.2228	226.66696	16.24332	6.4824
5	1.636	VV	0.2020	267.22491	17.91356	7.6424
6	1.928	VV	0.2938	335.29822	15.05656	9.5892
7	2.454	VV	0.3601	417.11304	15.10810	11.9290
8	2.848	VV	0.2516	190.19795	10.29267	5.4395
9	3.230	VV	0.5114	359.78363	9.25698	10.2894

Peak #	RetTime [min]	Type	Width [min]	Area [mAU*s]	Height [mAU]	Area %
10	4.011	VV	0.3527	192.45192	7.18111	5.5039
11	4.347	VV	0.2745	137.67372	6.46355	3.9373
12	5.038	VV	0.5891	301.79944	6.62951	8.6312
13	5.767	VB	0.7108	250.77094	4.48201	7.1718
14	10.442	BB	0.3191	2.49230	9.78714e-2	0.0713
15	11.787	BB	0.3842	7.55081	2.41867e-1	0.2159

Totals : 3496.62818 189.62891

Signal 3: DAD1 C, Sig=282,8 Ref=360,100

Peak #	RetTime [min]	Type	Width [min]	Area [mAU*s]	Height [mAU]	Area %
1	0.437	BV	0.0746	206.15526	42.49538	3.9195
2	0.710	VV	0.1760	419.88855	31.39880	7.9832
3	0.896	VV	0.2495	480.94238	24.23791	9.1440
4	1.426	VV	0.1935	294.35336	25.34132	5.5964
5	1.637	VV	0.2412	495.25851	27.09988	9.4161
6	1.932	VV	0.2950	528.38104	23.61619	10.0459
7	2.456	VV	0.3756	657.86664	22.73298	12.5077
8	2.849	VV	0.2628	324.63147	17.15285	6.1721
9	3.231	VV	0.5094	595.64349	15.39302	11.3247
10	4.008	VV	0.3644	315.96475	11.80084	6.0073
11	4.334	VV	0.2803	227.66063	10.44310	4.3284
12	5.040	VV	0.6074	438.53506	9.34579	8.3377
13	5.771	VB	0.6033	265.44913	5.67960	5.0469
14	11.803	BB	0.4128	8.94806	2.59139e-1	0.1701

Totals : 5259.67835 266.99680

Signal 4: DAD1 D, Sig=254,16 Ref=360,100

Peak #	RetTime [min]	Type	Width [min]	Area [mAU*s]	Height [mAU]	Area %
1	0.436	BV	0.0623	173.22313	43.61800	9.8690
2	0.647	VV	0.1337	142.22365	14.59979	8.1029
3	0.949	VV	0.1593	145.94382	13.16926	8.3148
4	1.214	VV	0.1108	609.97205	80.13186	34.7517
5	1.465	VV	0.1667	131.35022	10.59002	7.4834
6	1.812	VV	0.2026	115.94130	7.74570	6.6055
7	2.443	VV	0.3278	114.31069	4.90623	6.5126
8	2.863	VB	0.3048	85.38896	3.70632	4.8648
9	4.390	BV	0.4902	46.36963	1.22340	2.6418
10	4.910	VV	0.2564	43.22309	2.46673	2.4625

Peak #	RetTime [min]	Type	Width [min]	Area [mAU*s]	Height [mAU]	Area %
11	5.297	VV	0.3545	74.09493	2.91581	4.2214
12	6.314	VB	0.4296	44.32613	1.47932	2.5254
13	8.527	BB	0.2951	3.52497	1.52826e-1	0.2008
14	26.979	BB	0.7437	25.33595	4.03998e-1	1.4435

Totals : 1755.22852 187.10924

===

*** End of Report ***
