# Supplementary material for: Influence of Silver Nanoparticles (AgNPs) on Vegetative Growth and Concentrations of Nutrients and Phytohormones in Tomato
Source: Plants (Basel). 2026 Jan 28;15(3):405. doi: 10.3390/plants15030405 (PMC12899181; doi:10.3390/plants15030405)
Supplement: Supplementary file 1 [file plants-15-00405-s001.zip › S1. HPLC Analysis (plants-4015186)/cv. Vengador/Leaves/5 ppm/V-5-L-R2.pdf]

Data File C:\CHEM32\...ORMTMG\FITOHOR GABY Y ALE 30-11-2020 2012-10-03 09-08-53\023-2301.D
Sample Name: 5 PPM VENGADOR HOJA R2

===

Acq. Operator : TMG Seq. Line : 23
Acq. Instrument : Instrument 1 Location : Vial 23
Injection Date : 10/3/2012 9:21:58 PM Inj : 1
Inj Volume : 200.0 µl
Different Inj Volume from Sequence ! Actual Inj Volume : 50.0 µl
Acq. Method : C:\CHEM32\1\DATA\FITOHORMTMG\FITOHOR GABY Y ALE 30-11-2020 2012-10-03 09-08-53\FITOHORMONAS DR SOTO.M
Last changed : 8/14/2013 11:13:25 AM by TMG
Analysis Method : C:\CHEM32\1\METHODS\LAVADO COLUMNA ACET.M
Last changed : 10/21/2012 12:24:49 PM by TMG
(modified after loading)

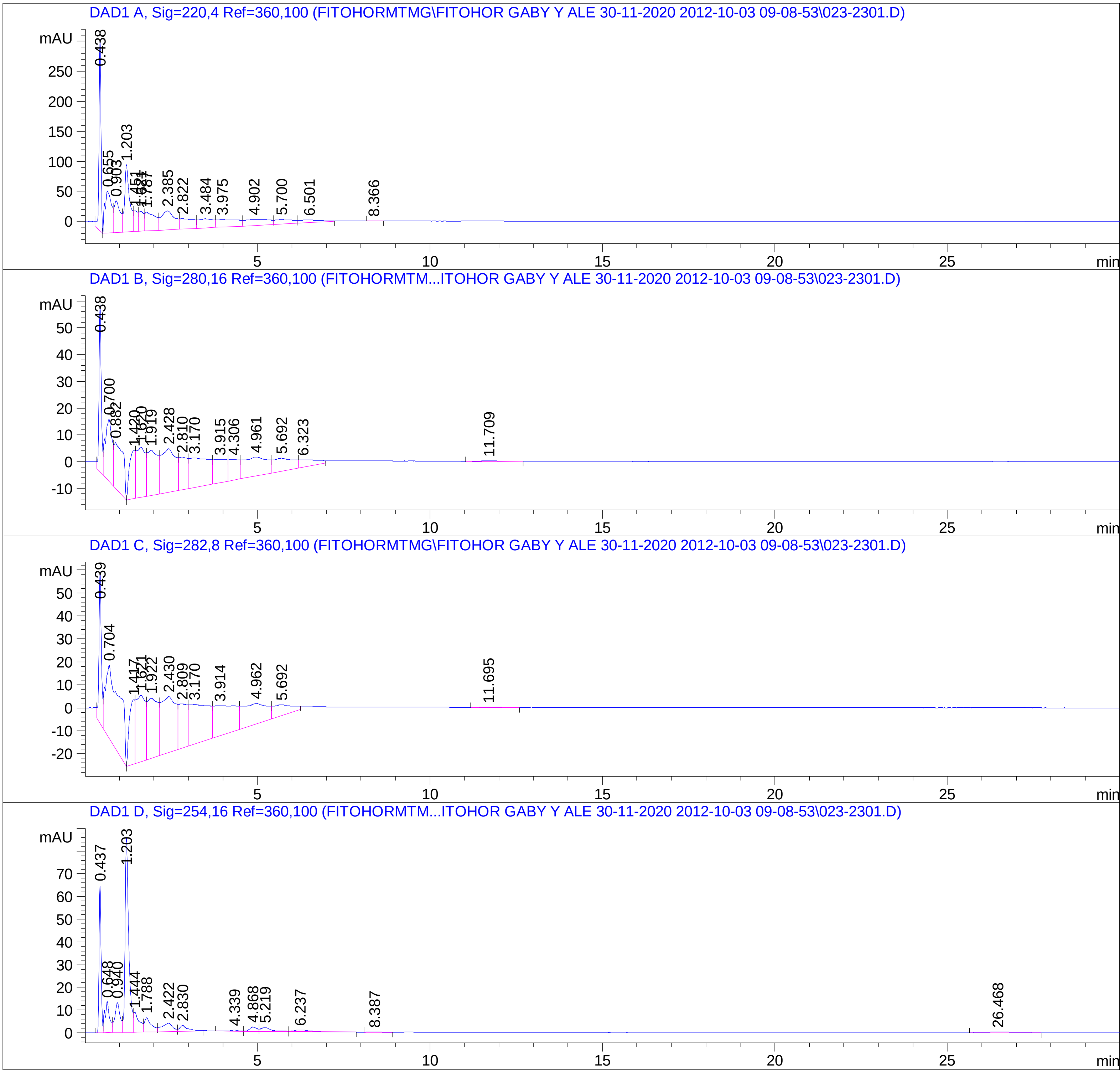

===

Area Percent Report

===

Sorted By : Signal
Multiplier: : 1.0000
Dilution: : 1.0000
Use Multiplier & Dilution Factor with ISTDs

Signal 1: DAD1 A, Sig=220,4 Ref=360,100

Peak #	RetTime [min]	Type	Width [min]	Area [mAU*s]	Height [mAU]	Area %
1	0.438	BV	0.0633	1289.13025	317.81635	14.9813
2	0.655	VV	0.1884	908.23242	69.52773	10.5548
3	0.903	VV	0.1723	649.75940	52.53695	7.5510
4	1.203	VV	0.1454	1154.52100	110.97836	13.4170
5	1.451	VV	0.1111	270.26303	34.59968	3.1408
6	1.621	VV	0.1362	319.18649	33.20410	3.7094
7	1.787	VV	0.2796	678.11212	30.95041	7.8805
8	2.385	VV	0.3824	856.47925	31.38467	9.9534
9	2.822	VV	0.3570	477.11523	17.34199	5.5447
10	3.484	VV	0.4032	460.26999	15.28918	5.3489
11	3.975	VV	0.5544	539.63684	12.43136	6.2713
12	4.902	VV	0.6226	502.55005	10.42066	5.8403
13	5.700	VV	0.5048	282.36789	7.54118	3.2815
14	6.501	VB	0.6039	213.54768	4.48168	2.4817
15	8.366	BB	0.1628	3.73647	3.14111e-1	0.0434

Totals : 8604.90812 748.81841

Signal 2: DAD1 B, Sig=280,16 Ref=360,100

Peak #	RetTime [min]	Type	Width [min]	Area [mAU*s]	Height [mAU]	Area %
1	0.438	BV	0.0714	284.74442	62.21922	7.1433
2	0.700	VV	0.1829	324.31467	23.23288	8.1360
3	0.882	VV	0.2379	323.48663	17.00390	8.1152
4	1.420	VV	0.1927	206.45566	17.87536	5.1793
5	1.620	VV	0.2337	332.19147	18.83879	8.3336
6	1.919	VV	0.2788	354.10846	16.87287	8.8834
7	2.428	VV	0.3817	478.72797	16.24900	12.0097
8	2.810	VV	0.2420	218.67169	12.14824	5.4857
9	3.170	VV	0.5054	422.71164	11.02405	10.6044
10	3.915	VV	0.3330	233.09708	8.74352	5.8476
11	4.306	VV	0.2971	167.10030	7.70326	4.1920
12	4.961	VV	0.6324	342.35306	7.10217	8.5885
13	5.692	VB	0.5317	192.62326	4.84628	4.8323

Peak #	RetTime [min]	Type	Width [min]	Area [mAU*s]	Height [mAU]	Area %
14	6.323	BB	0.4207	96.74551	2.78873	2.4270
15	11.709	BB	0.4335	8.85677	2.70882e-1	0.2222

Totals : 3986.18858 226.91913

Signal 3: DAD1 C, Sig=282,8 Ref=360,100

Peak #	RetTime [min]	Type	Width [min]	Area [mAU*s]	Height [mAU]	Area %
1	0.439	BV	0.0745	319.97815	66.06765	5.6585
2	0.704	VV	0.3696	948.13373	31.82517	16.7668
3	1.417	VV	0.1836	305.79483	27.96824	5.4077
4	1.621	VV	0.2520	550.71851	28.94522	9.7389
5	1.922	VV	0.2884	566.65381	26.19667	10.0207
6	2.430	VV	0.3848	723.51355	24.34312	12.7946
7	2.809	VV	0.2499	354.10873	19.31826	6.2621
8	3.170	VV	0.4869	656.87433	17.30309	11.6162
9	3.914	VV	0.5520	572.70093	13.15376	10.1277
10	4.962	VV	0.6680	459.88528	8.94247	8.1326
11	5.692	VB	0.5201	186.45929	4.81148	3.2974
12	11.695	BB	0.4152	9.99727	2.96659e-1	0.1768

Totals : 5654.81841 269.17179

Signal 4: DAD1 D, Sig=254,16 Ref=360,100

Peak #	RetTime [min]	Type	Width [min]	Area [mAU*s]	Height [mAU]	Area %
1	0.437	BV	0.0659	266.17096	64.81876	16.4314
2	0.648	VV	0.1317	129.21378	13.49689	7.9767
3	0.940	VV	0.1543	138.94661	13.04554	8.5775
4	1.203	VV	0.1104	648.15894	85.56290	40.0125
5	1.444	VV	0.1466	96.48476	8.75483	5.9562
6	1.788	VV	0.1953	90.03080	6.27675	5.5578
7	2.422	VV	0.3216	82.83956	3.69510	5.1139
8	2.830	VB	0.2448	47.25429	2.64088	2.9171
9	4.339	BV	0.2689	8.66530	4.57617e-1	0.5349
10	4.868	VV	0.2246	28.21403	1.90656	1.7417
11	5.219	VV	0.3062	37.85115	1.76483	2.3366
12	6.237	VB	0.4175	23.60869	8.11227e-1	1.4574
13	8.387	BB	0.2460	2.05633	1.05227e-1	0.1269
14	26.468	BB	0.7594	20.39628	3.19227e-1	1.2591

Totals : 1619.89148 203.65634

==

*** End of Report ***
