# Supplementary material for: Influence of Silver Nanoparticles (AgNPs) on Vegetative Growth and Concentrations of Nutrients and Phytohormones in Tomato
Source: Plants (Basel). 2026 Jan 28;15(3):405. doi: 10.3390/plants15030405 (PMC12899181; doi:10.3390/plants15030405)
Supplement: Supplementary file 1 [file plants-15-00405-s001.zip › S1. HPLC Analysis (plants-4015186)/cv. Vengador/Leaves/5 ppm/V-5-L-R3.pdf]

```
=====================================================================
Acq. Operator   : TMG                          Seq. Line :  24
Acq. Instrument : Instrument 1                  Location : Vial 24
Injection Date  : 10/3/2012 9:54:04 PM               Inj :   1
                                              Inj Volume : 200.0 µl
Different Inj Volume from Sequence !   Actual Inj Volume : 50.0 µl
Acq. Method     : C:\CHEM32\1\DATA\FITOHORMTMG\FITOHOR GABY Y ALE 30-11-2020 2012-10-03 09-08-
                  53\FITOHORMONAS DR SOTO.M
Last changed    : 8/14/2013 11:13:25 AM by TMG
Analysis Method : C:\CHEM32\1\METHODS\LAVADO COLUMNA ACET.M
Last changed    : 10/21/2012 12:24:49 PM by TMG
                  (modified after loading)
```

DAD1 A, Sig=220,4 Ref=360,100 (FITOHORMTMG\FITOHOR GABY Y ALE 30-11-2020 2012-10-03 09-08-53\024-2401.D)

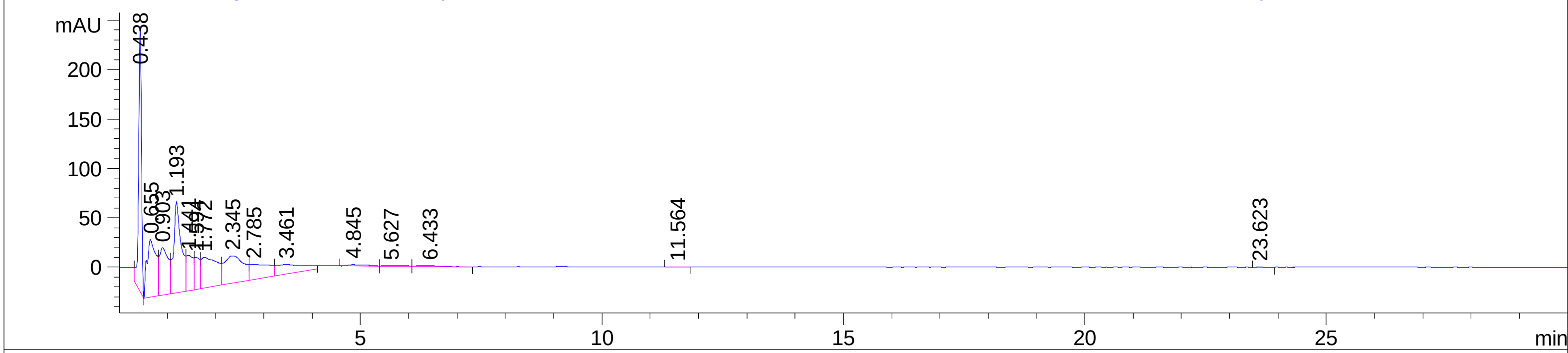

DAD1 B, Sig=280,16 Ref=360,100 (FITOHORMTM...ITOHOR GABY Y ALE 30-11-2020 2012-10-03 09-08-53\024-2401.D)

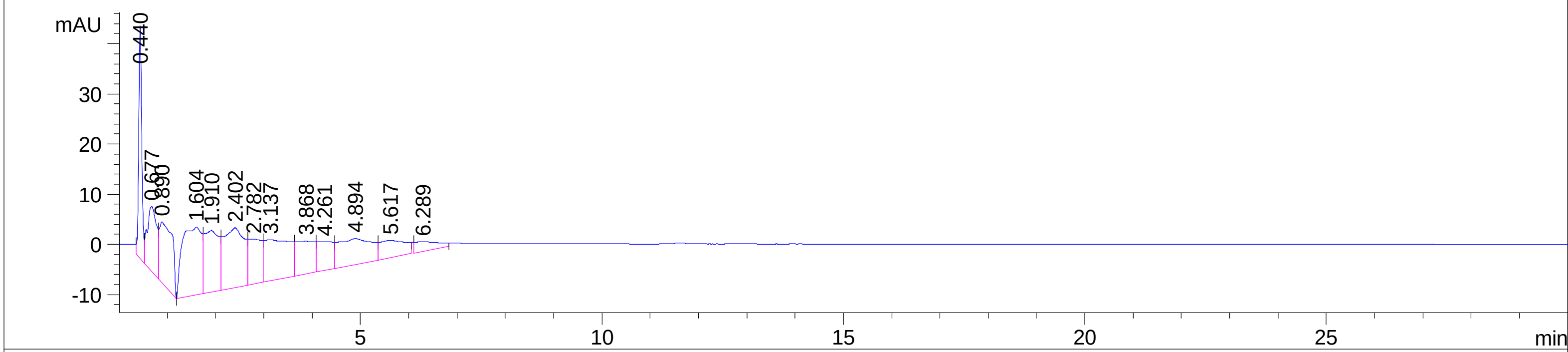

DAD1 C, Sig=282,8 Ref=360,100 (FITOHORMTMG\FITOHOR GABY Y ALE 30-11-2020 2012-10-03 09-08-53\024-2401.D)

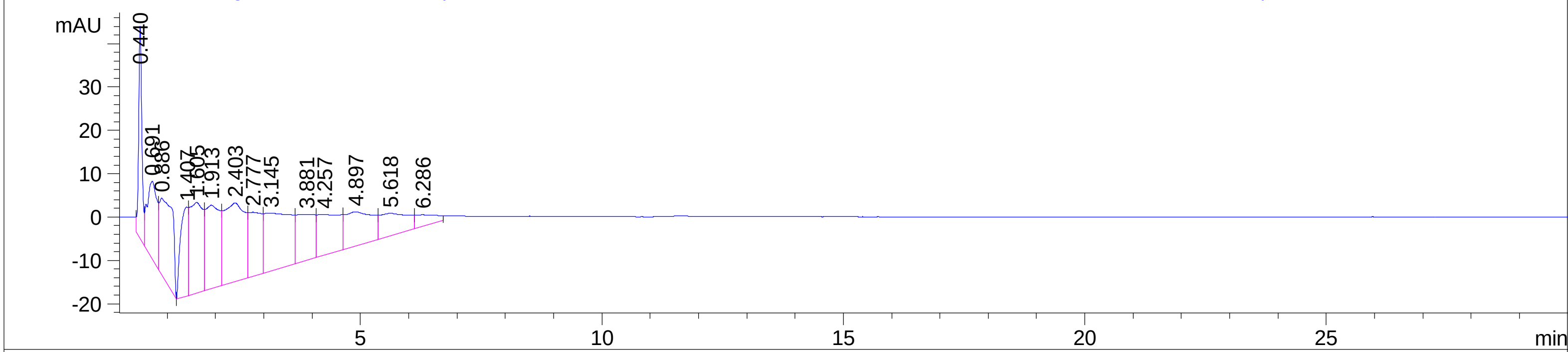

DAD1 D, Sig=254,16 Ref=360,100 (FITOHORMTM...ITOHOR GABY Y ALE 30-11-2020 2012-10-03 09-08-53\024-2401.D)

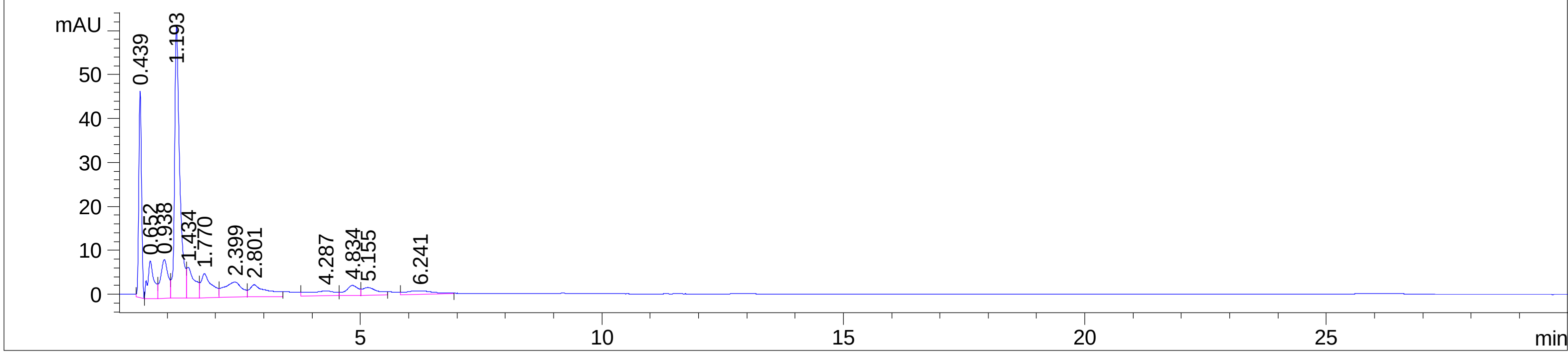

===

Area Percent Report

===

Sorted By : Signal
Multiplier: : 1.0000
Dilution: : 1.0000
Use Multiplier & Dilution Factor with ISTDs

Signal 1: DAD1 A, Sig=220,4 Ref=360,100

Peak #	RetTime [min]	Type	Width [min]	Area [mAU*s]	Height [mAU]	Area %
1	0.438	BV	0.0644	1105.57422	266.45029	17.0526
2	0.655	VV	0.1691	745.91974	58.36875	11.5052
3	0.903	VV	0.1870	635.76471	47.84431	9.8061
4	1.193	VV	0.1548	1023.65729	91.28444	15.7891
5	1.441	VV	0.1314	341.52850	35.75980	5.2678
6	1.594	VV	0.1104	249.97412	32.26667	3.8556
7	1.772	VV	0.2890	709.56561	31.24054	10.9445
8	2.345	VV	0.3924	772.47083	27.78506	11.9147
9	2.785	VV	0.3554	431.30905	15.56155	6.6526
10	3.461	VB	0.4981	377.81464	9.79301	5.8275
11	4.845	BV	0.3382	34.91053	1.42317	0.5385
12	5.627	VV	0.3594	22.71475	9.09290e-1	0.3504
13	6.433	VV	0.3719	23.12827	7.93931e-1	0.3567
14	11.564	BB	0.2912	6.47655	2.78634e-1	0.0999
15	23.623	VB	0.1589	2.52458	2.56250e-1	0.0389

Totals : 6483.33338 620.01570

Signal 2: DAD1 B, Sig=280,16 Ref=360,100

Peak #	RetTime [min]	Type	Width [min]	Area [mAU*s]	Height [mAU]	Area %
1	0.440	BV	0.0695	205.06325	46.46358	7.3107
2	0.677	VV	0.1960	174.60068	12.89163	6.2247
3	0.890	VV	0.2436	233.98294	12.09858	8.3417
4	1.604	VV	0.3552	378.74033	13.50504	13.5024
5	1.910	VV	0.2785	256.96155	12.26103	9.1609
6	2.402	VV	0.3850	353.43939	11.88163	12.6004
7	2.782	VV	0.2532	163.98668	8.97239	5.8463
8	3.137	VV	0.4804	298.68008	8.13419	10.6482
9	3.868	VV	0.3369	172.63487	6.47806	6.1546
10	4.261	VV	0.2943	129.78596	5.68502	4.6270
11	4.894	VV	0.6159	245.71436	5.13855	8.7599
12	5.617	VB	0.4897	127.56171	3.44764	4.5477
13	6.289	BB	0.4149	63.83398	1.91512	2.2757

Peak #	RetTime [min]	Type	Width [min]	Area [mAU*s]	Height [mAU]	Area %
Totals :				2804.98577	148.87246	

Signal 3: DAD1 C, Sig=282,8 Ref=360,100

Peak #	RetTime [min]	Type	Width [min]	Area [mAU*s]	Height [mAU]	Area %
1	0.440	BV	0.0726	229.79964	49.07496	5.2804
2	0.691	VV	0.2036	253.25168	17.84645	5.8192
3	0.886	VV	0.2554	357.80627	17.58376	8.2217
4	1.407	VV	0.1848	222.76561	20.48918	5.1187
5	1.605	VV	0.2485	392.45227	20.95213	9.0178
6	1.913	VV	0.2807	404.11163	19.26462	9.2857
7	2.403	VV	0.3893	548.15479	18.20251	12.5956
8	2.777	VV	0.2565	274.40585	14.78555	6.3053
9	3.145	VV	0.4847	499.42923	13.40443	11.4759
10	3.881	VV	0.3372	284.75024	10.67447	6.5430
11	4.257	VB	0.3934	292.80862	9.34957	6.7282
12	4.897	BV	0.5194	313.39719	7.92372	7.2013
13	5.618	VV	0.5176	203.76059	5.17179	4.6820
14	6.286	VB	0.3566	75.07320	2.63308	1.7250
Totals :				4351.96681	227.35620	

Signal 4: DAD1 D, Sig=254,16 Ref=360,100

Peak #	RetTime [min]	Type	Width [min]	Area [mAU*s]	Height [mAU]	Area %
1	0.439	BV	0.0643	186.64293	47.04562	14.2864
2	0.652	VV	0.1252	77.39664	8.57998	5.9242
3	0.938	VV	0.1554	95.49031	8.88087	7.3092
4	1.193	VV	0.1090	462.62482	62.03508	35.4111
5	1.434	VV	0.1545	81.04211	7.02830	6.2033
6	1.770	VV	0.2119	86.11167	5.46384	6.5913
7	2.399	VV	0.3589	88.38223	3.45002	6.7651
8	2.801	VB	0.3478	73.86589	2.76353	5.6540
9	4.287	BV	0.5028	43.80873	1.12378	3.3533
10	4.834	VV	0.2612	40.69204	2.24766	3.1147
11	5.155	VB	0.3157	38.50224	1.72955	2.9471
12	6.241	BB	0.5188	31.87988	8.00158e-1	2.4402
Totals :				1306.43949	151.14839	

==
