# Supplementary material for: Influence of Silver Nanoparticles (AgNPs) on Vegetative Growth and Concentrations of Nutrients and Phytohormones in Tomato
Source: Plants (Basel). 2026 Jan 28;15(3):405. doi: 10.3390/plants15030405 (PMC12899181; doi:10.3390/plants15030405)
Supplement: Supplementary file 1 [file plants-15-00405-s001.zip › S1. HPLC Analysis (plants-4015186)/cv. Vengador/Leaves/Control/V-T-L-R1.pdf]

===

Acq. Operator : TMG   Seq. Line : 10
Acq. Instrument : Instrument 1   Location : Vial 10
Injection Date : 10/3/2012 2:24:26 PM   Inj : 1
Inj Volume : 200.0 µl
Different Inj Volume from Sequence !   Actual Inj Volume : 50.0 µl
Acq. Method : C:\CHEM32\1\DATA\FITOHORMTMG\FITOHOR GABY Y ALE 30-11-2020 2012-10-03 09-08-53\FITOHORMONAS DR SOTO.M
Last changed : 8/14/2013 11:13:25 AM by TMG
Analysis Method : C:\CHEM32\1\METHODS\LAVADO COLUMNA ACET.M
Last changed : 10/21/2012 12:24:49 PM by TMG
(modified after loading)
Additional Info : Peak(s) manually integrated

DAD1 A, Sig=220,4 Ref=360,100 (FITOHORMTMG\FITOHOR GABY Y ALE 30-11-2020 2012-10-03 09-08-53\010-1001.D)

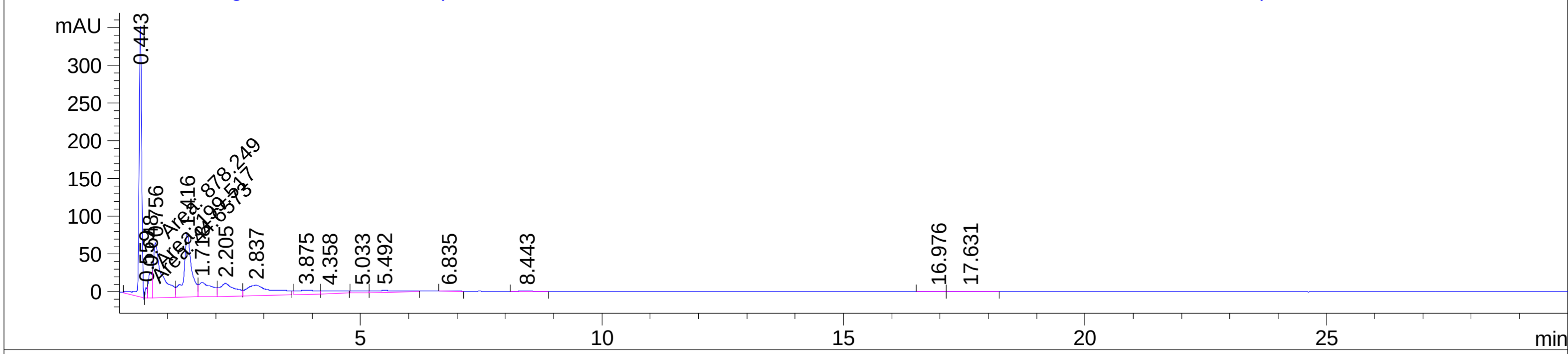

DAD1 B, Sig=280,16 Ref=360,100 (FITOHORMTM...ITOHOR GABY Y ALE 30-11-2020 2012-10-03 09-08-53\010-1001.D)

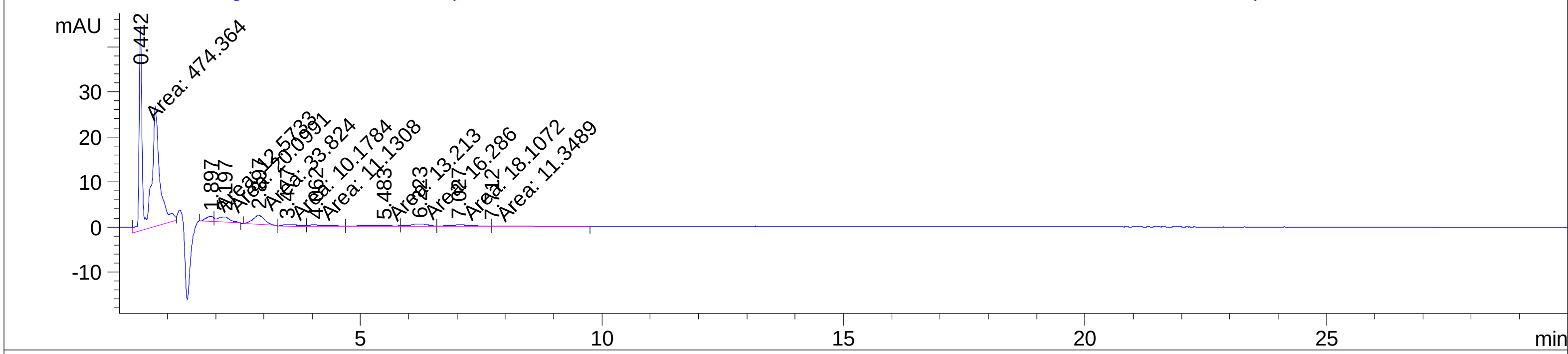

DAD1 C, Sig=282,8 Ref=360,100 (FITOHORMTMG\FITOHOR GABY Y ALE 30-11-2020 2012-10-03 09-08-53\010-1001.D)

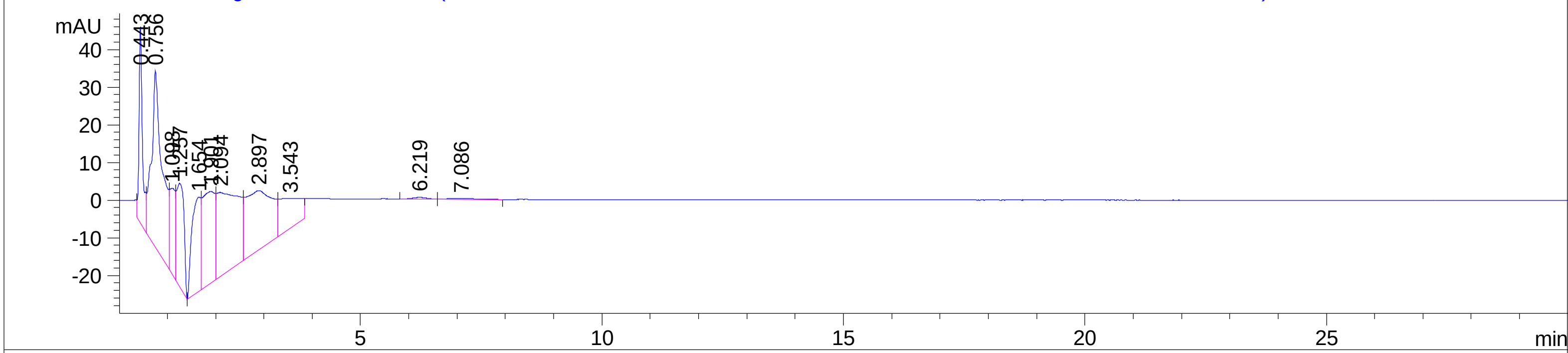

DAD1 D, Sig=254,16 Ref=360,100 (FITOHORMTM...ITOHOR GABY Y ALE 30-11-2020 2012-10-03 09-08-53\010-1001.D)

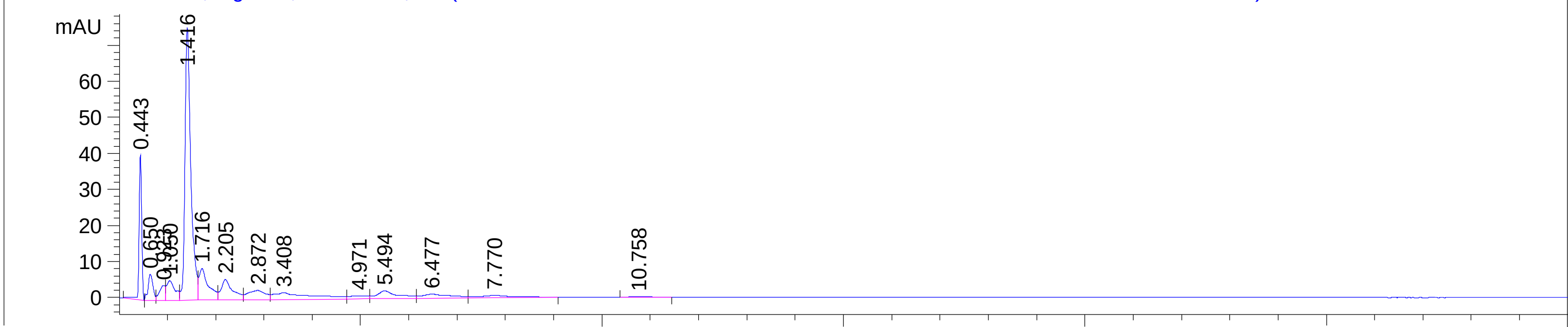

===

Area Percent Report

===

Sorted By : Signal
Multiplier: : 1.0000
Dilution: : 1.0000
Use Multiplier & Dilution Factor with ISTDs

Signal 1: DAD1 A, Sig=220,4 Ref=360,100

Peak #	RetTime [min]	Type	Width [min]	Area [mAU*s]	Height [mAU]	Area %
1	0.443	BV	0.0604	1342.80859	353.13016	26.2956
2	0.559	MF	0.0522	44.65729	14.25977	0.8745
3	0.648	FM	0.0972	199.51749	34.20354	3.9071
4	0.756	FM	0.2008	878.24921	72.90657	17.1984
5	1.416	VV	0.1587	953.00964	85.09631	18.6624
6	1.718	VV	0.2428	356.44400	19.00714	6.9801
7	2.205	VV	0.2949	373.95633	16.85166	7.3230
8	2.837	VB	0.5286	503.31744	13.32856	9.8562
9	3.875	BV	0.3861	155.88695	5.37873	3.0527
10	4.358	VB	0.4088	121.68861	3.80786	2.3830
11	5.033	BV	0.3139	60.91402	2.59476	1.1929
12	5.492	VB	0.5177	91.53729	2.37496	1.7925
13	6.835	BB	0.2289	4.57992	2.88918e-1	0.0897
14	8.443	BB	0.2944	7.59011	3.53710e-1	0.1486
15	16.976	VV	0.2553	5.30172	2.76994e-1	0.1038
16	17.631	VB	0.3585	7.12334	2.60916e-1	0.1395

Totals : 5106.58195 624.12056

Signal 2: DAD1 B, Sig=280,16 Ref=360,100

Peak #	RetTime [min]	Type	Width [min]	Area [mAU*s]	Height [mAU]	Area %
1	0.442	MM	0.1736	474.36380	45.54931	76.3718
2	1.897	MF	0.1744	12.57332	1.20125	2.0243
3	2.197	FM	0.3009	20.09912	1.11344	3.2359
4	2.897	MM	0.2801	33.82404	2.01255	5.4456
5	3.477	MF	0.4744	10.17837	3.57621e-1	1.6387
6	4.062	MF	0.5931	11.13082	3.12776e-1	1.7920
7	5.483	MF	0.7833	13.21302	2.81149e-1	2.1273
8	6.223	MF	0.4603	16.28603	5.89711e-1	2.6220
9	7.027	MF	0.8115	18.10721	3.71895e-1	2.9152
10	7.712	FM	1.3363	11.34893	1.41549e-1	1.8272

Totals : 621.12466 51.93125

Signal 3: DAD1 C, Sig=282,8 Ref=360,100

Peak #	RetTime [min]	Type	Width [min]	Area [mAU*s]	Height [mAU]	Area %
1	0.443	BV	0.0744	260.84586	52.13659	6.8665
2	0.756	VV	0.1995	714.86554	46.98207	18.8182
3	1.098	VV	0.1173	186.96608	22.90042	4.9217
4	1.257	VV	0.1735	305.09998	27.49665	8.0315
5	1.654	VV	0.2128	319.29196	25.04165	8.4051
6	1.901	VV	0.2443	443.49661	24.36958	11.6747
7	2.094	VV	0.3808	685.94128	22.43090	18.0568
8	2.897	VV	0.5033	605.87439	15.80403	15.9491
9	3.543	VV	0.3873	256.94379	8.02985	6.7638
10	6.219	BV	0.3007	9.84123	4.72991e-1	0.2591
11	7.086	VB	0.4517	9.63066	2.56726e-1	0.2535
Totals :				3798.79738	245.92146	

Signal 4: DAD1 D, Sig=254,16 Ref=360,100

Peak #	RetTime [min]	Type	Width [min]	Area [mAU*s]	Height [mAU]	Area %
1	0.443	BV	0.0603	152.42502	40.16671	10.1864
2	0.650	VV	0.1117	52.46880	7.31007	3.5064
3	0.923	VV	0.1193	33.77940	4.13380	2.2575
4	1.050	VV	0.1790	68.36701	5.42490	4.5689
5	1.416	VV	0.1270	641.85211	75.65681	42.8945
6	1.716	VV	0.1831	118.04458	8.76357	7.8888
7	2.205	VV	0.2302	93.93898	5.64404	6.2779
8	2.872	VV	0.3487	65.35981	2.53610	4.3679
9	3.408	VV	0.6803	102.18398	1.87938	6.8289
10	4.971	VV	0.3394	21.48278	8.54089e-1	1.4357
11	5.494	VV	0.4083	64.16258	2.14909	4.2879
12	6.477	VV	0.5624	46.01525	1.10831	3.0752
13	7.770	VB	0.6661	31.86802	6.01536e-1	2.1297
14	10.758	BB	0.3728	4.40287	1.42379e-1	0.2942
Totals :				1496.35120	156.37079	

===

*** End of Report ***
