# Supplementary material for: Influence of Silver Nanoparticles (AgNPs) on Vegetative Growth and Concentrations of Nutrients and Phytohormones in Tomato
Source: Plants (Basel). 2026 Jan 28;15(3):405. doi: 10.3390/plants15030405 (PMC12899181; doi:10.3390/plants15030405)
Supplement: Supplementary file 1 [file plants-15-00405-s001.zip › S1. HPLC Analysis (plants-4015186)/cv. Vengador/Leaves/Control/V-T-L-R2.pdf]

Data File C:\CHEM32\...ORMTMG\FITOHOR GABY Y ALE 30-11-2020 2012-10-03 09-08-53\011-1101.D
Sample Name: TESTIGO VENGADOR HOJA R2

===

Acq. Operator : TMG   Seq. Line : 11
Acq. Instrument : Instrument 1   Location : Vial 11
Injection Date : 10/3/2012 2:56:36 PM   Inj : 1
Inj Volume : 200.0 µl
Different Inj Volume from Sequence !   Actual Inj Volume : 50.0 µl
Acq. Method : C:\CHEM32\1\DATA\FITOHORMTMG\FITOHOR GABY Y ALE 30-11-2020 2012-10-03 09-08-53\FITOHORMONAS DR SOTO.M
Last changed : 8/14/2013 11:13:25 AM by TMG
Analysis Method : C:\CHEM32\1\METHODS\LAVADO COLUMNA ACET.M
Last changed : 10/21/2012 12:24:49 PM by TMG
(modified after loading)

DAD1 A, Sig=220,4 Ref=360,100 (FITOHORMTMG\FITOHOR GABY Y ALE 30-11-2020 2012-10-03 09-08-53\011-1101.D)

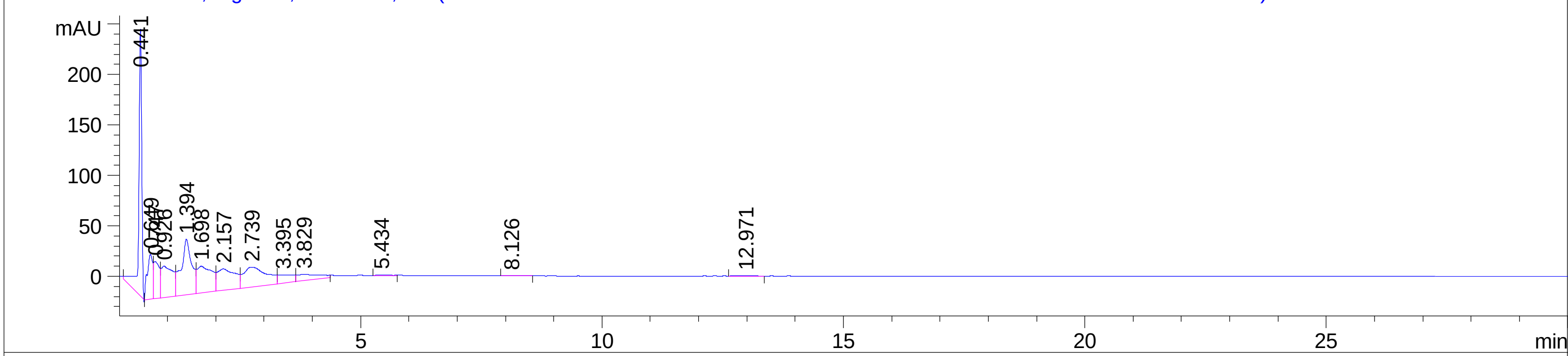

DAD1 B, Sig=280,16 Ref=360,100 (FITOHORMTM...ITOHOR GABY Y ALE 30-11-2020 2012-10-03 09-08-53\011-1101.D)

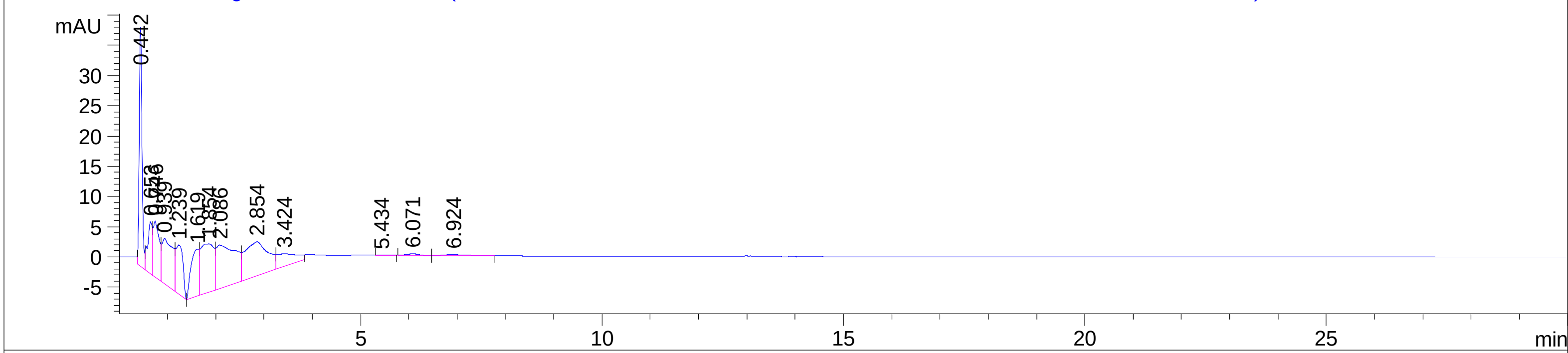

DAD1 C, Sig=282,8 Ref=360,100 (FITOHORMTMG\FITOHOR GABY Y ALE 30-11-2020 2012-10-03 09-08-53\011-1101.D)

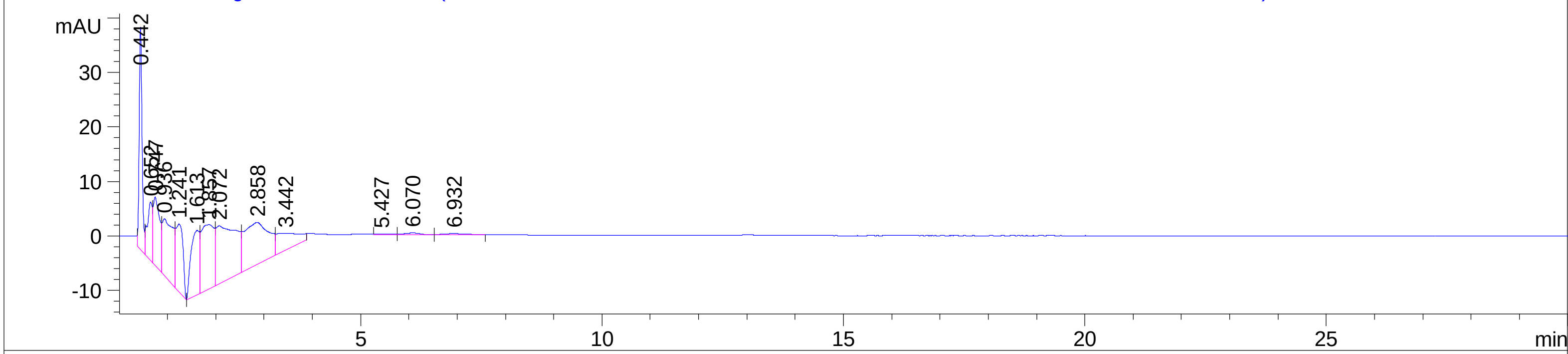

DAD1 D, Sig=254,16 Ref=360,100 (FITOHORMTM...ITOHOR GABY Y ALE 30-11-2020 2012-10-03 09-08-53\011-1101.D)

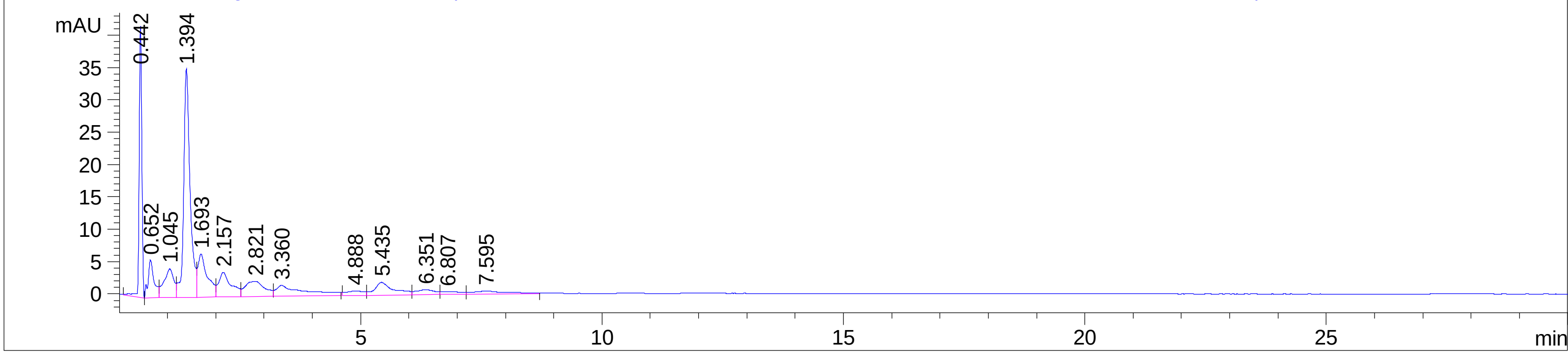

===

Area Percent Report

===

Sorted By : Signal
Multiplier: : 1.0000
Dilution: : 1.0000
Use Multiplier & Dilution Factor with ISTDs

Signal 1: DAD1 A, Sig=220,4 Ref=360,100

Peak #	RetTime [min]	Type	Width [min]	Area [mAU*s]	Height [mAU]	Area %
1	0.441	BV	0.0668	1137.89844	261.28418	21.5707
2	0.649	VV	0.1135	354.93744	44.28634	6.7284
3	0.747	VV	0.1133	293.83325	36.74982	5.5701
4	0.926	VV	0.2106	506.20731	30.99073	9.5960
5	1.394	VV	0.2074	844.33490	54.89618	16.0058
6	1.698	VV	0.2767	563.29974	26.42631	10.6783
7	2.157	VV	0.3387	541.32275	21.14268	10.2617
8	2.739	VV	0.4716	647.81799	19.49695	12.2805
9	3.395	VV	0.2861	171.45625	7.99733	3.2502
10	3.829	VB	0.4168	189.47488	6.19531	3.5918
11	5.434	BB	0.2058	9.98453	6.85825e-1	0.1893
12	8.126	BB	0.3146	8.03413	3.13950e-1	0.1523
13	12.971	BB	0.2470	6.59512	3.57850e-1	0.1250

Totals : 5275.19674 510.82346

Signal 2: DAD1 B, Sig=280,16 Ref=360,100

Peak #	RetTime [min]	Type	Width [min]	Area [mAU*s]	Height [mAU]	Area %
1	0.442	BV	0.0628	159.24478	39.66542	13.1664
2	0.653	VV	0.1034	61.19445	8.55738	5.0596
3	0.746	VV	0.1217	80.10759	9.19107	6.6233
4	0.939	VV	0.2076	120.67048	7.50277	9.9770
5	1.239	VV	0.1625	84.15339	8.14829	6.9578
6	1.619	VV	0.1845	84.52013	7.79225	6.9881
7	1.854	VV	0.2538	154.97504	8.00584	12.8134
8	2.086	VV	0.3856	198.73694	7.16439	16.4316
9	2.854	VV	0.4378	188.64165	5.61739	15.5969
10	3.424	VB	0.3976	59.24778	2.05973	4.8986
11	5.434	BB	0.2556	2.82049	1.55501e-1	0.2332
12	6.071	BV	0.2951	7.07976	3.04729e-1	0.5854
13	6.924	VB	0.4190	8.08818	2.31838e-1	0.6687

Totals : 1209.48065 104.39659

Signal 3: DAD1 C, Sig=282,8 Ref=360,100

Peak #	RetTime [min]	Type	Width [min]	Area [mAU*s]	Height [mAU]	Area %
1	0.442	BV	0.0645	170.77563	41.05861	9.9221
2	0.652	VV	0.1022	77.99804	10.80209	4.5317
3	0.747	VV	0.1291	118.44478	12.66405	6.8817
4	0.936	VV	0.2137	174.15286	10.49034	10.1183
5	1.241	VV	0.1638	130.06255	12.45390	7.5567
6	1.613	VV	0.1967	140.40698	11.80900	8.1577
7	1.857	VV	0.2451	217.29028	11.78808	12.6246
8	2.072	VV	0.3597	302.89529	10.58925	17.5983
9	2.858	VV	0.4629	273.79587	7.73587	15.9076
10	3.442	VB	0.3894	97.02754	3.13226	5.6373
11	5.427	BB	0.2744	2.81614	1.34455e-1	0.1636
12	6.070	BV	0.2991	7.62145	3.35464e-1	0.4428
13	6.932	VB	0.4083	7.87363	2.31779e-1	0.4575
Totals :				1721.16105	133.22515	

Signal 4: DAD1 D, Sig=254,16 Ref=360,100

Peak #	RetTime [min]	Type	Width [min]	Area [mAU*s]	Height [mAU]	Area %
1	0.442	BV	0.0606	160.33279	41.97288	15.5595
2	0.652	VV	0.1259	50.93378	5.83314	4.9429
3	1.045	VV	0.2031	63.50184	4.43468	6.1625
4	1.394	VV	0.1358	326.08475	35.28962	31.6448
5	1.693	VV	0.1916	94.22427	6.71884	9.1440
6	2.157	VV	0.2514	69.92954	3.82309	6.7863
7	2.821	VV	0.3803	67.39269	2.35207	6.5401
8	3.360	VB	0.5420	72.04853	1.68794	6.9919
9	4.888	BV	0.3753	17.92312	6.89520e-1	1.7393
10	5.435	VV	0.3799	54.17532	1.97569	5.2574
11	6.351	VV	0.3795	20.84757	7.47321e-1	2.0231
12	6.807	VV	0.3682	12.47511	4.63212e-1	1.2106
13	7.595	VB	0.5666	20.58194	4.34506e-1	1.9974
Totals :				1030.45126	106.42251	

===

*** End of Report ***
