# Supplementary material for: Influence of Silver Nanoparticles (AgNPs) on Vegetative Growth and Concentrations of Nutrients and Phytohormones in Tomato
Source: Plants (Basel). 2026 Jan 28;15(3):405. doi: 10.3390/plants15030405 (PMC12899181; doi:10.3390/plants15030405)
Supplement: Supplementary file 1 [file plants-15-00405-s001.zip › S1. HPLC Analysis (plants-4015186)/cv. Vengador/Leaves/Control/V-T-L-R3.pdf]

Data File C:\CHEM32\...ORMTMG\FITOHOR GABY Y ALE 30-11-2020 2012-10-03 09-08-53\012-1201.D
Sample Name: TESTIGO VENGADOR HOJA R2

===

Acq. Operator	: TMG	Seq. Line :	12
Acq. Instrument	: Instrument 1	Location :	Vial 12
Injection Date	: 10/3/2012 3:28:44 PM	Inj :	1
		Inj Volume :	200.0 µl
Different Inj Volume from Sequence !		Actual Inj Volume :	50.0 µl

Acq. Method : C:\CHEM32\1\DATA\FITOHORMTMG\FITOHOR GABY Y ALE 30-11-2020 2012-10-03 09-08-53\FITOHORMONAS DR SOTO.M
Last changed : 8/14/2013 11:13:25 AM by TMG
Analysis Method : C:\CHEM32\1\METHODS\LAVADO COLUMNA ACET.M
Last changed : 10/21/2012 12:24:49 PM by TMG
(modified after loading)

DAD1 A, Sig=220,4 Ref=360,100 (FITOHORMTMG\FITOHOR GABY Y ALE 30-11-2020 2012-10-03 09-08-53\012-1201.D)

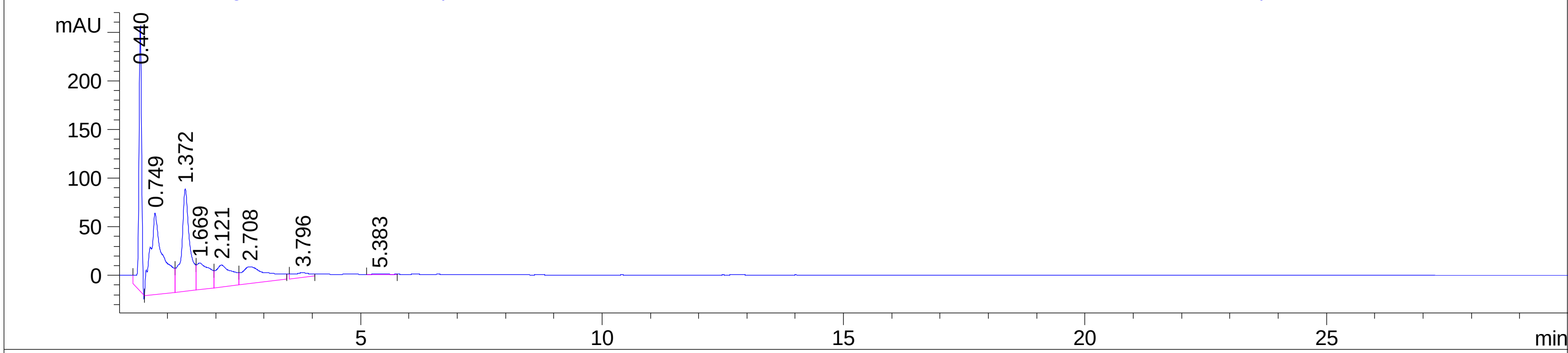

DAD1 B, Sig=280,16 Ref=360,100 (FITOHORMTM...ITOHOR GABY Y ALE 30-11-2020 2012-10-03 09-08-53\012-1201.D)

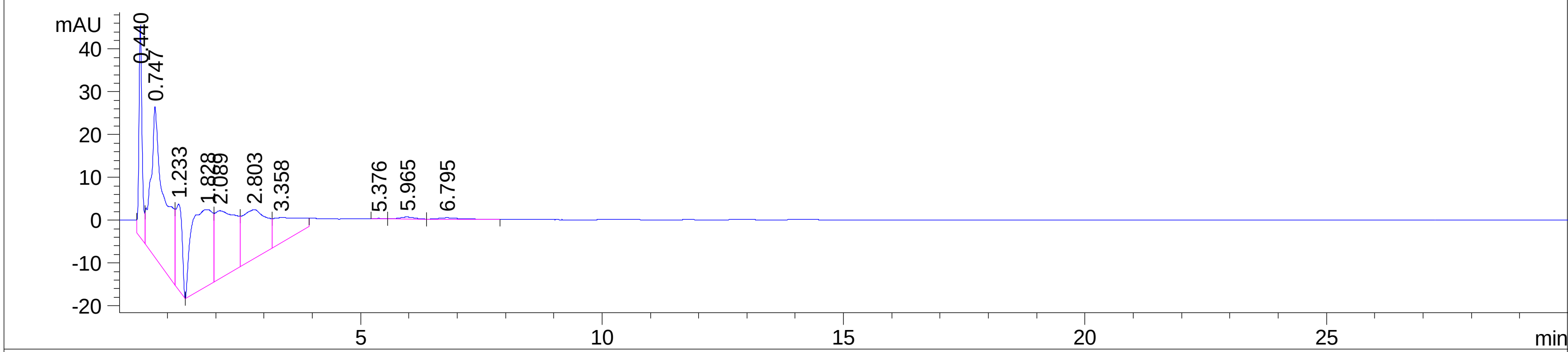

DAD1 C, Sig=282,8 Ref=360,100 (FITOHORMTMG\FITOHOR GABY Y ALE 30-11-2020 2012-10-03 09-08-53\012-1201.D)

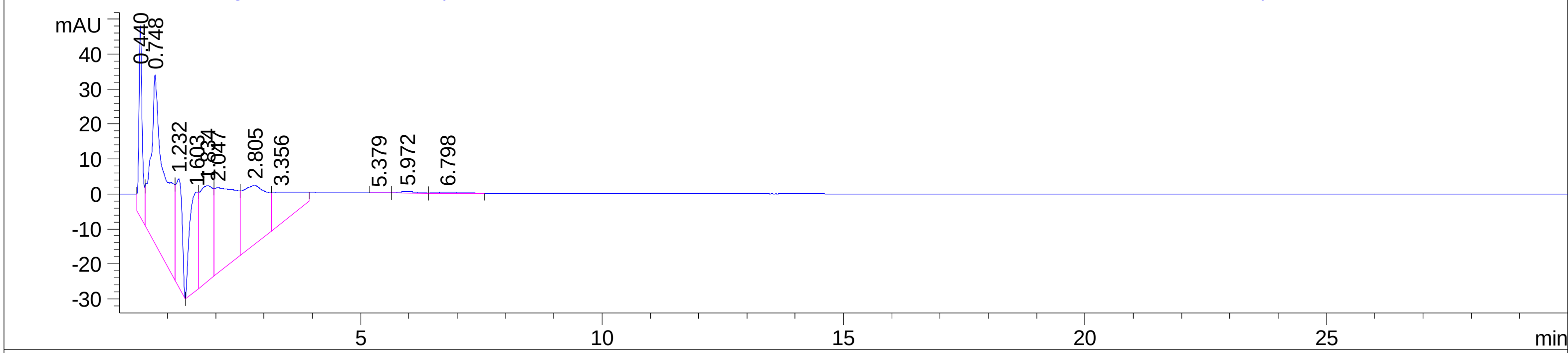

DAD1 D, Sig=254,16 Ref=360,100 (FITOHORMTM...ITOHOR GABY Y ALE 30-11-2020 2012-10-03 09-08-53\012-1201.D)

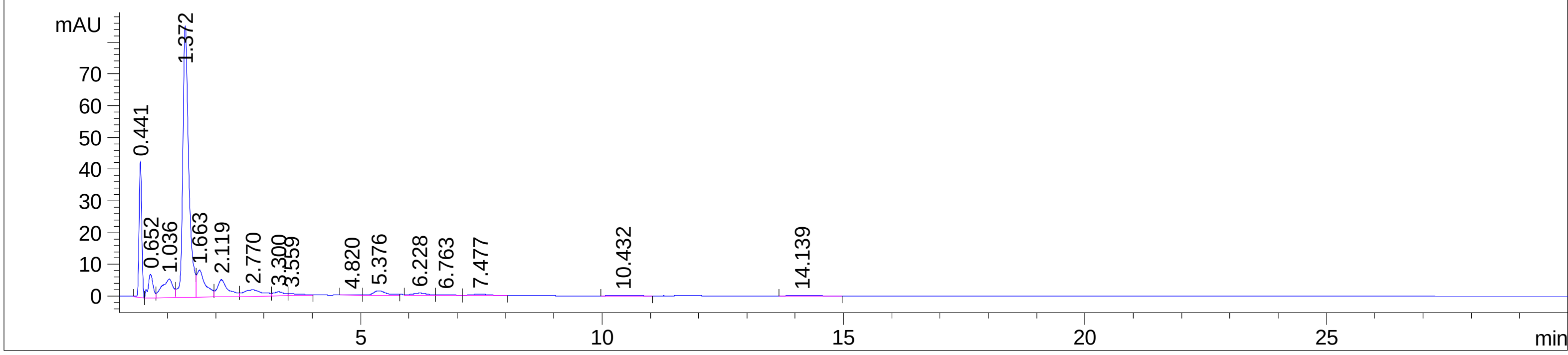

===

Area Percent Report

===

Sorted By : Signal
Multiplier: : 1.0000
Dilution: : 1.0000
Use Multiplier & Dilution Factor with ISTDs

Signal 1: DAD1 A, Sig=220,4 Ref=360,100

Peak #	RetTime [min]	Type	Width [min]	Area [mAU*s]	Height [mAU]	Area %
1	0.440	BV	0.0625	1075.21399	269.85278	18.7191
2	0.749	VV	0.2362	1544.98401	83.37946	26.8976
3	1.372	VV	0.1665	1284.93970	105.20793	22.3703
4	1.669	VV	0.2489	515.37329	27.22231	8.9725
5	2.121	VV	0.3160	528.98315	22.35902	9.2094
6	2.708	VB	0.5101	647.54419	17.40494	11.2735
7	3.796	BB	0.3478	133.55165	4.99645	2.3251
8	5.383	BB	0.2479	13.35897	7.79660e-1	0.2326
Totals :				5743.94895	531.20255	

Signal 2: DAD1 B, Sig=280,16 Ref=360,100

Peak #	RetTime [min]	Type	Width [min]	Area [mAU*s]	Height [mAU]	Area %
1	0.440	BV	0.0683	222.37343	49.59484	8.0811
2	0.747	VV	0.2536	699.95709	35.26695	25.4366
3	1.233	VV	0.1410	179.84392	19.97844	6.5356
4	1.828	VV	0.4250	558.31232	17.84317	20.2892
5	2.089	VV	0.3948	457.42953	15.84489	16.6231
6	2.803	VV	0.4544	403.63251	11.41742	14.6681
7	3.358	VB	0.4615	206.80157	5.86323	7.5152
8	5.376	BV	0.1786	2.07299	1.48707e-1	0.0753
9	5.965	VV	0.3285	10.79090	4.83241e-1	0.3921
10	6.795	VB	0.4189	10.56211	3.45234e-1	0.3838
Totals :				2751.77638	156.78612	

Signal 3: DAD1 C, Sig=282,8 Ref=360,100

Peak #	RetTime [min]	Type	Width [min]	Area [mAU*s]	Height [mAU]	Area %
1	0.440	BV	0.0716	261.23987	54.80286	6.4352
2	0.748	VV	0.2634	1000.73456	48.37412	24.6513
3	1.232	VV	0.1428	281.59567	30.76162	6.9366
4	1.603	VV	0.2019	336.37564	28.05319	8.2860
5	1.834	VV	0.2837	527.82043	27.28471	13.0019
6	2.047	VV	0.3626	705.17157	24.44495	17.3706
7	2.805	VV	0.4621	606.76318	16.85056	14.9465
8	3.356	VB	0.4434	318.25156	8.98219	7.8396
9	5.379	BB	0.2146	1.65127	1.09066e-1	0.0407
10	5.972	BV	0.2838	10.28771	5.09076e-1	0.2534
11	6.798	VB	0.3527	9.67251	3.47587e-1	0.2383
Totals :				4059.56397	240.51995	

Signal 4: DAD1 D, Sig=254,16 Ref=360,100

Peak #	RetTime [min]	Type	Width [min]	Area [mAU*s]	Height [mAU]	Area %
1	0.441	BV	0.0628	164.17378	42.72782	11.7136
2	0.652	VV	0.1115	53.85139	7.34536	3.8422
3	1.036	VV	0.2150	92.28441	5.81875	6.5844
4	1.372	VV	0.1291	742.64825	85.73705	52.9872
5	1.663	VV	0.1716	106.03451	8.49791	7.5655
6	2.119	VV	0.2168	82.77892	5.34060	5.9062
7	2.770	VV	0.4078	54.97328	1.98058	3.9223
8	3.300	VV	0.2257	19.58226	1.25720	1.3972
9	3.559	VB	0.2530	11.78155	6.21878e-1	0.8406
10	4.820	BV	0.2286	3.14712	1.98908e-1	0.2245
11	5.376	VB	0.2744	26.80946	1.43212	1.9128
12	6.228	BV	0.3328	15.30834	6.90083e-1	1.0922
13	6.763	VV	0.2920	5.34601	2.51541e-1	0.3814
14	7.477	VB	0.3701	11.29175	4.16752e-1	0.8057
15	10.432	BB	0.3802	3.75049	1.29375e-1	0.2676
16	14.139	BB	0.4193	7.80006	2.21243e-1	0.5565
Totals :				1401.56159	162.66716	

===

*** End of Report ***
