# Supplementary material for: Influence of Silver Nanoparticles (AgNPs) on Vegetative Growth and Concentrations of Nutrients and Phytohormones in Tomato
Source: Plants (Basel). 2026 Jan 28;15(3):405. doi: 10.3390/plants15030405 (PMC12899181; doi:10.3390/plants15030405)
Supplement: Supplementary file 1 [file plants-15-00405-s001.zip › S1. HPLC Analysis (plants-4015186)/cv. Vengador/Roots/10 ppm/V-10-R-R1.pdf]

Data File C:\CHEM32\...ORMTMG\FITOHOR GABY Y ALE 30-11-2020 2012-10-03 09-08-53\037-3701.D
Sample Name: 10 PPM VENGADOR RAIZ R1

===

Acq. Operator : TMG Seq. Line : 37
Acq. Instrument : Instrument 1 Location : Vial 37
Injection Date : 10/4/2012 4:51:34 AM Inj : 1
Inj Volume : 200.0 µl
Different Inj Volume from Sequence ! Actual Inj Volume : 50.0 µl
Acq. Method : C:\CHEM32\1\DATA\FITOHORMTMG\FITOHOR GABY Y ALE 30-11-2020 2012-10-03 09-08-53\FITOHORMONAS DR SOTO.M
Last changed : 8/14/2013 11:13:25 AM by TMG
Analysis Method : C:\CHEM32\1\METHODS\LAVADO COLUMNA ACET.M
Last changed : 10/21/2012 12:24:49 PM by TMG
(modified after loading)

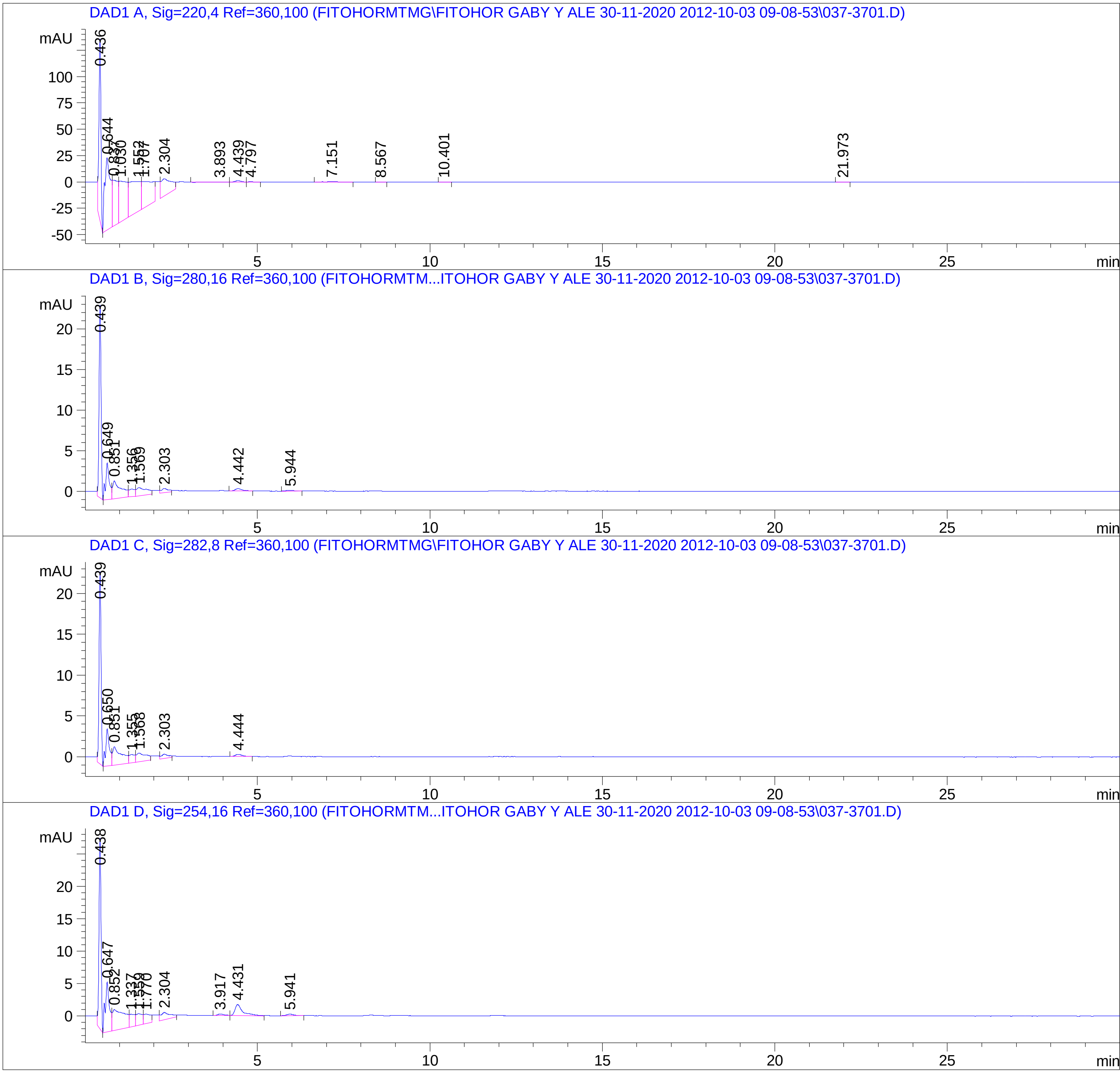

===

Area Percent Report

===

Sorted By : Signal
Multiplier: : 1.0000
Dilution: : 1.0000
Use Multiplier & Dilution Factor with ISTDs

Signal 1: DAD1 A, Sig=220,4 Ref=360,100

Peak #	RetTime [min]	Type	Width [min]	Area [mAU*s]	Height [mAU]	Area %
1	0.436	BV	0.0643	715.75171	172.72775	17.0784
2	0.644	VV	0.1527	796.11542	67.96290	18.9960
3	0.837	VV	0.1371	450.18771	43.36167	10.7418
4	1.030	VV	0.2134	625.86890	38.55613	14.9337
5	1.552	VV	0.3071	675.92908	28.25284	16.1282
6	1.707	VB	0.2718	543.21484	25.17054	12.9615
7	2.304	BB	0.2662	332.60477	16.28746	7.9362
8	3.893	BV	0.2489	5.06485	2.67536e-1	0.1209
9	4.439	VV	0.2047	18.25414	1.35903	0.4356
10	4.797	VB	0.1817	5.62859	4.27225e-1	0.1343
11	7.151	BB	0.4143	14.02404	4.46636e-1	0.3346
12	8.567	BV	0.1802	2.79246	2.39236e-1	0.0666
13	10.401	BB	0.1513	2.47638	2.42084e-1	0.0591
14	21.973	BV	0.1813	3.06066	2.45760e-1	0.0730

Totals : 4190.97353 395.54680

Signal 2: DAD1 B, Sig=280,16 Ref=360,100

Peak #	RetTime [min]	Type	Width [min]	Area [mAU*s]	Height [mAU]	Area %
1	0.439	BV	0.0655	96.52095	23.72913	45.1144
2	0.649	VV	0.1063	34.07162	4.50126	15.9253
3	0.851	VV	0.2238	38.11622	2.18168	17.8157
4	1.356	VV	0.1561	10.95960	9.39421e-1	5.1226
5	1.569	VB	0.2724	21.07561	1.00623	9.8509
6	2.303	BB	0.1887	7.27404	5.28210e-1	3.3999
7	4.442	BB	0.2357	4.21214	2.82985e-1	1.9688
8	5.944	BB	0.1998	1.71682	1.10156e-1	0.8025

Totals : 213.94702 33.27908

Signal 3: DAD1 C, Sig=282,8 Ref=360,100

Peak #	RetTime [min]	Type	Width [min]	Area [mAU*s]	Height [mAU]	Area %
1	0.439	BV	0.0655	95.96557	23.55113	44.8985
2	0.650	VV	0.1071	34.22522	4.48375	16.0126
3	0.851	VB	0.2297	40.15246	2.23349	18.7857
4	1.355	BV	0.1532	11.24880	1.00056	5.2629
5	1.568	VB	0.2585	20.73403	1.04928	9.7006
6	2.303	BB	0.1965	7.29388	5.05001e-1	3.4125
7	4.444	BB	0.2194	4.11908	2.74004e-1	1.9272
Totals :				213.73905	33.09722	

Signal 4: DAD1 D, Sig=254,16 Ref=360,100

Peak #	RetTime [min]	Type	Width [min]	Area [mAU*s]	Height [mAU]	Area %
1	0.438	BV	0.0661	121.58774	29.48101	31.7665
2	0.647	VV	0.1189	66.24512	7.66322	17.3074
3	0.852	VV	0.2944	75.82291	3.20025	19.8098
4	1.337	VV	0.1465	21.20832	1.92598	5.5410
5	1.559	VV	0.1737	22.10799	1.74627	5.7760
6	1.770	VB	0.1912	20.57357	1.45259	5.3751
7	2.304	BB	0.2587	21.43632	1.09350	5.6005
8	3.917	BV	0.2053	3.30776	2.51747e-1	0.8642
9	4.431	VB	0.2262	26.61404	1.70419	6.9533
10	5.941	BB	0.2383	3.85126	2.41235e-1	1.0062
Totals :				382.75503	48.75999	

==

*** End of Report ***
