# Supplementary material for: Influence of Silver Nanoparticles (AgNPs) on Vegetative Growth and Concentrations of Nutrients and Phytohormones in Tomato
Source: Plants (Basel). 2026 Jan 28;15(3):405. doi: 10.3390/plants15030405 (PMC12899181; doi:10.3390/plants15030405)
Supplement: Supplementary file 1 [file plants-15-00405-s001.zip › S1. HPLC Analysis (plants-4015186)/cv. Vengador/Roots/10 ppm/V-10-R-R2.pdf]

Data File C:\CHEM32\...ORMTMG\FITOHOR GABY Y ALE 30-11-2020 2012-10-03 09-08-53\038-3801.D
Sample Name: 10 PPM VENGADOR RAIZ R2

===

Acq. Operator : TMG Seq. Line : 38
Acq. Instrument : Instrument 1 Location : Vial 38
Injection Date : 10/4/2012 5:23:42 AM Inj : 1
Inj Volume : 200.0 µl
Different Inj Volume from Sequence ! Actual Inj Volume : 50.0 µl
Acq. Method : C:\CHEM32\1\DATA\FITOHORMTMG\FITOHOR GABY Y ALE 30-11-2020 2012-10-03 09-08-53\FITOHORMONAS DR SOTO.M
Last changed : 8/14/2013 11:13:25 AM by TMG
Analysis Method : C:\CHEM32\1\METHODS\LAVADO COLUMNA ACET.M
Last changed : 10/21/2012 12:24:49 PM by TMG
(modified after loading)

DAD1 A, Sig=220,4 Ref=360,100 (FITOHORMTMG\FITOHOR GABY Y ALE 30-11-2020 2012-10-03 09-08-53\038-3801.D)

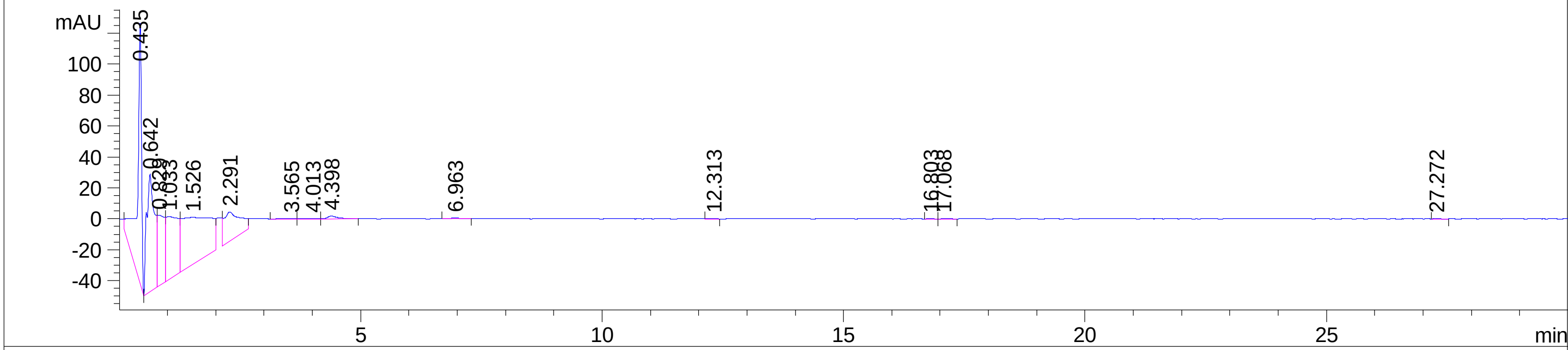

DAD1 B, Sig=280,16 Ref=360,100 (FITOHORMTM...ITOHOR GABY Y ALE 30-11-2020 2012-10-03 09-08-53\038-3801.D)

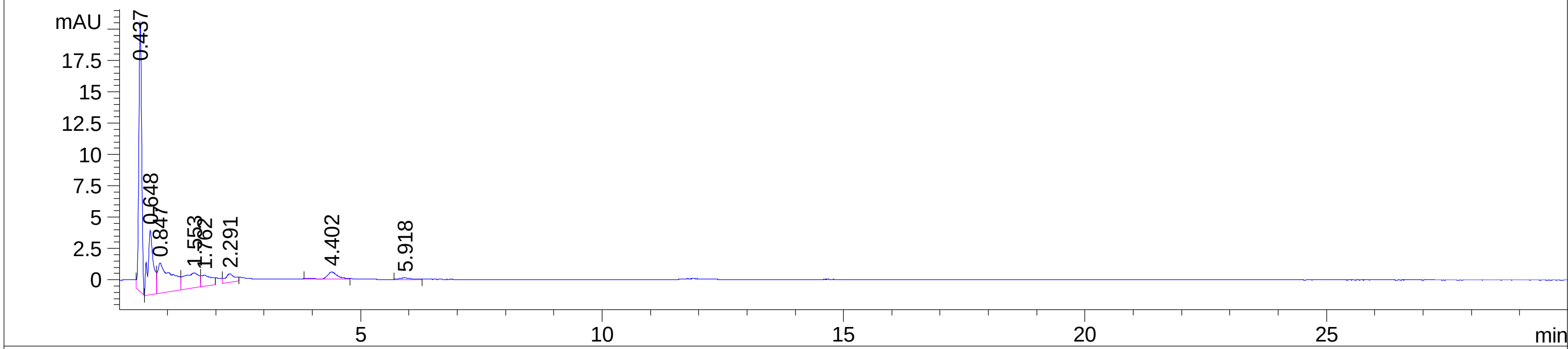

DAD1 C, Sig=282,8 Ref=360,100 (FITOHORMTMG\FITOHOR GABY Y ALE 30-11-2020 2012-10-03 09-08-53\038-3801.D)

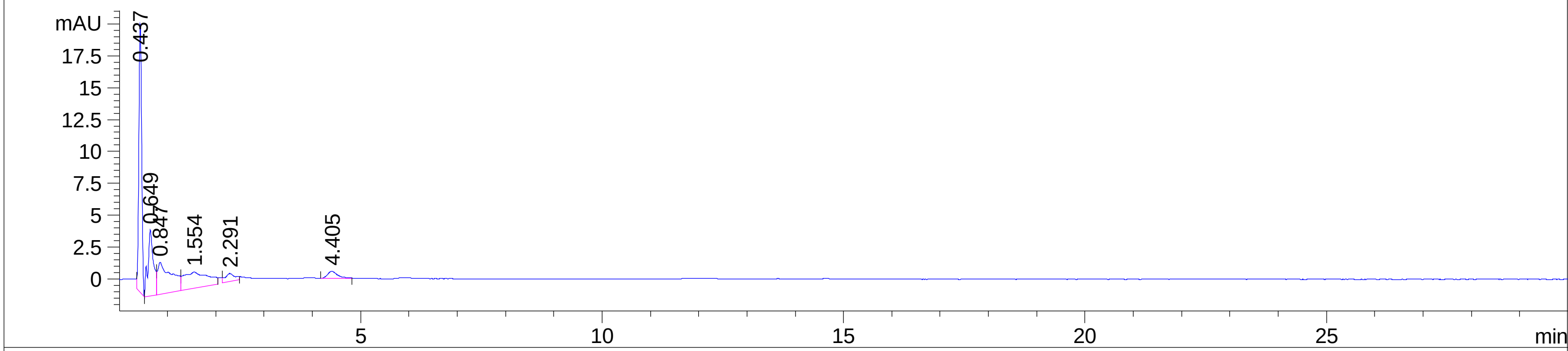

DAD1 D, Sig=254,16 Ref=360,100 (FITOHORMTM...ITOHOR GABY Y ALE 30-11-2020 2012-10-03 09-08-53\038-3801.D)

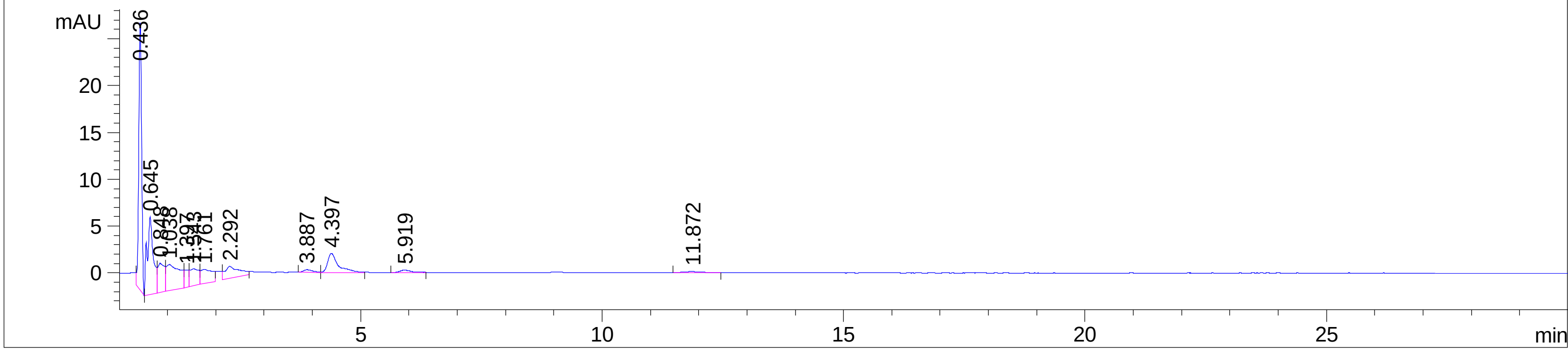

===

Area Percent Report

===

Sorted By : Signal
Multiplier: : 1.0000
Dilution: : 1.0000
Use Multiplier & Dilution Factor with ISTDs

Signal 1: DAD1 A, Sig=220,4 Ref=360,100

Peak #	RetTime [min]	Type	Width [min]	Area [mAU*s]	Height [mAU]	Area %
1	0.435	BV	0.0891	1042.85449	166.40746	21.6972
2	0.642	VV	0.1548	870.39313	75.32421	18.1090
3	0.829	VV	0.1292	427.87137	45.71990	8.9021
4	1.033	VV	0.2298	721.77893	40.91012	15.0170
5	1.526	VB	0.5014	1235.99426	30.45796	25.7155
6	2.291	BB	0.3046	443.95795	18.72592	9.2368
7	3.565	BV	0.2488	6.72390	3.83042e-1	0.1399
8	4.013	VV	0.2654	8.23073	4.18598e-1	0.1712
9	4.398	VV	0.2386	30.41440	1.82304	0.6328
10	6.963	BB	0.2642	8.11912	4.22560e-1	0.1689
11	12.313	BV	0.1404	3.04895	3.28190e-1	0.0634
12	16.803	VV	0.1387	2.45773	2.90633e-1	0.0511
13	17.068	VB	0.1524	2.61861	2.53740e-1	0.0545
14	27.272	VB	0.1369	1.94458	2.24785e-1	0.0405
Totals :				4806.40816	381.69015	

Signal 2: DAD1 B, Sig=280,16 Ref=360,100

Peak #	RetTime [min]	Type	Width [min]	Area [mAU*s]	Height [mAU]	Area %
1	0.437	BV	0.0659	88.68768	21.57985	37.5924
2	0.648	VV	0.1114	41.02636	5.12647	17.3900
3	0.847	VV	0.2470	47.11660	2.42187	19.9715
4	1.553	VV	0.2799	26.02589	1.19601	11.0317
5	1.762	VB	0.1963	12.94893	8.76642e-1	5.4887
6	2.291	BB	0.1866	9.09032	6.68679e-1	3.8531
7	4.402	BB	0.2383	9.11523	5.58961e-1	3.8637
8	5.918	BB	0.2151	1.90834	1.20257e-1	0.8089
Totals :				235.91934	32.54874	

Signal 3: DAD1 C, Sig=282,8 Ref=360,100

Peak #	RetTime [min]	Type	Width [min]	Area [mAU*s]	Height [mAU]	Area %
1	0.437	BV	0.0638	86.67731	21.15391	36.5996
2	0.649	VV	0.1112	41.26898	5.16372	17.4259
3	0.847	VV	0.2504	49.01319	2.50316	20.6959
4	1.554	VB	0.4237	42.75705	1.25432	18.0542
5	2.291	BB	0.1926	8.91682	6.39904e-1	3.7651
6	4.405	BB	0.2230	8.19272	5.52397e-1	3.4594

Totals : 236.82606 31.26741

Signal 4: DAD1 D, Sig=254,16 Ref=360,100

Peak #	RetTime [min]	Type	Width [min]	Area [mAU*s]	Height [mAU]	Area %
1	0.436	BV	0.0661	118.00545	28.61115	29.3530
2	0.645	VV	0.1202	72.29322	8.24872	17.9824
3	0.848	VV	0.1334	31.35062	3.11505	7.7982
4	1.038	VV	0.2421	52.05232	2.78410	12.9476
5	1.397	VV	0.0946	12.64469	1.83022	3.1453
6	1.543	VV	0.1697	22.28691	1.78493	5.5437
7	1.761	VB	0.2141	24.77486	1.52070	6.1626
8	2.292	BB	0.2631	25.75221	1.27777	6.4057
9	3.887	BV	0.1994	3.68577	2.76637e-1	0.9168
10	4.397	VB	0.2217	31.28134	2.03102	7.7810
11	5.919	BB	0.2262	4.30540	2.61395e-1	1.0709
12	11.872	BB	0.3522	3.58929	1.23826e-1	0.8928

Totals : 402.02208 51.86552

==

*** End of Report ***
