# Supplementary material for: Influence of Silver Nanoparticles (AgNPs) on Vegetative Growth and Concentrations of Nutrients and Phytohormones in Tomato
Source: Plants (Basel). 2026 Jan 28;15(3):405. doi: 10.3390/plants15030405 (PMC12899181; doi:10.3390/plants15030405)
Supplement: Supplementary file 1 [file plants-15-00405-s001.zip › S1. HPLC Analysis (plants-4015186)/cv. Vengador/Roots/10 ppm/V-10-R-R3.pdf]

===

Acq. Operator : TMG Seq. Line : 39
Acq. Instrument : Instrument 1 Location : Vial 39
Injection Date : 10/4/2012 5:55:48 AM Inj : 1
Inj Volume : 200.0 µl
Different Inj Volume from Sequence ! Actual Inj Volume : 50.0 µl
Acq. Method : C:\CHEM32\1\DATA\FITOHORMTMG\FITOHOR GABY Y ALE 30-11-2020 2012-10-03 09-08-53\FITOHORMONAS DR SOTO.M
Last changed : 8/14/2013 11:13:25 AM by TMG
Analysis Method : C:\CHEM32\1\METHODS\LAVADO COLUMNA ACET.M
Last changed : 10/21/2012 12:24:49 PM by TMG
(modified after loading)

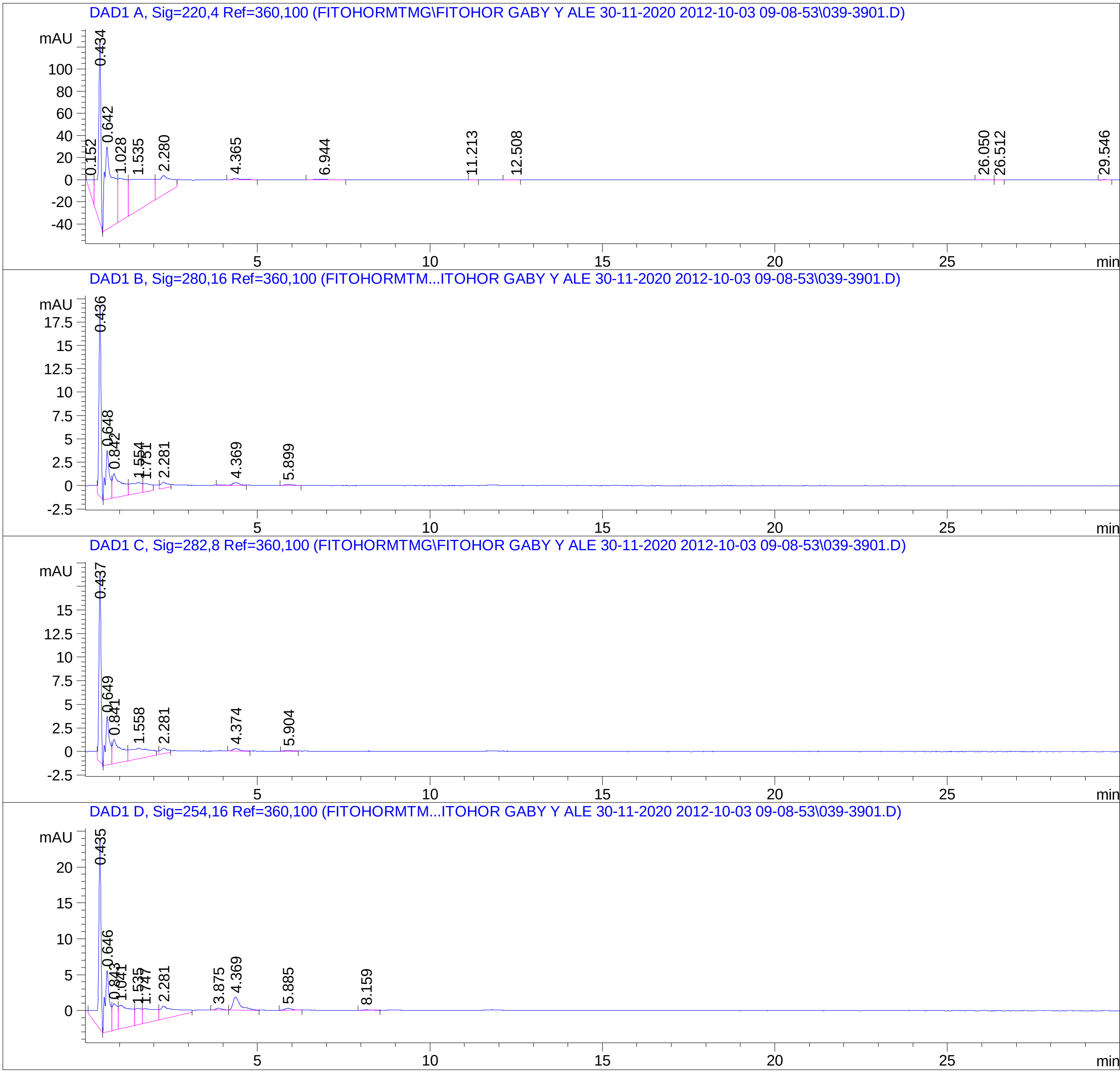

===

Area Percent Report

===

Sorted By : Signal
Multiplier: : 1.0000
Dilution: : 1.0000
Use Multiplier & Dilution Factor with ISTDs

Signal 1: DAD1 A, Sig=220,4 Ref=360,100

Peak #	RetTime [min]	Type	Width [min]	Area [mAU*s]	Height [mAU]	Area %
1	0.152	BV	0.1916	152.69601	11.16289	3.2343
2	0.434	VV	0.0815	876.12555	166.09918	18.5575
3	0.642	VV	0.2109	1240.78833	74.28173	26.2816
4	1.028	VV	0.2298	681.22089	38.61182	14.4292
5	1.535	VV	0.5213	1211.11865	28.41216	25.6531
6	2.280	VB	0.3706	513.77765	17.49638	10.8825
7	4.365	BB	0.2177	20.33025	1.33499	0.4306
8	6.944	BB	0.3088	9.65114	4.06828e-1	0.2044
9	11.213	VB	0.1379	2.51964	2.67593e-1	0.0534
10	12.508	BV	0.2441	4.28998	2.27443e-1	0.0909
11	26.050	BB	0.1765	3.55577	3.12999e-1	0.0753
12	26.512	BV	0.1566	2.33586	2.33723e-1	0.0495
13	29.546	BB	0.1175	2.72723	3.72017e-1	0.0578
Totals :				4721.13694	339.21975	

Signal 2: DAD1 B, Sig=280,16 Ref=360,100

Peak #	RetTime [min]	Type	Width [min]	Area [mAU*s]	Height [mAU]	Area %
1	0.436	BV	0.0671	86.41198	20.53721	36.6902
2	0.648	VV	0.1116	41.66057	5.19461	17.6889
3	0.842	VV	0.2390	48.61451	2.58951	20.6415
4	1.554	VV	0.3127	28.12875	1.13670	11.9434
5	1.751	VB	0.2077	14.19668	9.11546e-1	6.0279
6	2.281	BB	0.1925	9.75984	6.83832e-1	4.1440
7	4.369	BB	0.2378	4.81149	2.92623e-1	2.0429
8	5.899	BB	0.2062	1.93418	1.21164e-1	0.8212
Totals :				235.51799	31.46720	

Signal 3: DAD1 C, Sig=282,8 Ref=360,100

Peak #	RetTime [min]	Type	Width [min]	Area [mAU*s]	Height [mAU]	Area %
1	0.437	BV	0.0669	84.95403	20.27291	36.9883
2	0.649	VV	0.1100	40.36967	5.12034	17.5766
3	0.841	VB	0.2311	45.87547	2.53589	19.9738
4	1.558	BB	0.5013	44.26333	1.09580	19.2719
5	2.281	BB	0.1969	8.77987	6.06305e-1	3.8227
6	4.374	BB	0.2097	3.98487	2.77149e-1	1.7350
7	5.904	BB	0.1802	1.45059	1.03073e-1	0.6316

Totals : 229.67783 30.01146

Signal 4: DAD1 D, Sig=254,16 Ref=360,100

Peak #	RetTime [min]	Type	Width [min]	Area [mAU*s]	Height [mAU]	Area %
1	0.435	BV	0.0788	134.89833	26.77325	27.1500
2	0.646	VV	0.1206	75.02459	8.52915	15.0996
3	0.843	VV	0.1414	38.54418	3.70331	7.7575
4	1.041	VV	0.2977	77.34052	3.27329	15.5658
5	1.535	VV	0.1795	29.86999	2.26952	6.0117
6	1.747	VV	0.3100	50.01687	2.01214	10.0665
7	2.281	VB	0.3917	54.31785	1.74224	10.9322
8	3.875	BV	0.1987	3.48403	2.62689e-1	0.7012
9	4.369	VB	0.2205	27.83967	1.82000	5.6031
10	5.885	BB	0.2301	4.20012	2.72007e-1	0.8453
11	8.159	BB	0.2227	1.32713	8.11807e-2	0.2671

Totals : 496.86327 50.73878

===

*** End of Report ***
