# Supplementary material for: Influence of Silver Nanoparticles (AgNPs) on Vegetative Growth and Concentrations of Nutrients and Phytohormones in Tomato
Source: Plants (Basel). 2026 Jan 28;15(3):405. doi: 10.3390/plants15030405 (PMC12899181; doi:10.3390/plants15030405)
Supplement: Supplementary file 1 [file plants-15-00405-s001.zip › S1. HPLC Analysis (plants-4015186)/cv. Vengador/Roots/5 ppm/V-5-R-R1.pdf]

===

Acq. Operator : TMG Seq. Line : 25
Acq. Instrument : Instrument 1 Location : Vial 25
Injection Date : 10/3/2012 10:26:11 PM Inj : 1
Inj Volume : 200.0 µl
Different Inj Volume from Sequence ! Actual Inj Volume : 50.0 µl
Acq. Method : C:\CHEM32\1\DATA\FITOHORMTMG\FITOHOR GABY Y ALE 30-11-2020 2012-10-03 09-08-53\FITOHORMONAS DR SOTO.M
Last changed : 8/14/2013 11:13:25 AM by TMG
Analysis Method : C:\CHEM32\1\METHODS\LAVADO COLUMNA ACET.M
Last changed : 10/21/2012 12:24:49 PM by TMG
(modified after loading)

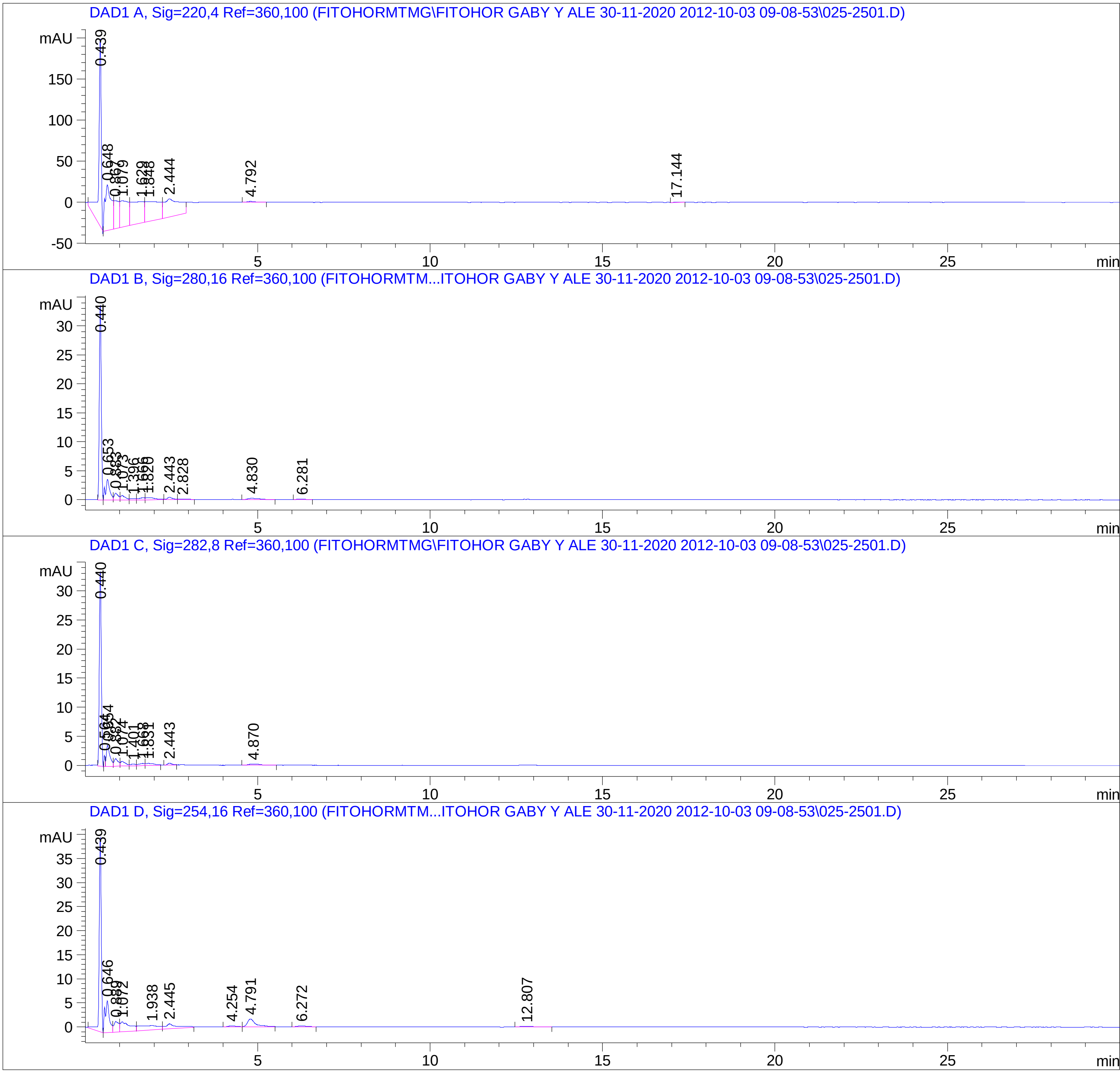

===

Area Percent Report

===

Sorted By : Signal
Multiplier: : 1.0000
Dilution: : 1.0000
Use Multiplier & Dilution Factor with ISTDs

Signal 1: DAD1 A, Sig=220,4 Ref=360,100

Peak #	RetTime [min]	Type	Width [min]	Area [mAU*s]	Height [mAU]	Area %
1	0.439	BV	0.0759	1162.62524	226.30595	23.8062
2	0.648	VV	0.1683	703.17883	55.31954	14.3985
3	0.867	VV	0.1304	335.56705	34.21838	6.8711
4	1.079	VV	0.2187	548.66571	32.22164	11.2346
5	1.629	VV	0.3402	690.11102	25.78577	14.1309
6	1.848	VV	0.3739	706.03577	23.81479	14.4570
7	2.444	VB	0.4127	716.38525	21.95670	14.6689
8	4.792	BB	0.2415	18.61188	1.07779	0.3811
9	17.144	BB	0.1442	2.53040	2.72738e-1	0.0518

Totals : 4883.71116 420.97329

Signal 2: DAD1 B, Sig=280,16 Ref=360,100

Peak #	RetTime [min]	Type	Width [min]	Area [mAU*s]	Height [mAU]	Area %
1	0.440	BV	0.0647	134.85623	33.65790	64.4853
2	0.653	VV	0.1170	29.68818	3.57225	14.1962
3	0.883	VV	0.1113	9.77226	1.22182	4.6729
4	1.073	VV	0.1348	7.73177	7.72582e-1	3.6972
5	1.396	VV	0.1707	2.71639	2.42510e-1	1.2989
6	1.666	VV	0.1720	4.24428	3.53731e-1	2.0295
7	1.820	VB	0.2635	7.28129	3.54751e-1	3.4817
8	2.443	BB	0.1670	4.76385	4.17797e-1	2.2780
9	2.828	BB	0.2265	1.97675	1.16180e-1	0.9452
10	4.830	BB	0.3295	4.71115	2.00957e-1	2.2528
11	6.281	BB	0.2028	1.38510	8.65204e-2	0.6623

Totals : 209.12724 40.99700

Signal 3: DAD1 C, Sig=282,8 Ref=360,100

Peak #	RetTime [min]	Type	Width [min]	Area [mAU*s]	Height [mAU]	Area %
1	0.440	BV	0.0648	134.39499	33.48776	64.8963
2	0.564	VV	0.0355	4.57657	1.94251	2.2099
3	0.654	VV	0.1057	25.81263	3.59618	12.4643
4	0.882	VV	0.1150	10.88538	1.30949	5.2563
5	1.074	VV	0.1387	8.52207	8.23418e-1	4.1151
6	1.401	VV	0.1750	3.30898	2.85966e-1	1.5978
7	1.668	VV	0.1727	4.68829	3.83404e-1	2.2639
8	1.831	VB	0.2371	6.84620	3.71366e-1	3.3059
9	2.443	BB	0.1407	3.34960	3.52989e-1	1.6174
10	4.870	BB	0.3133	4.70715	1.89806e-1	2.2730
Totals :				207.09186	42.74289	

Signal 4: DAD1 D, Sig=254,16 Ref=360,100

Peak #	RetTime [min]	Type	Width [min]	Area [mAU*s]	Height [mAU]	Area %
1	0.439	BV	0.0677	171.88419	40.37240	43.6993
2	0.646	VV	0.1240	58.58990	6.57160	14.8957
3	0.889	VV	0.1357	21.76071	2.19428	5.5324
4	1.072	VV	0.2513	40.63687	2.04993	10.3314
5	1.938	VV	0.5060	37.82312	9.15577e-1	9.6160
6	2.445	VB	0.3184	24.85875	1.01959	6.3200
7	4.254	BV	0.2159	3.07521	2.08681e-1	0.7818
8	4.791	VB	0.2456	27.40335	1.63408	6.9669
9	6.272	BB	0.2443	3.87826	2.13104e-1	0.9860
10	12.807	BB	0.3480	3.42391	1.18902e-1	0.8705
Totals :				393.33425	55.29816	

==

*** End of Report ***
