# Supplementary material for: Influence of Silver Nanoparticles (AgNPs) on Vegetative Growth and Concentrations of Nutrients and Phytohormones in Tomato
Source: Plants (Basel). 2026 Jan 28;15(3):405. doi: 10.3390/plants15030405 (PMC12899181; doi:10.3390/plants15030405)
Supplement: Supplementary file 1 [file plants-15-00405-s001.zip › S1. HPLC Analysis (plants-4015186)/cv. Vengador/Roots/5 ppm/V-5-R-R2.pdf]

===

Acq. Operator : TMG   Seq. Line : 26
Acq. Instrument : Instrument 1   Location : Vial 26
Injection Date : 10/3/2012 10:58:18 PM   Inj : 1
Inj Volume : 200.0 µl
Different Inj Volume from Sequence !   Actual Inj Volume : 50.0 µl
Acq. Method : C:\CHEM32\1\DATA\FITOHORMTMG\FITOHOR GABY Y ALE 30-11-2020 2012-10-03 09-08-53\FITOHORMONAS DR SOTO.M
Last changed : 8/14/2013 11:13:25 AM by TMG
Analysis Method : C:\CHEM32\1\METHODS\LAVADO COLUMNA ACET.M
Last changed : 10/21/2012 12:24:49 PM by TMG
(modified after loading)

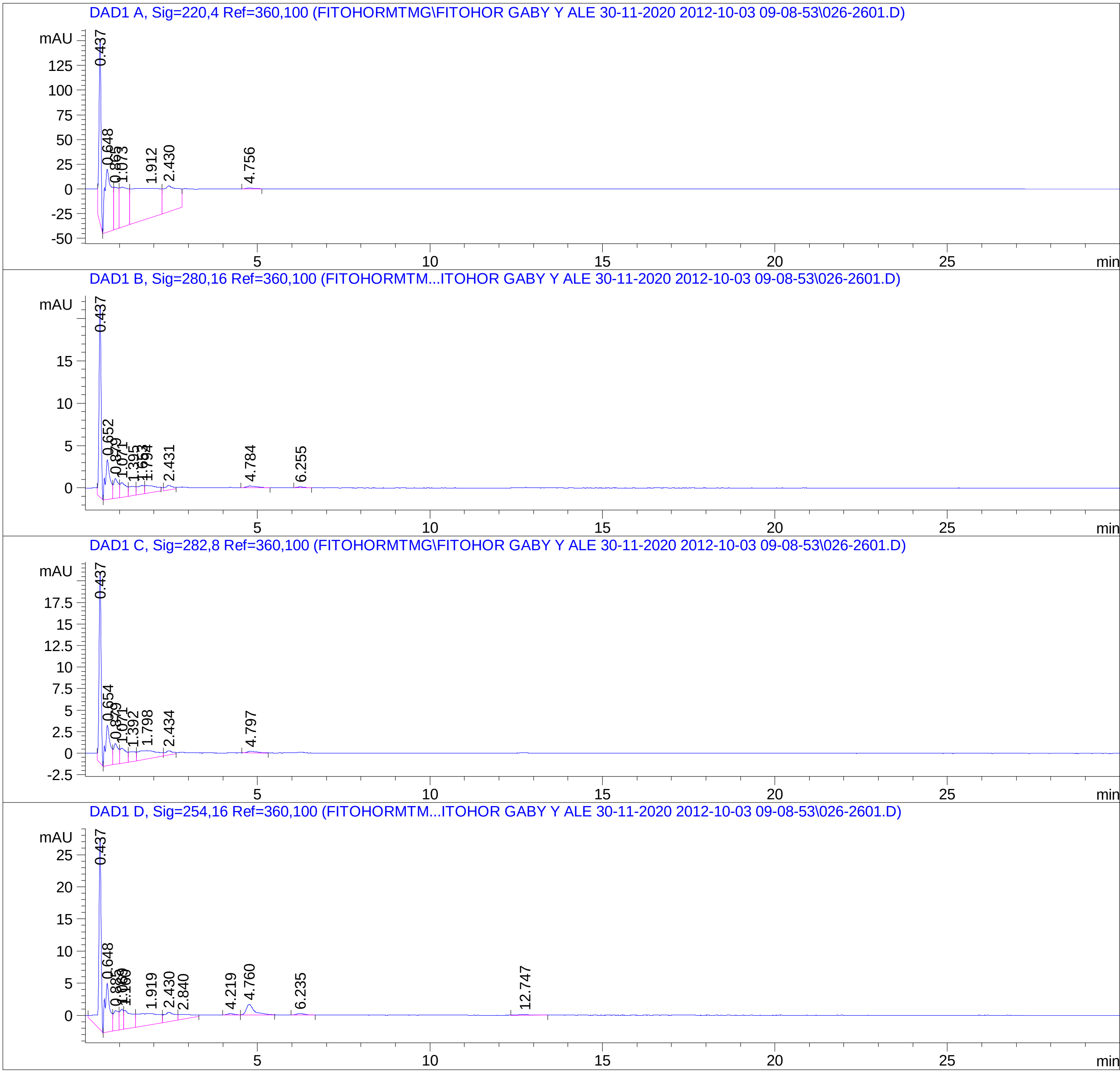

===

Area Percent Report

===

Sorted By : Signal
Multiplier: : 1.0000
Dilution: : 1.0000
Use Multiplier & Dilution Factor with ISTDs

Signal 1: DAD1 A, Sig=220,4 Ref=360,100

Peak #	RetTime [min]	Type	Width [min]	Area [mAU*s]	Height [mAU]	Area %
1	0.437	BV	0.0644	767.18951	185.04709	14.5097
2	0.648	VV	0.1754	845.64807	63.47724	15.9936
3	0.865	VV	0.1309	406.02435	42.70796	7.6790
4	1.073	VV	0.2294	712.64758	40.47126	13.4781
5	1.912	VV	0.7215	1753.03210	29.49052	33.1547
6	2.430	VB	0.3855	786.81873	25.96805	14.8809
7	4.756	BB	0.2250	16.07081	1.08391	0.3039

Totals : 5287.43116 388.24604

Signal 2: DAD1 B, Sig=280,16 Ref=360,100

Peak #	RetTime [min]	Type	Width [min]	Area [mAU*s]	Height [mAU]	Area %
1	0.437	BV	0.0661	93.34629	22.64160	38.1257
2	0.652	VV	0.1314	44.46178	4.65681	18.1597
3	0.879	VV	0.1341	22.71951	2.32278	9.2794
4	1.071	VV	0.1650	21.60486	1.71497	8.8241
5	1.395	VV	0.1673	13.93504	1.06039	5.6915
6	1.653	VV	0.2000	14.25443	1.00161	5.8220
7	1.794	VB	0.2850	20.86513	9.18646e-1	8.5220
8	2.431	BB	0.2051	8.24675	5.43118e-1	3.3682
9	4.784	BB	0.2713	4.00025	1.93432e-1	1.6338
10	6.255	BB	0.1854	1.40410	9.55985e-2	0.5735

Totals : 244.83813 35.14895

Signal 3: DAD1 C, Sig=282,8 Ref=360,100

Peak #	RetTime [min]	Type	Width [min]	Area [mAU*s]	Height [mAU]	Area %
1	0.437	BV	0.0662	91.95487	22.24757	37.1229
2	0.654	VV	0.1344	45.19508	4.68934	18.2456
3	0.879	VV	0.1359	24.01635	2.41710	9.6956
4	1.071	VV	0.1637	21.66573	1.75916	8.7466
5	1.392	VV	0.1750	15.57643	1.11497	6.2883
6	1.798	VV	0.4834	37.58834	9.54519e-1	15.1747
7	2.434	VB	0.2063	7.76206	5.13705e-1	3.1336
8	4.797	BB	0.2758	3.94534	1.85786e-1	1.5928

Totals : 247.70420 33.88215

Signal 4: DAD1 D, Sig=254,16 Ref=360,100

Peak #	RetTime [min]	Type	Width [min]	Area [mAU*s]	Height [mAU]	Area %
1	0.437	BV	0.0738	142.95041	29.87747	29.1864
2	0.648	VV	0.1338	73.59725	7.54700	15.0265
3	0.885	VV	0.1367	32.32130	3.12319	6.5991
4	1.069	VV	0.1086	24.68537	3.17705	5.0401
5	1.160	VV	0.2143	49.02129	2.91359	10.0088
6	1.919	VV	0.5596	78.86048	1.73060	16.1011
7	2.430	VV	0.2849	30.93051	1.44951	6.3151
8	2.840	VB	0.3288	20.14102	7.36972e-1	4.1122
9	4.219	BV	0.2017	2.85435	2.05919e-1	0.5828
10	4.760	VB	0.2414	27.52291	1.65996	5.6194
11	6.235	BB	0.2377	4.00686	2.38881e-1	0.8181
12	12.747	BB	0.3488	2.89219	1.00181e-1	0.5905

Totals : 489.78394 52.76031

===

*** End of Report ***
