# Supplementary material for: Influence of Silver Nanoparticles (AgNPs) on Vegetative Growth and Concentrations of Nutrients and Phytohormones in Tomato
Source: Plants (Basel). 2026 Jan 28;15(3):405. doi: 10.3390/plants15030405 (PMC12899181; doi:10.3390/plants15030405)
Supplement: Supplementary file 1 [file plants-15-00405-s001.zip › S1. HPLC Analysis (plants-4015186)/cv. Vengador/Roots/5 ppm/V-5-R-R3.pdf]

Data File C:\CHEM32\...ORMTMG\FITOHOR GABY Y ALE 30-11-2020 2012-10-03 09-08-53\027-2701.D
Sample Name: 5 PPM VENGADOR RAIZ R3

===

Acq. Operator : TMG   Seq. Line : 27
Acq. Instrument : Instrument 1   Location : Vial 27
Injection Date : 10/3/2012 11:30:24 PM   Inj : 1
                                                  Inj Volume : 200.0 µl
Different Inj Volume from Sequence !   Actual Inj Volume : 50.0 µl
Acq. Method : C:\CHEM32\1\DATA\FITOHORMTMG\FITOHOR GABY Y ALE 30-11-2020 2012-10-03 09-08-53\FITOHORMONAS DR SOTO.M
Last changed : 8/14/2013 11:13:25 AM by TMG
Analysis Method : C:\CHEM32\1\METHODS\LAVADO COLUMNA ACET.M
Last changed : 10/21/2012 12:24:49 PM by TMG
(modified after loading)

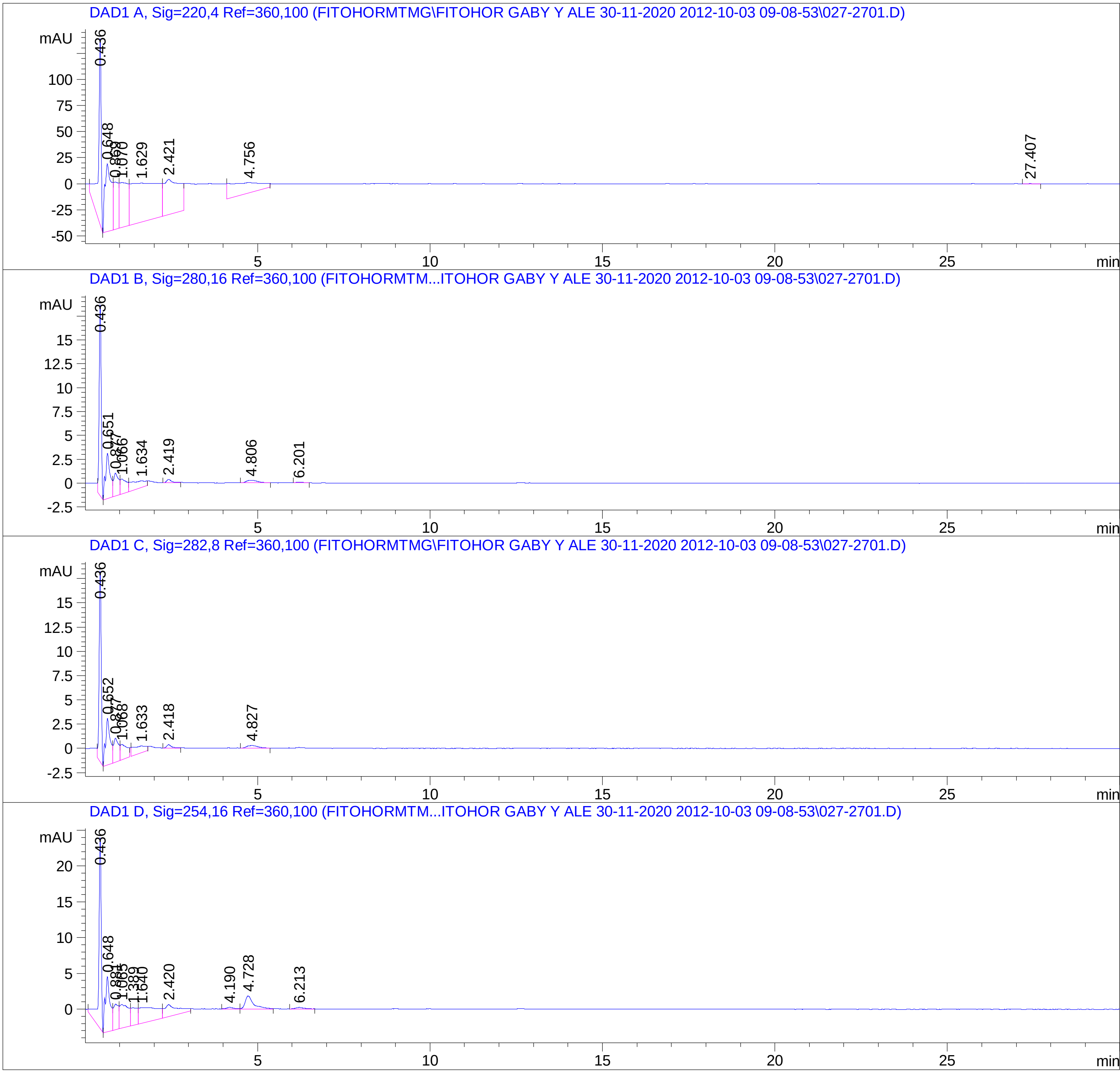

===

Area Percent Report

===

Sorted By : Signal
Multiplier: : 1.0000
Dilution: : 1.0000
Use Multiplier & Dilution Factor with ISTDs

Signal 1: DAD1 A, Sig=220,4 Ref=360,100

Peak #	RetTime [min]	Type	Width [min]	Area [mAU*s]	Height [mAU]	Area %
1	0.436	BV	0.0849	1039.90930	176.01813	15.0085
2	0.648	VV	0.1691	824.67542	64.54428	11.9021
3	0.869	VV	0.1490	485.18793	45.27225	7.0025
4	1.070	VV	0.2176	730.43604	43.11638	10.5420
5	1.629	VV	0.6701	2057.39746	37.37975	29.6934
6	2.421	VB	0.4134	1105.93115	33.82683	15.9614
7	4.756	BB	0.8806	681.83533	9.69408	9.8406
8	27.407	BB	0.2067	3.43308	2.24123e-1	0.0495

Totals : 6928.80570 410.07583

Signal 2: DAD1 B, Sig=280,16 Ref=360,100

Peak #	RetTime [min]	Type	Width [min]	Area [mAU*s]	Height [mAU]	Area %
1	0.436	BV	0.0647	83.74359	20.07582	40.5280
2	0.651	VV	0.1296	44.48655	4.73649	21.5294
3	0.877	VV	0.1348	24.13835	2.41194	11.6818
4	1.066	VV	0.1645	19.71474	1.56969	9.5410
5	1.634	VB	0.4171	24.04326	7.13532e-1	11.6358
6	2.419	BB	0.1473	3.73127	3.64939e-1	1.8058
7	4.806	BB	0.2896	5.67306	2.60954e-1	2.7455
8	6.201	BB	0.1704	1.10079	8.10615e-2	0.5327

Totals : 206.63160 30.21443

Signal 3: DAD1 C, Sig=282,8 Ref=360,100

Peak #	RetTime [min]	Type	Width [min]	Area [mAU*s]	Height [mAU]	Area %
1	0.436	BV	0.0672	83.15543	19.71642	40.7276
2	0.652	VV	0.1301	44.97030	4.76396	22.0254

Peak #	RetTime [min]	Type	Width [min]	Area [mAU*s]	Height [mAU]	Area %
3	0.877	VV	0.1377	24.80964	2.45907	12.1512
4	1.068	VB	0.1787	21.48289	1.57964	10.5218
5	1.633	BB	0.3649	20.49586	7.01361e-1	10.0384
6	2.418	BB	0.1497	3.60069	3.51084e-1	1.7635
7	4.827	BB	0.2715	5.65995	2.68977e-1	2.7721

Totals : 204.17476 29.84051

Signal 4: DAD1 D, Sig=254,16 Ref=360,100

Peak #	RetTime [min]	Type	Width [min]	Area [mAU*s]	Height [mAU]	Area %
1	0.436	BV	0.0753	136.16727	26.77486	27.3227
2	0.648	VV	0.1341	75.43725	7.71414	15.1369
3	0.881	VV	0.1412	37.75880	3.57619	7.5765
4	1.065	VV	0.2253	58.74145	3.33835	11.7868
5	1.389	VV	0.1704	31.23605	2.45543	6.2677
6	1.640	VV	0.4345	76.87605	2.19545	15.4256
7	2.420	VB	0.3508	45.83328	1.66685	9.1967
8	4.190	BV	0.2030	2.86721	2.05215e-1	0.5753
9	4.728	VB	0.2431	30.02639	1.79598	6.0250
10	6.213	BB	0.2471	3.42318	2.00542e-1	0.6869

Totals : 498.36692 49.92301

==

*** End of Report ***
