# Supplementary material for: Influence of Silver Nanoparticles (AgNPs) on Vegetative Growth and Concentrations of Nutrients and Phytohormones in Tomato
Source: Plants (Basel). 2026 Jan 28;15(3):405. doi: 10.3390/plants15030405 (PMC12899181; doi:10.3390/plants15030405)
Supplement: Supplementary file 1 [file plants-15-00405-s001.zip › S1. HPLC Analysis (plants-4015186)/cv. Vengador/Roots/Control/V-T-R-R1.pdf]

===

Acq. Operator : TMG Seq. Line : 13
Acq. Instrument : Instrument 1 Location : Vial 13
Injection Date : 10/3/2012 4:00:50 PM Inj : 1
Inj Volume : 200.0 µl
Different Inj Volume from Sequence ! Actual Inj Volume : 50.0 µl
Acq. Method : C:\CHEM32\1\DATA\FITOHORMTMG\FITOHOR GABY Y ALE 30-11-2020 2012-10-03 09-08-53\FITOHORMONAS DR SOTO.M
Last changed : 8/14/2013 11:13:25 AM by TMG
Analysis Method : C:\CHEM32\1\METHODS\LAVADO COLUMNA ACET.M
Last changed : 10/21/2012 12:24:49 PM by TMG
(modified after loading)

DAD1 A, Sig=220,4 Ref=360,100 (FITOHORMTMG\FITOHOR GABY Y ALE 30-11-2020 2012-10-03 09-08-53\013-1301.D)

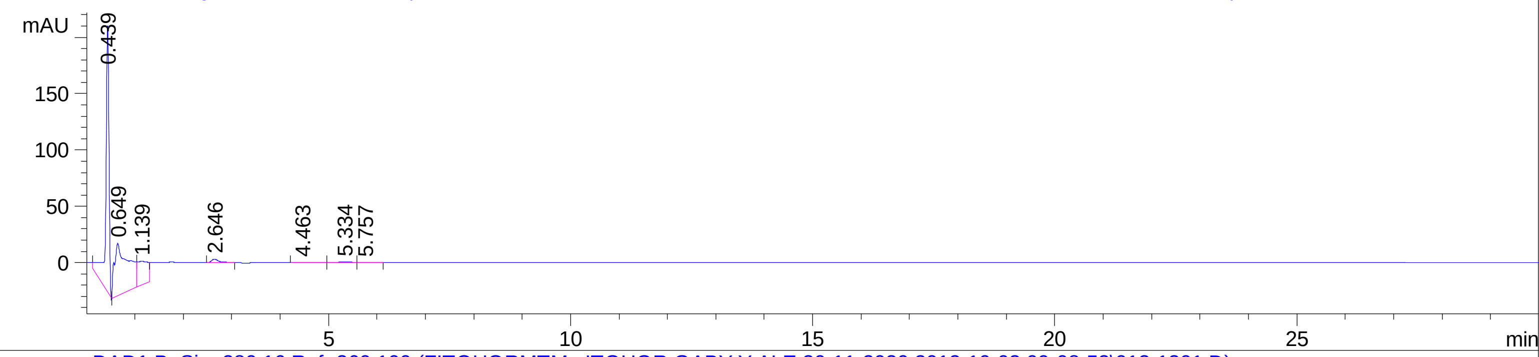

DAD1 B, Sig=280,16 Ref=360,100 (FITOHORMTM...ITOHOR GABY Y ALE 30-11-2020 2012-10-03 09-08-53\013-1301.D)

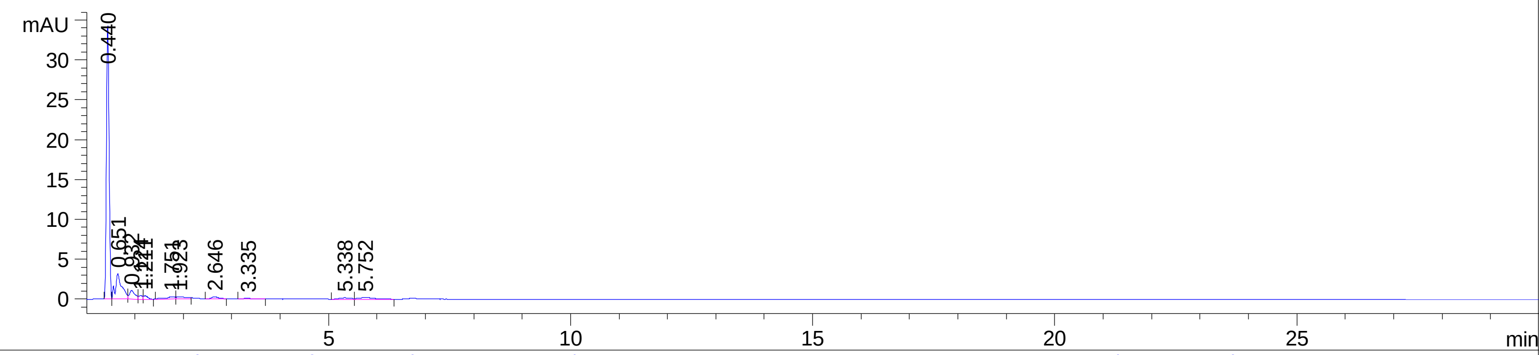

DAD1 C, Sig=282,8 Ref=360,100 (FITOHORMTMG\FITOHOR GABY Y ALE 30-11-2020 2012-10-03 09-08-53\013-1301.D)

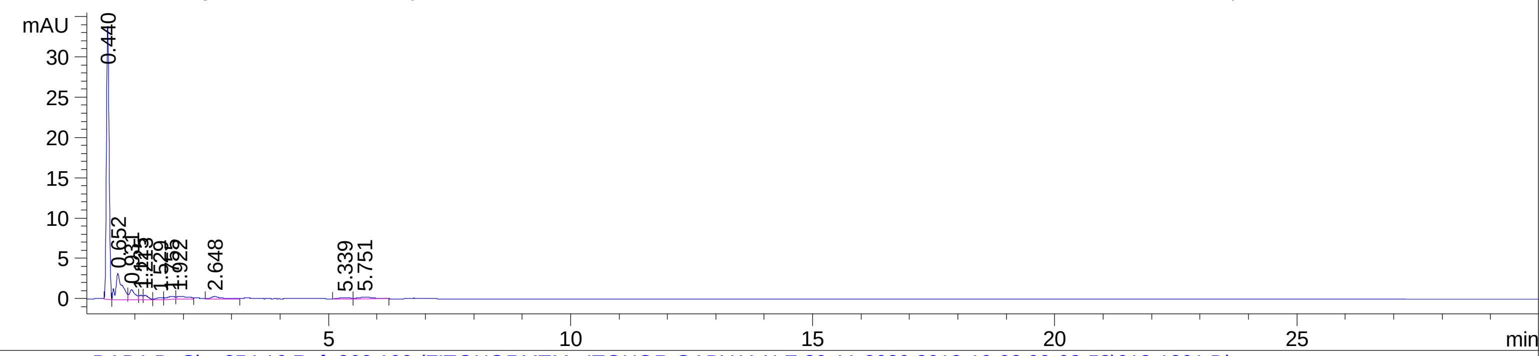

DAD1 D, Sig=254,16 Ref=360,100 (FITOHORMTM...ITOHOR GABY Y ALE 30-11-2020 2012-10-03 09-08-53\013-1301.D)

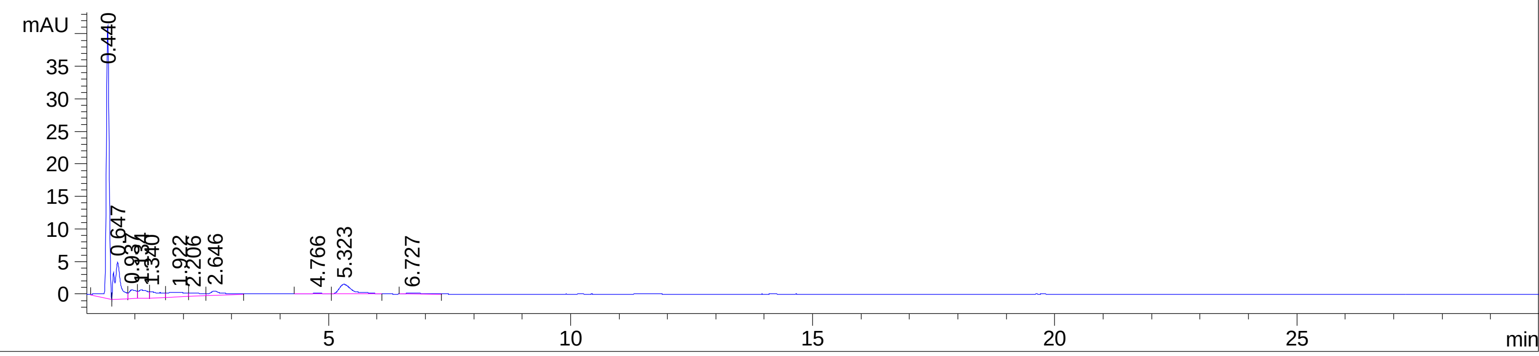

===

Area Percent Report

===

Sorted By : Signal
Multiplier: : 1.0000
Dilution: : 1.0000
Use Multiplier & Dilution Factor with ISTDs

Signal 1: DAD1 A, Sig=220,4 Ref=360,100

Peak #	RetTime [min]	Type	Width [min]	Area [mAU*s]	Height [mAU]	Area %
1	0.439	BV	0.0717	1116.75940	233.91942	46.2902
2	0.649	VV	0.2509	915.18585	46.24580	37.9349
3	1.139	VV	0.1995	316.93192	21.07339	13.1370
4	2.646	BB	0.1685	35.86349	3.15707	1.4866
5	4.463	BB	0.2550	4.82522	2.66827e-1	0.2000
6	5.334	BV	0.2610	16.22469	9.80899e-1	0.6725
7	5.757	VB	0.2439	6.72523	3.92485e-1	0.2788

Totals : 2412.51580 306.03588

Signal 2: DAD1 B, Sig=280,16 Ref=360,100

Peak #	RetTime [min]	Type	Width [min]	Area [mAU*s]	Height [mAU]	Area %
1	0.440	BV	0.0611	132.57471	34.32484	67.4887
2	0.651	VV	0.1253	29.52025	3.20864	15.0276
3	0.932	VV	0.1128	8.74167	1.09895	4.4500
4	1.124	VV	0.0801	2.34741	4.27491e-1	1.1950
5	1.211	VB	0.0895	2.59644	4.23695e-1	1.3217
6	1.751	BV	0.1855	3.97039	2.94108e-1	2.0212
7	1.923	VB	0.1997	4.36491	2.80318e-1	2.2220
8	2.646	BB	0.1750	3.54746	2.97721e-1	1.8059
9	3.335	BB	0.2319	1.59547	9.80413e-2	0.8122
10	5.338	BV	0.1932	2.59339	1.70734e-1	1.3202
11	5.752	VB	0.2979	4.58786	2.01267e-1	2.3355

Totals : 196.43997 40.82581

Signal 3: DAD1 C, Sig=282,8 Ref=360,100

Peak #	RetTime [min]	Type	Width [min]	Area [mAU*s]	Height [mAU]	Area %
1	0.440	BV	0.0611	131.33469	33.95887	64.1641
2	0.652	VV	0.1317	31.21938	3.20449	15.2524
3	0.931	VV	0.1180	10.41356	1.23924	5.0876
4	1.125	VV	0.0761	2.77355	5.21130e-1	1.3550
5	1.213	VV	0.1014	3.69252	5.29318e-1	1.8040
6	1.529	VV	0.1526	2.11647	2.08234e-1	1.0340
7	1.755	VV	0.1547	4.27828	3.76268e-1	2.0902
8	1.922	VB	0.2341	6.44211	3.47763e-1	3.1473
9	2.648	BB	0.2507	5.84308	3.23563e-1	2.8547
10	5.339	BV	0.1808	2.13719	1.51344e-1	1.0441
11	5.751	VB	0.2602	4.43476	2.15430e-1	2.1666

Totals : 204.68559 41.07566

Signal 4: DAD1 D, Sig=254,16 Ref=360,100

Peak #	RetTime [min]	Type	Width [min]	Area [mAU*s]	Height [mAU]	Area %
1	0.440	BV	0.0653	170.25143	41.99835	49.1721
2	0.647	VV	0.1256	51.12252	5.64698	14.7652
3	0.937	VV	0.1428	14.84121	1.38695	4.2864
4	1.134	VV	0.1685	16.74156	1.29747	4.8353
5	1.340	VV	0.2073	15.75852	9.81675e-1	4.5514
6	1.922	VV	0.3378	18.63494	6.79847e-1	5.3821
7	2.206	VV	0.2418	9.34047	5.19312e-1	2.6977
8	2.646	VB	0.2588	13.33977	6.86173e-1	3.8528
9	4.766	BV	0.2934	3.11946	1.45903e-1	0.9010
10	5.323	VB	0.2878	29.12952	1.49177	8.4132
11	6.727	BB	0.3227	3.95663	1.78458e-1	1.1428

Totals : 346.23604 55.01288

*** End of Report ***
