# Supplementary material for: Influence of Silver Nanoparticles (AgNPs) on Vegetative Growth and Concentrations of Nutrients and Phytohormones in Tomato
Source: Plants (Basel). 2026 Jan 28;15(3):405. doi: 10.3390/plants15030405 (PMC12899181; doi:10.3390/plants15030405)
Supplement: Supplementary file 1 [file plants-15-00405-s001.zip › S1. HPLC Analysis (plants-4015186)/cv. Vengador/Roots/Control/V-T-R-R2.pdf]

Data File C:\CHEM32\...ORMTMG\FITOHOR GABY Y ALE 30-11-2020 2012-10-03 09-08-53\014-1401.D
Sample Name: TESTIGO VENGADOR RAIZ R2

===

Acq. Operator : TMG   Seq. Line : 14
Acq. Instrument : Instrument 1   Location : Vial 14
Injection Date : 10/3/2012 4:32:57 PM   Inj : 1
Inj Volume : 200.0 µl
Different Inj Volume from Sequence !   Actual Inj Volume : 50.0 µl
Acq. Method : C:\CHEM32\1\DATA\FITOHORMTMG\FITOHOR GABY Y ALE 30-11-2020 2012-10-03 09-08-53\FITOHORMONAS DR SOTO.M
Last changed : 8/14/2013 11:13:25 AM by TMG
Analysis Method : C:\CHEM32\1\METHODS\LAVADO COLUMNA ACET.M
Last changed : 10/21/2012 12:24:49 PM by TMG
(modified after loading)

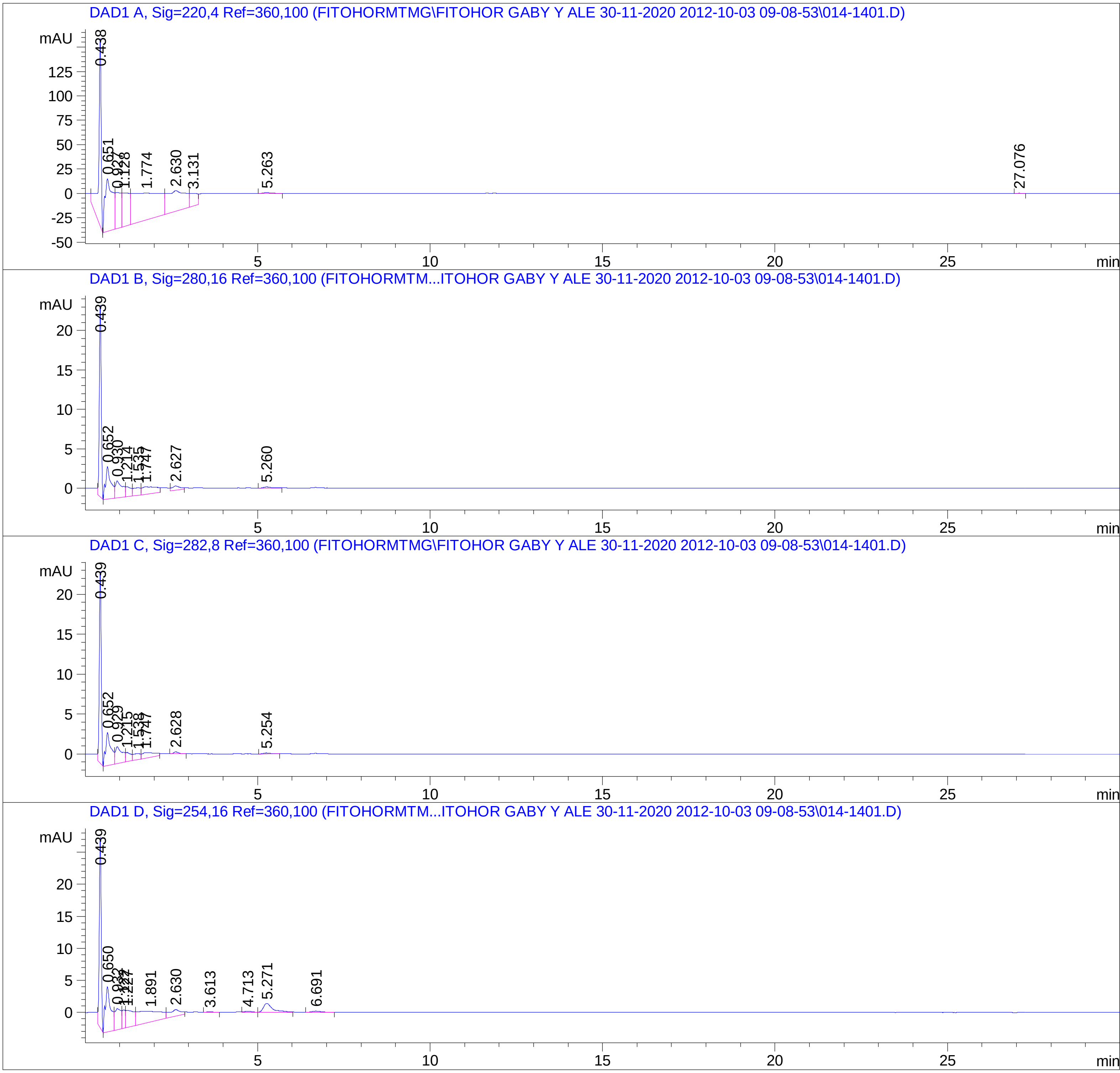

===

Area Percent Report

===

Sorted By : Signal
Multiplier: : 1.0000
Dilution: : 1.0000
Use Multiplier & Dilution Factor with ISTDs

Signal 1: DAD1 A, Sig=220,4 Ref=360,100

Peak #	RetTime [min]	Type	Width [min]	Area [mAU*s]	Height [mAU]	Area %
1	0.438	BV	0.0764	983.84406	190.14720	18.4188
2	0.651	VV	0.1956	807.93610	53.69286	15.1256
3	0.927	VV	0.1600	432.44977	37.09291	8.0960
4	1.128	VV	0.1911	515.95215	34.37584	9.6593
5	1.774	VV	0.7231	1596.39954	27.42442	29.8867
6	2.630	VV	0.4717	785.10510	21.02361	14.6982
7	3.131	VV	0.2310	202.84778	12.92909	3.7976
8	5.263	BB	0.2362	14.68882	8.91397e-1	0.2750
9	27.076	BB	0.1278	2.28845	2.67601e-1	0.0428

Totals : 5341.51176 377.84492

Signal 2: DAD1 B, Sig=280,16 Ref=360,100

Peak #	RetTime [min]	Type	Width [min]	Area [mAU*s]	Height [mAU]	Area %
1	0.439	BV	0.0638	95.56402	24.36721	40.5512
2	0.652	VV	0.1432	43.42780	4.11336	18.4280
3	0.930	VV	0.1884	30.91773	2.16778	13.1195
4	1.214	VB	0.1415	13.67064	1.27110	5.8009
5	1.535	BV	0.2093	13.41600	9.35743e-1	5.6929
6	1.747	VB	0.3644	27.14706	9.30376e-1	11.5195
7	2.627	BB	0.2302	9.13012	5.43027e-1	3.8742
8	5.260	BB	0.2483	2.38928	1.39148e-1	1.0139

Totals : 235.66264 34.46774

Signal 3: DAD1 C, Sig=282,8 Ref=360,100

Peak #	RetTime [min]	Type	Width [min]	Area [mAU*s]	Height [mAU]	Area %
1	0.439	BV	0.0638	94.43209	24.05601	44.8098
2	0.652	VV	0.1437	43.40134	4.09392	20.5948
3	0.929	VV	0.1815	29.41853	2.12496	13.9597
4	1.215	VB	0.1388	12.06142	1.14602	5.7234
5	1.538	BV	0.1879	10.53961	7.32330e-1	5.0012
6	1.747	VB	0.3193	16.66453	6.67511e-1	7.9076
7	2.628	BB	0.1489	2.36093	2.35621e-1	1.1203
8	5.254	BB	0.1955	1.86114	1.22320e-1	0.8831

Totals : 210.73958 33.17869

Signal 4: DAD1 D, Sig=254,16 Ref=360,100

Peak #	RetTime [min]	Type	Width [min]	Area [mAU*s]	Height [mAU]	Area %
1	0.439	BV	0.0646	118.61949	29.72425	27.1995
2	0.650	VV	0.1481	77.63747	7.06982	17.8023
3	0.932	VV	0.1610	39.46296	3.26634	9.0489
4	1.122	VV	0.0969	19.15097	2.83122	4.3913
5	1.227	VB	0.1992	41.18513	2.65133	9.4438
6	1.891	BV	0.6124	83.61177	1.65231	19.1722
7	2.630	VB	0.3203	24.62402	1.01777	5.6463
8	3.613	BB	0.1530	8.22243e-1	6.80360e-2	0.1885
9	4.713	BV	0.1953	1.89656	1.44373e-1	0.4349
10	5.271	VB	0.2740	25.38692	1.37097	5.8212
11	6.691	BB	0.2866	3.71231	1.87816e-1	0.8512

Totals : 436.10985 49.98423

===

*** End of Report ***
