# Supplementary material for: Influence of Silver Nanoparticles (AgNPs) on Vegetative Growth and Concentrations of Nutrients and Phytohormones in Tomato
Source: Plants (Basel). 2026 Jan 28;15(3):405. doi: 10.3390/plants15030405 (PMC12899181; doi:10.3390/plants15030405)
Supplement: Supplementary file 1 [file plants-15-00405-s001.zip › S1. HPLC Analysis (plants-4015186)/cv. Vengador/Roots/Control/V-T-R-R3.pdf]

===

Acq. Operator : TMG Seq. Line : 15
Acq. Instrument : Instrument 1 Location : Vial 15
Injection Date : 10/3/2012 5:05:04 PM Inj : 1
Inj Volume : 200.0 µl
Different Inj Volume from Sequence ! Actual Inj Volume : 50.0 µl
Acq. Method : C:\CHEM32\1\DATA\FITOHORMTMG\FITOHOR GABY Y ALE 30-11-2020 2012-10-03 09-08-53\FITOHORMONAS DR SOTO.M
Last changed : 8/14/2013 11:13:25 AM by TMG
Analysis Method : C:\CHEM32\1\METHODS\LAVADO COLUMNA ACET.M
Last changed : 10/21/2012 12:24:49 PM by TMG
(modified after loading)

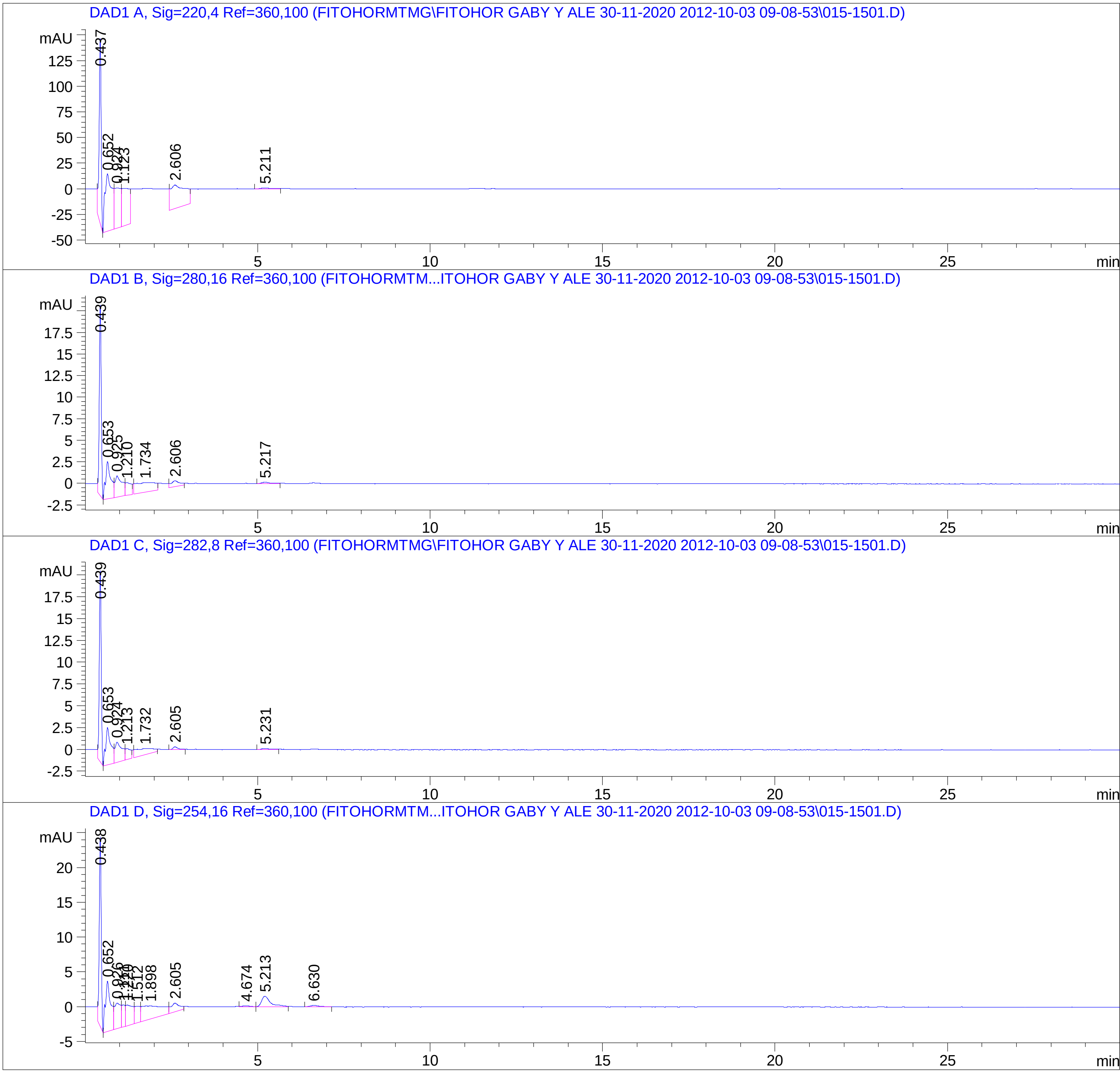

===

Area Percent Report

===

Sorted By : Signal
Multiplier: : 1.0000
Dilution: : 1.0000
Use Multiplier & Dilution Factor with ISTDs

Signal 1: DAD1 A, Sig=220,4 Ref=360,100

Peak #	RetTime [min]	Type	Width [min]	Area [mAU*s]	Height [mAU]	Area %
1	0.437	BV	0.0638	730.21649	178.10704	22.2710
2	0.652	VV	0.1882	802.41858	55.65912	24.4731
3	0.924	VV	0.1658	491.48132	39.33565	14.9898
4	1.123	VV	0.1958	560.20050	36.35483	17.0856
5	2.606	BB	0.3820	677.21222	22.83676	20.6544
6	5.211	BB	0.2703	17.24899	9.56930e-1	0.5261

Totals : 3278.77810 333.25032

Signal 2: DAD1 B, Sig=280,16 Ref=360,100

Peak #	RetTime [min]	Type	Width [min]	Area [mAU*s]	Height [mAU]	Area %
1	0.439	BV	0.0643	87.80804	22.12225	35.8173
2	0.653	VV	0.1438	45.69633	4.30717	18.6398
3	0.925	VV	0.1919	35.98191	2.44246	14.6772
4	1.210	VB	0.1489	16.71262	1.48901	6.8172
5	1.734	BB	0.4811	43.29988	1.11976	17.6622
6	2.606	BB	0.2461	12.48628	6.99904e-1	5.0932
7	5.217	BB	0.2592	3.17012	1.68739e-1	1.2931

Totals : 245.15520 32.34930

Signal 3: DAD1 C, Sig=282,8 Ref=360,100

Peak #	RetTime [min]	Type	Width [min]	Area [mAU*s]	Height [mAU]	Area %
1	0.439	BV	0.0645	87.11540	21.87375	41.1822
2	0.653	VV	0.1431	44.94878	4.25845	21.2487
3	0.924	VV	0.1868	33.18104	2.32065	15.6857
4	1.213	VB	0.1444	13.66497	1.28134	6.4599
5	1.732	BB	0.4586	27.03947	7.42950e-1	12.7824

Peak #	RetTime [min]	Type	Width [min]	Area [mAU*s]	Height [mAU]	Area %
6	2.605	BB	0.1542	2.96494	2.97594e-1	1.4016
7	5.231	BB	0.2430	2.62192	1.44938e-1	1.2395

Totals : 211.53654 30.91967

Signal 4: DAD1 D, Sig=254,16 Ref=360,100

Peak #	RetTime [min]	Type	Width [min]	Area [mAU*s]	Height [mAU]	Area %
1	0.438	BV	0.0653	110.10670	27.16432	23.9798
2	0.652	VV	0.1498	80.21441	7.21248	17.4697
3	0.926	VV	0.1623	45.84710	3.70680	9.9849
4	1.111	VV	0.0958	21.14753	3.17092	4.6057
5	1.220	VV	0.1801	41.88381	2.97699	9.1218
6	1.512	VV	0.1509	25.74608	2.36619	5.6072
7	1.898	VB	0.5217	79.38465	1.85350	17.2890
8	2.605	BB	0.2460	21.85736	1.22584	4.7603
9	4.674	BV	0.1793	1.82434	1.31966e-1	0.3973
10	5.213	VB	0.2734	27.39691	1.49761	5.9667
11	6.630	BB	0.2670	3.75490	1.94674e-1	0.8178

Totals : 459.16378 51.50128

==

*** End of Report ***
