# Supplementary material for: Influence of Silver Nanoparticles (AgNPs) on Vegetative Growth and Concentrations of Nutrients and Phytohormones in Tomato
Source: Plants (Basel). 2026 Jan 28;15(3):405. doi: 10.3390/plants15030405 (PMC12899181; doi:10.3390/plants15030405)
Supplement: Supplementary file 1 [file plants-15-00405-s001.zip › S1. HPLC Analysis (plants-4015186)/Phytohormone standards/ABA.pdf]

Data File C:\CHEM32\...ORMTMG\FITOHOR GABY Y ALE 30-11-2020 2012-10-03 09-08-53\004-0401.D
Sample Name: ABSICICO

===

Acq. Operator : TMG Seq. Line : 4
Acq. Instrument : Instrument 1 Location : Vial 4
Injection Date : 10/3/2012 11:15:05 AM Inj : 1
Inj Volume : 200.0 µl
Different Inj Volume from Sequence ! Actual Inj Volume : 20.0 µl
Acq. Method : C:\CHEM32\1\DATA\FITOHORMTMG\FITOHOR GABY Y ALE 30-11-2020 2012-10-03 09-08-53\FITOHORMONAS DR SOTO.M
Last changed : 8/14/2013 11:13:25 AM by TMG
Analysis Method : C:\CHEM32\1\METHODS\LAVADO COLUMNA ACET.M
Last changed : 7/27/2013 11:58:00 AM by TMG
Additional Info : Peak(s) manually integrated

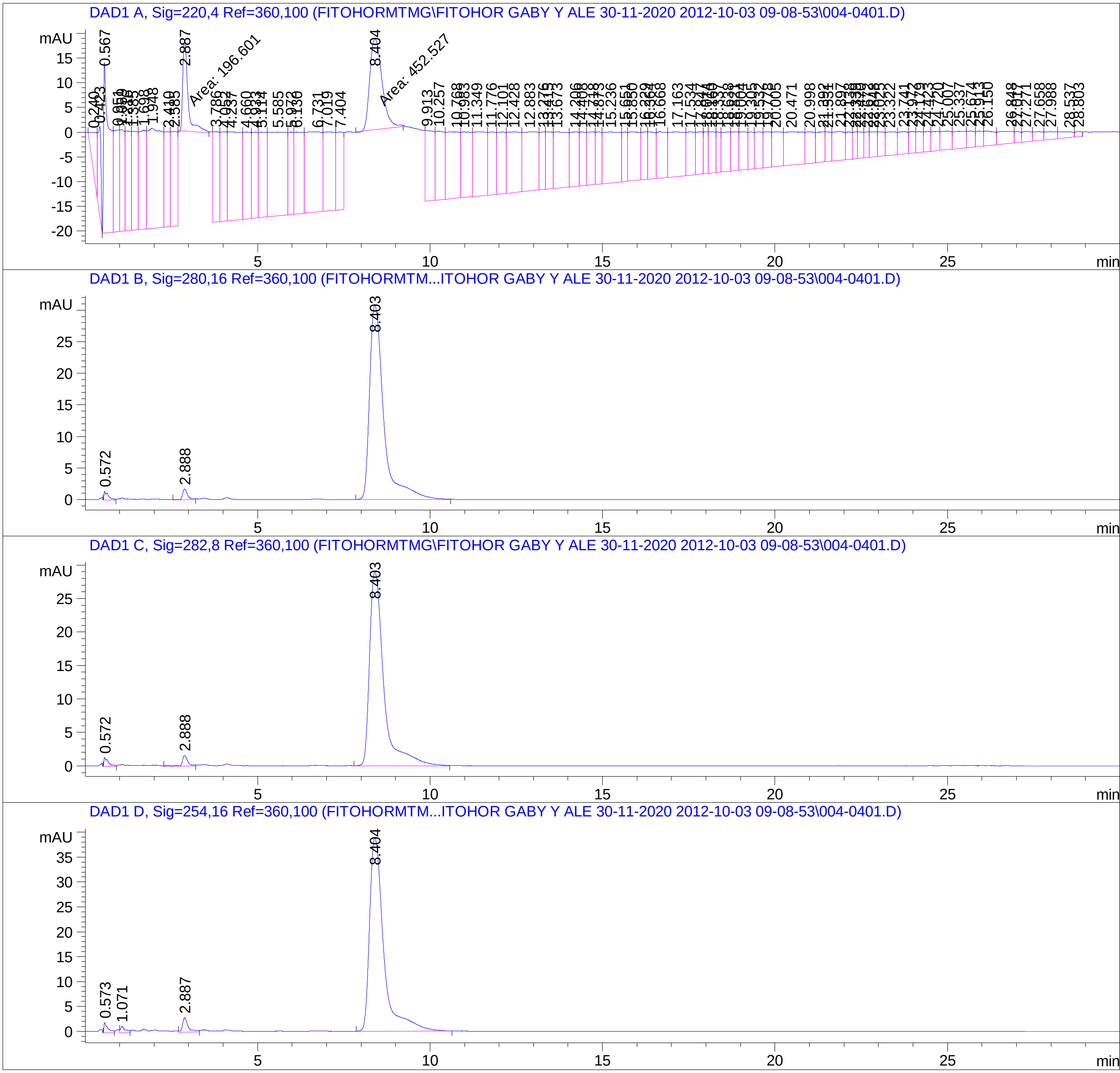

===

Area Percent Report

===

Sorted By : Signal
Multiplier: : 1.0000
Dilution: : 1.0000
Use Multiplier & Dilution Factor with ISTDs

Signal 1: DAD1 A, Sig=220,4 Ref=360,100

Peak #	RetTime [min]	Type	Width [min]	Area [mAU*s]	Height [mAU]	Area %
1	0.240	BV	0.1910	106.54630	7.62424	0.6701
2	0.423	VV	0.0941	112.73286	17.75606	0.7090
3	0.567	VV	0.1598	421.76059	34.21796	2.6527
4	0.951	VV	0.1446	216.62794	20.60934	1.3625
5	1.059	VV	0.1305	191.76080	20.62028	1.2061
6	1.216	VV	0.1513	224.51868	20.25664	1.4121
7	1.385	VV	0.1523	237.06110	20.00401	1.4910
8	1.698	VV	0.1948	293.50244	20.04389	1.8460
9	1.948	VV	0.3693	592.68701	20.26007	3.7277
10	2.410	VV	0.1496	220.07472	19.21934	1.3842
11	2.585	VV	0.1853	248.46382	19.15584	1.5627
12	2.887	MM	0.1780	196.60107	18.40551	1.2365
13	3.786	VV	0.1647	227.07588	18.31082	1.4282
14	4.052	VV	0.1689	233.57777	18.06200	1.4691
15	4.237	VV	0.3272	478.61710	17.94294	3.0102
16	4.660	VV	0.2048	257.89862	17.61613	1.6220
17	4.933	VV	0.1616	205.53485	17.42326	1.2927
18	5.114	VV	0.2067	285.61932	17.29207	1.7964
19	5.585	VV	0.4256	601.93256	16.97996	3.7858
20	5.972	VV	0.1442	175.02318	16.70555	1.1008
21	6.130	VV	0.2267	299.72250	16.59588	1.8851
22	6.731	VV	0.4144	518.84387	16.25406	3.2633
23	7.019	VV	0.2849	363.31580	15.99847	2.2851
24	7.404	VV	0.1927	213.87923	15.72174	1.3452
25	8.404	MM	0.4100	452.52686	18.39418	2.8462
26	9.913	VV	0.2179	242.75676	14.31075	1.5268
27	10.257	VV	0.2239	243.37378	13.92856	1.5307
28	10.768	VV	0.3406	363.76462	13.40527	2.2879
29	10.983	VV	0.2587	271.61987	13.27735	1.7083
30	11.349	VV	0.3232	338.78189	13.03713	2.1308
31	11.776	VV	0.2012	201.56699	12.70041	1.2677
32	12.101	VV	0.2138	203.09267	12.48181	1.2773
33	12.428	VV	0.3257	325.78629	12.27079	2.0490
34	12.883	VV	0.3766	365.18414	11.94634	2.2968
35	13.276	VV	0.1472	123.45515	11.69543	0.7765
36	13.415	VV	0.1792	158.34045	11.60344	0.9959
37	13.673	VB	0.3632	317.07312	11.44711	1.9942
38	14.206	BV	0.2519	185.67429	11.05862	1.1678

Peak #	RetTime [min]	Type	Width [min]	Area [mAU*s]	Height [mAU]	Area %
39	14.408	VV	0.1795	141.44844	10.88973	0.8896
40	14.718	VV	0.2141	163.03310	10.68203	1.0254
41	14.873	VV	0.1554	122.81744	10.58244	0.7725
42	15.236	VV	0.4245	347.44025	10.32619	2.1852
43	15.651	VV	0.1354	102.51645	10.01465	0.6448
44	15.850	VV	0.3080	226.97752	9.88015	1.4276
45	16.229	VV	0.1663	114.60667	9.66520	0.7208
46	16.364	VV	0.1962	141.29893	9.57578	0.8887
47	16.668	VV	0.2747	189.16785	9.40428	1.1898
48	17.163	VV	0.3888	282.76938	9.04425	1.7785
49	17.534	VV	0.2171	144.65710	8.73839	0.9098
50	17.847	VV	0.1734	115.09184	8.53327	0.7239
51	18.014	VV	0.1201	74.97261	8.40443	0.4715
52	18.160	VV	0.1632	107.77906	8.31423	0.6779
53	18.337	VV	0.1253	73.64214	8.16297	0.4632
54	18.638	VV	0.2251	131.78044	7.96085	0.8288
55	18.821	VV	0.1743	109.22569	7.85199	0.6870
56	19.004	VV	0.2012	122.75500	7.73421	0.7721
57	19.305	VV	0.1501	83.67295	7.50048	0.5263
58	19.532	VV	0.2036	108.25687	7.36075	0.6809
59	19.778	VV	0.1913	108.17919	7.20126	0.6804
60	20.005	VV	0.2587	146.71811	7.05293	0.9228
61	20.471	VV	0.4499	243.08273	6.72192	1.5289
62	20.998	VV	0.2467	115.11703	6.37539	0.7240
63	21.392	VV	0.2227	105.98103	6.09975	0.6666
64	21.531	VV	0.1457	66.69976	5.99840	0.4195
65	21.897	VV	0.3134	138.73264	5.83512	0.8726
66	22.138	VV	0.1626	64.55378	5.59414	0.4060
67	22.339	VV	0.1276	50.11079	5.43130	0.3152
68	22.470	VV	0.1546	56.43557	5.37062	0.3549
69	22.672	VV	0.1362	48.31474	5.21174	0.3039
70	22.848	VV	0.2076	73.34113	5.10458	0.4613
71	23.025	VV	0.1666	65.75954	4.96139	0.4136
72	23.322	VV	0.2700	100.77883	4.77934	0.6338
73	23.741	VV	0.2406	85.92664	4.54501	0.5404
74	23.972	VV	0.1775	55.97844	4.36904	0.3521
75	24.179	VV	0.1803	55.15255	4.22515	0.3469
76	24.423	VV	0.1800	50.27547	4.07214	0.3162
77	24.720	VV	0.2079	63.42447	3.85537	0.3989
78	25.007	VV	0.2840	79.50259	3.74067	0.5000
79	25.337	VV	0.2976	84.47227	3.49930	0.5313
80	25.674	VV	0.2058	49.01898	3.28961	0.3083
81	25.913	VV	0.2064	43.37860	3.11541	0.2728
82	26.150	VV	0.2891	63.48310	2.97301	0.3993
83	26.848	VV	0.3941	77.88060	2.42893	0.4898
84	27.017	VV	0.1577	26.43446	2.30722	0.1663
85	27.271	VV	0.2500	39.72234	2.08750	0.2498
86	27.658	VV	0.2600	35.32682	1.80803	0.2222
87	27.988	VV	0.2926	35.79609	1.57813	0.2251
88	28.537	VV	0.3869	35.35997	1.17581	0.2224

Peak #	RetTime [min]	Type	Width [min]	Area [mAU*s]	Height [mAU]	Area %
89	28.803	VV	0.1857	14.36688	1.09046	0.0904

Totals : 1.58996e4 937.15820

Signal 2: DAD1 B, Sig=280,16 Ref=360,100

Peak #	RetTime [min]	Type	Width [min]	Area [mAU*s]	Height [mAU]	Area %
1	0.572	VV	0.1183	12.28708	1.34841	1.3555
2	2.888	BB	0.1618	17.92354	1.68903	1.9773
3	8.403	BB	0.4342	876.25482	30.77424	96.6672

Totals : 906.46544 33.81168

Signal 3: DAD1 C, Sig=282,8 Ref=360,100

Peak #	RetTime [min]	Type	Width [min]	Area [mAU*s]	Height [mAU]	Area %
1	0.572	VV	0.1184	12.07804	1.32395	1.4083
2	2.888	VB	0.1709	18.13990	1.59301	2.1152
3	8.403	VB	0.4345	827.38879	29.02704	96.4765

Totals : 857.60673 31.94401

Signal 4: DAD1 D, Sig=254,16 Ref=360,100

Peak #	RetTime [min]	Type	Width [min]	Area [mAU*s]	Height [mAU]	Area %
1	0.573	VV	0.1121	16.89024	2.00938	1.4461
2	1.071	VV	0.1354	12.46048	1.25977	1.0668
3	2.887	BV	0.1722	33.21460	2.84499	2.8438
4	8.404	BB	0.4302	1105.40564	38.82482	94.6432

Totals : 1167.97095 44.93895

==

*** End of Report ***
