# Supplementary material for: Influence of Silver Nanoparticles (AgNPs) on Vegetative Growth and Concentrations of Nutrients and Phytohormones in Tomato
Source: Plants (Basel). 2026 Jan 28;15(3):405. doi: 10.3390/plants15030405 (PMC12899181; doi:10.3390/plants15030405)
Supplement: Supplementary file 1 [file plants-15-00405-s001.zip › S1. HPLC Analysis (plants-4015186)/Phytohormone standards/GA3.pdf]

Data File C:\CHEM32\...ORMTMG\FITOHOR GABY Y ALE 30-11-2020 2012-10-03 09-08-53\006-0601.D
Sample Name: GIBERELINAS

===

Acq. Operator : TMG   Seq. Line : 6
Acq. Instrument : Instrument 1   Location : Vial 6
Injection Date : 10/3/2012 12:18:06 PM   Inj : 1
Inj Volume : 200.0 µl
Different Inj Volume from Sequence !   Actual Inj Volume : 20.0 µl
Acq. Method : C:\CHEM32\1\DATA\FITOHORMTMG\FITOHOR GABY Y ALE 30-11-2020 2012-10-03 09-08-53\FITOHORMONAS DR SOTO.M
Last changed : 8/14/2013 11:13:25 AM by TMG
Analysis Method : C:\CHEM32\1\METHODS\LAVADO COLUMNA ACET.M
Last changed : 7/27/2013 11:58:00 AM by TMG

DAD1 A, Sig=220,4 Ref=360,100 (FITOHORMTMG\FITOHOR GABY Y ALE 30-11-2020 2012-10-03 09-08-53\006-0601.D)

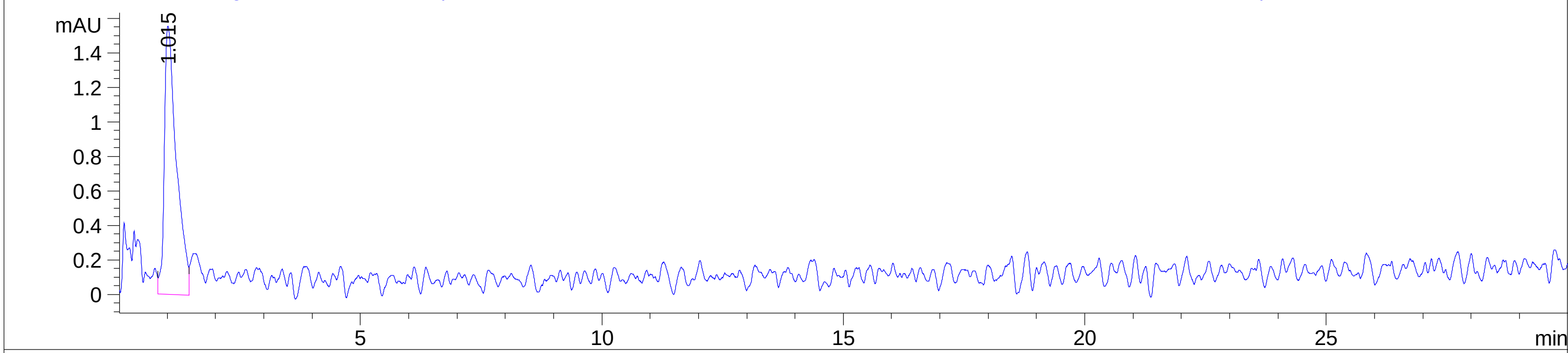

DAD1 B, Sig=280,16 Ref=360,100 (FITOHORMTM...ITOHOR GABY Y ALE 30-11-2020 2012-10-03 09-08-53\006-0601.D)

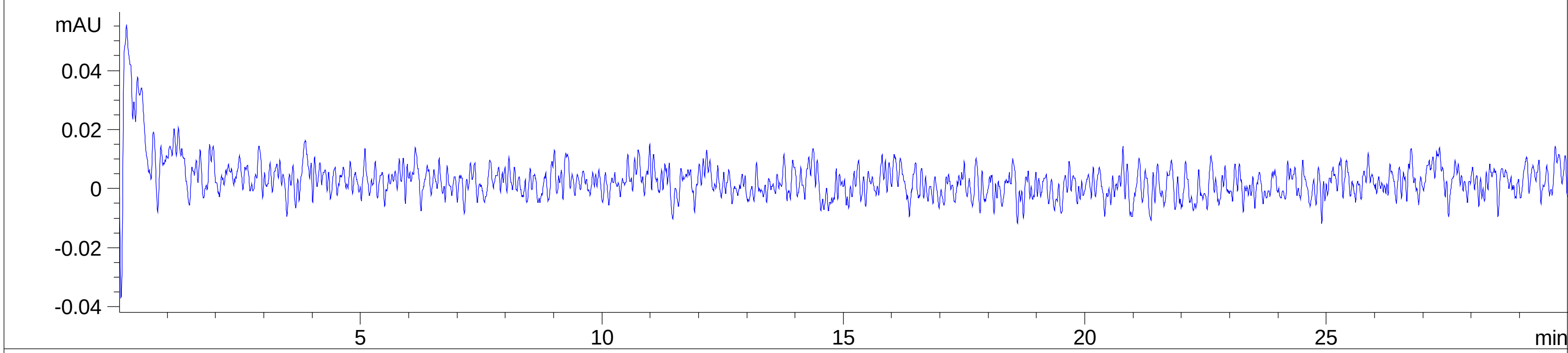

DAD1 C, Sig=282,8 Ref=360,100 (FITOHORMTMG\FITOHOR GABY Y ALE 30-11-2020 2012-10-03 09-08-53\006-0601.D)

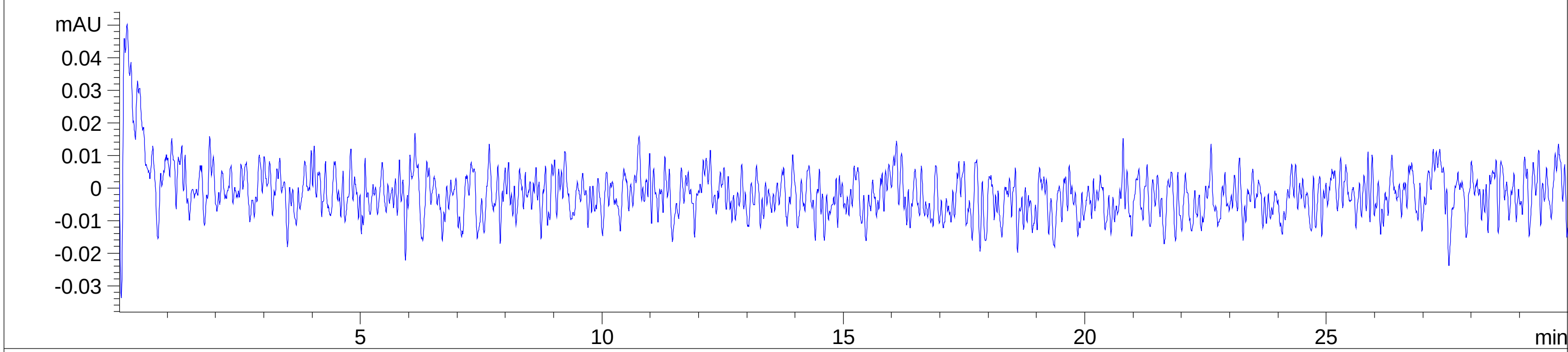

DAD1 D, Sig=254,16 Ref=360,100 (FITOHORMTM...ITOHOR GABY Y ALE 30-11-2020 2012-10-03 09-08-53\006-0601.D)

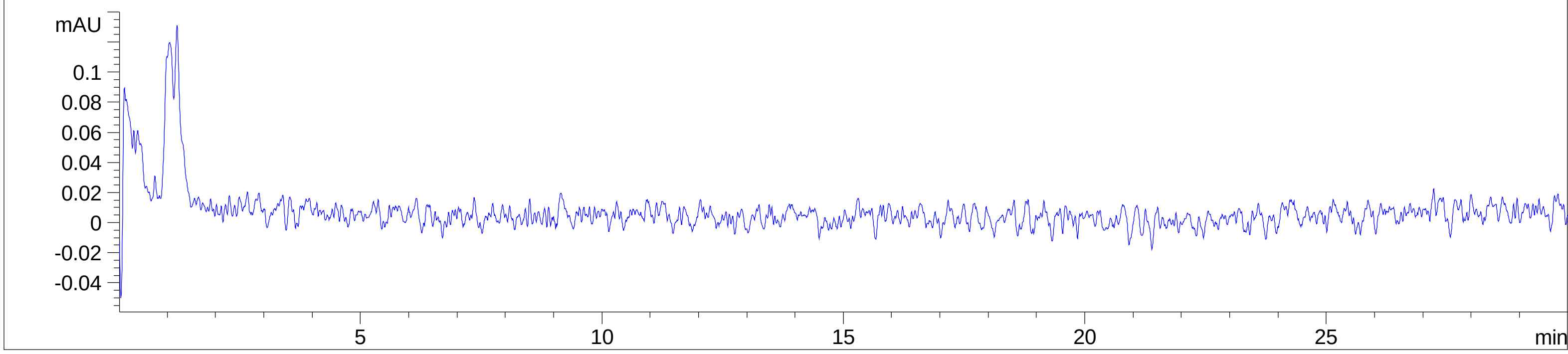

===

Area Percent Report

===

Sorted By : Signal
Multiplier: : 1.0000
Dilution: : 1.0000
Use Multiplier & Dilution Factor with ISTDs

Signal 1: DAD1 A, Sig=220,4 Ref=360,100

Peak #	RetTime [min]	Type	Width [min]	Area [mAU*s]	Height [mAU]	Area %
1	1.015	VV	0.2541	27.23650	1.55670	100.0000

Totals : 27.23650 1.55670

Signal 2: DAD1 B, Sig=280,16 Ref=360,100

Signal 3: DAD1 C, Sig=282,8 Ref=360,100

Signal 4: DAD1 D, Sig=254,16 Ref=360,100

===

*** End of Report ***
