# Supplementary material for: Influence of Silver Nanoparticles (AgNPs) on Vegetative Growth and Concentrations of Nutrients and Phytohormones in Tomato
Source: Plants (Basel). 2026 Jan 28;15(3):405. doi: 10.3390/plants15030405 (PMC12899181; doi:10.3390/plants15030405)
Supplement: Supplementary file 1 [file plants-15-00405-s001.zip › S1. HPLC Analysis (plants-4015186)/Phytohormone standards/IAA.pdf]

Data File C:\CHEM32\...ORMTMG\FITOHOR GABY Y ALE 30-11-2020 2012-10-03 09-08-53\003-0301.D
Sample Name: INDOLACETICO

===

Acq. Operator : TMG   Seq. Line : 3
Acq. Instrument : Instrument 1   Location : Vial 3
Injection Date : 10/3/2012 10:43:36 AM   Inj : 1
Inj Volume : 200.0 µl
Different Inj Volume from Sequence !   Actual Inj Volume : 20.0 µl
Acq. Method : C:\CHEM32\1\DATA\FITOHORMTMG\FITOHOR GABY Y ALE 30-11-2020 2012-10-03 09-08-53\FITOHORMONAS DR SOTO.M
Last changed : 8/14/2013 11:13:25 AM by TMG
Analysis Method : C:\CHEM32\1\METHODS\LAVADO COLUMNA ACET.M
Last changed : 7/27/2013 11:58:00 AM by TMG

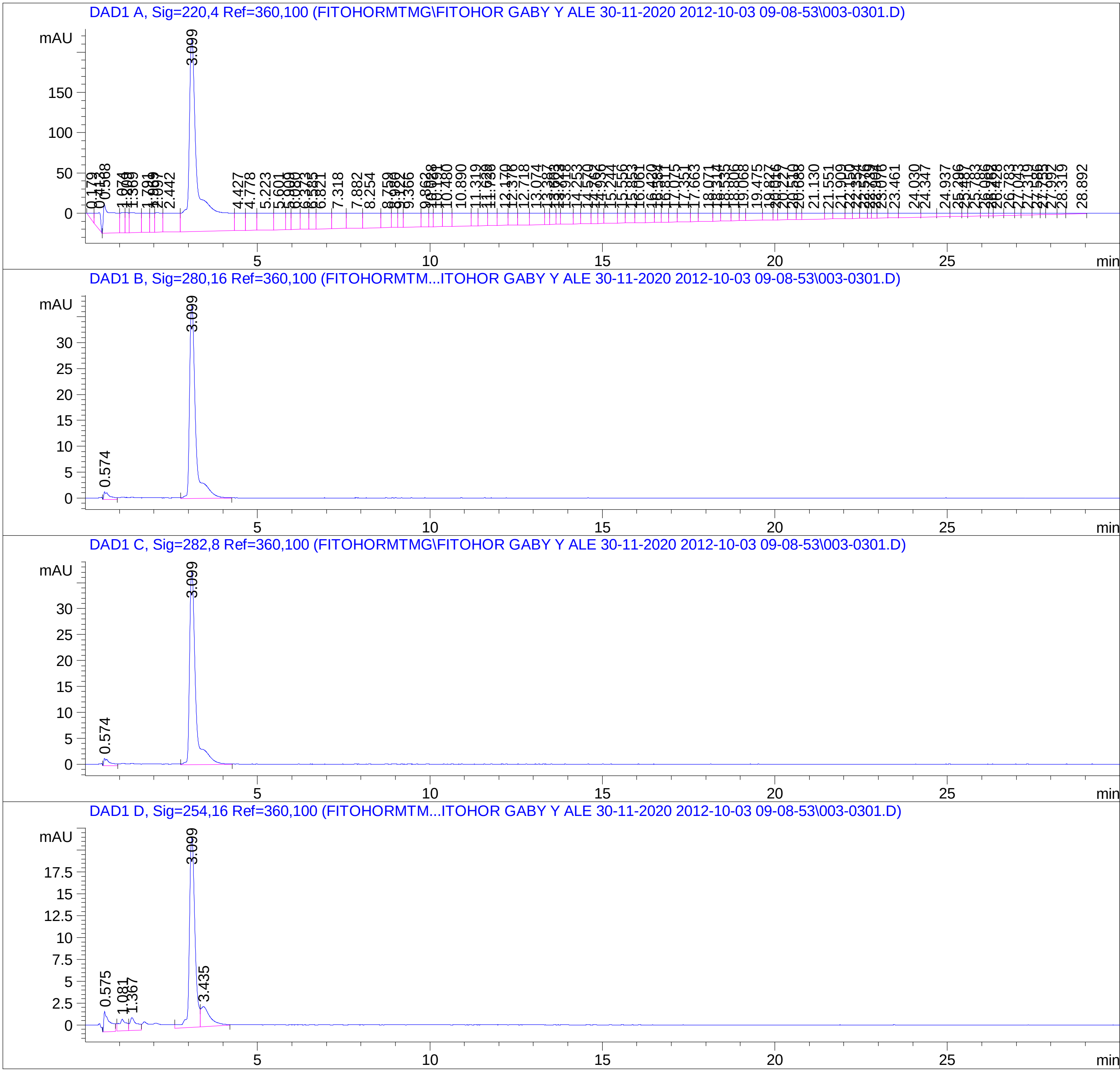

===

Area Percent Report

===

Sorted By : Signal
Multiplier: : 1.0000
Dilution: : 1.0000
Use Multiplier & Dilution Factor with ISTDs

Signal 1: DAD1 A, Sig=220,4 Ref=360,100

Peak #	RetTime [min]	Type	Width [min]	Area [mAU*s]	Height [mAU]	Area %
1	0.179	BV	0.1680	82.63705	7.41879	0.3307
2	0.413	VV	0.1350	209.05197	20.83956	0.8366
3	0.568	VV	0.2611	754.27289	36.19141	3.0184
4	1.074	VV	0.1250	235.40117	24.74927	0.9420
5	1.209	VV	0.1030	179.62128	24.64651	0.7188
6	1.369	VV	0.2750	526.36389	24.86495	2.1064
7	1.791	VV	0.1852	344.10651	24.00457	1.3770
8	1.969	VV	0.1158	196.58203	23.93436	0.7867
9	2.097	VV	0.1882	336.94370	23.94169	1.3484
10	2.442	VV	0.3687	715.14282	23.38758	2.8618
11	3.099	VV	0.2646	4653.10400	239.56856	18.6207
12	4.427	VV	0.2619	421.59869	21.77185	1.6871
13	4.778	VV	0.2433	423.49945	21.35704	1.6947
14	5.223	VV	0.3536	610.71643	20.98148	2.4439
15	5.601	VV	0.2630	427.46118	20.69121	1.7106
16	5.909	VV	0.1341	185.16438	20.36187	0.7410
17	6.080	VV	0.2147	317.69904	20.28204	1.2714
18	6.373	VV	0.2217	308.79111	20.04976	1.2357
19	6.585	VV	0.1784	256.00363	19.86290	1.0245
20	6.821	VV	0.3389	526.88885	19.64338	2.1085
21	7.318	VV	0.3210	474.33438	19.28270	1.8982
22	7.882	VV	0.3770	540.23663	18.81220	2.1619
23	8.254	VV	0.3831	580.67737	18.45238	2.3237
24	8.759	VV	0.2556	336.96338	18.07000	1.3485
25	8.980	VV	0.1548	188.11646	17.87311	0.7528
26	9.122	VV	0.1338	175.98016	17.72660	0.7042
27	9.366	VV	0.3720	540.43701	17.51393	2.1627
28	9.862	VV	0.1946	229.63667	17.10762	0.9190
29	10.028	VV	0.1108	134.83705	16.96031	0.5396
30	10.191	VV	0.2077	264.60864	16.80963	1.0589
31	10.480	VV	0.2162	267.93811	16.61072	1.0722
32	10.890	VV	0.4213	546.00891	16.27964	2.1850
33	11.319	VV	0.1619	182.20618	15.87436	0.7291
34	11.629	VV	0.2170	266.20251	15.61011	1.0653
35	11.736	VV	0.2119	250.38245	15.54823	1.0020
36	12.170	VV	0.2411	287.10327	15.14781	1.1489
37	12.376	VV	0.2180	247.32973	15.02584	0.9898
38	12.718	VV	0.2612	311.02762	14.68022	1.2447

Peak #	RetTime [min]	Type	Width [min]	Area [mAU*s]	Height [mAU]	Area %
39	13.074	VV	0.3146	371.66162	14.42373	1.4873
40	13.387	VV	0.1240	123.00693	14.06721	0.4922
41	13.603	VV	0.1452	151.75024	13.91730	0.6073
42	13.723	VV	0.1176	116.02250	13.86969	0.4643
43	13.918	VV	0.2724	292.77255	13.75264	1.1716
44	14.253	VV	0.1771	173.58603	13.40702	0.6947
45	14.570	VV	0.2398	238.06892	13.23842	0.9527
46	14.762	VV	0.1631	152.60092	12.98264	0.6107
47	14.936	VV	0.1481	133.71432	12.78153	0.5351
48	15.244	VV	0.3041	296.40393	12.61373	1.1861
49	15.556	VV	0.1991	168.20757	12.33157	0.6731
50	15.853	VV	0.2451	199.49553	12.05498	0.7983
51	16.061	VV	0.2417	212.66110	11.94505	0.8510
52	16.420	VV	0.2139	187.90903	11.54734	0.7520
53	16.584	VV	0.1554	132.49744	11.41531	0.5302
54	16.811	VV	0.1738	144.37273	11.24487	0.5777
55	17.075	VV	0.1889	159.21548	10.99934	0.6371
56	17.351	VV	0.2969	252.96443	10.81555	1.0123
57	17.663	VV	0.1910	143.03259	10.49633	0.5724
58	18.071	VV	0.3325	251.94398	10.18724	1.0082
59	18.314	VV	0.1994	134.52031	9.97102	0.5383
60	18.535	VV	0.2419	182.50507	9.77374	0.7303
61	18.806	VV	0.1803	132.80089	9.54810	0.5314
62	19.068	VV	0.1585	105.42869	9.28638	0.4219
63	19.475	VV	0.3578	262.45917	9.01160	1.0503
64	19.827	VV	0.2371	156.71873	8.66183	0.6272
65	20.016	VV	0.1154	69.42540	8.48860	0.2778
66	20.227	VV	0.2267	140.46167	8.33123	0.5621
67	20.510	VV	0.2017	126.66448	8.13212	0.5069
68	20.688	VV	0.1257	74.58471	7.93092	0.2985
69	21.130	VV	0.4753	301.55270	7.59407	1.2067
70	21.551	VV	0.1873	99.11172	7.26077	0.3966
71	21.909	VV	0.2785	146.87376	6.95008	0.5878
72	22.150	VV	0.1692	89.30585	6.71227	0.3574
73	22.354	VV	0.1563	79.67238	6.53250	0.3188
74	22.576	VV	0.1866	87.83630	6.46045	0.3515
75	22.739	VV	0.0925	41.55600	6.17359	0.1663
76	22.904	VV	0.1281	57.50200	6.09578	0.2301
77	23.076	VV	0.2433	113.27386	5.97106	0.4533
78	23.461	VV	0.2345	101.06970	5.71056	0.4045
79	24.030	VV	0.4801	202.84134	5.16558	0.8117
80	24.347	VB	0.3421	133.69734	4.90338	0.5350
81	24.937	BV	0.2827	99.46985	4.38483	0.3981
82	25.296	VV	0.2658	85.38433	4.08626	0.3417
83	25.487	VV	0.1495	39.98211	3.90471	0.1600
84	25.783	VV	0.2781	80.43513	3.69256	0.3219
85	26.086	VV	0.1948	49.95805	3.41322	0.1999
86	26.262	VV	0.1086	24.01762	3.23744	0.0961
87	26.428	VV	0.2406	57.66861	3.16501	0.2308
88	26.783	VV	0.2322	51.77145	2.87286	0.2072

Peak #	RetTime [min]	Type	Width [min]	Area [mAU*s]	Height [mAU]	Area %
89	27.043	VV	0.1585	30.13446	2.61409	0.1206
90	27.319	VV	0.2490	44.94801	2.46186	0.1799
91	27.586	VV	0.1935	30.66730	2.18852	0.1227
92	27.755	VV	0.1235	17.53380	2.01573	0.0702
93	27.992	VV	0.2442	35.40528	1.84264	0.1417
94	28.319	VV	0.2181	23.31933	1.58095	0.0933
95	28.892	VV	0.4735	41.39917	1.04667	0.1657

Totals : 2.49889e4 1417.19870

Signal 2: DAD1 B, Sig=280,16 Ref=360,100

Peak #	RetTime [min]	Type	Width [min]	Area [mAU*s]	Height [mAU]	Area %
1	0.574	VV	0.1525	17.42189	1.46782	3.7872
2	3.099	VB	0.1760	442.60355	37.40549	96.2128

Totals : 460.02543 38.87331

Signal 3: DAD1 C, Sig=282,8 Ref=360,100

Peak #	RetTime [min]	Type	Width [min]	Area [mAU*s]	Height [mAU]	Area %
1	0.574	VV	0.1526	17.03961	1.43412	3.7134
2	3.099	VB	0.1760	441.82516	37.35737	96.2866

Totals : 458.86477 38.79149

Signal 4: DAD1 D, Sig=254,16 Ref=360,100

Peak #	RetTime [min]	Type	Width [min]	Area [mAU*s]	Height [mAU]	Area %
1	0.575	VB	0.1491	27.21936	2.34994	7.7898
2	1.081	BV	0.1950	20.10154	1.32463	5.7528
3	1.367	VV	0.1857	20.06505	1.46617	5.7424
4	3.099	BV	0.1657	238.09547	21.74952	68.1398
5	3.435	VB	0.2713	43.94071	2.29547	12.5753

Totals : 349.42214 29.18572

===

*** End of Report ***
