# Supplementary material for: Influence of Silver Nanoparticles (AgNPs) on Vegetative Growth and Concentrations of Nutrients and Phytohormones in Tomato
Source: Plants (Basel). 2026 Jan 28;15(3):405. doi: 10.3390/plants15030405 (PMC12899181; doi:10.3390/plants15030405)
Supplement: Supplementary file 1 [file plants-15-00405-s001.zip › S1. HPLC Analysis (plants-4015186)/Phytohormone standards/KIN.pdf]

===

```
Acq. Operator   : TMG                              Seq. Line :   7
Acq. Instrument : Instrument 1                      Location : Vial 7
Injection Date  : 10/3/2012 12:49:37 PM                  Inj :   1
                                                  Inj Volume : 200.0 µl
Different Inj Volume from Sequence !       Actual Inj Volume : 20.0 µl
Acq. Method     : C:\CHEM32\1\DATA\FITOHORMTMG\FITOHOR GABY Y ALE 30-11-2020 2012-10-03 09-08-
                  53\FITOHORMONAS DR SOTO.M
Last changed    : 8/14/2013 11:13:25 AM by TMG
Analysis Method : C:\CHEM32\1\METHODS\LAVADO COLUMNA ACET.M
Last changed    : 10/21/2012 12:24:49 PM by TMG
                  (modified after loading)
```

DAD1 A, Sig=220,4 Ref=360,100 (FITOHORMTMG\FITOHOR GABY Y ALE 30-11-2020 2012-10-03 09-08-53\007-0701.D)

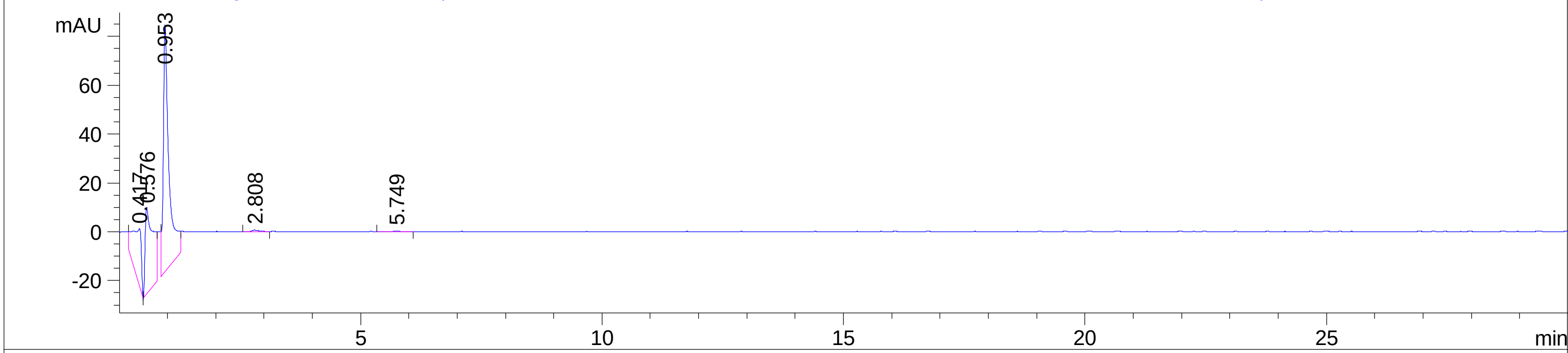

DAD1 B, Sig=280,16 Ref=360,100 (FITOHORMTM...ITOHOR GABY Y ALE 30-11-2020 2012-10-03 09-08-53\007-0701.D)

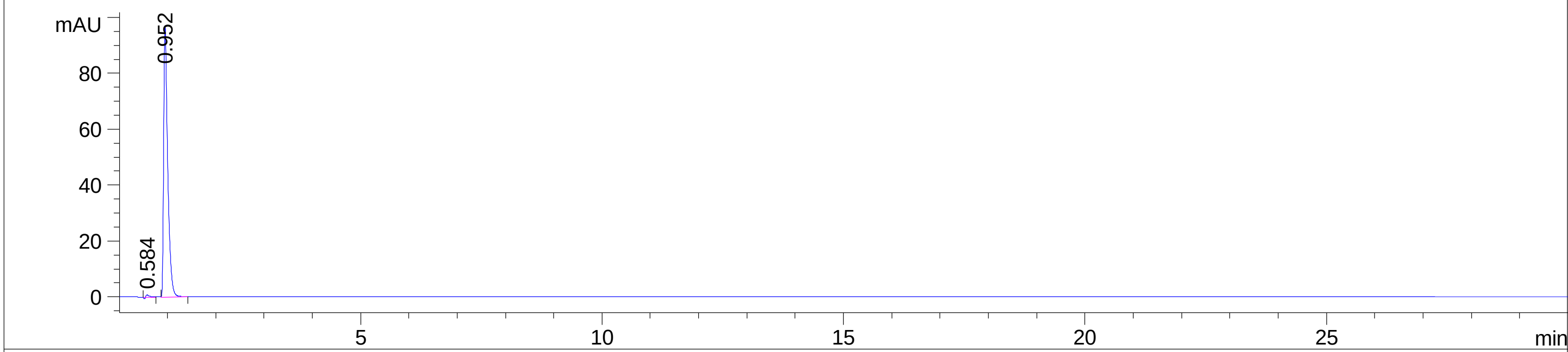

DAD1 C, Sig=282,8 Ref=360,100 (FITOHORMTMG\FITOHOR GABY Y ALE 30-11-2020 2012-10-03 09-08-53\007-0701.D)

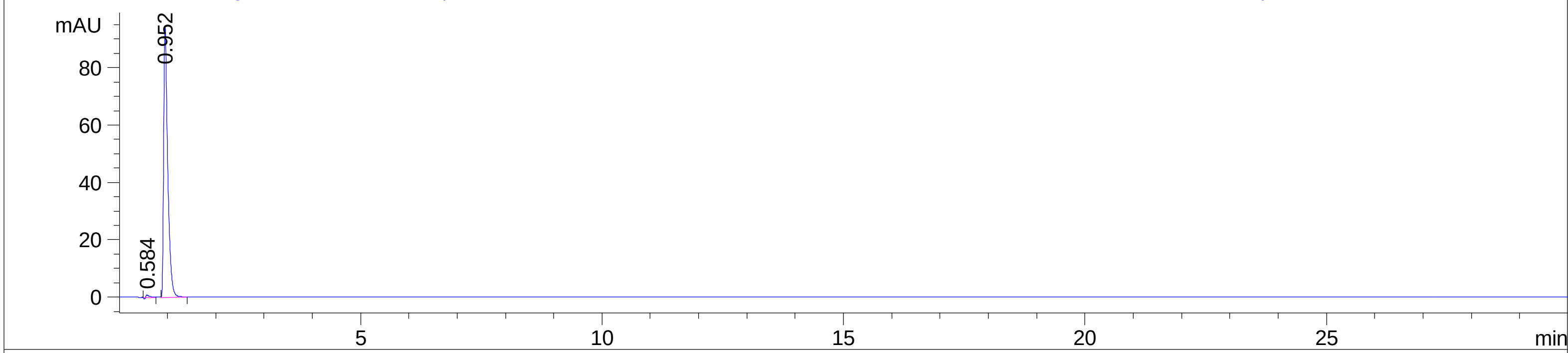

DAD1 D, Sig=254,16 Ref=360,100 (FITOHORMTM...ITOHOR GABY Y ALE 30-11-2020 2012-10-03 09-08-53\007-0701.D)

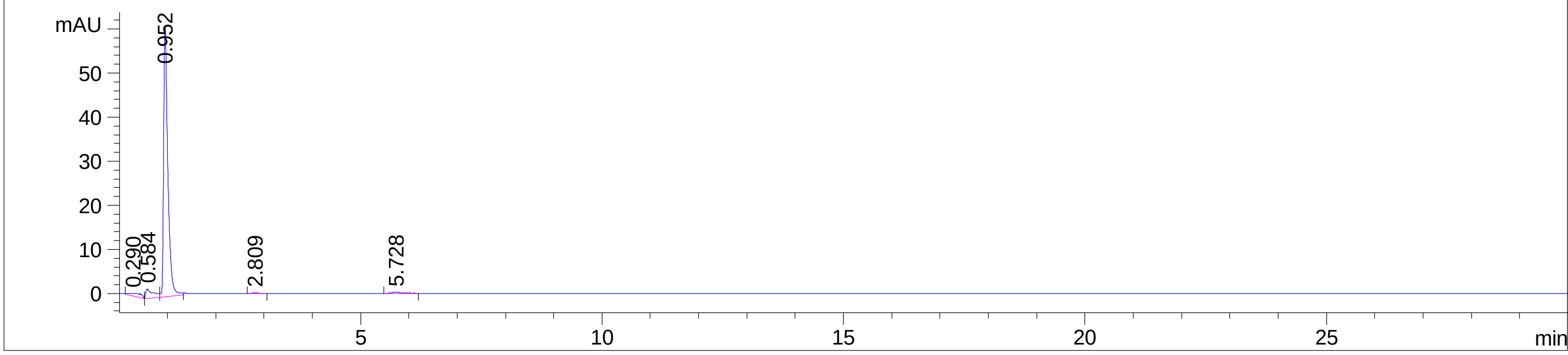

===

Area Percent Report

===

Sorted By : Signal
Multiplier: : 1.0000
Dilution: : 1.0000
Use Multiplier & Dilution Factor with ISTDs

Signal 1: DAD1 A, Sig=220,4 Ref=360,100

Peak #	RetTime [min]	Type	Width [min]	Area [mAU*s]	Height [mAU]	Area %
1	0.417	BV	0.1579	270.33969	22.87482	17.9608
2	0.576	VB	0.1553	405.16309	34.93147	26.9181
3	0.953	BB	0.1155	818.27344	99.99034	54.3642
4	2.808	BB	0.1728	7.59421	6.47833e-1	0.5045
5	5.749	VB	0.2233	3.79884	2.26947e-1	0.2524

Totals : 1505.16926 158.67141

Signal 2: DAD1 B, Sig=280,16 Ref=360,100

Peak #	RetTime [min]	Type	Width [min]	Area [mAU*s]	Height [mAU]	Area %
1	0.584	BB	0.0803	4.95568	8.99403e-1	0.8576
2	0.952	BB	0.0885	572.90759	97.61751	99.1424

Totals : 577.86327 98.51691

Signal 3: DAD1 C, Sig=282,8 Ref=360,100

Peak #	RetTime [min]	Type	Width [min]	Area [mAU*s]	Height [mAU]	Area %
1	0.584	BB	0.0796	4.88475	8.95817e-1	0.8677
2	0.952	BB	0.0884	558.05859	95.12027	99.1323

Totals : 562.94334 96.01609

Signal 4: DAD1 D, Sig=254,16 Ref=360,100

Peak #	RetTime [min]	Type	Width [min]	Area [mAU*s]	Height [mAU]	Area %
1	0.290	BV	0.2687	12.82154	5.78280e-1	3.1006
2	0.584	VV	0.1374	22.57860	2.17000	5.4601
3	0.952	VB	0.0905	373.00937	61.71595	90.2037
4	2.809	BB	0.1445	8.57445e-1	8.89090e-2	0.2074
5	5.728	BB	0.2578	4.25202	2.41092e-1	1.0283
Totals :				413.51898	64.79422	

==

*** End of Report ***
