# Supplementary material for: Influence of Silver Nanoparticles (AgNPs) on Vegetative Growth and Concentrations of Nutrients and Phytohormones in Tomato
Source: Plants (Basel). 2026 Jan 28;15(3):405. doi: 10.3390/plants15030405 (PMC12899181; doi:10.3390/plants15030405)
Supplement: Supplementary file 1 [file plants-15-00405-s001.zip › S1. HPLC Analysis (plants-4015186)/Phytohormone standards/SA.pdf]

Data File C:\CHEM32\...ORMTMG\FITOHOR GABY Y ALE 30-11-2020 2012-10-03 09-08-53\005-0501.D
Sample Name: SALICILICO

===

Acq. Operator	: TMG	Seq. Line :	5
Acq. Instrument	: Instrument 1	Location :	Vial 5
Injection Date	: 10/3/2012 11:46:36 AM	Inj :	1
		Inj Volume :	200.0 µl
Different Inj Volume from Sequence !		Actual Inj Volume :	20.0 µl

Acq. Method : C:\CHEM32\1\DATA\FITOHORMTMG\FITOHOR GABY Y ALE 30-11-2020 2012-10-03 09-08-53\FITOHORMONAS DR SOTO.M
Last changed : 8/14/2013 11:13:25 AM by TMG
Analysis Method : C:\CHEM32\1\METHODS\LAVADO COLUMNA ACET.M
Last changed : 7/27/2013 11:58:00 AM by TMG

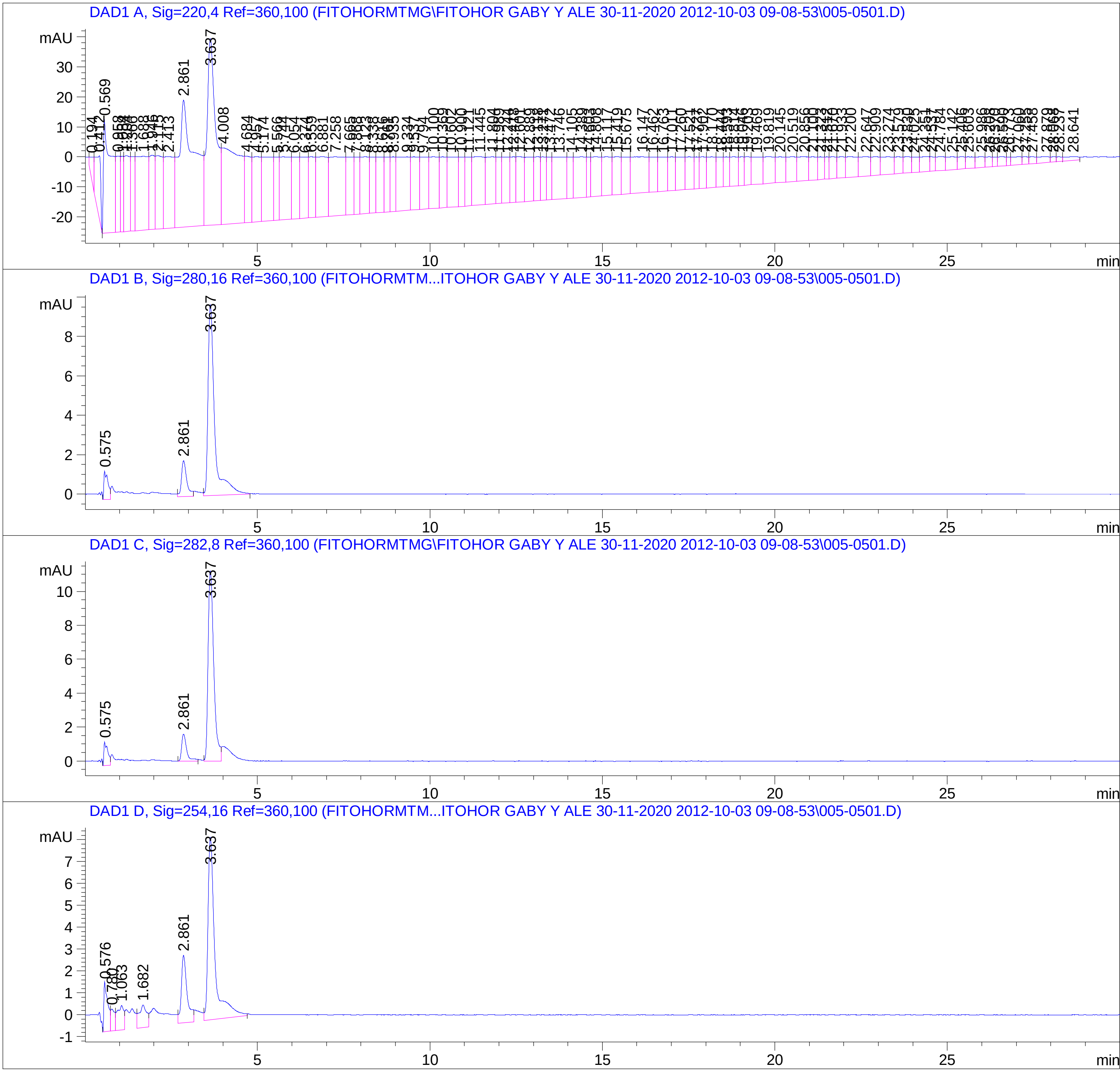

===

Area Percent Report

===

Sorted By : Signal
Multiplier: : 1.0000
Dilution: : 1.0000
Use Multiplier & Dilution Factor with ISTDs

Signal 1: DAD1 A, Sig=220,4 Ref=360,100

Peak #	RetTime [min]	Type	Width [min]	Area [mAU*s]	Height [mAU]	Area %
1	0.194	BV	0.1242	65.23337	7.30479	0.2774
2	0.412	VV	0.1373	207.71811	20.66633	0.8832
3	0.569	VV	0.1998	587.80914	37.72097	2.4994
4	0.958	VV	0.1333	237.43741	25.33759	1.0096
5	1.084	VV	0.0762	133.98923	25.12176	0.5697
6	1.204	VV	0.1553	288.72223	25.27826	1.2277
7	1.366	VV	0.1154	198.54012	24.80940	0.8442
8	1.688	VV	0.2983	579.47937	24.64540	2.4640
9	1.946	VV	0.1601	280.63162	24.79117	1.1933
10	2.115	VV	0.2053	346.01550	24.42772	1.4713
11	2.413	VV	0.2650	477.68820	23.91646	2.0312
12	2.861	VV	0.4132	1368.35522	42.32382	5.8184
13	3.637	VV	0.2587	1144.76758	62.20002	4.8677
14	4.008	VV	0.4948	952.28876	25.43705	4.0493
15	4.684	VV	0.1771	290.05362	22.09754	1.2333
16	4.957	VV	0.2142	349.04227	21.63676	1.4842
17	5.174	VV	0.2641	463.04880	21.42961	1.9689
18	5.566	VV	0.1445	206.74574	21.06633	0.8791
19	5.744	VV	0.2611	436.94858	20.97008	1.8580
20	6.054	VV	0.1838	300.45929	20.64314	1.2776
21	6.374	VV	0.2005	298.03308	20.38864	1.2673
22	6.559	VV	0.1897	259.99631	20.26631	1.1055
23	6.881	VV	0.3059	439.31787	20.02704	1.8680
24	7.258	VV	0.3901	599.75562	19.65032	2.5502
25	7.665	VV	0.1771	266.05728	19.26893	1.1313
26	7.868	VV	0.1582	206.45883	19.08929	0.8779
27	8.123	VV	0.2233	303.90369	18.92816	1.2922
28	8.338	VV	0.1417	204.36339	18.67235	0.8690
29	8.616	VV	0.1859	271.82443	18.44498	1.1558
30	8.761	VV	0.1494	190.72415	18.33152	0.8110
31	8.935	VV	0.1456	189.64430	18.19907	0.8064
32	9.334	VV	0.3084	447.41168	17.85414	1.9025
33	9.537	VV	0.2086	279.91327	17.68583	1.1902
34	9.794	VV	0.1992	273.82745	17.44258	1.1644
35	10.100	VV	0.2335	309.27307	17.22036	1.3151
36	10.369	VV	0.1790	231.19289	16.96544	0.9831
37	10.602	VB	0.2460	332.95178	16.74062	1.4158
38	10.900	BV	0.1569	182.11584	16.47692	0.7744

Peak #	RetTime [min]	Type	Width [min]	Area [mAU*s]	Height [mAU]	Area %
39	11.121	VV	0.1710	199.41171	16.27013	0.8479
40	11.445	VV	0.3117	371.64594	16.07581	1.5803
41	11.804	VV	0.2426	295.15732	15.75316	1.2551
42	11.989	VV	0.1402	157.22386	15.52427	0.6685
43	12.244	VV	0.1832	211.85388	15.33030	0.9008
44	12.418	VV	0.1366	157.13792	15.20609	0.6682
45	12.601	VV	0.2103	233.02557	15.07468	0.9909
46	12.889	VV	0.2231	229.38235	14.78053	0.9754
47	13.118	VV	0.1626	176.74658	14.66842	0.7516
48	13.271	VV	0.1364	144.46416	14.47973	0.6143
49	13.472	VV	0.1446	142.51489	14.26138	0.6060
50	13.746	VV	0.3096	360.63943	14.04415	1.5335
51	14.105	VV	0.1472	156.89885	13.73890	0.6672
52	14.389	VV	0.2996	306.07355	13.54567	1.3015
53	14.603	VV	0.0991	92.16105	13.25853	0.3919
54	14.808	VV	0.2475	250.35509	13.18554	1.0645
55	15.117	VV	0.2383	241.78122	12.92291	1.0281
56	15.419	VV	0.2254	196.84291	12.65721	0.8370
57	15.675	VV	0.1935	190.37701	12.37295	0.8095
58	16.147	VV	0.4130	392.18860	12.00837	1.6676
59	16.462	VV	0.1979	174.28346	11.69055	0.7411
60	16.763	VV	0.2200	206.02049	11.44592	0.8760
61	17.011	VV	0.1889	151.35751	11.25261	0.6436
62	17.260	VV	0.2051	178.48921	11.01039	0.7590
63	17.521	VV	0.2137	171.91373	10.80211	0.7310
64	17.727	VV	0.1259	96.40928	10.62201	0.4099
65	17.902	VV	0.1732	130.77000	10.50419	0.5561
66	18.170	VV	0.2136	172.05179	10.26078	0.7316
67	18.444	VV	0.1629	125.97070	10.00324	0.5356
68	18.593	VV	0.1440	105.02137	9.88057	0.4466
69	18.814	VV	0.2010	145.99426	9.73271	0.6208
70	19.046	VV	0.1454	97.49127	9.52458	0.4145
71	19.203	VV	0.1689	115.32254	9.41536	0.4904
72	19.469	VV	0.2655	182.52690	9.11789	0.7761
73	19.819	VV	0.2728	189.79012	8.90007	0.8070
74	20.145	VV	0.2342	154.14354	8.55487	0.6554
75	20.519	VV	0.2542	154.94270	8.28361	0.6588
76	20.856	VV	0.2580	166.18347	7.94645	0.7066
77	21.100	VV	0.2074	120.17129	7.72947	0.5110
78	21.323	VV	0.1712	92.41502	7.53042	0.3930
79	21.512	VV	0.1118	58.02465	7.37134	0.2467
80	21.670	VV	0.1852	99.33826	7.28025	0.4224
81	21.933	VV	0.2031	101.33179	7.07367	0.4309
82	22.200	VV	0.2813	150.00839	6.80293	0.6379
83	22.647	VV	0.2615	136.44562	6.43192	0.5802
84	22.909	VV	0.2520	106.83962	6.23205	0.4543
85	23.274	VV	0.3064	142.28094	5.92001	0.6050
86	23.579	VV	0.2022	83.12332	5.63146	0.3535
87	23.830	VV	0.1972	80.80862	5.50535	0.3436
88	24.025	VV	0.1703	68.63163	5.18933	0.2918

Peak #	RetTime [min]	Type	Width [min]	Area [mAU*s]	Height [mAU]	Area %
89	24.351	VV	0.2339	88.40511	5.00841	0.3759
90	24.537	VV	0.1353	45.57479	4.77700	0.1938
91	24.784	VV	0.2365	79.10111	4.60206	0.3363
92	25.142	VV	0.2774	89.00883	4.26687	0.3785
93	25.406	VV	0.1926	57.70194	4.04047	0.2454
94	25.603	VV	0.2074	62.59871	3.85635	0.2662
95	25.986	VV	0.2165	59.99370	3.59862	0.2551
96	26.208	VV	0.1665	42.08740	3.35076	0.1790
97	26.370	VV	0.1321	28.88126	3.17675	0.1228
98	26.590	VV	0.2208	46.55033	3.03729	0.1979
99	26.773	VV	0.1111	21.41455	2.80526	0.0911
100	27.060	VV	0.2591	51.62449	2.62884	0.2195
101	27.255	VV	0.1680	28.67702	2.46076	0.1219
102	27.458	VV	0.1824	30.22909	2.31446	0.1285
103	27.879	VV	0.3116	46.99440	1.93319	0.1998
104	28.068	VV	0.1292	17.53467	1.74476	0.0746
105	28.237	VV	0.1431	16.60247	1.60006	0.0706
106	28.641	VV	0.3647	38.85075	1.33049	0.1652
Totals :				2.35176e4	1452.81397	

Signal 2: DAD1 B, Sig=280,16 Ref=360,100

Peak #	RetTime [min]	Type	Width [min]	Area [mAU*s]	Height [mAU]	Area %
1	0.575	VV	0.1071	11.73999	1.44072	7.1987
2	2.861	BB	0.1640	19.98270	1.82086	12.2530
3	3.637	BB	0.2000	131.36183	9.69693	80.5483
Totals :				163.08452	12.95852	

Signal 3: DAD1 C, Sig=282,8 Ref=360,100

Peak #	RetTime [min]	Type	Width [min]	Area [mAU*s]	Height [mAU]	Area %
1	0.575	VV	0.1035	10.87937	1.38772	6.9590
2	2.861	BB	0.1542	15.99789	1.57931	10.2331
3	3.637	BV	0.1768	129.45766	11.20504	82.8079
Totals :				156.33491	14.17207	

Signal 4: DAD1 D, Sig=254,16 Ref=360,100

Peak #	RetTime [min]	Type	Width [min]	Area [mAU*s]	Height [mAU]	Area %
1	0.576	VV	0.1080	18.20533	2.21301	8.5732
2	0.780	VV	0.1013	7.66688	1.00174	3.6105
3	1.063	VV	0.1772	14.96713	1.11081	7.0483
4	1.682	VV	0.2029	15.73850	1.02639	7.4115
5	2.861	BB	0.1740	36.47911	3.08413	17.1786
6	3.637	BB	0.2107	119.29543	8.34968	56.1781
Totals :				212.35239	16.78575	

*** End of Report ***
